# Palladium-catalyzed Suzuki-Miyaura coupling of thioureas or thioamides

**DOI:** 10.1038/s41467-019-13701-5

**Published:** 2019-12-13

**Authors:** Shaoyu Mai, Wendong Li, Xue Li, Yingwei Zhao, Qiuling Song

**Affiliations:** 1 Institute of Next Generation Matter Transformation, College of Chemical Engineering and College of Material Sciences Engineering at Huaqiao University, 668 Jimei Boulevard, Xiamen, Fujian 361021 China; 2 Key Laboratory of Molecule Synthesis and Function Discovery, Fujian Province University, College of Chemistry at Fuzhou University, Fuzhou, Fujian 350108 China

**Keywords:** Homogeneous catalysis, Synthetic chemistry methodology, Synthetic chemistry methodology

## Abstract

Cross-coupling reactions involving metal carbene intermediates play an increasingly important role in C–C bond formation. Expanding the carbene precursors to a broader range of starting materials and more diverse products is an ongoing challenge in synthetic organic chemistry. Herein, we report a Suzuki-Miyaura coupling reaction of in situ-generated Pd–carbene complexes via desulfurization of thioureas or thioamides. This strategy enables the preparation of a broad array of substituted amidinium salts and unsymmetrical diaryl ketones. The reaction is readily scalable, compatible with bromo groups on aromatic rings, tolerant to moisture and air and has a broad substrate scope. Furthermore, a single crystal structure of Pd-diaminocarbene complex is obtained and proven to be the key intermediate in both catalytic and stoichiometric reactions. Preliminary mechanistic studies demonstrate the dual role of the silver salt as a desulfurating reagent assisting the elimination of sulfur and as oxidant facilitating the Pd^II^/Pd^0^/Pd^II^ catalytic cycle.

## Abstract (web summary)

Alternative carbene precursors for metal-catalyzed cross coupling may expand the portfolio of methods for C-C bond construction. Here, the authors report a Suzuki−Miyaura coupling of Pd−carbene complexes formed by desulfurization of thioureas or thioamides and affording a broad array of amidinium salts and diaryl ketones.

## Introduction

**Fig. 1:**
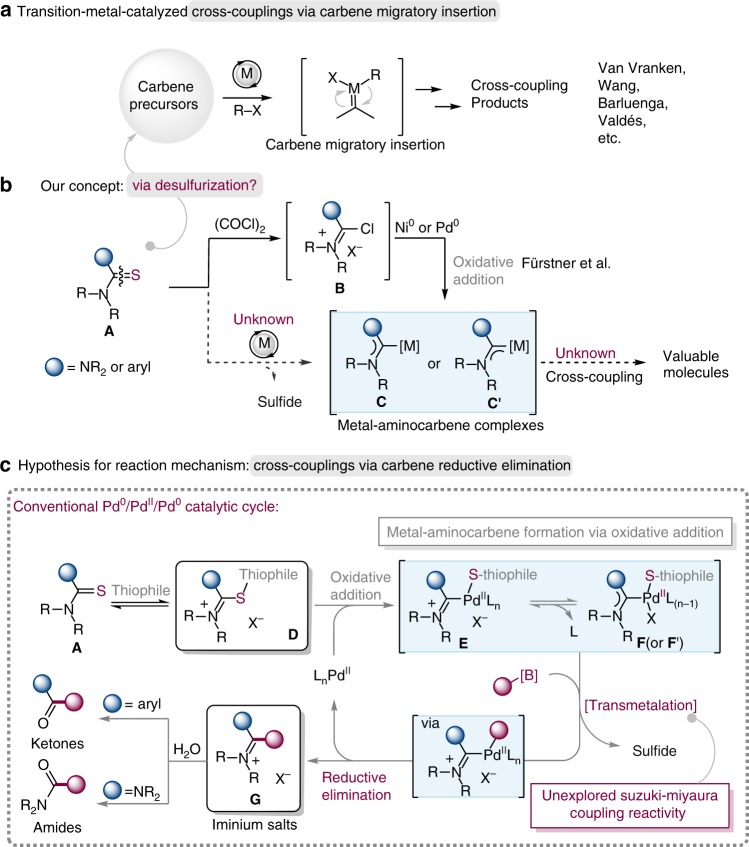
Direct desulfurization for the generation of carbenes. **a** Transition-metal-catalyzed carbene coupling reactions by carbene migratory insertion. M = metal. **b** Our concept for the catalytical generation of metal-aminocarbene from thioamide derivatives **A** and its crossing coupling reactions. **c** Hypothesis of mechanism for desulfurative Suzuki–Miyaura coupling reaction based on Pd^0^/Pd^II^/Pd^0^ catalytic cycle by carbene reductive elimination. X^−^  = anion; L = Ligand.

In the past decade, the transition-metal-catalyzed carbene coupling reactions have emerged as one of the most powerful and reliable methods for carbon–carbon bond construction^1–15^. In most of these coupling reactions, diazo compounds were frequently employed to serve as very useful precursors for metal carbene generation, and many elegant transformations have been developed with diazo compounds, as a consequence, more and more chemists have been attracted to devote to this field^10–13^. In spite of the great advance and importance of diazo compounds in carbene coupling reactions, the discovery and development of carbene synthons which possess versatile reactivity and are stable, as well as convenient to prepare is still highly challenging^14,15^. In 2007, Barluenga et al. reported the first palladium-catalyzed carbene cross-coupling with tosylhydrazones (the precursors for *in situ* generated diazo compounds)^2^. Subsequently, they demonstrated a powerful reductive coupling of tosylhydrazones with boronic acids in the absence of a metal catalyst^3^. In addition, the allenyl ketones^5^, ene-yne-ketones^6^, alkynes^7^, cyclopropenes^8^ along with chromium(0) Fischer carbene complexes^9^, have been proven as effective metal carbene precursors in cross-couplings by Wang and co-workers, which have prominently enriched the toolboxes of synthetic chemists (Fig. 1a). In 1993, Kuhn and co-workers^16–18^ reported an efficient method for the synthesis of metal *N*-heterocyclic carbene (NHC) complexes: it involves the synthesis of NHC complexes via desulfurization of imidazole-derived thioureas in the first step, and subsequent coordination to corresponding metal salts. Such desulfurization of thioureas needs stoichiometric amounts of potassium or sodium as desulfurating reagents via the removal of K_2_S or Na_2_S. However, the stoichiometric alkali metals, harsh reaction conditions and limited scope of acyclic thioureas restricted their application in organic synthesis, especially in metal-catalyzed organic reactions. In addition, Yang et al. have recently demonstrated that thioureas can serve as ligands to a variety of different transition-metal-catalyzed reactions, because of the strong coordinative and adsorptive properties of the sulfur atom, and they have also found their application in the total synthesis of natural products^19^. In 2005, Fürstner et al. reported the elegant synthesis of metal-diaminocarbene complexes **C** (from thioureas) or metal-monoaminocarbene complexes **C′** (from thioamides) by oxidative addition of electron-rich metal centers (e.g., Pd^0^, Ni^0^) into the Vilsmeier-type salts **B**, which were prepared from thioamide derivatives **A** and oxalyl chloride (Fig. 1b)^20^. For the reactivity of metal-aminocarbene complexes, Grushin, Yates and Cavell demonstrated that the alkyl/aryl/acyl-carbene reductive elimination from imidazole-derived Pd^II^ heterocylic carbene complexes, as the side reaction in metal-NHC system, could lead to catalyst deactivation along with undesired carbon–carbon bond formation^21,22^, indicating that the reaction of metal-aminocarbene and its ligand prefer carbene reductive elimination to carbene migratory insertion.

**Fig. 2:**
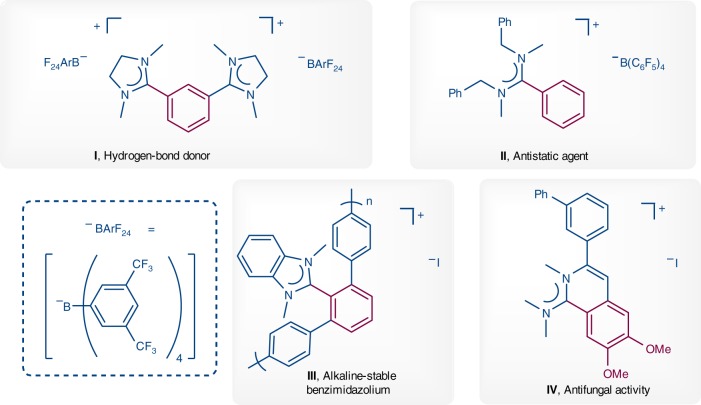
Selected compounds containing amidinium salt unit. The significance of amidinium salts **I**–**IV**.

In continuation of our work on the reactivity of the thioamide-derived substrates^23,24^ and inspired by Kuhn’s NHCs synthesis, we are intrigued by the possibility of the thioamide derivatives (thioureas can be regarded as two parts of thioamide) as metal carbene precursors. The use of thioamide derivatives **A** for the catalytical generation of metal-aminocarbenes through the elimination of sulfide is particularly appealing and is expected to offer great opportunities, especially when it leads to unconventional reaction mechanism and unique retrosynthetic relationships (Fig. 1b). To elaborate the feasibility of our plan, we propose a plausible mechanism depicted in Fig. 1c, which involves Pd^0^/Pd^II^/Pd^0^ catalytic cycle. Generally, the reversible intermediate **D** can be easily generated from thioamide **A** as a result of the coordination of thiophile (or thiophilic auxiliary)^25–29^, which leads to oxidative addition of the C–S bond^30–35^ in the presence of active L_n_Pd^0^, affording the formal sulfur-containing metal-aminocarbene intermediate in equilibrium between the cationic form **E** and the neutral form **F** or **F′** (not shown in Fig. 1c)^20^. The development of a catalytic version of this type of reactions from thioamide derivatives remains a formidable challenge, probably due to the high-energy barrier in desulfurization process, the poor compatibility of desulfurating reagents with substrates and the difficulty in finding a suitable condition and appropriate metal which can not only capture the *in situ*-generated carbene species but also accomplish the catalytic crossing-coupling cycle, meanwhile, the metal catalyst can’t be poisoned by sulfur species. Given the commercial availability, stability, and nontoxicity of organoboron reagents, transition-metal catalyzed Suzuki−Miyaura coupling (SMC) will be an ideal choice for achieving the aforementioned catalytic cycle^36–44^. Thus, the coupling of the Pd-aminocarbene complex with organoboron ^II^compound can in principle produce the iminium salt intermediate **G** via reductive elimination, which will set the stage for the preparation of a broad array of substituted amidinium salts (from thioureas) and unsymmetric ketones (from thioamides). Remarkably, the amidinium salts are prevalent in many functional molecules or materials and bioactive molecules^45–48^ (Fig. 2), such as amidinium salt **I** (a hydrogen-bond donor to activate electron-deficient quinones and a catalyst in a quinone-mediated model synthetic transformation)^45^, amidinium salt **II** (antistatic agent)^46^, **III** (alkaline-stable benzimidazolium in high-performance hydroxide conducting membranes)^47^ and amidinium salt **IV** (antifungal agent)^48^. Moreover, amidinium salts are a well-known subclass of ionic liquids (ILs)^49,50^.

Herein, we now report a successful example of the use of an *in situ-*generated Pd−carbene complex through desulfurization of thioureas or thioamides to accomplish Suzuki−Miyaura coupling reaction to afford a broad array of substituted amidinium salts or valuable diaryl ketones.

## Results

### Synthetic methodology development

Firstly, after detecting the formation of amide phenyl(piperidin-1-yl)methanone (**3**) in 15% yield (Table 1, entry 2) by using di(piperidin-1-yl)methanethione (**1a**) and phenyl boronic acid (**2a**) as the model substrates, we next investigated the effect of Pd catalysts and found that common Pd(PPh_3_)_2_Cl_2_ was the best choice (Table 1, entries 1–5). According to the related reports, the desulfurization of thioamide derivatives were efficiently performed with Ag_2_CO_3_^25^, CuSO_4_•5H_2_O^26^, CoCl_2_•6H_2_O^27^, and PhI(OAc)_2_^28^ by the formation of Ag_2_S, CuS, CoS and elemental sulfur respectively. Screening of common desulfurating reagents showed that Ag_2_CO_3_ exhibited best activity (Table 1, entries 5–9). To facilitate transmetallation of phenyl boronic acid, a series of additional bases was also studied and Na_2_CO_3_ was the optimal one (entries 11–13). The amount of Pd catalyst as well as the silver salt loading and base usage highly affected the efficiency of this transformation. When submitting **1a** and **2a** along with 10 mol% of Pd(PPh_3_)_2_Cl_2_ in trifluoroethanol (TFE, without dehydration) under air atmosphere with 1.5 equivalents of Ag_2_CO_3_, a 93% yield of the amide phenyl(piperidin-1-yl)methanone (**3**) could be obtained (Table 1, entry 16). Silver salt was essential for the success of this reaction (Table 1, entry 17). Based on our proposed mechanism, the hydrolysis of intermediate **G** can provide amide using thiourea as the substrate (Fig. 1c). To capture the intermediate **G**, we tried to add two equivalents of HOTf into the reaction system without workup. Fortunately, we found the amidinium salt **4** with OTf anion was obtained in 98% yield (Table 1, entry 19) and its structure has been unambiguously confirmed by X-ray crystallographic analysis (Table 2, CCDC 1895265).

**Table 1:** Optimization of reaction conditions.


entry	catalyst	additive (equiv)	base (equiv)	solvent	3/4 yield%
1	Pd (PPh_3_)_4_	Ag_2_CO_3_ (3)	NaHCO_3_ (2)	*t*-AmylOH	–^a^
2	Pd (OAc)_2_	Ag_2_CO_3_ (3)	NaHCO_3_ (2)	*t*-AmylOH	15/–
3	PdCl_2_ (MeCN)_2_	Ag_2_CO_3_ (3)	NaHCO_3_(2)	*t*-AmylOH	23/–
4	PdCl_2_ (dppf)	Ag_2_CO_3_ (3)	NaHCO_3_ (2)	*t*-AmylOH	36/–
5	PdCl_2_ (PPh_3_)_2_	Ag_2_CO_3_ (3)	NaHCO_3_ (2)	*t*-AmylOH	60/–
6	PdCl_2_ (PPh_3_)_2_	AgOAc (3)	NaHCO_3_ (2)	*t*-AmylOH	18/–
7	PdCl_2_ (PPh_3_)_2_	CuSO_4_•5H_2_O (3)	NaHCO_3_ (2)	*t*-AmylOH	–^a^
8	PdCl_2_ (PPh_3_)_2_	CoCl_2_•6H_2_O (3)	NaHCO_3_ (2)	*t*-AmylOH	–^a^
9	PdCl_2_ (PPh_3_)_2_	PhI(OAc)_2_ (3)	NaHCO_3_ (2)	*t*-AmylOH	–^a^
10	PdCl_2_ (PPh_3_)_2_	Ag_2_CO_3_ (3)	NaHCO_3_ (2)	*t*-BuOH	36/–
11	PdCl_2_ (PPh_3_)_2_	Ag_2_CO_3_ (3)	NaHCO_3_ (2)	TFE	61/–
12	PdCl_2_ (PPh_3_)_2_	Ag_2_CO_3_ (3)	K_2_CO_3_ (2)	TFE	23/–
13	PdCl_2_ (PPh_3_)_2_	Ag_2_CO_3_ (3)	Na_2_CO_3_ (2)	TFE	68/–
14	PdCl_2_ (PPh_3_)_2_	Ag_2_CO_3_ (3)	Na_2_CO_3_ (1)	TFE	79/–
15	PdCl_2_ (PPh_3_)_2_	Ag_2_CO_3_ (3)	Na_2_CO_3_ (0.5)	TFE	80/–
16	PdCl_2_ (PPh_3_)_2_	Ag_2_CO_3_ (1.5)	Na_2_CO_3_ (0.5)	TFE	93/–
17	PdCl_2_ (PPh_3_)_2_	–	Na_2_CO_3_ (0.5)	TFE	–^a^
18^b^	PdCl_2_ (PPh_3_)_2_	Ag_2_CO_3_ (1.5)	Na_2_CO_3_ (0.5)	TFE	71/–
19^c^	PdCl_2_ (PPh_3_)_2_	Ag_2_CO_3_ (1.5)	Na_2_CO_3_ (0.5)	TFE	–**/(98)**^d^

Reaction conditions: **1a** (0.2 mmol), **2a** (0.4 mmol), Pd catalyst (10 mol%), additive (1.5–3 equiv), base (0.5–2 equiv), solvent (1.5 mL), 4 h, 90 °C, under air. Isolated yield

^a^ No reaction

^b^ PdCl_2_(PPh_3_)_2_ (5 mol%)

^c^ Once the reaction was completed, HOTf (2 equiv) was added

^d^ Amidinium salt **4** was isolated in 98% yield, X = OTf. X^−^ = anion; dppf *=* 1,1′-Bis(diphenylphosphino)ferrocene; *t*-AmylOH = 2-Methyl-2-butanol; TFE = 2,2,2-Trifluoroethanol; HOTf = Trifluoromethanesulfonic acid

**Table 2:** Substrate scopes with respect to the construction of amidinium salts^a^.

^a^Reactions were performed with **1** (0.2 mmol, 1 equiv), **2** (0.4 mmol, 2 equiv), and PdCl_2_(PPh_3_)_2_ (10 mol%), Ag_2_CO_3_ (1.5 equiv) and Na_2_CO_3_ (0.5 equiv) in TFE (1.5 mL) at 90 ^o^C for 4 h under air. Then, HOTf (2 equiv) was added after the reaction. Isolated yields

### Substrate scope with respect to amidinium salts

With the optimized reaction conditions (Table 1, entry 19) in hand, we investigated the substrate scope of the construction of amidinium salts first (Table 2). In general, boronic acids bearing both electron-donating (e.g., –Me, –*i*Pr, –OEt, –Ph) and electron-withdrawing groups (e.g., –F, –Cl, –CN) on the aromatic rings could react smoothly to produce amidinium salts in good yields (**4**–**12**, **18**–**19**).

It is noteworthy, yet very unusual in palladium chemistry, that bromo-substituted boronic acids were tolerated well under our conditions (**13** and **20**), and this provides an extremely important choice for cross-coupling reactions and makes the further structural elaboration feasible. Significantly, boronic acids bearing versatile functional groups, such as –OH, –CHO, –Ac, and –COOEt are competent reaction partners as well (**8**, **15**–**17**). Polyphenylene species, like naphthalene and 9,9-dimethyl-9*H*-fluorene were also amenable motifs (**21**–**22**). Heteroaromatic substrates, such as unprotected indole and dibenzo[b,d]thiophene were successfully converted to the corresponding products in 73–76% (**23** and **24**). Next, we investigated the effect of substituents on thioureas. Good results were obtained with di(pyrrolidin-1-yl)methanethione and 1,1,3,3-tetramethyl-thiourea (**25** and **26**). Expanding the scope to the cyclic thioureas was also effective, affording five-, six-, and seven-membered amidinium salts in 86%, 76%, and 90% yield, respectively (**27**–**29**). Notably, *ortho* substituted boronic acids also exhibited acceptable reactivity, although corresponding amidinium salts were obtained in lower yields (**30** and **31**). Importantly, unsymmetric thioureas were also good candidates in this method, featuring the positive ion delocalization in N–C–N triple atom (**32** and **33**). Considering the importance of carbazole moiety^51^ (organic light emitting diodes, OLEDs), Amoxapine^52^ (antidepressant) and Cytisine^53^ within optoelectronic materials, drugs, or natural products, we synthesized the structurally complex amidinium salts in satisfactory yield (**34**–**36**) using corresponding substrates bearing thiourea units. Moreover, estrone-derived arylboronic acid pinacol ester could also smoothly convert to amidinium salt **37** in 92% yield, indicating that our protocol enables a practical late-stage modification in medicinal chemistry.

### Substrate scope with respect to diaryl ketones

The existence of Pd^II^-monoaminocarbene complexes^20^ and the success of above reactions prompted us to expand this catalytic system to more general thioamide-containing substrates. Thioamides can be easily obtained by different functional group transformation, meanwhile, they also serve as versatile building blocks to provide valuable compounds^54^. Conventionally, Lawesson’s reagent and its analogues are employed to the synthesis of thioamides, starting from aryl amides, aryl carboxylic acids, and nitriles^55^. Willgerodt–Kindler reaction is another alternative means to access thioamides, starting from different types of starting materials such as aryl aldehydes, aryl alkyl ketones, aryl amides, and aryl acetic acids in one step^56,57^. It means that these common compounds can conveniently be used to prepare diaryl ketones in two steps with our method, which can enlarge the accessible chemical space. Although different strategies have been developed, alternative syntheses of diaryl ketones, which can commendably deal with the problem of chemoselectivity and poor functional group compatibility^58,59^, are still highly desirable and a pursued goal in the synthetic field^39,41^. Our primary studies on the reaction of thioamide with phenyl boronic acid (**2a**) also gave positive results through employing the Cu(OAc)_2_•H_2_O as additive instead of Ag_2_CO_3_ (Table 3). Using 7.5 mol% PdCl_2_(PPh_3_)_2_, thioamides smoothly reacted with aryl boronic acids to afford both symmetric and unsymmetric diaryl ketones in medium to excellent yields (Table 3, **38**–**89**). This transformation exhibited an excellent functional group tolerance. In addition to alkyl groups, halo, vinyl, ketone, ester, cyano, nitro, hydroxyl groups, complex structure (adapalene), as well as heterocycles such as indole, thiophene, pyridine and quinolone were all tolerated well. Again, bromine substituted substrates were not impeded by our catalytic protocol, thus indicating that the activation of thiourea or thioamide was prior to the oxidative addition of aryl bromide by Pd catalysis. Next, a potent antiproliferative agent^60^ **88** was readily obtained in 63% yield from the corresponding thioamide and indolyboronic acid under the standard coupling conditions. Fenofibrate, a cholesterol-modulating drug^61^, could be obtained in 90% yield under similar operation (Table 3, **89**).

**Table 3:** Substrate scope of simple and efficient synthesis of diaryl ketones.^a^

^a^Reactions were performed with thioamide (0.2 mmol, 1.0 equiv), **2** (0.4 mmol, 2 equiv), and PdCl_2_(PPh_3_)_2_ (7.5 mol%), Cu(OAc)_2_•H_2_O (2 equiv) and Na_2_CO_3_ (0.5 equiv) in TFE (1.5 mL) at 90 ^o^C for 4 h under air. Isolated yields

^b^Using PdCl_2_(PPh_3_)_2_ (10 mol%) and Ag_2_CO_3_ (1.5 equiv)

### Mechanistic investigation

**Fig. 3:**
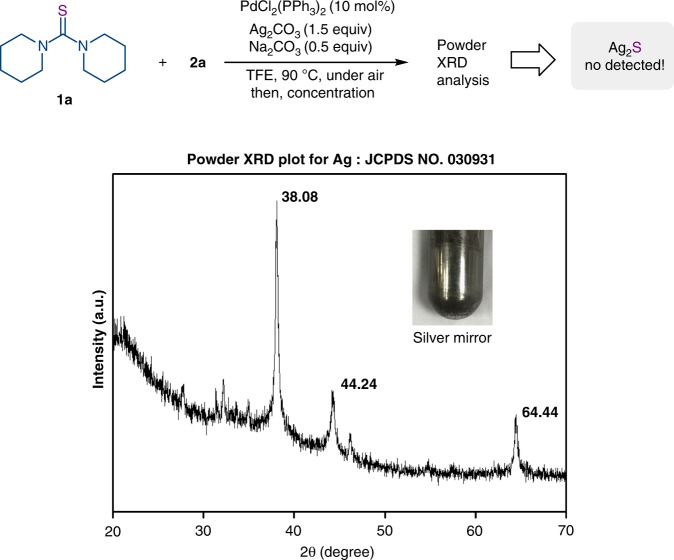
Powder XRD analysis of the reaction mixture. (inset) The photo of the silver mirror.

On the basis of the aforementioned reaction mechanism (Fig. 1c) and relevant reports, this coupling would in principle produce one molecule of Ag_2_S to finish the transmetalation with organoboron compound^25–28^, suggesting that the silver should not change in valence state. Remarkably, the black colored precipitate, collected after the reaction through direct concentration in vacuo, was characterized by the powder XRD analysis (X-ray diffraction analysis); notably, no Ag_2_S (JCPDS 14–72) was detected. As can be seen from Fig. 3, it is a clear indication for the existence of elemental silver in the powder X-ray analysis picture, which well matched the standard XRD pattern of elemental silver (JCPDS No.030931). Moreover, we also observed a silver mirror during the reaction, indicating that the Ag salt would serve as an oxidant to furnish the catalytic cycle.

**Fig. 4:**
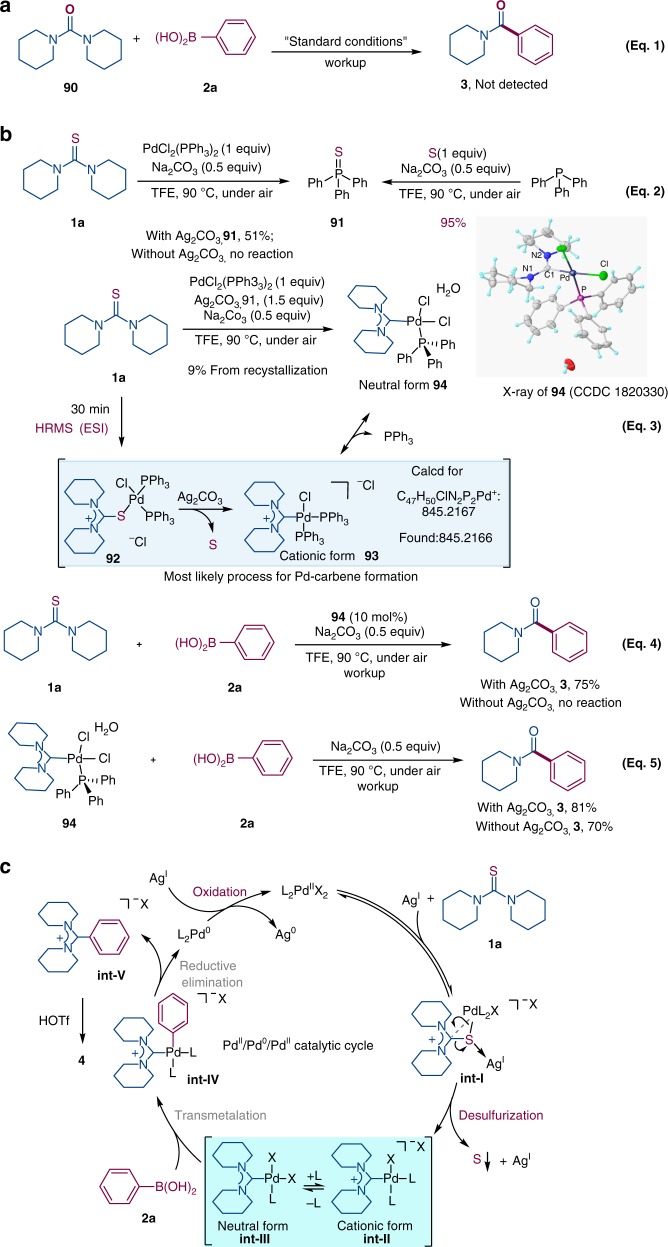
Insights into the reaction mechanism. **a** Transformation of urea **90**. **b** Mechanistic investigation. **c** The proposed mechanism.

To demonstrate the detailed reaction process, several control experiments were performed. When urea **90** was exposed to the standard conditions, no reaction occurred, suggesting the necessity of the unique structure of thiourea for the success of the transformation (Fig. 4a, eq 1). In order to identify the fate of sulfur element and to intercept the possible Pd intermediates, the thiourea **1a** was mixed with a stoichiometric amount of PdCl_2_(PPh_3_)_2_ and 1.5 equiv of Ag_2_CO_3_ at 90 °C for 4 h. Surprisingly, only in the presence of Ag_2_CO_3_, triphenylphosphine sulfide **91** was isolated in 51% yield (PhSPh was also detected under the catalytic reaction, please see the Supplementary Fig. 4). We speculated that the elemental sulfur might be generated during the reaction and then was captured by the triphenylphosphine (released from the Pd catalysis) (Fig. 4b, eq 2 left). Indeed, the PPh_3_ was readily sulfurized in TFE to triphenylphosphine sulfide **91** in 95% yield (Fig. 4b, eq 2 right).

After filtration of inorganic solids and addition of ether solvent, yellow crystal **94** was precipitated and separated, and the unique carbene structure was confirmed by X-ray crystallography (Fig. 4b). All these operations were conducted under air atmosphere. The square structure of this complex indicates it is a *cis*-configured Pd^II^ species (CCDC 1820330). The bond lengths of Pd-C1 (2.004 Å), C1-N1 (1.339 Å) and C1-N2 (1.325 Å), as well as the N–C-N angle (122.8°) are consistent with those known similar acyclic diaminocarbene-Pd complexes^20^, meanwhile, the two C–N bond lengths (1.339 Å and 1.325 Å) are typical C=N bond length, it sufficiently suggested the phenomenon of electron delocalization in N–C–N triple atom system (Fig. 4b, eq 3). Moreover, the HRMS (high resolution mass spectrometry) analysis of the mixture of the standard reaction for 30 min showed the distinctive Pd peak at m/z = 845.2166, which matched the cationic form intermediate **93** (calcd mass: 845.2167). According to the control experiment (Table 1, entry 17), the Ag salt would play dual roles in this transformation: it acts as a thiophilic auxiliary for desulfurization to form metal-aminocarbene complex, at the same time, it also serves as an oxidant to oxidize Pd^0^ to Pd^II^ to furnish the catalytic cycle. Next, complex **94** catalyzed the Suzuki−Miyaura reaction under the standard conditions in the presence of Ag_2_CO_3_ delivering benzamide **3** in high yield, suggesting the plausible intermediacy of it in the catalytic cycle (Fig. 4b, eq 4). Not surprisingly, in the absence of Ag_2_CO_3_, there was no product **3** detected. The reaction between stoichiometric amount of complex **94** and arylboronic acid **2a** was performed subsequently under the standard conditions (Fig. 4b, eq 5), rendering **3** in 81% yield (with Ag_2_CO_3_) or in 70% yield (without Ag_2_CO_3_), which further confirmed the possibility that complex **94** is the key intermediate in our transformation. Moreover, to get more insight into the reaction mechanism, the dependence of the initial rate on the concentrations of thiourea, PhB(OH)_2_ (**2a**), PdCl_2_(PPh_3_)_2_ and Ag_2_CO_3_ were examined respectively (see Supplementary Figs. 6–13). Zero-order relationships of the initial rate with the concentration of thiourea and PhB(OH)_2_ were observed. A first-order dependence of the initial rate on the amount of the palladium catalyst was established. For Ag_2_CO_3_, there was an induction phase observed in initial rate–concentration plots. After the induction phase, the reaction is clearly first order in Ag_2_CO_3_.

### Proposed mechanism

On the basis of the aforementioned results, a plausible mechanism, based on silver-facilitated Pd^II^/Pd^0^/Pd^II^ catalytic cycle (for the possibility of heterogeneous Pd catalysis, see Supplementary Fig. 5), is depicted in Fig. 4. First, the reversible intermediate **int-I** is formed via coordination or complexation^62,63^ of L_2_Pd^II^X_2_ with substrate (**1a**) in the presence of a silver salt^25^, which leads to Ag^I^-facilitated desulfurization to produce the Pd^II^-diaminocarbene intermediate along with the expulsion of sulfur and Ag^I^ salt (or their complex). We consider that sulfur-extrusion reaction is similar to the decarbonylation^64^, desulfitation and decarboxylation^65,66^. Next, The intermediate is in equilibrium between the cationic form **int-II** and the neutral form **int-III**, which involves the displacement of ligand on intermediate **int-II** by the nucleophilic X^−^ anion, yielding the neutral *cis*-form **int-III**^22^. With the presence of phenyl boronic acid (**2a)**, the Pd^II^-species **int-IV** is generated via the transmetalation. According to the related studies^21,22^ from Yates and Cavell, the C–C bond formation of Pd^II^-species **int-IV** could occur with a low activation barrier, proceeding via a concerted reductive elimination of the aryl and carbene moiety. Thus, the desired amidinium salt **int-V** is released through reductive elimination on Pd^II^ center to Pd^0^ species; once again, the Ag^I^ salt produced by desulfurization step would also serve as an oxidant to oxidize Pd^0^ to Pd^II^ to furnish the catalytic cycle with the Ag^0^ (elemental silver) as by-product, which is consistent with the experimental results (Fig. 4c).

### Further applications

**Fig. 5:**
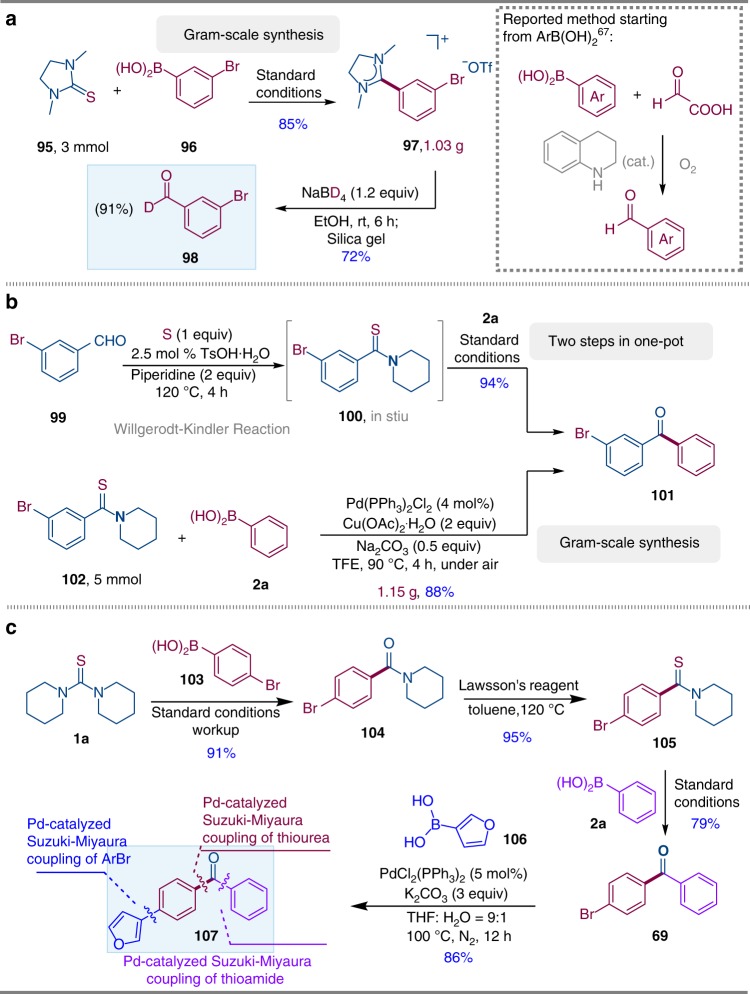
Synthetic applications. **a** Concise synthesis of deuterated aldehyde **98**. **b** One-pot synthesis of ketone **101** via Willgerodt-Kindler reaction and gram-scale synthesis of ketone **101**. **c** Orthogonal coupling for three different C–C bonds formation.

With the development of the Pd-catalyzed Suzuki–Miyaura coupling of thioureas and thioamides, we decided to pursue their applications (Fig. 5). Firstly, A gram-scale version of the reaction using cyclic thiourea **95** (3 mmol) and 3-bromophenylboronic acid **96** was performed, and the desired amidinium salt **97** was obtained in 85% yield on 1.03 g scale (Fig. 5a). Next, deuterated aromatic aldehyde **98** (91% deuterated ratio) was obtained in 72% via the reduction of amidinium salt **97** using NaBD_4_ and subsequent hydrolysis. Very recently, the synthesis of aromatic aldehydes from arylboronic acids was firstly reported by Mariano and co-workes^67^ via organocatalytic formylation reactions of boronic acids with glyoxylic acid. To the best of our knowledge, the synthesis of deuterated aromatic aldehydes from arylboronic acids, even if it takes two steps, has never been reported before, which leads to unique retrosynthetic relationship. To further demonstrate the practicability of our method, we have also developed a one-pot protocol starting directly from aldehydes. For example, 3-bromobenzaldehyde (**99**) could be converted to the corresponding thioamide via Willgerodt-Kindler reaction in one step^68^, no further purification is required, **2a**, Pd catalyst, Cu salt, Na_2_CO_3_ and TFE were added to the same reaction flask, and the corresponding cross-coupling product **101** was obtained in 94% yield in two steps after stirring the mixture at 90 ^o^C for 4 h under air (Fig. 5b). A gram-scale version of the reaction using brominated substrates **102** and **2a** was also carried out, and the desired ketone **101** was obtained in 88% yield on 1.15 g scale using only 4 mol % PdCl_2_(PPh_3_)_2_ (Fig. 5b). Moreover, a sequential Pd-catalyzed Suzuki–Miyaura couplings starting from thiourea **1a** was carried out in Fig. 5c. Thiourea **1a** was first coupled with *para*-bromo-phenylboronic acid **103**, and amide **104** was obtained in 91% yield, leaving bromo group untouched. Lawsson’s reagent readily converted amide **104** into thioamide **105**, which further underwent a Pd-catalyzed Suzuki–Miyaura coupling with phenylboronic acid **2a**, rendering ketone **69** in 79% yield, once again, bromo group on aromatic ring was remained intact. A third Suzuki–Miyaura coupling between **69** and 3-furanylboronic acid **106** was performed, furnishing a ketone **107** in 86% yield. Therefore the combination of three Suzuki–Miyaura reactions provides the free design of ketones with three different C–C bonds formation from the resource of boronic acids, while simple thiourea species donates a carbonyl bridge.

## Discussion

In summary, we have reported a Suzuki–Miyaura coupling reaction of thioureas or thioamides to afford a broad array of substituted amidinium salts or valuable diaryl ketones. Inspired by the stoichiometric carbene-generating methods, we successfully realize the direct generation of metal–carbene complex in a catalytic manner. Single crystal structure of Pd-diaminocarbene complex is obtained and proven to be the key intermediate by both catalytic and stoichiometric reactions. Meanwhile, HRMS analysis further supports our hypothetical intermediates for reaction mechanism. Preliminary mechanistic studies demonstrate the dual roles of silver salt: (i) a desulfurating reagent that assist the elimination of sulfur; (ii) an oxidant that facilitate the Pd^II^/Pd^0^/Pd^II^ catalytic cycle. Further studies on expansion of this reaction to other substrates to achieve anothercoupling reactions as well as the development of this carbene chemistry are underway in our laboratory.

## Methods

### General procedure for the synthesis of amidinium salts

Thiourea (0.2 mmol, 1.0 equiv), aryl boronic acid (0.4 mmol, 2.0 equiv), PdCl_2_(PPh_3_)_2_ (14.0 mg, 0.02 mmol, 10 mol %), Ag_2_CO_3_ (82.7 mg, 0.3 mmol, 1.5 equiv), Na_2_CO_3_ (10.6 mg, 0.1 mmol, 0.5 equiv), and TFE (1.5 mL), were placed in a 50 mL Schlenk sealed tube (with a Teflon cap) equipped with a magnetic stir bar. The reaction was stirred at 90 ^o^C for 4 h under air. Subsequently, the reaction mixture was cooled to room temperature and HOTf (60 mg, 0.4 mmol, 2 equiv) was added. The crude reaction mixture was concentrated in vacuo, and the residue was purified by flash chromatography on silica gel to provide the desired amidinium salts using CH_2_Cl_2_/MeOH as the eluent.

### General procedure for the synthesis of amide 3

Thiourea (0.2 mmol, 1.0 equiv), aryl boronic acid (0.4 mmol, 2.0 equiv), PdCl_2_(PPh_3_)_2_ (14.0 mg, 0.02 mmol, 10 mol %), Ag_2_CO_3_ (82.7 mg, 0.3 mmol, 1.5 equiv), Na_2_CO_3_ (10.6 mg, 0.1 mmol, 0.5 equiv), and TFE (1.5 mL), were placed in a 50 mL Schlenk sealed tube (with a Teflon cap) equipped with a magnetic stir bar. The reaction was stirred at 90 ^o^C for 4 h under air. Subsequently, the reaction mixture was cooled to room temperature and 1 M HCl (10 mL) was added. The mixture was then extracted with EtOAc. The organic layer was concentrated in vacuo, and the residue was purified by flash chromatography on silica gel to afford the amide **3** using petroleum ether/EtOAc as the eluent.

### General procedure for the synthesis of diaryl ketones

Thioamide (0.2 mmol, 1.0 equiv), aryl boronic acid (0.4 mmol, 2.0 equiv), PdCl_2_(PPh_3_)_2_ (10.5 mg, 0.015 mmol, 7.5 mol %), Cu(OAc)_2_·H_2_O (79.9 mg, 0.4 mmol, 2 equiv), Na_2_CO_3_ (10.6 mg, 0.1 mmol, 0.5 equiv), and TFE (1.5 mL), were placed in a 50 mL Schlenk sealed tube (with a Teflon cap) equipped with a magnetic stir bar. The reaction was stirred at 90 ^o^C for 4 h under air. Finally, the reaction mixture was cooled to room temperature and the crude reaction mixture was concentrated in vacuo. The remaining residue was purified by flash chromatography on silica gel to afford the diaryl ketones using petroleum ether/EtOAc as the eluent.

## Supplementary information

Supplementary Information

## Data Availability

Experimental procedures and characterization data are available within this article and its Supplementary Information. Data are also available from the corresponding author on request. The X-ray crystallographic coordinates for structures of **4** and **94** reported in this Article have been deposited at the Cambridge Crystallographic Data Centre (CCDC), under deposition numbers CCDC 1895265 and CCDC 1820330 respectively. These data can be obtained free of charge from The Cambridge Crystallographic Data Centre via http://www.ccdc.cam.ac.uk/data_request/cif.
