# Supplementary material for: Palladium-catalyzed Suzuki-Miyaura coupling of thioureas or thioamides
Source: Nat Commun. 2019 Dec 13;10:5709. doi: 10.1038/s41467-019-13701-5 (PMC6911099; doi:10.1038/s41467-019-13701-5)
Supplement: Supplementary file 1 — Supplementary Information [file 41467_2019_13701_MOESM1_ESM.pdf]

Supplementary Information

Palladium-Catalyzed Suzuki-Miyaura Coupling of Thioureas or Thioamides

Mai *et al*.

Supplementary Methods

General Information. Unless specified, all metal complexes, reagents, and starting materials were purchased from commercial sources and used as received. Metal salts were stored in a nitrogen atmosphere dry box. Acetonitrile, methanol, toluene, THF, Et_2O, and CH_2Cl_2 were dried by filtration through alumina according to the procedure of Grubbs. Analytical thin layer chromatography was performed on 0.25 mm extra hard silica gel plates with UV254 fluorescent indicator. ^{1}H NMR and ^{13}C NMR spectra were recorded at ambient temperature using Bruker Avance III 500MHz or 400MHz NMR spectrometer. The data are reported as follows: chemical shift in ppm from internal tetramethylsilane on the δ scale, multiplicity (br = broad, s = singlet, d = doublet, t = triplet, q = quartet, m = multiplet), coupling constants (Hz) and integrations. High-resolution mass spectra (HRMS) were recorded with a Waters Micromass GCT Premier using an Agilent 1290 with electrospray ionization (ESI) technique. X-ray diffraction (XRD) analyzer use the Smar/SmartLa.

Mechanism investigation

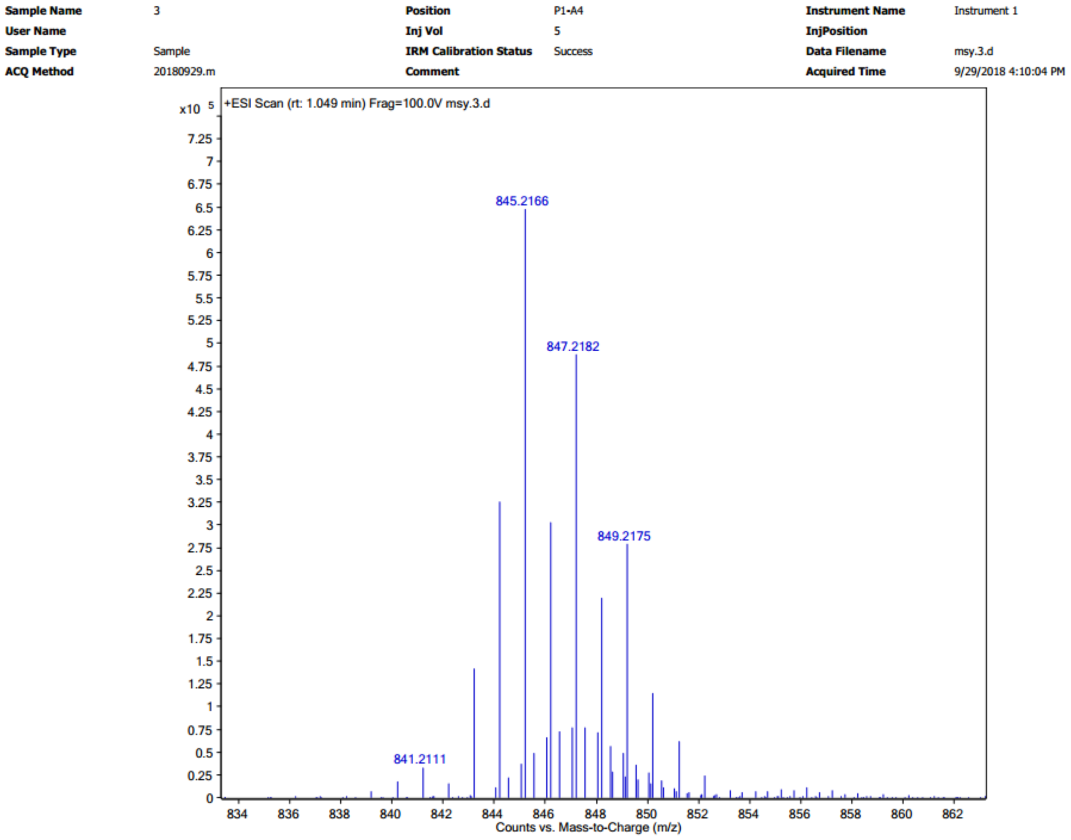

Supplementary Figure 1. HRMS(ESI) analysis of the possible Pd intermediate **93**.

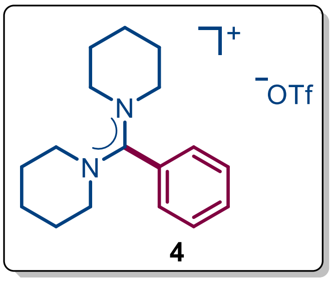

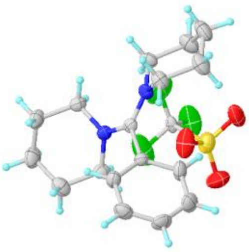

X-ray structure of **4**

CCDC 1895265

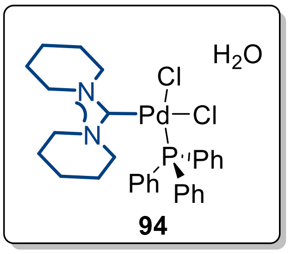

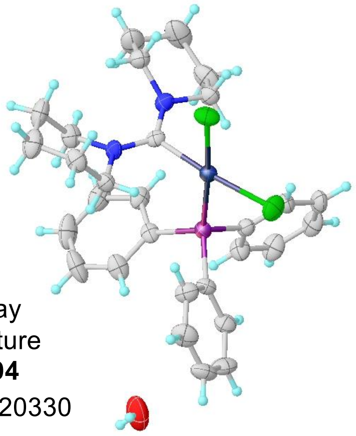

X-ray structure of **94**

CCDC 1820330

Supplementary Figure 2. X-ray structures of **4** and **94**.

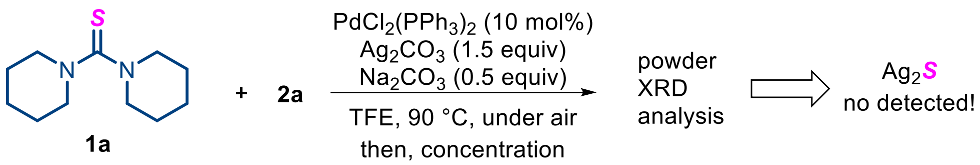

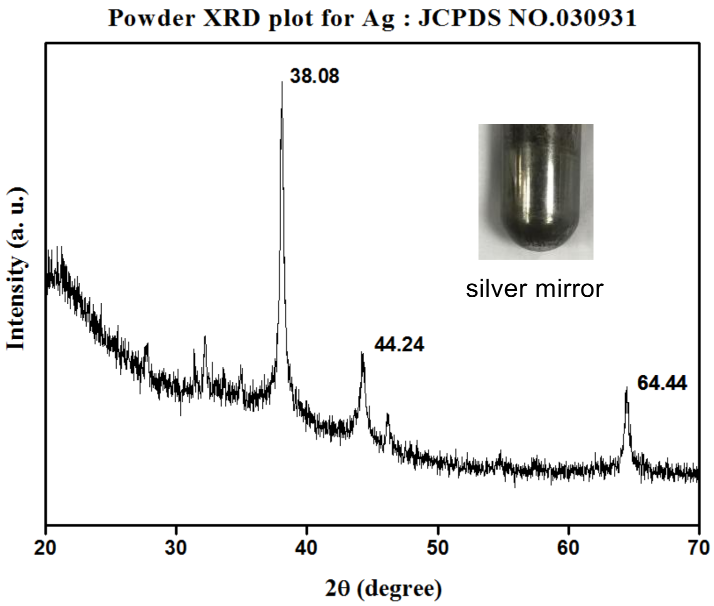

Supplementary Figure 3. Power XRD analysis of the reaction mixture.

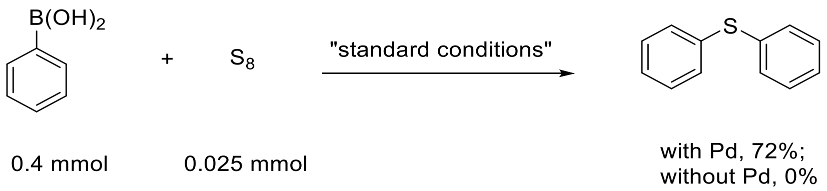

Supplementary Figure 4. Attempt to identity the fate of element sulfur. The reaction was stirred at 90 ℃ for 4 h with $PdCl_2(PPh_3)_2$ (14.0 mg, 0.020 mmol, 10 mol %) or without Pd catalyst.

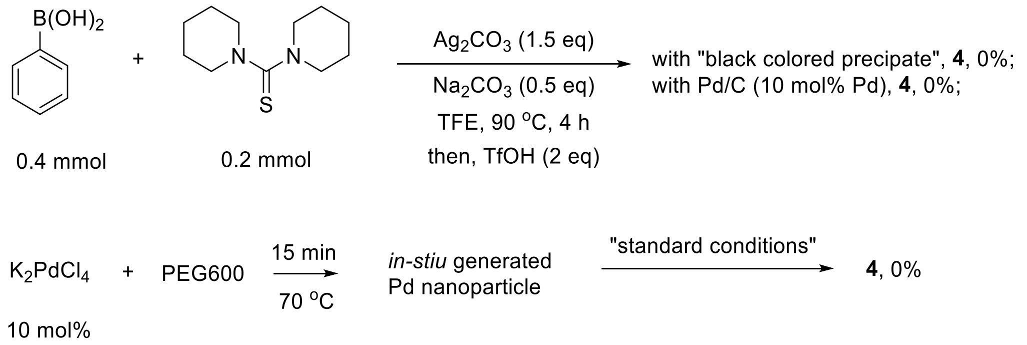

Supplementary Figure 5. Attempt to exclude the possibility of heterogeneous catalysis. General procedure for the preparation of Pd nanoparticle: A weighed amount of K_2PdCl_4 (10 mol %) and PEG 600 (100 mg) was heated at 70°C for 15 min to complete the formation of Pd nanoparticles.

Experimental procedure

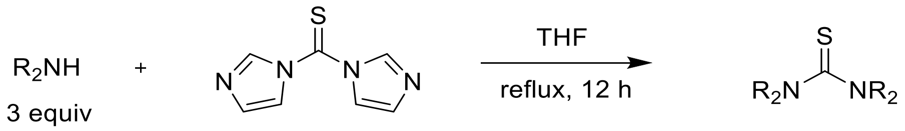

A 100 mL round bottom Schlenk flask equipped with a magnetic stirring bar was charged with 1,1'-thiocarbonyl diimidazole (10 mmol), secondary amine (30 mmol) and THF (1.5 mL). The reaction mixture was stirred at 75 °C under nitrogen atmosphere for 12 h. After the reaction was completed, the solvent was evaporated and the organic product purified by column chromatography (petroleum ether/EtOAc), giving the expected symmetric thioureas.

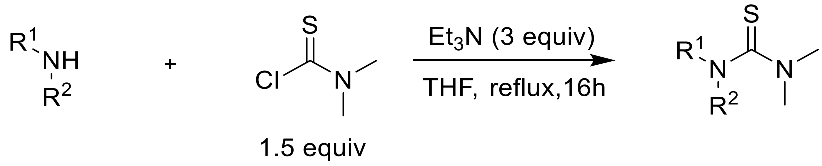

A 25 mL round bottom Schlenk flask equipped with a magnetic stirring bar was charged with secondary amine (1 mmol), dimethylcarbamothioic chloride (185.4 mg, 1.5 mmol, 1.5 euqiv), and THF (2 mL). The reaction mixture was stirred at 75 °C under nitrogen atmosphere for 16 h. After the reaction was completed, the solvent was evaporated and the organic product purified by column chromatography (petroleum ether/EtOAc), giving the expected unsymmetric thioureas.

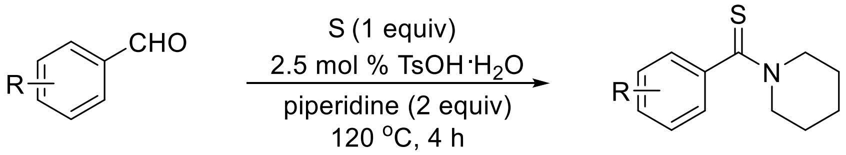

Willgerodt-Kindler Reaction

A 100 mL round bottom Schlenk flask equipped with a magnetic stirring bar was charged with aromatic aldehyde (5 mmol), S (160.3 mg, 5 mmol, 1 euqiv), *p*-toluenesulfonic acid monohydrate (23.8 mg, 0.125 mmol, 2.5 mol %) and piperidine (851.5 mg, 10 mmol, 2 equiv). The reaction mixture was stirred at 120 °C under air atmosphere for 4 h. After the reaction was completed, the solvent was evaporated and the organic product purified by column chromatography (petroleum ether/EtOAc), giving the expected substituted thioamides.

General procedure for the synthesis of amidinium salts. Thiourea (0.2 mmol, 1.0 equiv), aryl boronic acid (0.4 mmol, 2.0 equiv), $PdCl_2(PPh_3)_2$ (14.0 mg, 0.02 mmol, 10 mol %), Ag_2CO_3 (82.7 mg, 0.3 mmol, 1.5 equiv), Na_2CO_3 (10.6 mg, 0.1 mmol, 0.5 equiv), and TFE (1.5 mL), were placed in a 50 mL Schlenk sealed tube (with a Teflon cap) equipped with a magnetic stir bar. The reaction was stirred at 90 ^{o}C for 4 h under air. Subsequently, the reaction mixture was cooled to room temperature and HOTf (60 mg, 0.4 mmol, 2 equiv) was added. The crude reaction mixture was concentrated in vacuo, and the residue was purified by flash chromatography on silica gel to provide the desired amidinium salts using CH_2Cl_2/MeOH as the eluent.

General procedure for the synthesis of amides. Thiourea (0.2 mmol, 1.0 equiv), aryl boronic acid (0.4 mmol, 2.0 equiv), $PdCl_2(PPh_3)_2$ (14.0 mg, 0.02 mmol, 10 mol %), Ag_2CO_3 (82.7 mg, 0.3 mmol, 1.5 equiv), Na_2CO_3 (10.6 mg, 0.1 mmol, 0.5 equiv), and TFE (1.5 mL), were placed in a 50 mL Schlenk sealed tube (with a Teflon cap) equipped with a magnetic stir bar. The reaction was stirred at 90 ^{o}C for 4 h under air. Subsequently, the reaction mixture was cooled to room temperature and 1M HCl (10 mL) was added. The mixture was then extracted with EtOAc. The organic layer was concentrated in vacuo, and the residue was purified by flash chromatography on silica gel to afford the amide **3** using petroleum ether/EtOAc as the eluent.

General procedure for the synthesis of diaryl ketones. Thioamide (0.2 mmol, 1.0 equiv), aryl boronic acid (0.4 mmol, 2.0 equiv), $PdCl_2(PPh_3)_2$ (10.5 mg, 0.015 mmol, 7.5 mol %), $Cu(OAc)_2$•H_2O (79.9 mg, 0.4 mmol, 2 equiv), Na_2CO_3 (10.6 mg, 0.1 mmol, 0.5 equiv), and TFE (1.5 mL), were placed in a 50 mL Schlenk sealed tube (with a Teflon cap) equipped with a magnetic stir bar. The reaction was stirred at 90 ^{o}C for 4 h under air. Finally, the reaction mixture was cooled to room temperature and the crude reaction mixture was concentrated in vacuo. The remaining residue was purified by flash chromatography on silica gel to afford the diaryl ketones using petroleum ether/EtOAc as the eluent.

Kinetic studies

The dependence of the initial rate on the concentrations of thiourea (**95)**, $PhB(OH)_2$ (**2a**), $PdCl_2(PPh_3)_2$ and Ag_2CO_3 was examined. All data was collected on NMR using 1, 3, 5-trimethoxybenzene as the internal standard. The initial rates were calculated as the slopes of time zero on the conversion curves of amidinium salt **27** against time (less than 20% conversion).

General Procedure:

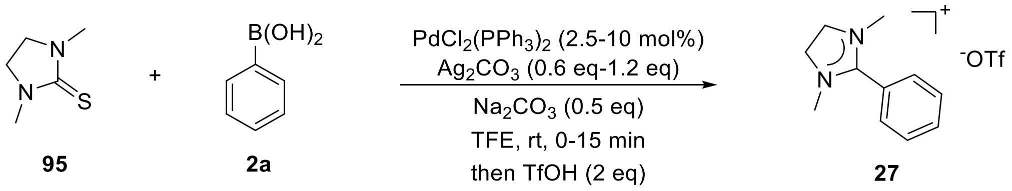

95 **2a** **27**

To a 25 mL Schlenk sealed tube (with a Teflon cap) equipped with a magnetic stirring bar was charged with thiourea **95** (0.04-0.01 mmol), **2a** (0.08-0.2 mmol), $PdCl_2(PPh_3)_2$ (2.5-10 mol %), Ag_2CO_3 (0.6-1.5 equiv), Na_2CO_3 (10.6 mg, 0.1 mmol, 0.5 equiv), and TFE (1 mL) sequentially. The mixture was stirred under rt for 1-15 min. The concentration of the product **27** obtained in each sample was determined with respect to the internal standard 1, 3, 5-trimethoxybenzene.

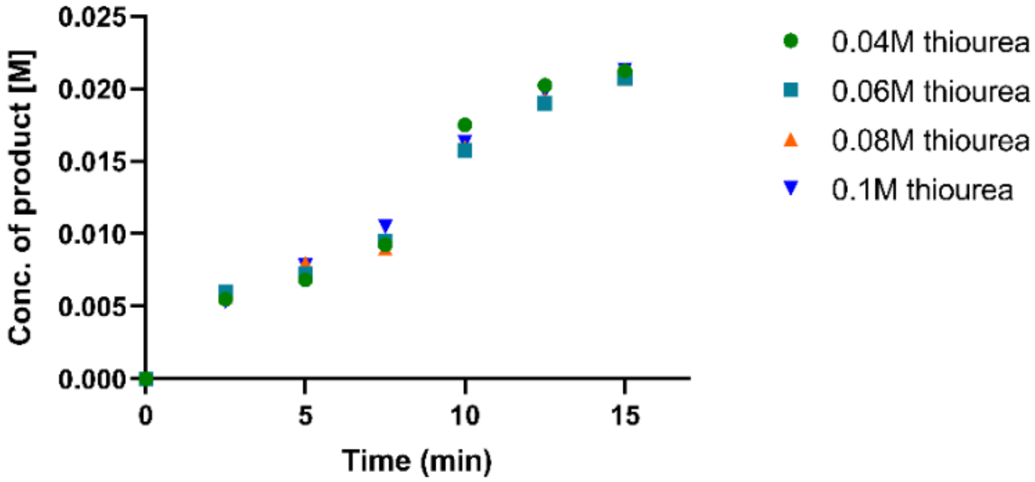

Supplementary Figure 6. Time-dependent formation of **27** at different initial concentration of thiourea **95**. s

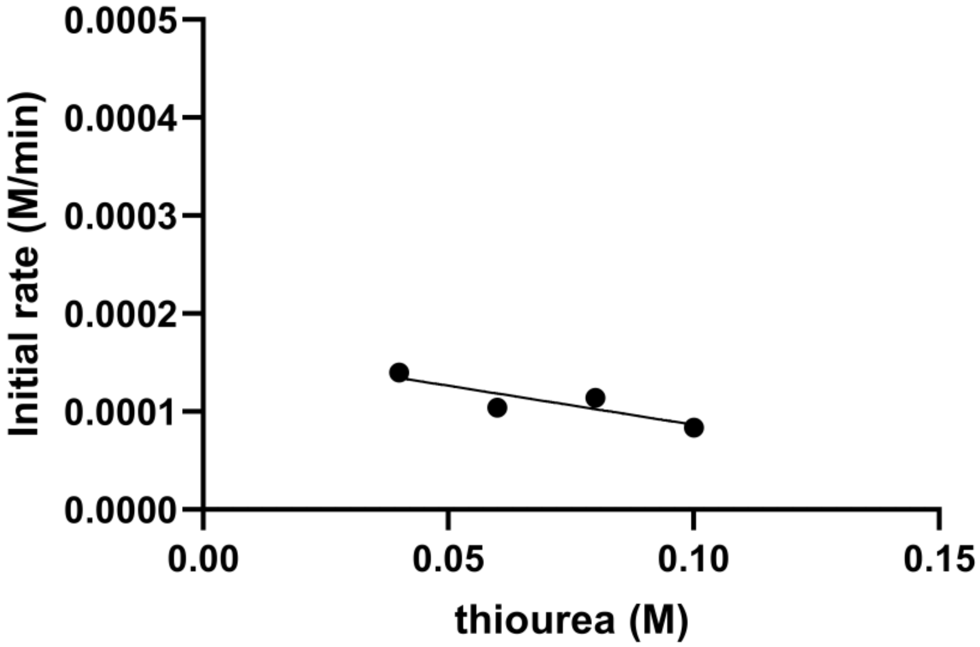

Supplementary Figure 7. Dependence of the initial rate on thioura **95**.

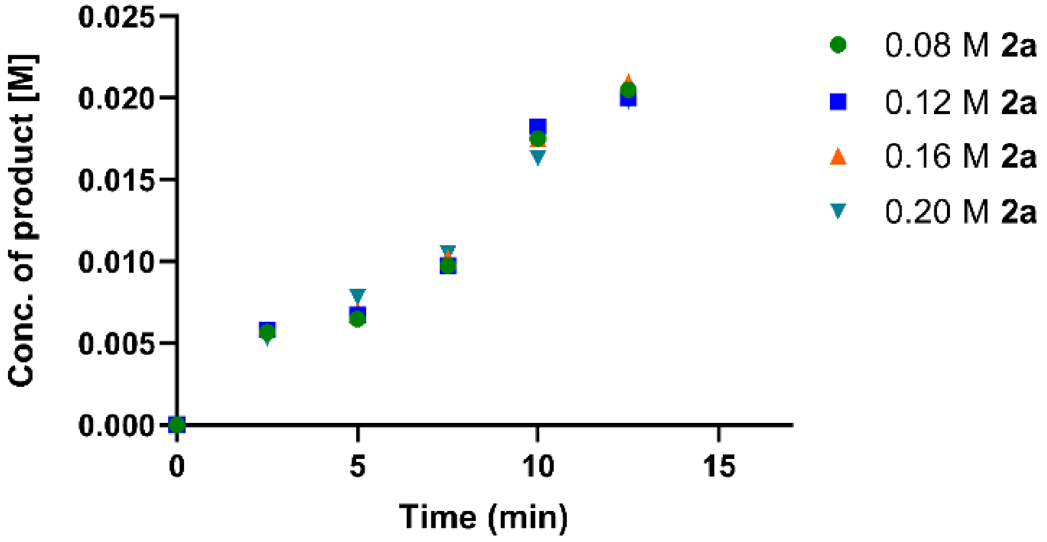

Supplementary Figure 8. Time-dependent formation of **27** at different initial concentration of PhB(OH)$_2$ **2a**.

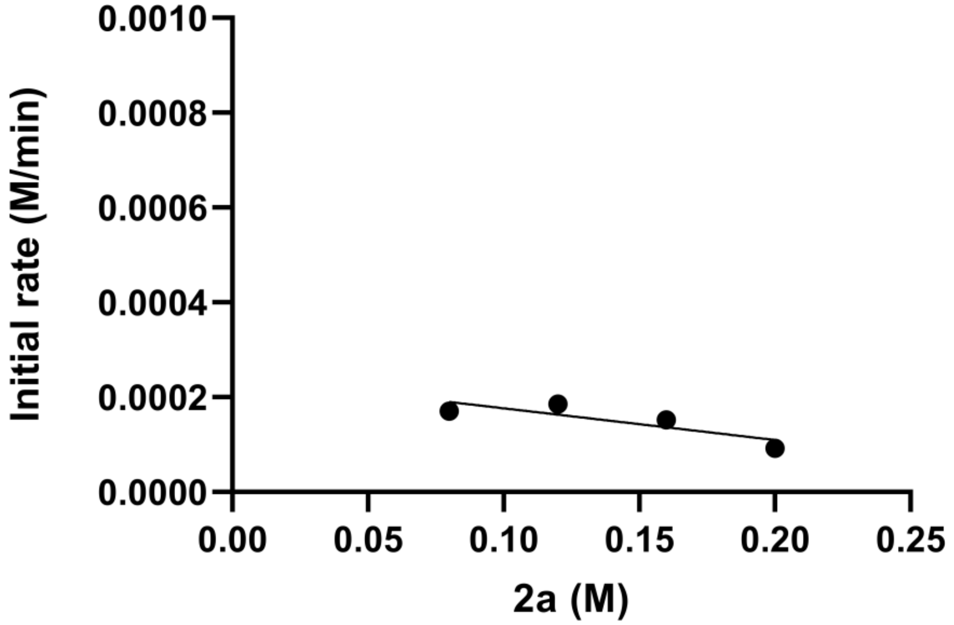

Supplementary Figure 9. Dependence of the initial rate on PhB(OH)$_2$ **2a**.

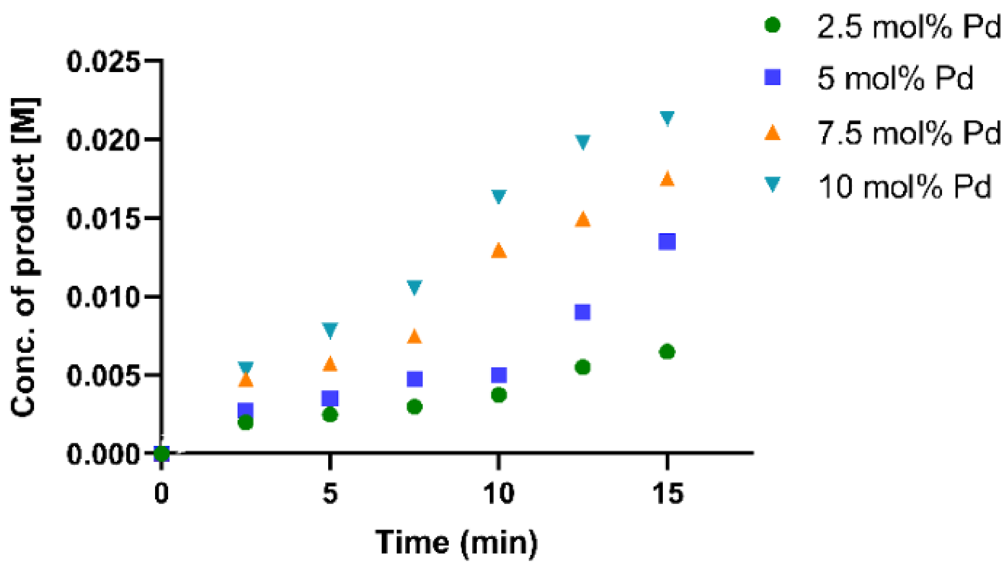

Supplementary Figure 10. Time-dependent formation of **27** at different initial concentration of $PdCl_2(PPh_3)_2$.

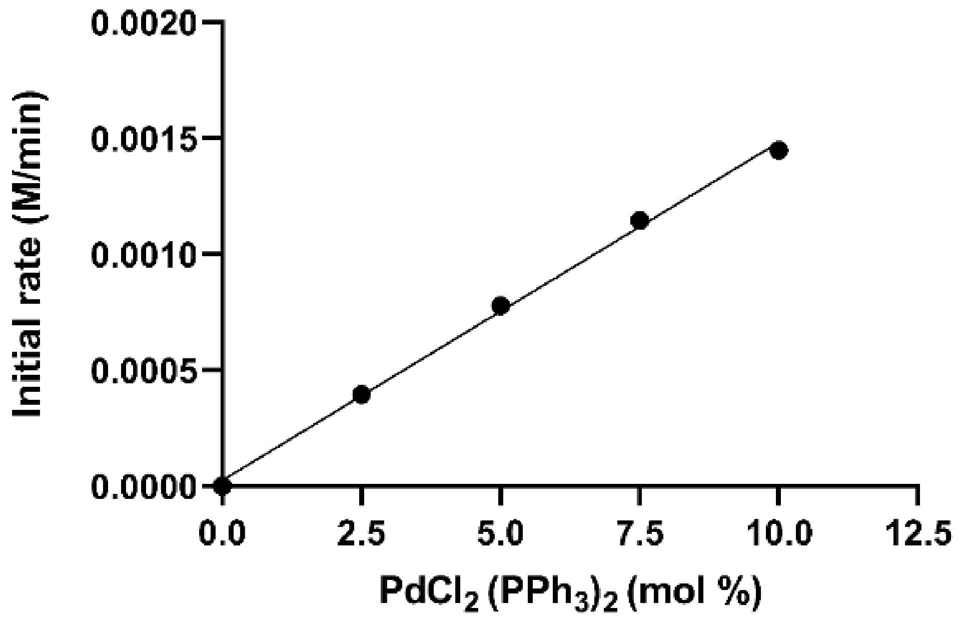

Supplementary Figure 11. Dependence of the initial rate on $PdCl_2(PPh_3)_2$.

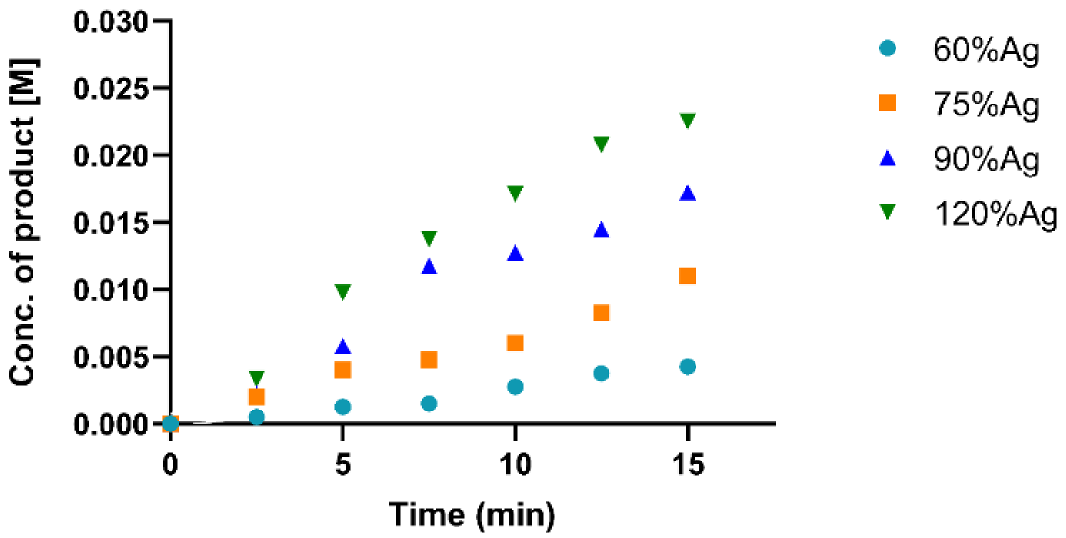

Supplementary Figure 12. Time-dependent formation of **27** at different initial concentration of Ag_2CO_3.

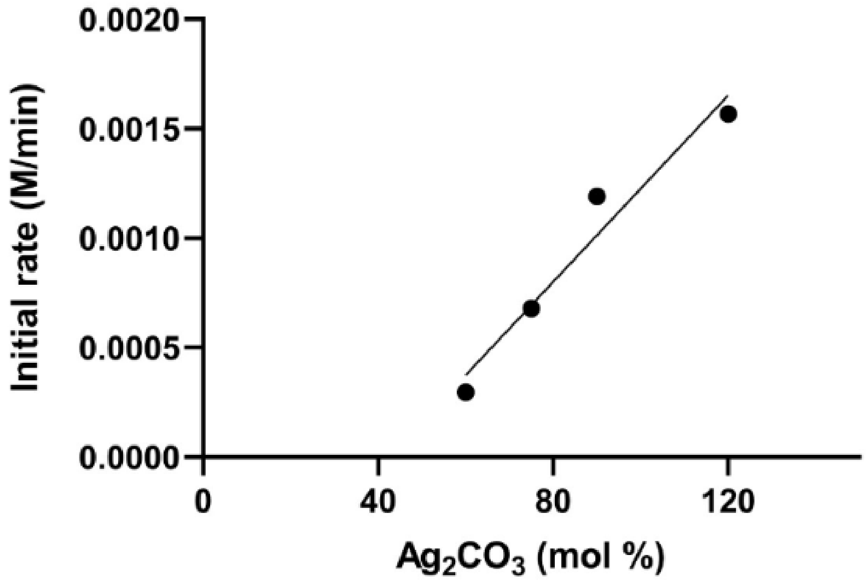

Supplementary Figure 13. Dependence of the initial rate on Ag_2CO_3.

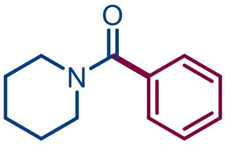

3

Phenyl(piperidin-1-yl)methanone (3) (CAS: 776-75-0), colorless oil, 93% yield. Flash column chromatography conditions: PE/ EA = 2/1 ^{1}H NMR (500 MHz, $CDCl_3$) δ 7.35 (s, 5H), 3.68 (s, 2H), 3.30 (s, 2H), 1.56 (d, *J* = 82.2 Hz, 6H). ^{13}C NMR (126 MHz, $CDCl_3$) δ 170.3, 136.5, 129.4, 128.4, 126.8, 48.8, 43.1, 26.6, 25.6, 24.6.

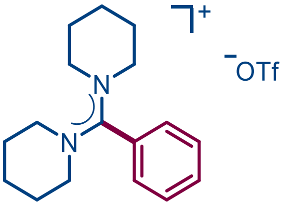

4

1,1'-(Phenylmethylene)dipiperidine, trifluoromethanesulfonate salt (4), white solid, 98% yield. Flash column chromatography conditions: DCM/ MeOH = 20/1. ^{1}H NMR (500 MHz, $CDCl_3$) δ 7.64 (td, *J* = 8.5, 4.2 Hz, 1H), 7.57 (d, *J* = 4.4 Hz, 4H), 3.50 (s, 8H), 1.73 (s, 12H). ^{19}F NMR (471 MHz, $CDCl_3$) δ -78.2. ^{13}C NMR (126 MHz, $CDCl_3$) δ 168.4, 133.3, 129.8, 129.7, 129.3, 52.8, 26.4, 22.9. HRMS (ESI) m/z calcd. for $C_{17}H_{25}N_2^+$ $[M]^+$: 257.2012, found 257.2011.

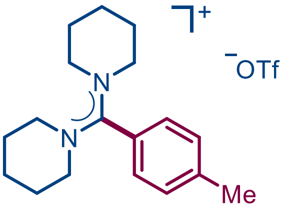

5

1,1'-(*p*-Tolylmethylene)dipiperidine, trifluoromethanesulfonate salt (5), colorless oil, 96% yield. Flash column chromatography conditions: DCM/ MeOH = 20/1. ^{1}H NMR (500 MHz, $CDCl_3$) δ 7.44 (d, *J* = 8.1 Hz, 2H), 7.36 (d, *J* = 7.9 Hz, 2H), 3.50 (s, 8H), 2.43 (s, 3H), 1.73 (s, 12H). ^{19}F NMR (471 MHz, $CDCl_3$) δ -78.2. ^{13}C NMR (126 MHz, $CDCl_3$) δ 168.6, 144.5, 130.5, 129.4, 126.5, 52.8, 26.5, 22.9, 21.6. HRMS (ESI) m/z calcd. for $C_{18}H_{27}N_2^+$ $[M]^+$: 271.2169, found 271.2169.

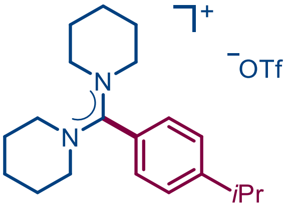

6

1,1'-((4-Isopropylphenyl)methylene)dipiperidine, trifluoromethanesulfonate salt (6), colorless oil, 94% yield. Flash column chromatography conditions: DCM/ MeOH = 20/1. ^{1}H NMR (500 MHz, $CDCl_3$) δ 7.46 (d, *J* = 8.3 Hz, 2H), 7.39 (d, *J* = 8.2 Hz, 2H), 3.49 (s, 8H), 3.03 – 2.91 (m, 1H), 1.72 (s, 12H), 1.26 (d, *J* = 6.9 Hz, 6H). ^{19}F NMR (471 MHz, $CDCl_3$) δ -78.2. ^{13}C NMR (126 MHz, $CDCl_3$) δ 168.6, 155.1, 129.5, 127.9, 126.7, 120.8 (q, *J* = 320.9 Hz), 52.8, 34.2, 26.4, 23.5, 22.9. HRMS (ESI) m/z calcd. for $C_{20}H_{31}N_2^+$ $[M]^+$: 299.2482, found 299.2486.

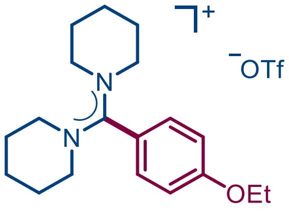

7

1,1'-((4-Ethoxyphenyl)methylene)dipiperidine, trifluoromethanesulfonate salt (7), colorless oil, 86% yield. Flash column chromatography conditions: DCM/ MeOH = 20/1. ^{1}H NMR (500 MHz, $CDCl_3$) δ 7.48 (d, *J* = 8.8 Hz, 2H), 7.02 (d, *J* = 8.8 Hz, 2H), 4.09 (q, *J* = 7.0 Hz, 2H), 3.49 (m, 8H), 1.73 (s, 12H), 1.43 (t, *J* = 7.0 Hz, 3H). ^{19}F NMR (471 MHz, $CDCl_3$) δ -78.2. ^{13}C NMR (126 MHz, $CDCl_3$) δ 168.6, 163.2, 131.6, 120.8 (q, *J* = 320.9 Hz), 120.6, 115.6, 64.1, 52.8, 26.4, 22.9, 14.5. HRMS (ESI) m/z calcd. for $C_{19}H_{29}ON_2^+$ $[M]^+$: 301.2274, found 301.2279.

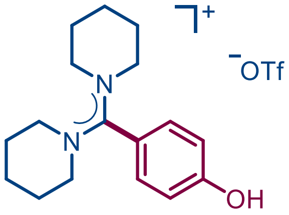

8

4-(Di(piperidin-1-yl)methyl)phenol, trifluoromethanesulfonate salt (8), colorless oil, 75% yield. Flash column chromatography conditions: DCM/ MeOH = 20/1. ^{1}H NMR (400 MHz, $CDCl_3$) δ 9.17 (s, 1H), 7.35 (s, 2H), 7.04 (s, 2H), 3.50 (d, *J* = 58.9 Hz, 8H), 1.74 (s, 12H). ^{19}F NMR (471 MHz, $CDCl_3$) δ -78.3. ^{13}C NMR (126 MHz, $CDCl_3$) δ 169.4, 162.9, 131.7, 118.8, 117.2, 52.8, 26.5, 23.0. HRMS (ESI) m/z calcd. for $C_{17}H_{25}ON_2^+$ $[M]^+$: 273.1961, found 273.1963.

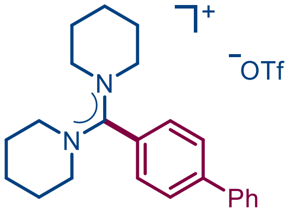

9

1,1'-([1,1'-Biphenyl]-4-ylmethylene)dipiperidine, trifluoromethanesulfonate salt (9), colorless oil, 90% yield. Flash column chromatography conditions: DCM/ MeOH = 20/1. ^{1}H NMR (500 MHz, $CDCl_3$) δ 7.78 (d, *J* = 8.4 Hz, 2H), 7.69 – 7.64 (m, 2H), 7.63 – 7.59 (m, 2H), 7.53 – 7.46 (m, 2H), 7.45 – 7.38 (m, 1H), 3.56 (s, 8H), 1.75 (s, 12H). ^{19}F NMR (471 MHz, $CDCl_3$) δ -78.2. ^{13}C NMR (126 MHz, $CDCl_3$) δ 168.4, 146.3, 138.9, 130.0, 129.1, 128.7, 128.4, 128.2, 127.2, 52.9, 26.5, 22.9. HRMS (ESI) m/z calcd. for $C_{23}H_{29}N_2^+$ $[M]^+$: 333.2325, found 333.2329.

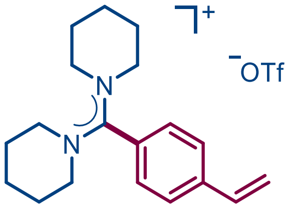

10

1,1'-((4-Vinylphenyl)methylene)dipiperidine, trifluoromethanesulfonate salt (10), colorless oil, 88% yield. Flash column chromatography conditions: DCM/ MeOH = 20/1. ^{1}H NMR (500 MHz, $CDCl_3$) δ 7.56 (d, *J* = 8.3 Hz, 2H), 7.52 (d, *J* = 8.2 Hz, 2H), 6.73 (dd, *J* = 17.6, 10.9 Hz, 1H), 5.89 (d, *J* = 17.6 Hz, 1H), 5.43 (d, *J* = 10.9 Hz, 1H), 3.52 (s, 8H), 1.71 (s, 12H). ^{19}F NMR (471 MHz, $CDCl_3$) δ -78.2. ^{13}C NMR (126 MHz, $CDCl_3$) δ 168.2, 142.6, 135.2, 129.9, 128.5, 127.4, 117.9, 52.9, 26.5, 22.9. HRMS (ESI) m/z calcd. for $C_{19}H_{27}N_2^+$ $[M]^+$: 283.2169, found 283.2173.

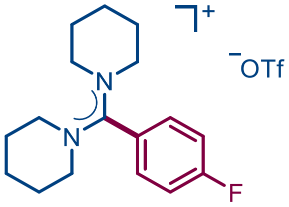

11

1,1'-((4-Fluorophenyl)methylene)dipiperidine, trifluoromethanesulfonate salt (11), colorless oil, 94% yield. Flash column chromatography conditions: DCM/ MeOH = 20/1. ^{1}H NMR (500 MHz, $CDCl_3$) δ 7.71 – 7.61 (m, 2H), 7.27 (m, 2H), 3.48 (s, 8H), 1.74 (s, 12H). ^{19}F NMR (471 MHz, $CDCl_3$) δ -78.2, -103.5. ^{13}C NMR (126 MHz, $CDCl_3$) δ 167.5, 165.5 (d, *J* = 256.6 Hz), 132.2 (d, *J* = 9.2 Hz), 125.6 (d, *J* = 3.4 Hz), 120.8 (q, *J* = 320.7 Hz), 117.3 (d, *J* = 22.3 Hz), 52.9, 26.4, 22.9. HRMS (ESI) m/z calcd. for $C_{17}H_{24}FN_2^+$ $[M]^+$: 275.1918, found 275.1914.

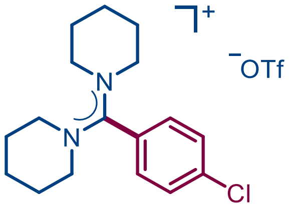

12

1,1'-((4-Chlorophenyl)methylene)dipiperidine, trifluoromethanesulfonate salt (12), colorless oil, 91% yield. Flash column chromatography conditions: DCM/ MeOH = 20/1. ^{1}H

NMR (500 MHz, $CDCl_3$) δ 7.56 (dd, *J* = 20.5, 8.3 Hz, 4H), 3.48 (s, 8H), 1.72 (s, 12H). ^{19}F NMR (471 MHz, $CDCl_3$) δ -78.2. ^{13}C NMR (126 MHz, $CDCl_3$) δ 167.3, 139.9, 131.0, 130.2, 128.0, 120.8 (q, *J* = 320.7 Hz), 52.9, 26.3, 22.8. HRMS (ESI) m/z calcd. for $C_{17}H_{24}ClN_2^+$ $[M]^+$: 291.1623, found 291.1626.

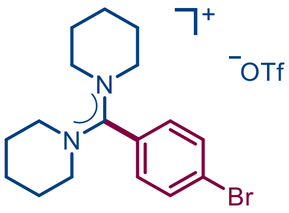

13

1,1'-((4-Bromophenyl)methylene)dipiperidine, trifluoromethanesulfonate salt (13), white solid, 98% yield. Flash column chromatography conditions: DCM/ MeOH = 20/1. ^{1}H NMR (500 MHz, $CDCl_3$) δ 7.70 (d, *J* = 8.5 Hz, 2H), 7.50 (d, *J* = 8.5 Hz, 2H), 3.47 (s, 8H), 1.70 (s, 12H). ^{19}F NMR (471 MHz, $CDCl_3$) δ -78.2. ^{13}C NMR (126 MHz, $CDCl_3$) δ 167.3, 133.1, 131.0, 128.4, 128.3, 120.7 (q, *J* = 320.9 Hz), 52.8, 26.3, 22.8. HRMS (ESI) m/z calcd. for $C_{17}H_{24}BrN_2^+$ $[M]^+$: 335.1117, found 335.1119.

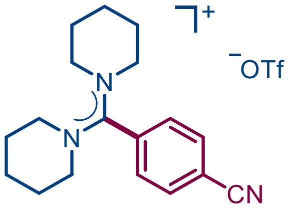

14

4-(Di(piperidin-1-yl)methyl)benzonitrile, trifluoromethanesulfonate salt (14), colorless oil, 90% yield. Flash column chromatography conditions: DCM/ MeOH = 20/1. ^{1}H NMR (500 MHz, $CDCl_3$) δ 7.86 (td, *J* = 8.5, 6.5 Hz, 4H), 3.47 (s, 8H), 1.73 (s, 12H). ^{19}F NMR (471 MHz, $CDCl_3$) δ -78.2. ^{13}C NMR (126 MHz, $CDCl_3$) δ 166.3, 134.0, 133.4, 130.4, 120.7 (q, *J* = 320.7 Hz), 117.2, 116.8, 53.0, 26.2, 22.7. HRMS (ESI) m/z calcd. for $C_{18}H_{24}N_3^+$ $[M]^+$: 282.1965, found 282.1969.

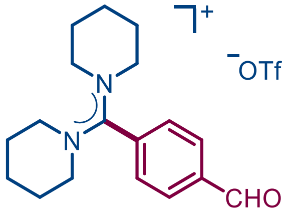

15

4-(Di(piperidin-1-yl)methyl)benzaldehyde, trifluoromethanesulfonate salt (15), colorless oil, 77% yield. Flash column chromatography conditions: DCM/ MeOH = 20/1. ^{1}H NMR (500 MHz, $CDCl_3$) δ 10.11 (s, 1H), 8.09 (d, *J* = 8.3 Hz, 2H), 7.85 (d, *J* = 8.2 Hz, 2H), 3.51 (s, 8H), 1.74 (s, 12H). ^{19}F NMR (471 MHz, $CDCl_3$) δ -78.2. ^{13}C NMR (126 MHz, $CDCl_3$) δ

190.9, 167.0, 139.3, 135.1, 130.7, 130.3, 120.8 (q, J = 320.6 Hz), 53.0, 26.4, 22.8. HRMS (ESI) m/z calcd. for $C_{18}H_{25}ON_2^+$ [M]$^+$: 285.1961, found 285.1961.

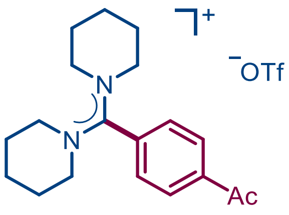

16

1-(4-(Di(piperidin-1-yl)methyl)phenyl)ethan-1-one, trifluoromethanesulfonate salt (16), colorless oil, 86% yield. Flash column chromatography conditions: DCM/ MeOH = 20/1. ^{1}H NMR (500 MHz, $CDCl_3$) δ 8.11 (d, J = 8.3 Hz, 2H), 7.74 (d, J = 8.3 Hz, 2H), 3.47 (s, 8H), 2.64 (s, 3H), 1.71 (s, 12H). ^{19}F NMR (471 MHz, $CDCl_3$) δ -78.2. ^{13}C NMR (126 MHz, $CDCl_3$) δ 196.9, 167.1, 140.4, 133.7, 129.8, 129.4, 120.7 (q, J = 320.8 Hz), 52.9, 26.8, 26.3, 22.7. HRMS (ESI) m/z calcd. for $C_{19}H_{27}ON_2^+$ [M]$^+$: 299.2118, found 299.2121.

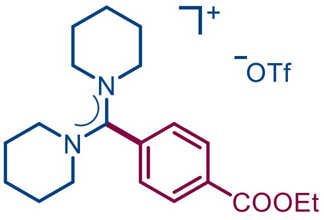

17

Ethyl 4-(di(piperidin-1-yl)methyl)benzoate, trifluoromethanesulfonate salt (17), colorless oil, 95% yield. Flash column chromatography conditions: DCM/ MeOH = 20/1. ^{1}H NMR (500 MHz, $CDCl_3$) δ 8.22 (d, J = 8.4 Hz, 2H), 7.71 (d, J = 8.4 Hz, 2H), 4.42 (q, J = 7.1 Hz, 2H), 3.52 (s, 8H), 1.74 (s, 12H), 1.41 (t, J = 7.1 Hz, 3H). ^{19}F NMR (471 MHz, $CDCl_3$) δ -78.2. ^{13}C NMR (126 MHz, $CDCl_3$) δ 167.3, 165.0, 134.8, 133.7, 130.8, 129.5, 61.8, 53.0, 26.4, 22.8, 14.2. HRMS (ESI) m/z calcd. for $C_{20}H_{29}O_2N_2^+$ [M]$^+$: 329.2224, found 329.2225.

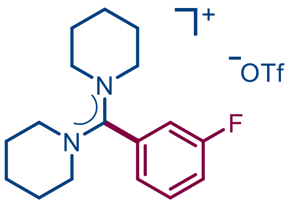

18

1,1'-((3-Fluorophenyl)methylene)dipiperidine, trifluoromethanesulfonate salt (18), colorless oil, 82% yield. Flash column chromatography conditions: DCM/ MeOH = 20/1. ^{1}H NMR (500 MHz, $CDCl_3$) δ 7.58 (tdd, J = 7.3, 5.6, 1.6 Hz, 1H), 7.42 (d, J = 7.7 Hz, 1H), 7.38 – 7.29 (m, 2H), 3.48 (s, 8H), 1.72 (s, 12H). ^{19}F NMR (471 MHz, $CDCl_3$) δ -78.2, -108.7. ^{13}C NMR (126 MHz, $CDCl_3$) δ 166.6, 162.8 (d, J = 251.6 Hz), 131.9 (d, J = 8.2 Hz), 131.6 (d, J = 7.5 Hz), 125.3 (d, J = 3.3 Hz), 120.8 (q, J = 320.8 Hz), 120.4 (d, J = 21.0 Hz), 116.4 (d, J =

23.3 Hz), 52.9, 26.3, 22.8. HRMS (ESI) m/z calcd. for $C_{17}H_{24}FN_2^+$ $[M]^+$: 275.1918, found 275.1919.

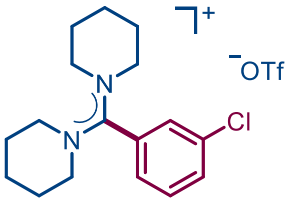

19

1,1'-((3-Chlorophenyl)methylene)dipiperidine, trifluoromethanesulfonate salt (19), colorless oil, 84% yield. Flash column chromatography conditions: DCM/ MeOH = 20/1. ^1H NMR (500 MHz, $CDCl_3$) δ 7.61 (dt, *J* = 7.4, 1.8 Hz, 1H), 7.59 – 7.51 (m, 3H), 3.50 (s, 8H), 1.72 (s, 12H). ^{19}F NMR (471 MHz, $CDCl_3$) δ -78.2. ^{13}C NMR (126 MHz, $CDCl_3$) δ 166.7, 135.8, 133.4, 131.4, 131.3, 128.9, 127.8, 120.8 (q, *J* = 320.7 Hz), 52.9, 26.3, 22.8. HRMS (ESI) m/z calcd. for $C_{17}H_{24}ClN_2^+$ $[M]^+$: 291.1623, found 291.1623.

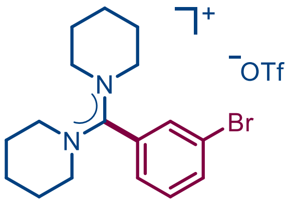

20

1,1'-((3-Bromophenyl)methylene)dipiperidine, trifluoromethanesulfonate salt (20), white solid, 92% yield. Flash column chromatography conditions: DCM/ MeOH = 20/1. ^1H NMR (500 MHz, $CDCl_3$) δ 7.77 (dd, *J* = 8.1, 0.6 Hz, 1H), 7.65 (dd, *J* = 10.8, 4.7 Hz, 2H), 7.48 (t, *J* = 7.9 Hz, 1H), 3.51 (s, 8H), 1.73 (s, 12H). ^{19}F NMR (471 MHz, $CDCl_3$) δ -78.2. ^{13}C NMR (126 MHz, $CDCl_3$) δ 166.7, 136.4, 131.7, 131.7, 131.6, 128.4, 123.7, 120.8 (q, *J* = 320.9 Hz), 53.0, 26.4, 22.8. HRMS (ESI) m/z calcd. for $C_{17}H_{24}BrN_2^+$ $[M]^+$: 335.1117, found 335.1115.

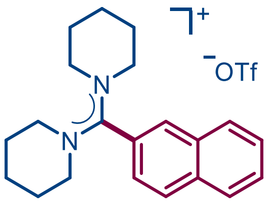

21

1,1'-(Naphthalen-2-ylmethylene)dipiperidine, trifluoromethanesulfonate salt (21), colorless oil, 88% yield. Flash column chromatography conditions: DCM/ MeOH = 20/1. ^1H NMR (500 MHz, $CDCl_3$) δ 8.18 (s, 1H), 8.03 (dd, *J* = 8.0, 4.9 Hz, 2H), 7.93 (d, *J* = 8.1 Hz, 1H), 7.71 – 7.60 (m, 2H), 7.56 (dd, *J* = 8.5, 1.7 Hz, 1H), 3.59 (s, 8H), 1.76 (s, 12H). ^{19}F NMR (471 MHz, $CDCl_3$) δ -78.2. ^{13}C NMR (126 MHz, $CDCl_3$) δ 168.5, 135.1, 132.6, 130.7, 130.0, 129.2, 129.2, 128.0, 127.9, 126.7, 124.5, 120.8 (q, *J* = 320.6 Hz), 53.0, 26.5, 22.9. HRMS (ESI) m/z calcd. for $C_{21}H_{27}N_2^+$ $[M]^+$: 307.2169, found 307.2168.

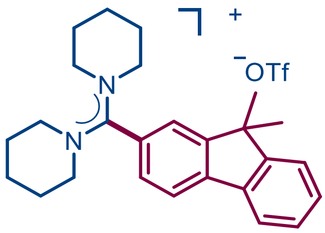

22

1,1'-((9,9-Dimethyl-9H-fluoren-2-yl)methylene)dipiperidine, trifluoromethanesulfonate salt (22), colorless oil, 77% yield. Flash column chromatography conditions: DCM/ MeOH = 20/1. ^{1}H NMR (500 MHz, $CDCl_3$) δ 7.88 (d, *J* = 7.8 Hz, 1H), 7.81 – 7.75 (m, 1H), 7.64 (d, *J* = 1.0 Hz, 1H), 7.53 (dd, *J* = 7.8, 1.5 Hz, 1H), 7.50 – 7.45 (m, 1H), 7.44 – 7.35 (m, 2H), 3.55 (s, 8H), 1.76 (s, 12H), 1.53 (s, 6H). ^{19}F NMR (471 MHz, $CDCl_3$) δ -78.1. ^{13}C NMR (126 MHz, $CDCl_3$) δ 169.1, 155.2, 154.4, 144.9, 137.1, 129.3, 128.8, 127.6, 127.5, 124.1, 123.0, 121.1, 121.1, 120.9 (q, *J* = 320.7 Hz), 52.9, 47.3, 26.8, 26.6, 23.0. HRMS (ESI) m/z calcd. for $C_{26}H_{33}N_2^+$ $[M]^+$: 373.2638, found 373.2639.

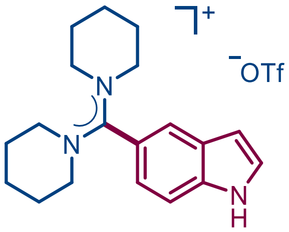

23

5-(Di(piperidin-1-yl)methyl)-1*H*-indole, trifluoromethanesulfonate salt (23), colorless oil, 76% yield. Flash column chromatography conditions: DCM/ MeOH = 20/1. ^{1}H NMR (500 MHz, $CDCl_3$) δ 10.52 (s, 1H), 7.69 (s, 1H), 7.62 (d, *J* = 8.4 Hz, 1H), 7.37 (t, *J* = 2.8 Hz, 1H), 6.98 (dd, *J* = 8.4, 1.6 Hz, 1H), 6.54 (s, 1H), 3.44 (br, 8H), 1.71 (s, 12H). ^{19}F NMR (471 MHz, $CDCl_3$) δ -78.2. ^{13}C NMR (126 MHz, $CDCl_3$) δ 170.8, 139.0, 128.1, 127.9, 122.6, 121.5, 118.9, 113.5, 102.6, 52.8, 26.5, 23.0. HRMS (ESI) m/z calcd. for $C_{19}H_{28}N_3^+$ $[M]^+$: 296.2121, found 296.2120.

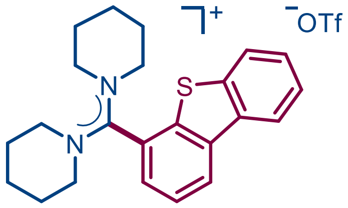

24

1,1'-(Dibenzo[*b*,*d*]thiophen-4-ylmethylene)dipiperidine, trifluoromethanesulfonate salt (24), colorless oil, 73% yield. Flash column chromatography conditions: DCM/ MeOH = 20/1. ^{1}H NMR (500 MHz, $CDCl_3$) δ 8.37 (d, *J* = 7.9 Hz, 1H), 8.27 – 8.18 (m, 1H), 7.96 (d, *J* = 7.4 Hz, 1H), 7.93 – 7.86 (m, 1H), 7.70 (t, *J* = 7.7 Hz, 1H), 7.60 – 7.54 (m, 2H), 3.71 (s, 8H), 1.74

(s, 12H). ^{19}F NMR (471 MHz, $CDCl_3$) δ -78.2. ^{13}C NMR (126 MHz, $CDCl_3$) δ 165.4, 138.2, 138.0, 137.3, 134.6, 128.9, 128.2, 126.1, 125.6, 125.4, 124.8, 123.0, 122.2, 52.9, 25.9, 23.0. HRMS (ESI) m/z calcd. for $C_{23}H_{27}SN_2^+$ $[M]^+$: 363.1889, found 363.1888.

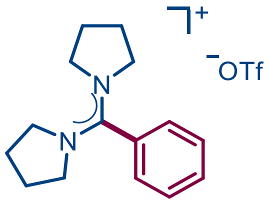

25

1,1'-(Phenylmethylene)dipyrrolidine, trifluoromethanesulfonate salt (25), white solid, 82% yield. Flash column chromatography conditions: DCM/ MeOH = 20/1. ^{1}H NMR (500 MHz, $CDCl_3$) δ 7.52 (m, 5H), 4.18 (t, J = 6.4 Hz, 4H), 3.20 (t, J = 6.6 Hz, 4H), 2.12 – 1.99 (m, 4H), 1.93 – 1.72 (m, 4H). ^{19}F NMR (471 MHz, $CDCl_3$) δ -78.3. ^{13}C NMR (126 MHz, $CDCl_3$) δ 162.2, 132.3, 131.0, 130.0, 126.2, 120.9 (q, J = 320.7 Hz), 54.6, 51.5, 26.1, 24.0. HRMS (ESI) m/z calcd. for $C_{15}H_{21}N_2^+$ $[M]^+$: 229.1699, found 229.1700.

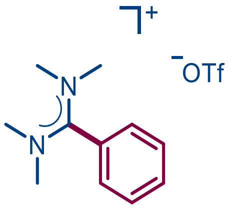

26

***N,N,N',N'*-Tetramethyl-1-phenylmethanediamine, trifluoromethanesulfonate salt (26)**, colorless oil, 88% yield. Flash column chromatography conditions: DCM/ MeOH = 20/1. ^{1}H NMR (500 MHz, $CDCl_3$) δ 7.64 – 7.60 (m, 1H), 7.59 – 7.53 (m, 4H), 3.21 (m, 12H). ^{19}F NMR (471 MHz, $CDCl_3$) δ -78.3. ^{13}C NMR (126 MHz, $CDCl_3$) δ 169.8, 169.8, 133.2, 130.0, 129.5, 129.0, 120.8 (q, J = 320.5 Hz), 43.8. HRMS (ESI) m/z calcd. for $C_{11}H_{17}N_2^+$ $[M]^+$: 177.1386, found 177.1387.

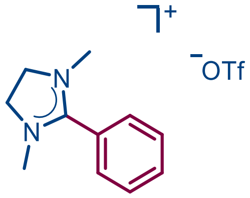

27

1,3-Dimethyl-2-phenylimidazolidine, trifluoromethanesulfonate salt (27), colorless oil, 86% yield. Flash column chromatography conditions: DCM/ MeOH = 20/1. ^{1}H NMR (500 MHz, $CDCl_3$) δ 7.69 – 7.52 (m, 5H), 4.15 (s, 4H), 2.96 (s, 6H). ^{19}F NMR (471 MHz, $CDCl_3$) δ -78.3. ^{13}C NMR (126 MHz, $CDCl_3$) δ 166.4, 132.8, 129.8, 128.5, 121.8, 120.9 (d, J = 320.5 Hz), 50.4, 34.7. HRMS (ESI) m/z calcd. for $C_{11}H_{15}N_2^+$ $[M]^+$: 175.1230, found 175.1229.

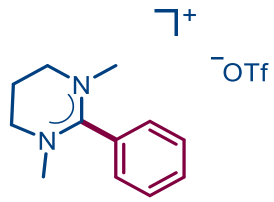

28

1,3-Dimethyl-2-phenylhexahydropyrimidine, trifluoromethanesulfonate salt (28), colorless oil, 76% yield. Flash column chromatography conditions: DCM/ MeOH = 20/1. ^{1}H NMR (500 MHz, $CDCl_3$) δ 7.57 (dd, *J* = 5.9, 2.6 Hz, 3H), 7.55 – 7.50 (m, 2H), 3.79 – 3.65 (m, 4H), 2.90 (s, 6H), 2.38 – 2.21 (m, 2H). ^{19}F NMR (471 MHz, $CDCl_3$) δ -78.2. ^{13}C NMR (126 MHz, $CDCl_3$) δ 162.6, 131.6, 130.0, 128.4, 127.2, 120.9 (q, *J* = 320.5 Hz), 47.8, 42.2, 19.0. HRMS (ESI) m/z calcd. for $C_{12}H_{17}N_2^+$ $[M]^+$:189.1386, found 189.1389.

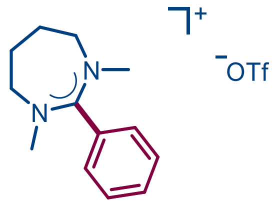

29

1,3-Dimethyl-2-phenyl-1,3-diazepane, trifluoromethanesulfonate salt (29), colorless oil, 90% yield. Flash column chromatography conditions: DCM/ MeOH = 20/1. ^{1}H NMR (500 MHz, $CDCl_3$) δ 7.64 – 7.58 (m, 1H), 7.58 – 7.50 (m, 4H), 3.91 (s, 4H), 2.90 (s, 6H), 2.12 (s, 4H). ^{19}F NMR (471 MHz, $CDCl_3$) δ -78.2. ^{13}C NMR (126 MHz, $CDCl_3$) δ 168.2, 133.2, 130.1, 129.3, 128.9, 120.9 (q, *J* = 320.8 Hz), 53.7, 42.8, 21.9. HRMS (ESI) m/z calcd. for $C_{13}H_{19}N_2^+$ $[M]^+$:203.1543, found 203.1540.

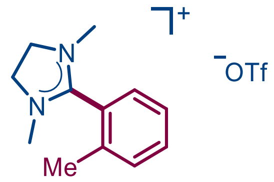

30

1,3-Dimethyl-2-(o-tolyl)imidazolidine, trifluoromethanesulfonate salt (30), colorless oil, 44% yield. Flash column chromatography conditions: DCM/ MeOH = 20/1. ^{1}H NMR (400 MHz, $CDCl_3$) δ 7.67 – 7.34 (m, 4H), 4.24 (d, *J* = 54.8 Hz, 4H), 2.92 (s, 3H), 2.29 (s, 3H). ^{19}F NMR (376 MHz, $CDCl_3$) δ -78.2. ^{13}C NMR (101 MHz, $CDCl_3$) δ 166.5, 136.1, 132.7, 131.2, 128.6, 127.5, 121.3, 50.4, 34.2, 18.8. HRMS (ESI) m/z calcd. for $C_{12}H_{17}N_2^+$ $[M]^+$: 189.1386, found 189.1392.

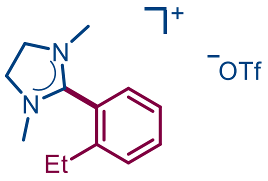

31

2-(2-Ehylphenyl)-1,3-dimethylimidazolidine, trifluoromethanesulfonate salt (31), colorless oil, 32% yield. Flash column chromatography conditions: DCM/ MeOH = 20/1. ^{1}H NMR (400 MHz, $CDCl_3$) δ 7.51 (m, 4H), 4.24 (d, *J* = 75.3 Hz, 4H), 2.91 (s, 6H), 2.53 (q, *J* = 6.8 Hz, 2H), 1.23 (t, *J* = 5.7 Hz, 3H). ^{19}F NMR (376 MHz, $CDCl_3$) δ -78.3. ^{13}C NMR (101 MHz, $CDCl_3$) δ 166.6, 142.0, 132.9, 129.4, 128.6, 127.6, 120.8, 50.4, 34.3, 25.7, 14.8. HRMS (ESI) m/z calcd. for $C_{13}H_{19}N_2^+$ $[M]^+$: 203.1543, found 203.1544.

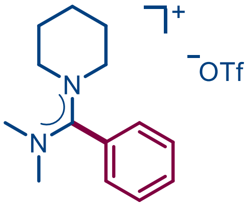

32

***N,N*-Dimethyl-1-phenyl-1-(piperidin-1-yl)methanamine, trifluoromethanesulfonate salt (32)**, colorless oil, 66% yield. Flash column chromatography conditions: DCM/ MeOH = 20/1. ^{1}H NMR (500 MHz, $CDCl_3$) δ 7.64 (m, 1H), 7.57 (d, *J* = 4.4 Hz, 4H), 3.21 (m, 10H), 1.73 (s, 6H). ^{19}F NMR (471 MHz, $CDCl_3$) δ -78.2. ^{13}C NMR (126 MHz, $CDCl_3$) δ 169.1, 130.0, 129.5, 129.4, 120.9 (q, *J* = 320.4 Hz), 52.6, 44.1, 26.4, 23.0. HRMS (ESI) m/z calcd. for $C_{14}H_{21}N_2^+$ $[M]^+$: 217.1699, found 217.1700.

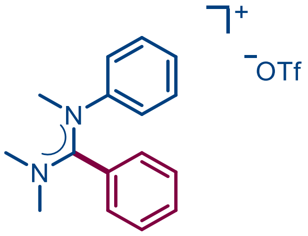

33

***N,N,N'*-Trimethyl-*N'*,1-diphenylmethanediamine, trifluoromethanesulfonate salt (33)**, colorless oil, 93% yield. Flash column chromatography conditions: DCM/ MeOH = 20/1. ^{1}H NMR (500 MHz, $CDCl_3$) δ 7.73 (d, *J* = 6.9 Hz, 2H), 7.64 – 7.56 (m, 3H), 7.51 – 7.40 (m, 4H), 7.34 (t, *J* = 6.9 Hz, 1H), 3.41 (s, 3H), 3.02 (s, 3H), 2.94 (s, 3H). ^{19}F NMR (471 MHz, $CDCl_3$) δ -78.2. ^{13}C NMR (126 MHz, $CDCl_3$) δ 168.3, 143.3, 133.2, 130.4, 130.1, 129.6, 129.4, 128.6, 125.2, 120.9 (q, *J* = 320.5 Hz), 45.6, 44.8, 44.0. HRMS (ESI) m/z calcd. for $C_{16}H_{19}N_2^+$ $[M]^+$: 239.1543, found 239.1543.

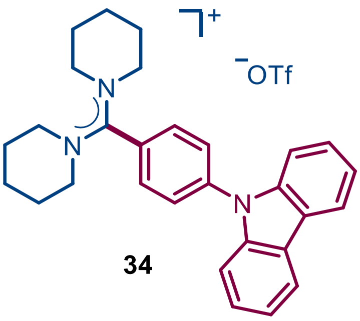

34

9-(4-(Di(piperidin-1-yl)methyl)phenyl)-9*H*-carbazole, trifluoromethanesulfonate salt (34), white solid, 91% yield. Flash column chromatography conditions: DCM/ MeOH = 20/1. ^{1}H NMR (500 MHz, $CDCl_3$) δ 8.13 (d, *J* = 7.7 Hz, 1H), 7.85 (dd, *J* = 26.0, 8.4 Hz, 2H), 7.52 (d, *J* = 8.2 Hz, 1H), 7.44 (t, *J* = 7.4 Hz, 1H), 7.32 (t, *J* = 7.4 Hz, 1H), 3.59 (s, 4H), 1.76 (s, 6H). ^{19}F NMR (471 MHz, $CDCl_3$) δ -78.1. ^{13}C NMR (126 MHz, $CDCl_3$) δ 167.6, 142.6, 139.7, 121.0, 120.8 (d, *J* = 320.8 Hz), 120.5, 109.6, 53.0, 26.4, 22.9. HRMS (ESI) m/z calcd. for $C_{29}H_{32}N_3^+$ $[M]^+$: 422.2591, found 422.2589.

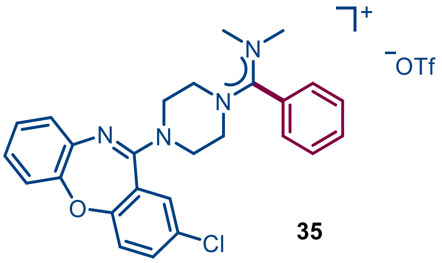

35

1-(4-(2-Chlorodibenzo[*b,f*][1,4]oxazepin-11-yl)piperazin-1-yl)-*N*,*N*-dimethyl-1-phenylmethanamine, trifluoromethanesulfonate salt (35), white solid, 89% yield. Flash column chromatography conditions: DCM/ MeOH = 20/1. ^{1}H NMR (500 MHz, $CDCl_3$) δ 7.62 (t, *J* = 6.8 Hz, 3H), 7.59 – 7.53 (m, 2H), 7.38 (dd, *J* = 8.6, 2.5 Hz, 1H), 7.30 (d, *J* = 2.5 Hz, 1H), 7.16 (d, *J* = 8.6 Hz, 1H), 7.08 (t, *J* = 6.3 Hz, 3H), 7.04 – 6.97 (m, 1H), 3.68 (s, 8H), 3.47 (s, 3H), 2.98 (s, 3H). ^{19}F NMR (471 MHz, $CDCl_3$) δ -78.1. ^{13}C NMR (126 MHz, $CDCl_3$) δ 169.3, 159.3, 158.2, 151.6, 139.4, 133.4, 133.1, 130.7, 130.0, 130.0, 129.0, 128.7, 127.1, 125.9, 125.3, 124.2, 122.8, 120.8 (q, *J* = 320.5 Hz), 120.2, 50.7, 47.6, 44.2. HRMS (ESI) m/z calcd. for $C_{26}H_{26}ClON_4^+$ $[M]^+$: 445.1790, found 445.1789.

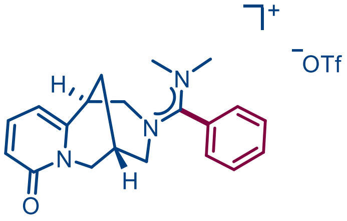

36

(5*R*)-3-((*R*)-(Dimethylamino)(phenyl)methyl)-1,2,3,4,5,6-hexahydro-8*H*-1,5-methanopyrido[1,2-*a*][1,5]diazocin-8-one, trifluoromethanesulfonate salt (36), white solid, 72% yield. Flash column chromatography conditions: DCM/ MeOH = 20/1. ^{1}H NMR (400 MHz, Acetone) δ 7.75 – 7.40 (m, 4H), 7.25 (m, 1H), 6.74 (m, 1H), 6.24 (m, 2H), 4.46 (d, *J* = 15.9

Hz, 1H), 4.15 (s, 2H), 4.00 (dd, J = 15.8, 6.2 Hz, 1H), 3.85 (d, J = 13.7 Hz, 1H), 3.50 (s, 3H), 3.29 (d, J = 8.6 Hz, 4H), 2.90 (s, 2H), 2.23 (d, J = 13.0 Hz, 1H), 2.07 (d, J = 17.9 Hz, 1H). ^{19}F NMR (471 MHz, Acetone) δ -79.0. ^{13}C NMR (101 MHz, Acetone) δ 170.9, 164.0, 149.5, 141.3, 133.8, 130.6, 130.2, 129.7, 126.8, 123.6, 120.4, 118.3, 117.3, 108.5, 57.5, 49.6, 44.8, 34.8, 28.9, 24.8. HRMS (ESI) m/z calcd. for $C_{20}H_{24}ON_3^+$ $[M]^+$: 322.1914, found 322.1914.

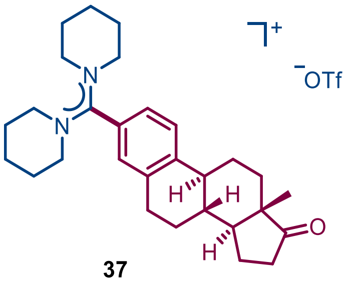

37

(8R,9S,13S,14S)-3-(Di(piperidin-1-yl)methyl)-13-methyl-6,7,8,9,11,12,13,14,15,16-decahydro-17H-cyclopenta[a]phenanthren-17-one, trifluoromethanesulfonate salt (37), white solid, 92% yield. Flash column chromatography conditions: DCM/ MeOH = 20/1. ^{1}H NMR (500 MHz, $CDCl_3$) δ 7.43 (m, 1H), 7.25 (m, 2H), 3.45 (s, 8H), 3.11 – 2.84 (m, 2H), 2.53 – 2.24 (m, 3H), 2.19 – 1.86 (m, 6H), 1.79 – 1.39 (m, 16H), 0.89 (s, 3H). ^{19}F NMR (471 MHz, $CDCl_3$) δ -78.2. ^{13}C NMR (126 MHz, $CDCl_3$) δ 220.4, 168.6, 146.0, 138.6, 129.7, 126.7, 126.6, 126.4, 120.7 (q, 321.1 Hz), 52.7, 50.3, 47.7, 44.4, 37.4, 35.7, 31.3, 29.0, 26.4, 25.9, 25.3, 22.8, 21.4, 13.7. HRMS (ESI) m/z calcd. for $C_{29}H_{41}ON_2^+$ $[M]^+$: 433.3213, found 433.3218.

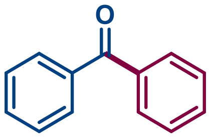

38

Benzophenone (38) (CAS: 119-61-9), white solid, 91% yield. Flash column chromatography conditions: PE/ EA = 20/1. ^{1}H NMR (500 MHz, $CDCl_3$) δ 7.81 (dt, J = 8.4, 1.5 Hz, 2H), 7.62 – 7.56 (m, 1H), 7.52 – 7.45 (m, 2H).^{13}C NMR (126 MHz, $CDCl_3$) δ 196.8, 137.6, 132.5, 130.1, 128.3.

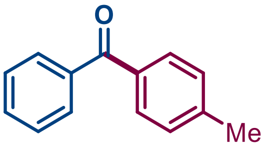

39

Phenyl(*p*-tolyl)methanone (39) (CAS: 134-84-9), white solid, 82% yield. Flash column chromatography conditions: PE/ EA = 20/1. ^{1}H NMR (500 MHz, CDCl3) δ 7.82 – 7.77 (m, 2H), 7.75 – 7.71 (m, 2H), 7.61 – 7.55 (m, 1H), 7.50 – 7.45 (m, 2H), 7.29 (d, J = 7.9 Hz, 2H), 2.45 (s, 3H). ^{13}C NMR (126 MHz, CDCl3) δ 196.5, 143.3, 138.0, 134.9, 132.2, 130.3, 130.0, 129.0, 128.2, 21.7.

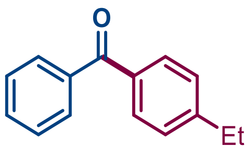

40

(4-Ethylphenyl)(phenyl)methanone (40) (CAS: 18220-90-1), white solid, 83% yield. Flash column chromatography conditions: PE/ EA = 20/1. ^{1}H NMR (500 MHz, $CDCl_3$) δ 7.82 – 7.78 (m, 2H), 7.77 – 7.73 (m, 2H), 7.60 – 7.55 (m, 1H), 7.51 – 7.45 (m, 2H), 7.31 (d, J = 8.4 Hz, 2H), 2.74 (q, J = 7.6 Hz, 2H), 1.29 (t, J = 7.6 Hz, 3H). ^{13}C NMR (126 MHz, $CDCl_3$) δ 196.5, 149.4, 138.0, 135.1, 132.2, 130.4, 130.0, 128.2, 127.8, 29.0, 15.3.

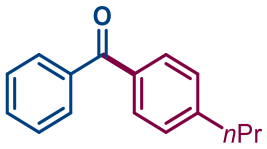

41

Phenyl(4-propylphenyl)methanone (41) (CAS: 64357-39-7), white solid, 70% yield. Flash column chromatography conditions: PE/ EA = 20/1. ^{1}H NMR (500 MHz, $CDCl_3$) δ 7.83 – 7.78 (m, 2H), 7.76 – 7.73 (m, 2H), 7.61 – 7.55 (m, 1H), 7.51 – 7.45 (m, 2H), 7.29 (d, J = 8.3 Hz, 2H), 2.67 (t, J = 7.4 Hz, 2H), 1.72 – 1.66 (m, 2H), 0.97 (t, J = 7.4 Hz, 3H).^{13}C NMR (126 MHz, $CDCl_3$) δ 196.6, 148.0, 138.0, 135.1, 132.2, 130.3, 130.0, 128.4 (d, J = 8.5 Hz), 128.2, 38.1, 24.3, 13.8.

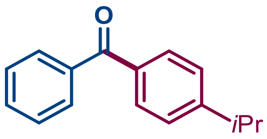

42

(4-Isopropylphenyl)(phenyl)methanone (42) (CAS: 18864-76-1), white solid, 85% yield. Flash column chromatography conditions: PE/ EA = 20/1. ^{1}H NMR (500 MHz, $CDCl_3$) δ 7.83 – 7.78 (m, 2H), 7.78 – 7.74 (m, 2H), 7.62 – 7.56 (m, 1H), 7.50 – 7.46 (m, 2H), 7.33 (d, J = 8.1 Hz, 2H), 3.00 (dt, J = 13.8, 6.9 Hz, 1H), 1.30 (d, J = 6.9 Hz, 6H). ^{13}C NMR (126 MHz, $CDCl_3$) δ 196.5, 154.0, 138.0, 135.2, 132.2, 130.5, 130.0, 128.2, 126.4, 34.3, 23.8.

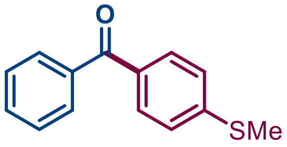

43

(4-(Methylthio)phenyl)(phenyl)methanone (43) (CAS: 23405-48-3), white solid, 50% yield. Flash column chromatography conditions: PE/ EA = 20/1. ^{1}H NMR (500 MHz, $CDCl_3$) δ 7.76 (t, J = 8.5 Hz, 4H), 7.60 – 7.55 (m, 1H), 7.48 (t, J = 7.7 Hz, 2H), 7.29 (d, J = 8.3 Hz, 2H),

2.54 (s, 3H).^{13}C NMR (126 MHz, $CDCl_3$) δ 195.9, 145.3, 137.9, 133.6, 132.2, 130.7, 129.9, 128.3, 124.8, 14.9.

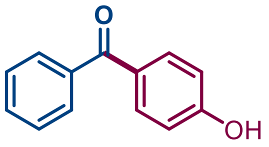

44

(4-Hydroxyphenyl)(phenyl)methanone (44) (CAS: 1137-42-4), white solid, 80% yield. Flash column chromatography conditions: PE/ EA = 20/1. ^{1}H NMR (500 MHz, $CDCl_3$) δ 7.95 – 7.69 (m, 4H), 7.62 – 7.54 (m, 1H), 7.51 – 7.41 (m, 2H), 6.94 (d, *J* = 8.8 Hz, 2H). ^{13}C NMR (126 MHz, $CDCl_3$) δ 196.7, 160.7, 138.0, 133.1, 132.2, 129.8, 129.5, 128.2, 115.3.

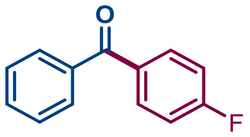

45

(4-Fluorophenyl)(phenyl)methanone (45) (CAS: 345-83-5), white solid, 78% yield. Flash column chromatography conditions: PE/ EA = 20/1. ^{1}H NMR (500 MHz, $CDCl_3$) δ 7.93 – 7.82 (m, 2H), 7.81 – 7.74 (m, 2H), 7.62 – 7.56 (m, 1H), 7.54 – 7.46 (m, 2H), 7.20 – 7.12 (m, 2H).^{13}C NMR (126 MHz, $CDCl_3$) δ 195.3, 165.4 (d, *J* = 252.0 Hz) 137.5, 133.8, 132.8,132.4, 129.9, 128.4, 115.5 (d, *J* = 21.9 Hz).

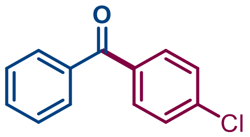

46

(4-Chlorophenyl)(phenyl)methanone (46) (CAS: 134-85-0), white solid, 82% yield. Flash column chromatography conditions: PE/ EA = 20/1. ^{1}H NMR (500 MHz, $CDCl_3$) δ 7.82 – 7.72 (m, 4H), 7.62 – 7.58 (m, 1H), 7.53 – 7.43 (m, 4H).^{13}C NMR (126 MHz, $CDCl_3$) δ 195.5, 138.9, 137.2, 135.9, 132.7, 131.5, 130.0, 128.7, 128.4.

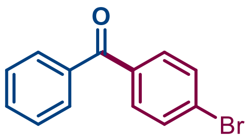

47

(4-Bromophenyl)(phenyl)methanone (47) (CAS: 90-90-4), white solid, 75% yield. Flash column chromatography conditions: PE/ EA = 20/1. ^{1}H NMR (500 MHz, $CDCl_3$) δ 7.77 (dd, *J* = 8.2, 1.2 Hz, 2H), 7.70 – 7.66 (m, 2H), 7.65 – 7.57 (m, 3H), 7.49 (dd, *J* = 10.7, 4.7 Hz, 2H). ^{13}C NMR (126 MHz, $CDCl_3$) δ 195.6, 137.1, 136.3, 132.7, 131.6, 131.5, 129.9, 128.4, 127.5.

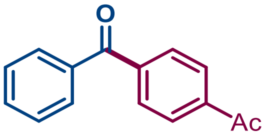

48

1-(4-benzoylphenyl)ethan-1-one (48) (CAS: 53689-84-2), white solid, 83% yield. Flash column chromatography conditions: PE/ EA = 20/1. ^{1}H NMR (500 MHz, $CDCl_3$) δ 8.08 – 8.03 (m, 2H), 7.89 – 7.84 (m, 2H), 7.84 – 7.77 (m, 2H), 7.64 – 7.59 (m, 1H), 7.49 (dd, *J* = 10.8, 4.7 Hz, 2H), 2.67 (s, 3H). ^{13}C NMR (126 MHz, $CDCl_3$) δ 197.6, 196.0, 141.3, 139.5, 136.9, 133.0, 130.1， 130.0, 128.5, 128.2, 26.9.

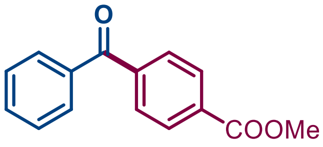

49

Methyl 4-benzoylbenzoate (49) (CAS: 6158-54-9), white solid, 89% yield. Flash column chromatography conditions: PE/ EA = 20/1. ^{1}H NMR (500 MHz, $CDCl_3$) δ 8.18 – 8.12 (m, 2H), 7.88 – 7.82 (m, 2H), 7.81 – 7.76 (m, 2H), 7.65 – 7.58 (m, 1H), 7.53 – 7.45 (m, 2H), 3.96 (s, 3H).^{13}C NMR (126 MHz, $CDCl_3$) δ 196.1, 166.3, 141.3, 136.9, 133.2, 133.0, 130.1, 129.8, 129.5, 128.5, 52.5.

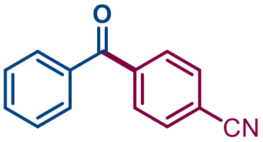

50

4-Benzoylbenzonitrile (50) (CAS: 1503-49-7), white solid, 92% yield. Flash column chromatography conditions: PE/ EA = 20/1. ^{1}H NMR (500 MHz, $CDCl_3$) δ 7.91 – 7.84 (m, 2H), 7.83 – 7.73 (m, 4H), 7.67 – 7.61 (m, 1H), 7.55 – 7.48 (m, 2H).^{13}C NMR (126 MHz, $CDCl_3$) δ 195.1, 141.2, 136.3, 133.3, 132.2, 130.3, 130.1, 128.7, 118.0, 115.7.

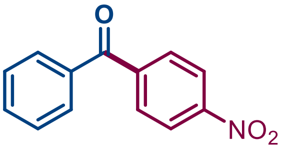

51

(4-Nitrophenyl)(phenyl)methanone (51) (CAS: 1144-74-7), white solid, 91% yield. Flash column chromatography conditions: PE/ EA = 20/1. ^{1}H NMR (500 MHz, $CDCl_3$) δ 8.38 – 8.31 (m, 2H), 7.97 – 7.90 (m, 2H), 7.83 – 7.77 (m, 2H), 7.68 – 7.62 (m, 1H), 7.56 – 7.49 (m, 2H). ^{13}C NMR (126 MHz, $CDCl_3$) δ 194.8, 149.8, 142.9, 136.3, 133.5, 130.7, 130.1, 128.7, 128.4, 124.4, 123.6.

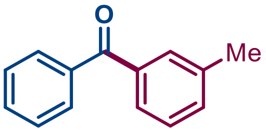

52

Phenyl(*m*-tolyl)methanone (52) (CAS: 643-65-2), white solid, 88% yield. Flash column chromatography conditions: PE/ EA = 20/1. ^{1}H NMR (500 MHz, CDCl$_3$) δ 7.84 – 7.76 (m, 2H), 7.63 (s, 1H), 7.62 – 7.55 (m, 2H), 7.48 (t, *J* = 7.7 Hz, 2H), 7.38 (dt, *J* = 15.0, 7.6 Hz, 2H), 2.42 (s, 3H).^{13}C NMR (126 MHz, CDCl$_3$) δ 197.0, 138.2, 137.8, 137.6, 133.2, 132.4, 130.5, 130.1, 128.3, 128.1, 127.4, 21.4.

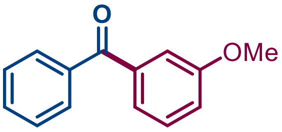

53

(3-Methoxyphenyl)(phenyl)methanone (53) (CAS: 6136-67-0), white solid, 83% yield. Flash column chromatography conditions: PE/ EA = 20/1. ^{1}H NMR (500 MHz, CDCl$_3$) δ 7.84 – 7.78 (m, 2H), 7.62 – 7.56 (m, 1H), 7.51 – 7.45 (m, 2H), 7.41 – 7.31 (m, 3H), 7.13 (ddd, *J* = 7.9, 2.7, 1.2 Hz, 1H), 3.86 (s, 3H). ^{13}C NMR (126 MHz, CDCl$_3$) δ 196.6, 159.7, 138.9, 137.6, 132.5, 130.1, 129.2, 128.3, 122.9, 118.9, 114.3, 55.5.

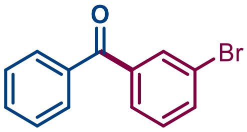

54

(3-Bromophenyl)(phenyl)methanone (54) (CAS: 1016-77-9), white solid, 86% yield. Flash column chromatography conditions: PE/ EA = 20/1. ^{1}H NMR (500 MHz, CDCl$_3$) δ 7.93 (t, *J* = 1.8 Hz, 1H), 7.79 (dd, *J* = 8.3, 1.3 Hz, 2H), 7.73 – 7.68 (m, 2H), 7.63 – 7.58 (m, 1H), 7.53 – 7.46 (m, 2H), 7.36 (t, *J* = 7.8 Hz, 1H). ^{13}C NMR (126 MHz, CDCl$_3$) δ 195.1, 139.5, 136.9, 135.3, 132.9, 132.8, 130.0, 129.9, 128.6, 128.5, 122.6.

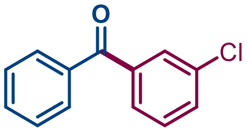

55

(3-Chlorophenyl)(phenyl)methanone (55) (CAS: 1016-78-0), white solid, 85% yield. Flash column chromatography conditions: PE/ EA = 20/1. ^{1}H NMR (500 MHz, CDCl$_3$) δ 7.79 (dt, *J* = 3.8, 1.8 Hz, 3H), 7.69 – 7.65 (m, 1H), 7.64 – 7.59 (m, 1H), 7.56 (ddd, *J* = 8.0, 2.1, 1.0 Hz, 1H), 7.50 (dd, *J* = 10.8, 4.7 Hz, 2H), 7.43 (t, *J* = 7.8 Hz, 1H). ^{13}C NMR (126 MHz, CDCl$_3$) δ 195.3, 139.3, 136.9, 134.6, 132.9, 132.4, 130.0, 129.9, 129.7, 128.5, 128.1.

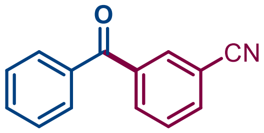

56

3-Benzoylbenzonitrile (56) (CAS: 6136-62-5), white solid, 93% yield. Flash column chromatography conditions: PE/ EA = 20/1. ^{1}H NMR (500 MHz, $CDCl_3$) δ 8.08 – 8.01 (m, 2H), 7.88 – 7.84 (m, 1H), 7.79 – 7.74 (m, 2H), 7.68 – 7.61 (m, 2H), 7.52 – 7.48 (m, 2H). ^{13}C NMR (126 MHz, $CDCl_3$) δ 194.4, 138.6, 136.3, 135.4, 133.9, 133.5, 133.3, 130.0, 129.4, 128.7, 118.0, 112.8.

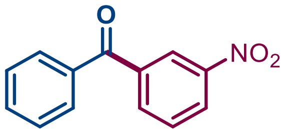

57

(3-Nitrophenyl)(phenyl)methanone (57), white solid, 90% yield. Flash column chromatography conditions: PE/ EA = 20/1. ^{1}H NMR (500 MHz, $CDCl_3$) δ 8.65 – 8.58 (m, 1H), 8.44 (ddd, *J* = 8.2, 2.3, 1.1 Hz, 1H), 8.18 – 8.10 (m, 1H), 7.83 – 7.77 (m, 2H), 7.71 (t, *J* = 7.9 Hz, 1H), 7.68 – 7.64 (m, 1H), 7.56 – 7.51 (m, 2H). ^{13}C NMR (126 MHz, $CDCl_3$) δ 194.8, 149.8, 142.9, 136.3, 133.5, 130.7, 130.1, 128.7, 128.4, 124.4, 123.6.

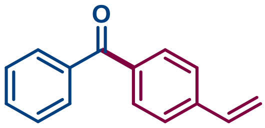

58

Phenyl(4-vinylphenyl)methanone (58) (CAS: 3139-85-3), white solid, 82% yield. Flash column chromatography conditions: PE/ EA = 20/1. ^{1}H NMR (500 MHz, $CDCl_3$) δ 7.84 – 7.76 (m, 4H), 7.62 – 7.56 (m, 1H), 7.55 – 7.46 (m, 4H), 6.78 (dd, *J* = 17.6, 10.9 Hz, 1H), 5.95 – 5.85 (m, 1H), 5.41 (d, *J* = 10.9 Hz, 1H). ^{13}C NMR (126 MHz, $CDCl_3$) δ 196.2, 141.6, 137.8, 136.7, 136.0, 132.4, 130.6, 130.0, 128.3, 126.1, 116.6.

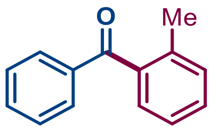

59

Phenyl(o-tolyl)methanone (59) (CAS: 131-58-8), white solid, 82% yield. Flash column chromatography conditions: PE/ EA = 20/1. ^{1}H NMR (500 MHz, $CDCl_3$) δ 7.85 – 7.78 (m, 2H), 7.58 (dd, *J* = 10.5, 4.3 Hz, 1H), 7.46 (t, *J* = 7.7 Hz, 2H), 7.40 (td, *J* = 7.5, 1.3 Hz, 1H), 7.34 – 7.28 (m, 2H), 7.28 – 7.22 (m, 1H), 2.34 (s, 3H). ^{13}C NMR (126 MHz, $CDCl_3$) δ 198.6, 138.6, 137.7, 136.7, 133.1, 131.0, 130.2, 130.1, 128.5, 128.4, 125.2, 19.9.

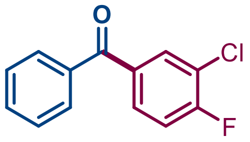

60

(3-Chloro-4-fluorophenyl)(phenyl)methanone (60) (CAS: 133280-45-2), white solid, 90% yield. Flash column chromatography conditions: PE/ EA = 20/1. ^{1}H NMR (500 MHz, $CDCl_3$) δ 7.90 (dd, *J* = 7.1, 2.1 Hz, 1H), 7.80 – 7.75 (m, 2H), 7.72 (ddd, *J* = 8.5, 4.6, 2.1 Hz, 1H), 7.65 – 7.59 (m, 1H), 7.55 – 7.48 (m, 2H), 7.28 – 7.23 (m, 1H). ^{13}C NMR (126 MHz, $CDCl_3$) δ 194.1, 160.7 (d, *J* = 256.3 Hz), 136.9, 134.7 (d, *J* = 3.7 Hz), 132.9 (s), 132.8, 130.4 (d, *J* = 8.2 Hz), 129.9, 128.5, 121.6 (d, *J* = 18.2 Hz), 116.6 (d, *J* = 21.7 Hz).

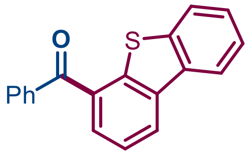

61

Dibenzo[*b*,*d*]thiophen-4-yl(phenyl)methanone (61) (CAS: 6407-31-4), white solid, 77% yield. Flash column chromatography conditions: PE/ EA = 20/1. ^{1}H NMR (500 MHz, $CDCl_3$) δ 8.41 (dd, *J* = 7.8, 1.0 Hz, 1H), 8.24 – 8.17 (m, 1H), 7.97 (dd, *J* = 6.5, 2.2 Hz, 1H), 7.92 (dd, *J* = 7.5, 1.0 Hz, 1H), 7.83 – 7.78 (m, 2H), 7.64 – 7.59 (m, 1H), 7.58 – 7.48 (m, 5H). ^{13}C NMR (126 MHz, $CDCl_3$) δ 195.8, 141.8, 140.8, 138.1, 137.4, 134.0, 131.9, 131.6, 130.3, 129.6, 128.3, 127.2, 125.8, 124.5, 123.7, 122.8, 121.4.

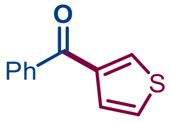

62

Phenyl(thiophen-3-yl)methanone (62) (CAS: 6453-99-2), white solid, 62% yield. Flash column chromatography conditions: PE/ EA = 20/1. ^{1}H NMR (500 MHz, $CDCl_3$) δ 7.94 (dd, *J* = 2.9, 1.2 Hz, 1H), 7.87 – 7.83 (m, 2H), 7.63 – 7.57 (m, 2H), 7.52 – 7.47 (m, 2H), 7.39 (dd, *J* = 5.1, 2.9 Hz, 1H).^{13}C NMR (126 MHz, $CDCl_3$) δ 190.0, 141.3, 138.6, 134.0, 132.3, 129.4, 128.6, 128.4, 126.2.

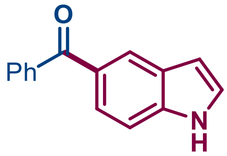

63

(1*H*-Indol-5-yl)(phenyl)methanone (63) (CAS: 134977-99-4), white solid, 68% yield. Flash column chromatography conditions: PE/ EA = 20/1. ^{1}H NMR (500 MHz, $CDCl_3$) δ 8.83 (s,

1H), 8.17 – 8.13 (m, 1H), 7.85 – 7.78 (m, 3H), 7.60 – 7.56 (m, 1H), 7.51 – 7.47 (m, 2H), 7.44 (d, *J* = 8.6 Hz, 1H), 7.28 (dd, *J* = 3.1, 2.5 Hz, 1H), 6.66 –6.30 (m, 1H). ^{13}C NMR (126 MHz, $CDCl_3$) δ 197.6, 139.0, 138.3, 131.7, 130.0, 129.7, 128.1, 127.2, 125.8, 125.3, 124.2, 111.1, 104.2.

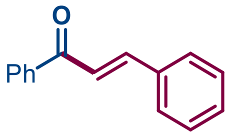

64

(*E*)-Chalcone (64) (CAS: 614-47-1), white solid, 50% yield. Flash column chromatography conditions: PE/ EA = 20/1. ^{1}H NMR (500 MHz, $CDCl_3$) δ 8.07 – 8.00 (m, 2H), 7.82 (d, *J* = 15.7 Hz, 1H), 7.65 (dd, *J* = 6.5, 2.9 Hz, 2H), 7.59 (t, *J* = 7.4 Hz, 1H), 7.57 – 7.48 (m, 3H), 7.44 – 7.37 (m, 3H). ^{13}C NMR (126 MHz, $CDCl_3$) δ 190.5, 144.8, 138.1, 134.8, 132.7, 130.5, 128.9, 128.6, 128.4, 128.4, 128.1, 122.0. ^{13}C NMR (126 MHz, $CDCl_3$) δ 190.5, 144.8, 138.1, 134.8, 132.7, 130.5, 128.9, 128.6, 128.4, 128.4, 122.0.

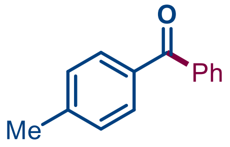

65

Phenyl(p-tolyl)methanone (65) (CAS: 134-84-9), white solid, 87% yield. Flash column chromatography conditions: PE/ EA = 20/1. ^{1}H NMR (500 MHz, $CDCl_3$) δ 7.81 – 7.76 (m, 2H), 7.74 – 7.71 (m, 2H), 7.60 – 7.56 (m, 1H), 7.50 – 7.46 (m, 2H), 7.28 (d, *J* = 7.9 Hz, 2H), 2.44 (s, 3H).^{13}C NMR (126 MHz, $CDCl_3$) δ 196.6, 143.3, 138.0, 134.9, 132.2, 130.3, 130.0 129.0, 128.2, 21.7.

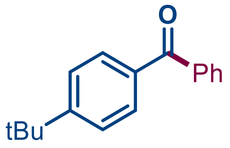

66

(*tert*-Butyl)phenyl)(phenyl)methanone (66) (CAS: 22679-54-5), white solid, 85% yield. Flash column chromatography conditions: PE/ EA = 20/1. ^{1}H NMR (500 MHz, $CDCl_3$) δ 7.83 – 7.79 (m, 2H), 7.79 – 7.75 (m, 2H), 7.60 – 7.56 (m, 1H), 7.52 – 7.46 (m, 4H), 1.37 (s, 9H). ^{13}C NMR (126 MHz, $CDCl_3$) δ 196.5, 156.2, 137.9, 134.8, 132.2, 130.2, 130.0, 128.2, 125.3, 35.1, 31.2.

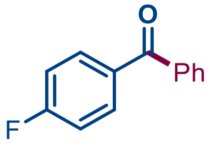

67

(4-Fluorophenyl)(phenyl)methanone (67) (CAS: 345-83-5), white solid, 70% yield. Flash column chromatography conditions: PE/ EA = 20/1. ^{1}H NMR (500 MHz, $CDCl_3$) δ 7.93 – 7.82 (m, 2H), 7.81 – 7.74 (m, 2H), 7.62 – 7.56 (m, 1H), 7.54 – 7.46 (m, 2H), 7.20 – 7.12 (m, 2H).^{13}C NMR (126 MHz, $CDCl_3$) δ 195.3, 165.4 (d, J = 252.0 Hz) 137.5, 133.8, 132.8,132.4, 129.9, 128.4, 115.5 (d, J = 21.9 Hz).

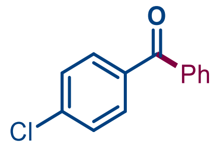

68

(4-Chlorophenyl)(phenyl)methanone (68) (CAS: 134-85-0), white solid, 86% yield. Flash column chromatography conditions: PE/ EA = 20/1. ^{1}H NMR (500 MHz, $CDCl_3$) δ 7.82 – 7.72 (m, 4H), 7.62 – 7.58 (m, 1H), 7.53 – 7.43 (m, 4H).^{13}C NMR (126 MHz, $CDCl_3$) δ 195.5, 138.9, 137.2, 135.9, 132.7, 131.5, 130.0, 128.7, 128.4.

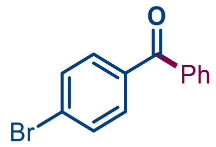

69

(4-Bromophenyl)(phenyl)methanone (69) (CAS: 90-90-4), white solid, 79% yield. Flash column chromatography conditions: PE/ EA = 20/1. ^{1}H NMR (500 MHz, $CDCl_3$) δ 7.77 (dd, J = 8.2, 1.2 Hz, 2H), 7.70 – 7.66 (m, 2H), 7.65 – 7.57 (m, 3H), 7.49 (dd, J = 10.7, 4.7 Hz, 2H). ^{13}C NMR (126 MHz, $CDCl_3$) δ 195.6, 137.1, 136.3, 132.7, 131.6, 131.5, 129.9, 128.4, 127.5.

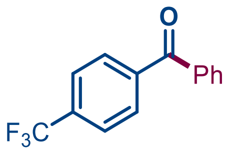

70

Phenyl(4-(trifluoromethyl)phenyl)methanone (70) (CAS: 728-86-9), white solid, 51% yield. Flash column chromatography conditions: PE/ EA = 20/1. ^{1}H NMR (500 MHz, $CDCl_3$) δ 7.90 (d, J = 8.0 Hz, 2H), 7.81 (dd, J = 8.2, 1.2 Hz, 2H), 7.76 (d, J = 8.2 Hz, 2H), 7.67 – 7.59 (m, 1H), 7.51 (dd, J = 10.8, 4.7 Hz, 2H). ^{13}C NMR (126 MHz, $CDCl_3$) δ 195.5, 140.7, 136.7, 133.8, 133.6, 133.1, 130.1 (d, J = 4.3 Hz), 128.5, 125.3 (q, J = 3.7 Hz), 123.64 (q, J = 272.6 Hz).

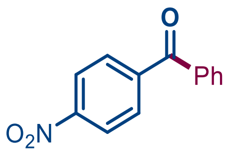

71

(4-Nitrophenyl)(phenyl)methanone (71) (CAS: 1144-74-7), white solid, 68% yield. Flash column chromatography conditions: PE/ EA = 20/1. ^{1}H NMR (500 MHz, CDCl3) δ 8.38 – 8.31 (m, 2H), 7.97 – 7.90 (m, 2H), 7.83 – 7.77 (m, 2H), 7.68 – 7.62 (m, 1H), 7.56 – 7.49 (m, 2H). ^{13}C NMR (126 MHz, CDCl3) δ 194.8, 149.8, 142.9, 136.3, 133.5, 130.7, 130.1, 128.7, 128.4, 124.4, 123.6.

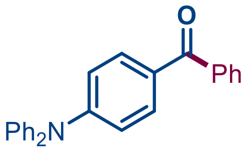

72

(4-(Diphenylamino)phenyl)(phenyl)methanone (72) (CAS: 16911-33-4), white solid, 88% yield. Flash column chromatography conditions: PE/ EA = 20/1. ^{1}H NMR (500 MHz, $CDCl_3$) δ 7.81 – 7.76 (m, 1H), 7.74 – 7.67 (m, 1H), 7.57 – 7.50 (m, 1H), 7.46 (t, *J* = 7.5 Hz, 1H), 7.38 – 7.30 (m, 2H), 7.21 – 7.17 (m, 2H), 7.15 (t, *J* = 7.4 Hz, 1H), 7.07 – 6.98 (m, 1H). ^{13}C NMR (126 MHz, $CDCl_3$) δ 195.1, 151.9, 146.4, 138.4, 131.9, 131.6, 129.6, 129.6, 129.5, 128.1, 125.9, 124.6, 119.5.

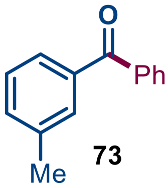

73

Phenyl(m-tolyl)methanone (73) (CAS: 643-65-2), white solid, 89% yield. Flash column chromatography conditions: PE/ EA = 20/1. ^{1}H NMR (500 MHz, $CDCl_3$) δ 7.84 – 7.76 (m, 2H), 7.63 (s, 1H), 7.62 – 7.55 (m, 2H), 7.48 (t, *J* = 7.7 Hz, 2H), 7.38 (dt, *J* = 15.0, 7.6 Hz, 2H), 2.42 (s, 3H).^{13}C NMR (126 MHz, $CDCl_3$) δ 197.0, 138.2, 137.8, 137.6, 133.2, 132.4, 130.5, 130.1, 128.3, 128.1, 127.4, 21.4.

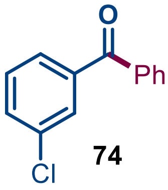

74

(3-Chlorophenyl)(phenyl)methanone (74) (CAS: 1016-78-0), white solid, 90% yield. Flash column chromatography conditions: PE/ EA = 20/1. ^{1}H NMR (500 MHz, $CDCl_3$) δ 7.79 (dt, *J* = 3.8, 1.8 Hz, 3H), 7.69 – 7.65 (m, 1H), 7.64 – 7.59 (m, 1H), 7.56 (ddd, *J* = 8.0, 2.1, 1.0 Hz, 1H), 7.50 (dd, *J* = 10.8, 4.7 Hz, 2H), 7.43 (t, *J* = 7.8 Hz, 1H). ^{13}C NMR (126 MHz, $CDCl_3$) δ 195.3, 139.3, 136.9, 134.6, 132.9, 132.4, 130.0, 129.9, 129.7, 128.5, 128.1.

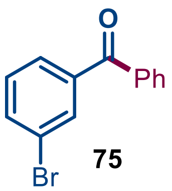

75

(3-Bromophenyl)(phenyl)methanone (75) (CAS: 1016-77-9), white solid, 98% yield. Flash column chromatography conditions: PE/ EA = 20/1. ^{1}H NMR (500 MHz, $CDCl_3$) δ 7.93 (t, *J* = 1.8 Hz, 1H), 7.79 (dd, *J* = 8.3, 1.3 Hz, 2H), 7.73 – 7.68 (m, 2H), 7.63 – 7.58 (m, 1H), 7.53 – 7.46 (m, 2H), 7.36 (t, *J* = 7.8 Hz, 1H). ^{13}C NMR (126 MHz, $CDCl_3$) δ 195.1, 139.5, 136.9, 135.3, 132.9, 132.8, 130.0, 129.9, 128.6, 128.5, 122.6.

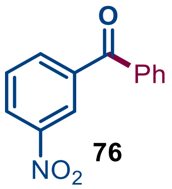

76

(3-Nitrophenyl)(phenyl)methanone (76) (CAS: 2243-80-3), white solid, 71% yield. Flash column chromatography conditions: PE/ EA = 20/1. ^{1}H NMR (500 MHz, $CDCl_3$) δ 8.65 – 8.58 (m, 1H), 8.44 (ddd, *J* = 8.2, 2.3, 1.1 Hz, 1H), 8.18 – 8.10 (m, 1H), 7.83 – 7.77 (m, 2H), 7.71 (t, *J* = 7.9 Hz, 1H), 7.68 – 7.64 (m, 1H), 7.56 – 7.51 (m, 2H). ^{13}C NMR (126 MHz, $CDCl_3$) δ 194.8, 149.8, 142.9, 136.3, 133.5, 130.7, 130.1, 128.7, 128.4, 124.4, 123.6.

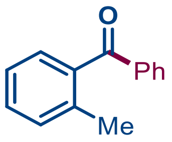

77

Phenyl(o-tolyl)methanone (77) (CAS: 131-58-8), white solid, 89% yield. Flash column chromatography conditions: PE/ EA = 20/1. ^{1}H NMR (500 MHz, CDCl3) δ 7.81 (d, J = 7.3 Hz, 2H), 7.58 (t, J = 7.4 Hz, 1H), 7.46 (t, J = 7.7 Hz, 2H), 7.42 – 7.38 (m, 1H), 7.31 (dd, J = 11.4, 7.6 Hz, 2H), 7.25 (dd, J = 10.4, 4.2 Hz, 1H), 2.34 (s, 3H). ^{13}C NMR (126 MHz, CDCl3) δ 198.7, 138.6, 137.7, 136.8, 133.2, 131.0, 130.3, 130.1, 128.5, 128.4, 125.2, 20.0.

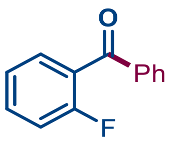

78

(2-Fluorophenyl)(phenyl)methanone (78) (CAS: 342-24-5), white solid, 78% yield. Flash column chromatography conditions: PE/ EA = 20/1. ^{1}H NMR (500 MHz, $CDCl_3$) δ 7.84 (dd, *J* = 5.3, 4.2 Hz, 2H), 7.63 – 7.50 (m, 3H), 7.50 – 7.45 (m, 2H), 7.30 – 7.25 (m, 1H), 7.17 (dd, *J* = 13.5, 4.7 Hz, 1H). ^{13}C NMR (126 MHz, $CDCl_3$) δ 193.5, 160.1 (d, *J* = 252.4 Hz), 137.4,

133.44 (s), 133.1 (d, J = 8.3 Hz), 130.8 (d, J = 2.8 Hz), 129.8, 128.5, 127.0 (d, J = 14.8 Hz), 124.3 (d, J = 3.6 Hz), 116.3 (d, J = 21.7 Hz).

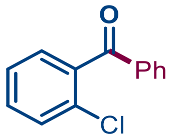

79

(2-Chlorophenyl)(phenyl)methanone (79) (CAS: 5162-03-8), white solid, 77% yield. Flash column chromatography conditions: PE/ EA = 20/1. ^{1}H NMR (500 MHz, $CDCl_3$) δ 7.87 – 7.79 (m, 2H), 7.63 – 7.57 (m, 1H), 7.51 – 7.43 (m, 4H), 7.40 – 7.33 (m, 2H).^{13}C NMR (126 MHz, $CDCl_3$) δ 195.3, 138.6 , 136.5, 133.8, 131.2 (d, J = 17.9 Hz), 130.1 (d, J = 1.6 Hz), 129.1, 128.6, 126.7.

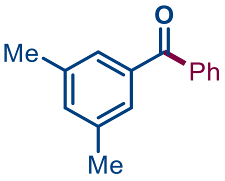

80

(3,5-Dimethylphenyl)(phenyl)methanone (80) (CAS: 13319-70-5), white solid, 81% yield. Flash column chromatography conditions: PE/ EA = 20/1. ^{1}H NMR (500 MHz, $CDCl_3$) δ 7.82 – 7.78 (m, 2H), 7.60 – 7.56 (m, 1H), 7.50 – 7.46 (m, 2H), 7.40 (s, 2H), 7.22 (s, 1H), 2.38 (s, 6H). ^{13}C NMR (126 MHz, $CDCl_3$) δ 197.2, 138.0, 137.9, 137.7 , 134.1, 132.3, 130.0, 128.2, 127.8, 21.3.

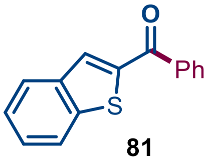

81

Benzo[*b*]thiophen-2-yl(phenyl)methanone (81) (CAS: 6454-02-0), white solid, 84% yield. Flash column chromatography conditions: PE/ EA = 20/1. ^{1}H NMR (500 MHz, $CDCl_3$) δ 7.96 – 7.83 (m, 5H), 7.67 – 7.60 (m, 1H), 7.56 – 7.52 (m, 2H), 7.49 (ddd, J = 8.2, 7.1, 1.2 Hz, 1H), 7.42 (ddd, J = 8.1, 7.2, 1.1 Hz, 1H). ^{13}C NMR (126 MHz, $CDCl_3$) δ 189.7, 143.1, 142.7, 139.0, 137.8, 132.5, 132.2, 129.3, 128.5, 127.4, 126.0, 125.0, 122.9.

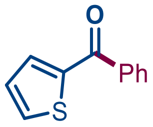

82

Phenyl(thiophen-2-yl)methanone (82) (CAS: 135-00-2), white solid, 73% yield. Flash column chromatography conditions: PE/ EA = 20/1. ^{1}H NMR (500 MHz, $CDCl_3$) δ 7.89 –

7.84 (m, 2H), 7.72 (dd, J = 4.9, 1.1 Hz, 1H), 7.65 (dd, J = 3.8, 1.1 Hz, 1H), 7.62 – 7.56 (m, 1H), 7.53 – 7.45 (m, 2H), 7.16 (dd, J = 4.9, 3.8 Hz, 1H). ^{13}C NMR (126 MHz, $CDCl_3$) δ 188.2, 143.6, 138.1, 134.8, 134.2, 132.2, 129.1, 128.4, 127.9.

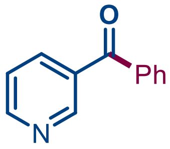

83

Phenyl(pyridin-3-yl)methanone (83) (CAS: 5424-19-1), white solid, 60% yield. Flash column chromatography conditions: PE/ EA = 20/1. ^{1}H NMR (500 MHz, $CDCl_3$) δ 9.01 (s, 1H), 8.83 (s, 1H), 8.13 (dt, J = 7.9, 1.9 Hz, 1H), 7.87 – 7.78 (m, 2H), 7.67 – 7.60 (m, 1H), 7.51 (dd, J = 10.8, 4.8 Hz, 2H), 7.47 (dd, J = 7.8, 4.8 Hz, 1H). ^{13}C NMR (126 MHz, $CDCl_3$) δ 194.8, 152.6, 150.7, 137.3, 136.6, 133.2, 130.0, 128.6, 123.5.

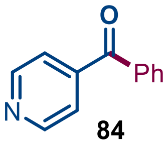

84

Phenyl(pyridin-4-yl)methanone (84) (CAS: 14548-46-0), white solid, 82% yield. Flash column chromatography conditions: PE/ EA = 20/1. ^{1}H NMR (500 MHz, $CDCl_3$) δ 8.86 (s, 1H), 7.89 – 7.76 (m, 1H), 7.69 – 7.56 (m, 2H), 7.55 – 7.41 (m, 1H). ^{13}C NMR (126 MHz, $CDCl_3$) δ 195.1, 150.1, 144.5, 135.8, 133.5, 130.1, 128.6, 123.1.

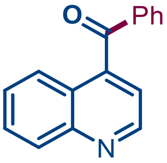

85

Phenyl(quinolin-3-yl)methanone (85) (CAS: 54885-00-6), white solid, 61% yield. Flash column chromatography conditions: PE/ EA = 20/1. ^{1}H NMR (500 MHz, $CDCl_3$) δ 9.32 (d, J = 2.2 Hz, 1H), 8.56 (d, J = 1.8 Hz, 1H), 8.20 (dd, J = 8.5, 0.6 Hz, 1H), 7.92 (dd, J = 8.1, 1.3 Hz, 1H), 7.89 – 7.82 (m, 3H), 7.70 – 7.60 (m, 2H), 7.59 – 7.48 (m, 2H). ^{13}C NMR (126 MHz, $CDCl_3$) δ 194.9, 150.3, 149.5, 138.8, 137.0, 133.1, 131.8, 130.1, 130.0, 129.5, 129.1, 128.6, 127.6, 126.6.

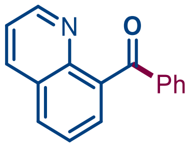

86

Phenyl(quinolin-8-yl)methanone (86) (CAS: 54885-04-0), white solid, 55% yield. Flash column chromatography conditions: PE/ EA = 20/1. ^{1}H NMR (500 MHz, $CDCl_3$) δ 8.84 (dd, J = 4.2, 1.8 Hz, 1H), 8.21 (dd, J = 8.3, 1.7 Hz, 1H), 7.96 (dd, J = 8.2, 1.4 Hz, 1H), 7.87 – 7.80 (m, 2H), 7.74 (dd, J = 7.0, 1.4 Hz, 1H), 7.63 (dd, J = 8.1, 7.1 Hz, 1H), 7.59 – 7.50 (m, 1H), 7.41 (ddd, J = 8.4, 3.8, 1.5 Hz, 3H). ^{13}C NMR (126 MHz, $CDCl_3$) δ 197.9, 150.8, 146.1, 139.3, 137.8, 136.0, 133.2, 130.2, 129.7, 128.3, 128.2, 128.2, 125.8, 121.6.

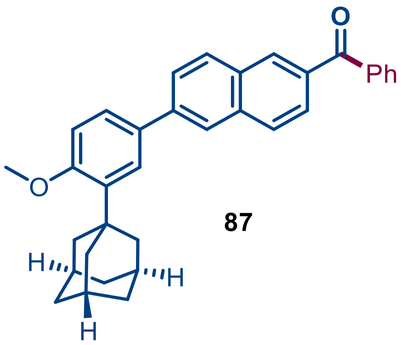

87

Phenyl(quinolin-8-yl)methanone (87), white solid, 80% yield. Flash column chromatography conditions: PE/ EA = 20/1. ^{1}H NMR (500 MHz, $CDCl_3$) δ 8.29 (s, 1H), 8.08 – 8.04 (m, 1H), 7.97 (dt, J = 8.6, 7.1 Hz, 3H), 7.90 – 7.86 (m, 2H), 7.82 (dd, J = 8.6, 1.8 Hz, 1H), 7.66 – 7.61 (m, 2H), 7.59 – 7.50 (m, 3H), 7.01 (d, J = 8.5 Hz, 1H), 3.92 (s, 3H), 2.21 (d, J = 2.7 Hz, 6H), 2.12 (s, 3H), 1.82 (s, 6H). ^{13}C NMR (126 MHz, $CDCl_3$) δ 196.7, 159.0, 141.5, 139.1, 138.1, 135.8, 134.4, 132.5, 132.3, 131.8, 131.0, 130.1, 129.8, 128.39, 128.35, 126.6, 126.2, 126.0, 125.8, 124.8, 112.2, 55.2, 40.6, 37.2, 29.1. HRMS (ESI) calcd for $C_{34}H_{33}O_2^+$ $[M+H]^+$: 473.2475, found 473.2474.

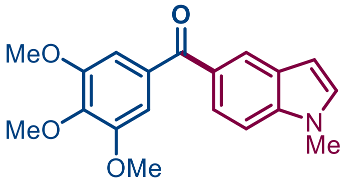

88

(1-Methyl-1*H*-indol-5-yl)(3,4,5-trimethoxyphenyl)methanone (88) (CAS: 946077-08-3), white solid, 63% yield. Flash column chromatography conditions: PE/ EA = 20/1. ^{1}H NMR (500 MHz, $CDCl_3$) δ 8.13 (d, J = 1.2 Hz, 1H), 7.79 (dd, J = 8.6, 1.6 Hz, 1H), 7.39 (d, J = 8.6 Hz, 1H), 7.14 (d, J = 3.1 Hz, 1H), 7.08 (s, 2H), 6.59 (d, J = 3.1 Hz, 1H), 3.94 (s, 3H), 3.87 (s, 6H), 3.84 (s, 3H). ^{13}C NMR (126 MHz, $CDCl_3$) δ 196.4, 152.7, 141.3, 138.9, 134.2, 130.5, 129.3, 127.7, 125.1, 123.8, 109.1, 107.6, 102.9, 61.0, 56.3, 33.1.

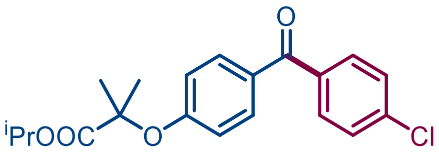

89

Isopropyl 2-(4-(4-chlorobenzoyl)phenoxy)-2-methylpropanoate (89) (CAS: 49562-28-9), white solid, 90% yield. Flash column chromatography conditions: PE/ EA = 20/1. ^{1}H NMR (500 MHz, $CDCl_3$) δ 7.78 – 7.65 (m, 4H), 7.49 – 7.39 (m, 2H), 6.94 – 6.80 (m, 2H), 5.08 (dt, *J* = 12.5, 6.3 Hz, 1H), 1.66 (s, 6H), 1.26 – 1.01 (m, 6H). ^{13}C NMR (126 MHz, $CDCl_3$) δ 194.2, 173.1, 159.7, 138.3, 136.4, 131.9, 131.1, 130.2, 128.5, 117.2, 79.4, 69.3, 25.4, 21.5.

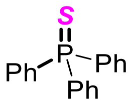

91

Triphenylphosphine sulfide (91), white solid, 95% yield. Flash column chromatography conditions: PE/ EA = 20/1. ^{1}H NMR (500 MHz, CDCl3) δ 7.78 – 7.69 (m, 6H), 7.55 – 7.48 (m, 3H), 7.47 – 7.40 (m, 6H). ^{13}C NMR (126 MHz, CDCl3) δ 132.8 (d, J = 85.1 Hz), 132.2 (d, J = 10.7 Hz), 131.5 (d, J = 2.9 Hz), 128.4 (d, J = 12.5 Hz).

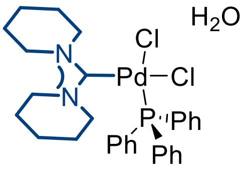

94

Pd-carbene intermediate (94), yellow solid, 9% yield. ^{1}H NMR (500 MHz, $CDCl_3$) δ 7.69 (s, 5H), 7.52 – 7.35 (m, 10H), 3.33 (s, 4H), 1.58 – 1.20 (m, 16H). ^{13}C NMR (126 MHz, CDCl3) δ 198.3, 134.5, 131.0, 128.6, 128.5, 54.6, 25.4, 23.7.

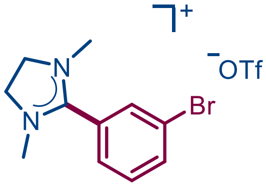

97

2-(3-Bromophenyl)-1,3-dimethylimidazolidine, trifluoromethanesulfonate salt (97), colorless oil, 85% yield. Flash column chromatography conditions: DCM/ MeOH = 20/1. ^{1}H NMR (500 MHz, $CDCl_3$) δ 7.77 (d, *J* = 8.1 Hz, 1H), 7.73 (t, *J* = 1.6 Hz, 1H), 7.63 (d, *J* = 7.8 Hz, 1H), 7.50 (t, *J* = 7.9 Hz, 1H), 4.16 (s, 4H), 2.97 (s, 6H). ^{19}F NMR (471 MHz, $CDCl_3$) δ -78.3. ^{13}C NMR (126 MHz, $CDCl_3$) δ 164.9, 135.9, 131.5, 131.0, 127.4, 123.7, 123.6, 120.8 (q, *J* = 320.4 Hz), 50.5, 34.7. HRMS (ESI) m/z calcd. for $C_{11}H_{14}BrN_2^+$ $[M]^+$: 253.0335, found 253.0334.

(91%) 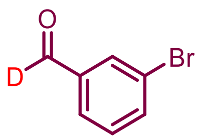

98

Deuterated 3-bromobenzaldehyde (98, colorless oil, 72% yield. Flash column chromatography conditions: PE/ EA = 50/1. ^{1}H NMR (500 MHz, $CDCl_3$) δ 9.96 (s, 0.09H), 8.01 (m, 1H), 7.84 – 7.79 (m, 1H), 7.75 (m, 1H), 7.42 (t, *J* = 7.8 Hz, 1H). ^{13}C NMR (126 MHz, $CDCl_3$) δ 190.7 – 190.1 (t, *J* = 320.4 Hz) 137.9 – 137.8 (t, *J* = 320.4 Hz), 137.3, 132.3, 130.6, 128.3, 123.3. HRMS (ESI) m/z calcd. for $C_7H_5DBrO^+$ $[M+H]^+$: 185.9659, found 185.9655.

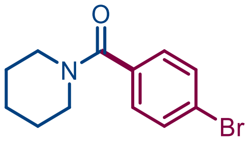

104

(4-Bromophenyl)(piperidin-1-yl)methanone (104) (CAS: 98612-93-2), white solid, 91% yield. Flash column chromatography conditions: PE/ EA = 20/1. ^{1}H NMR (500 MHz, $CDCl_3$) δ 7.55 – 7.50 (m, 2H), 7.28 – 7.24 (m, 2H), 3.49 (d, *J* = 185.1 Hz, 4H), 1.69 – 1.47 (m, 6H); ^{13}C NMR (126 MHz, $CDCl_3$) δ 169.2, 135.3, 131.6, 128.6, 123.6, 48.8, 43.2, 26.6, 25.6, 24.5.

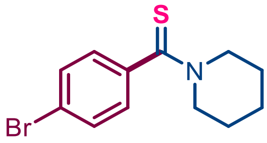

105

(4-Bromophenyl)(piperidin-1-yl)methanethione (105) (CAS: 72116-31-5), white solid, 95% yield. Flash column chromatography conditions: PE/ EA = 10/1. ^{1}H NMR (500 MHz, $CDCl_3$) δ 7.47 (d, *J* = 8.3 Hz, 2H), 7.15 (d, *J* = 8.3 Hz, 2H), 4.44 – 4.22 (m, 2H), 3.61 – 3.40 (m, 2H), 1.88 – 1.67 (m, 4H), 1.60 – 1.54 (m, 2H). ^{13}C NMR (126 MHz, $CDCl_3$) δ 198.1, 142.1, 131.6, 127.2, 122.5, 53.3, 50.7, 26.9, 25.5, 24.1.

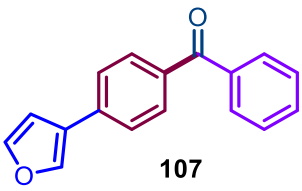

107

(4-(Furan-3-yl)phenyl)(phenyl)methanone (107), white solid, 86% yield. Flash column chromatography conditions: PE/ EA = 20/1. ^{1}H NMR (500 MHz, $CDCl_3$) δ 7.86 – 7.80 (m, 5H), 7.62 – 7.57 (m, 3H), 7.50 (ddd, *J* = 13.7, 5.4, 1.7 Hz, 3H), 6.76 (dd, *J* = 1.8, 0.9 Hz, 1H). ^{13}C NMR (126 MHz, $CDCl_3$) δ 196.1, 144.2, 139.6, 137.8, 136.7, 135.9, 132.3, 130.9, 130.0, 128.3, 125.7, 125.5, 108.7.

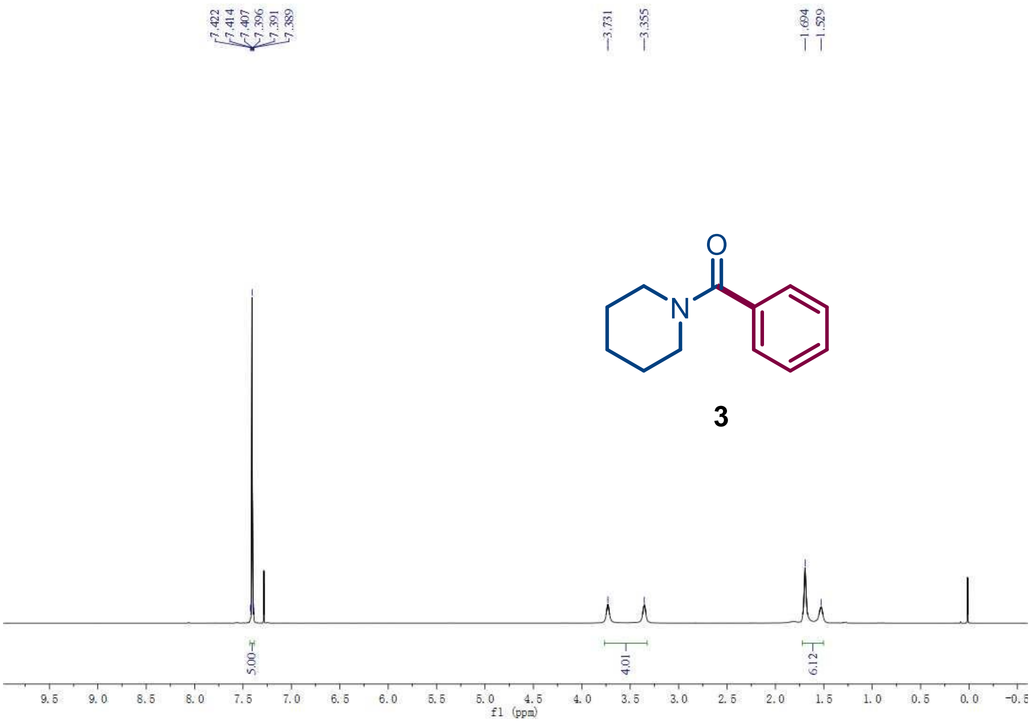

3

Supplementary Figure 14. ^{1}H NMR Spectrum of Compound **3**

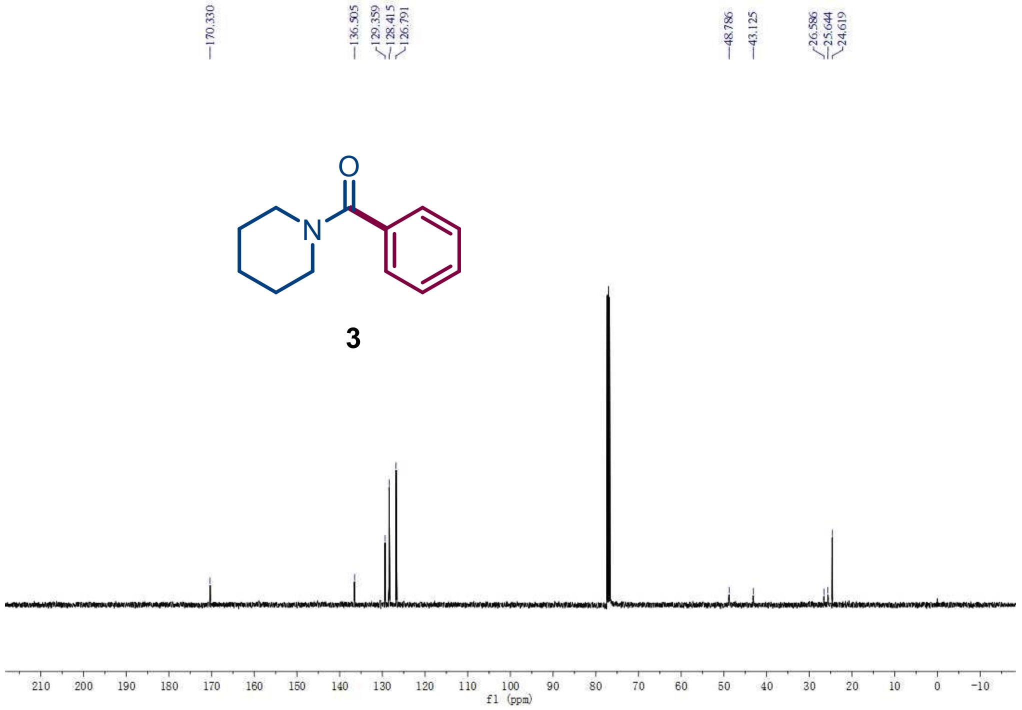

3

Supplementary Figure 15. ^{13}C NMR Spectrum of Compound **3**

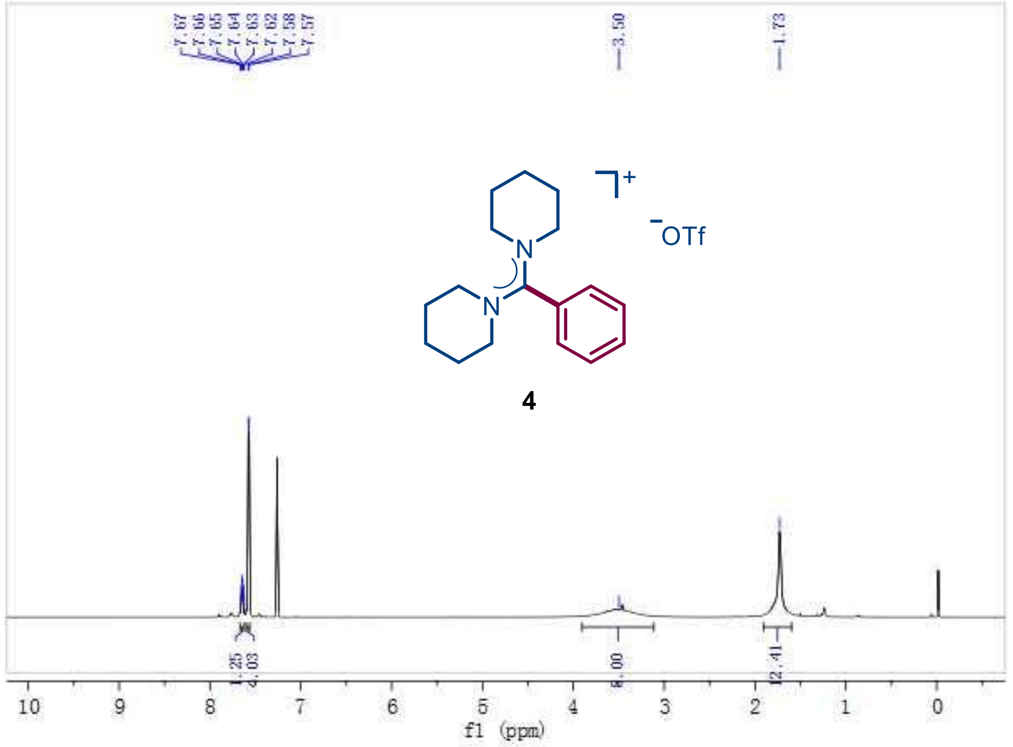

Supplementary Figure 16. ^{1}H NMR Spectrum of Compound **4**

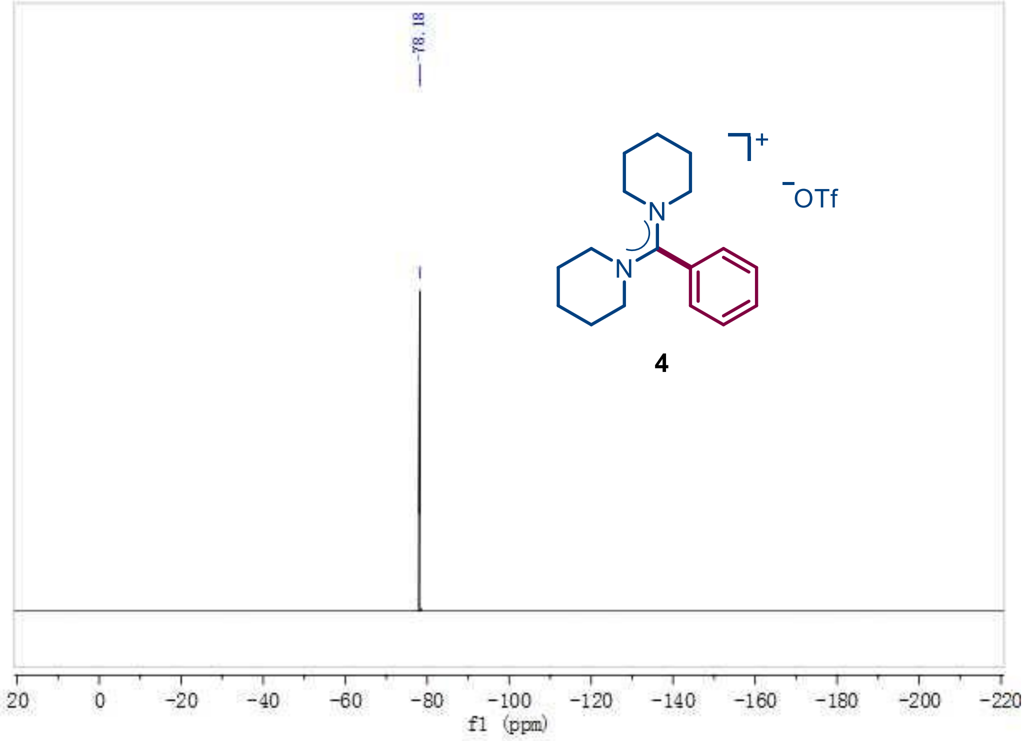

Supplementary Figure 17. ^{19}F NMR Spectrum of Compound **4**

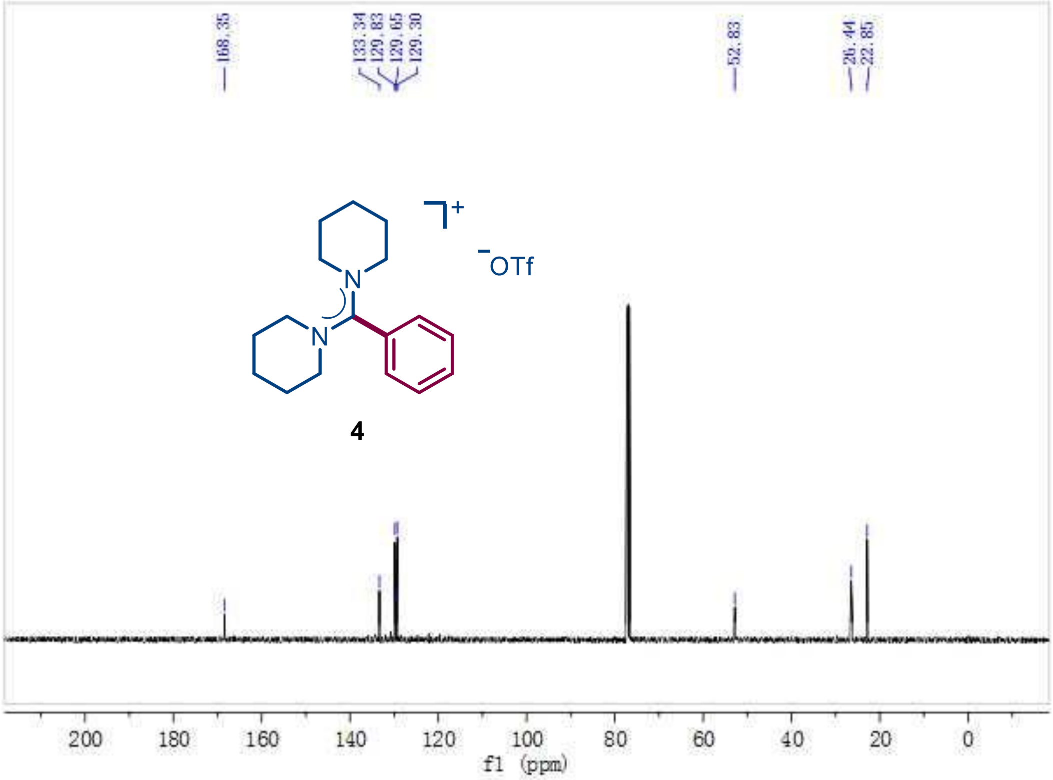

Supplementary Figure 18. ^{13}C NMR Spectrum of Compound **4**

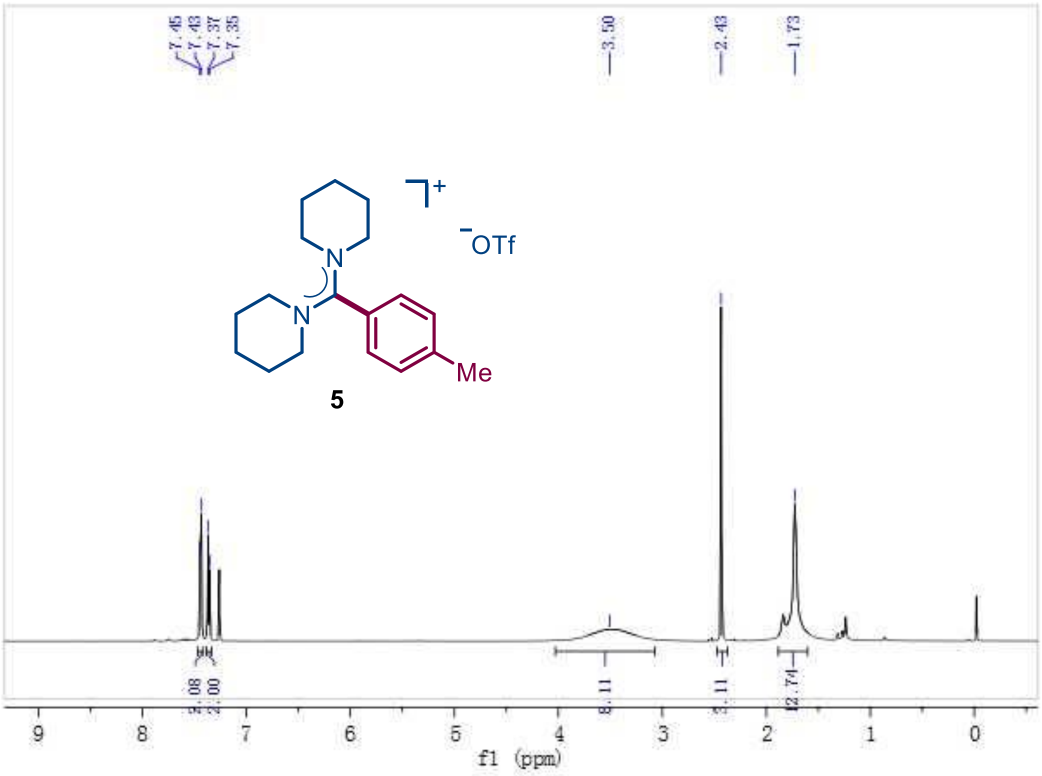

Supplementary Figure 19. ^{1}H NMR Spectrum of Compound **5**

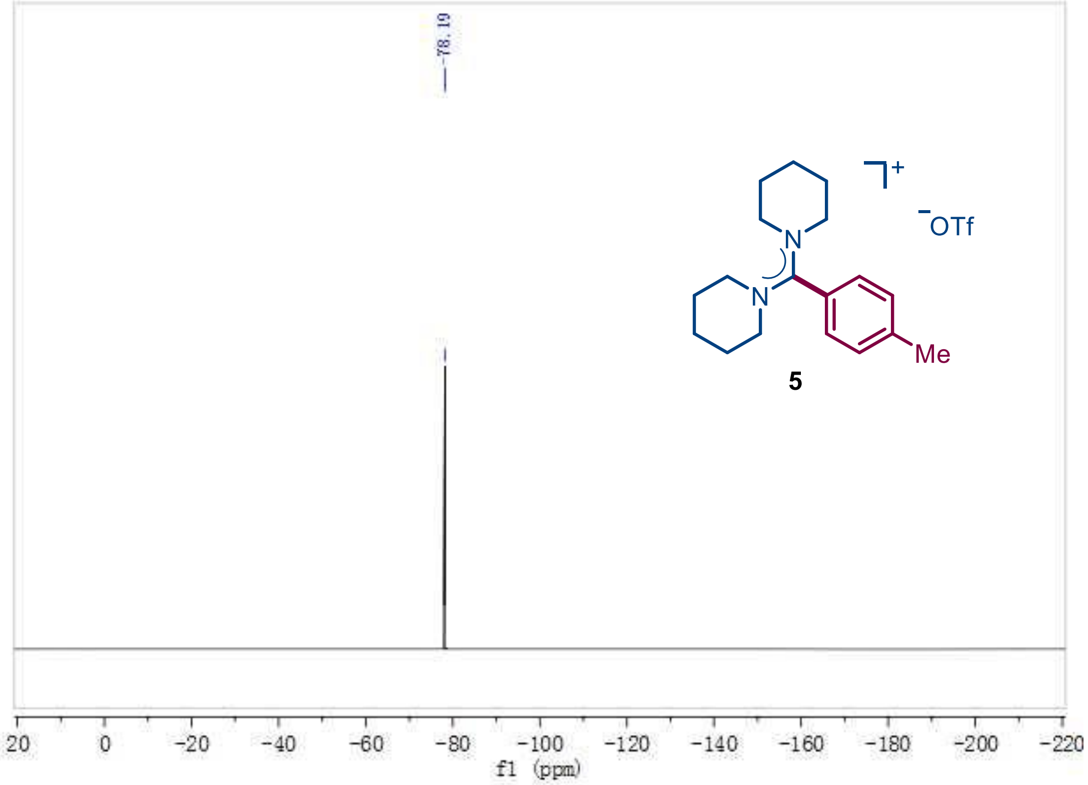

Supplementary Figure 20. ^{19}F NMR Spectrum of Compound **5**

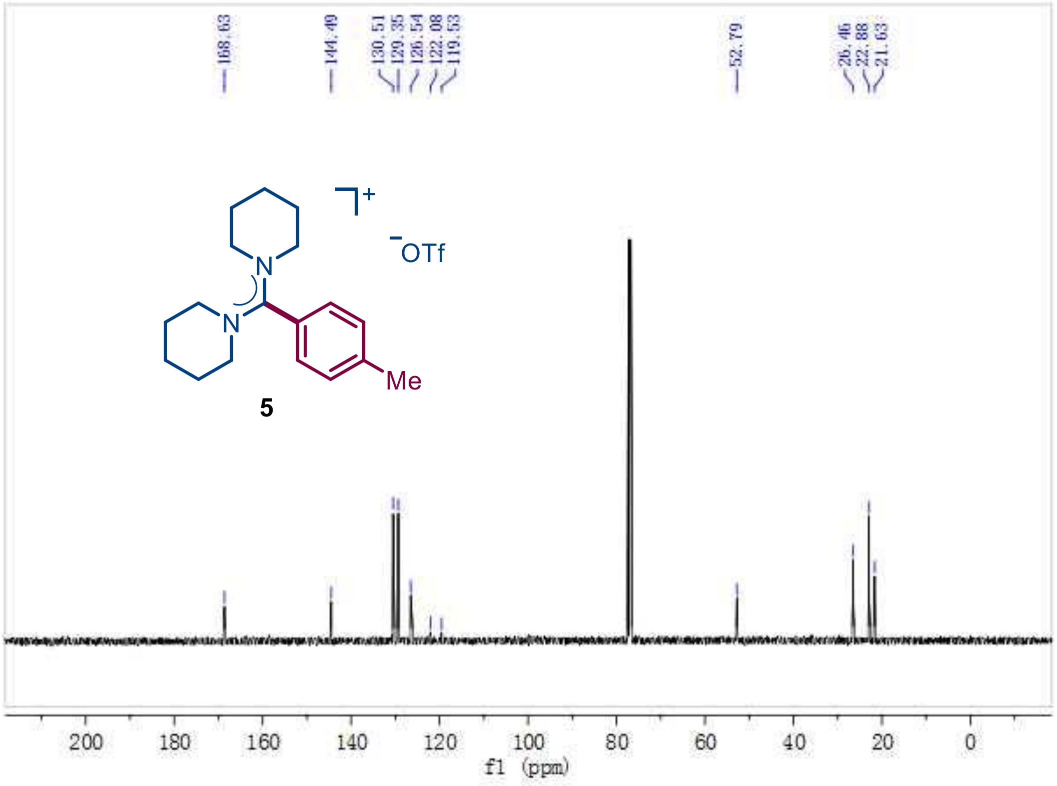

Supplementary Figure 21. ^{13}C NMR Spectrum of Compound **5**

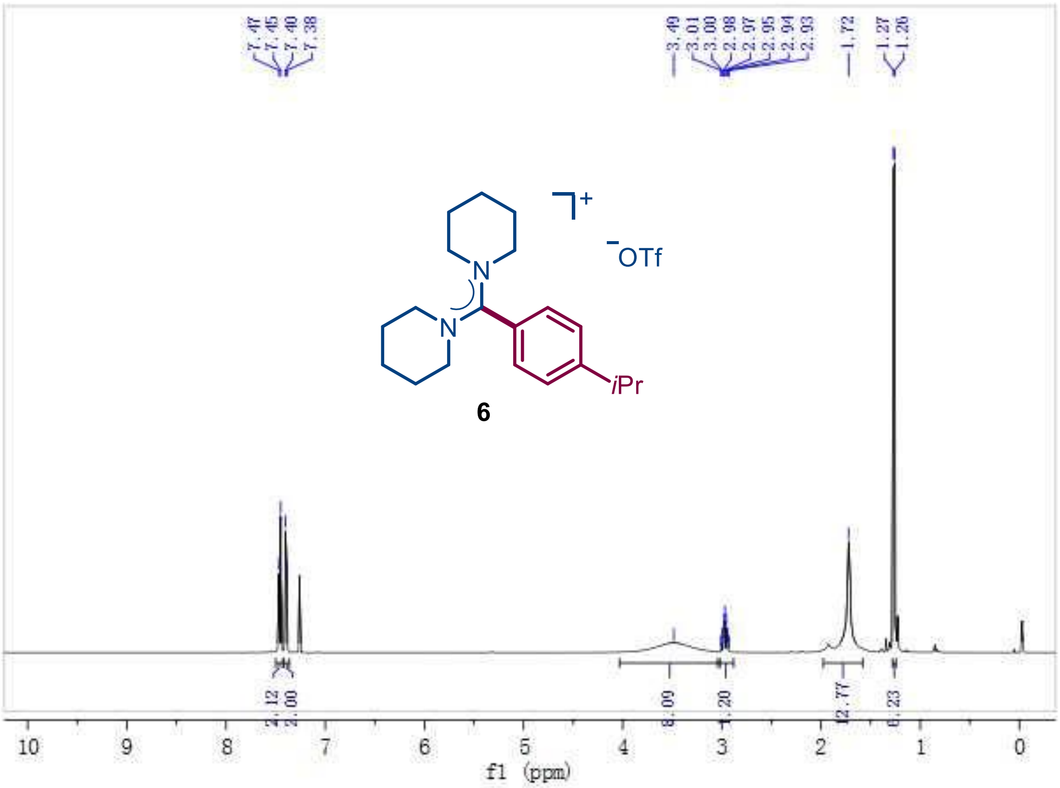

Supplementary Figure 22. ^{1}H NMR Spectrum of Compound **6**

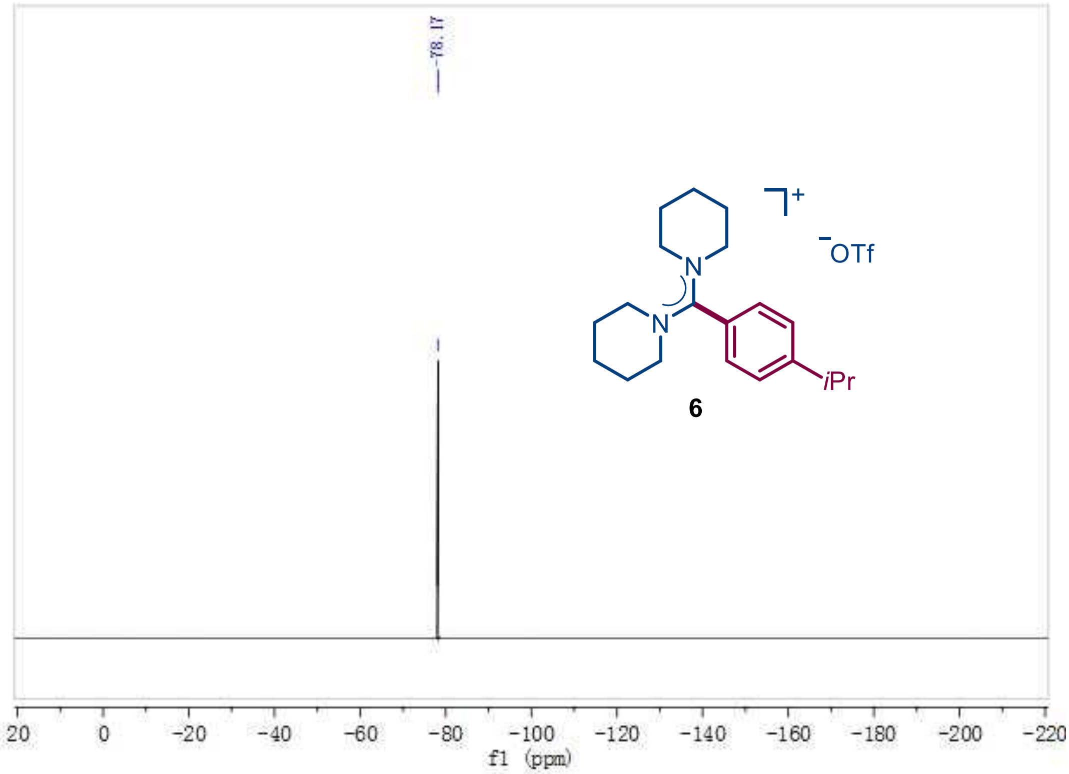

Supplementary Figure 23. ^{19}F NMR Spectrum of Compound **6**

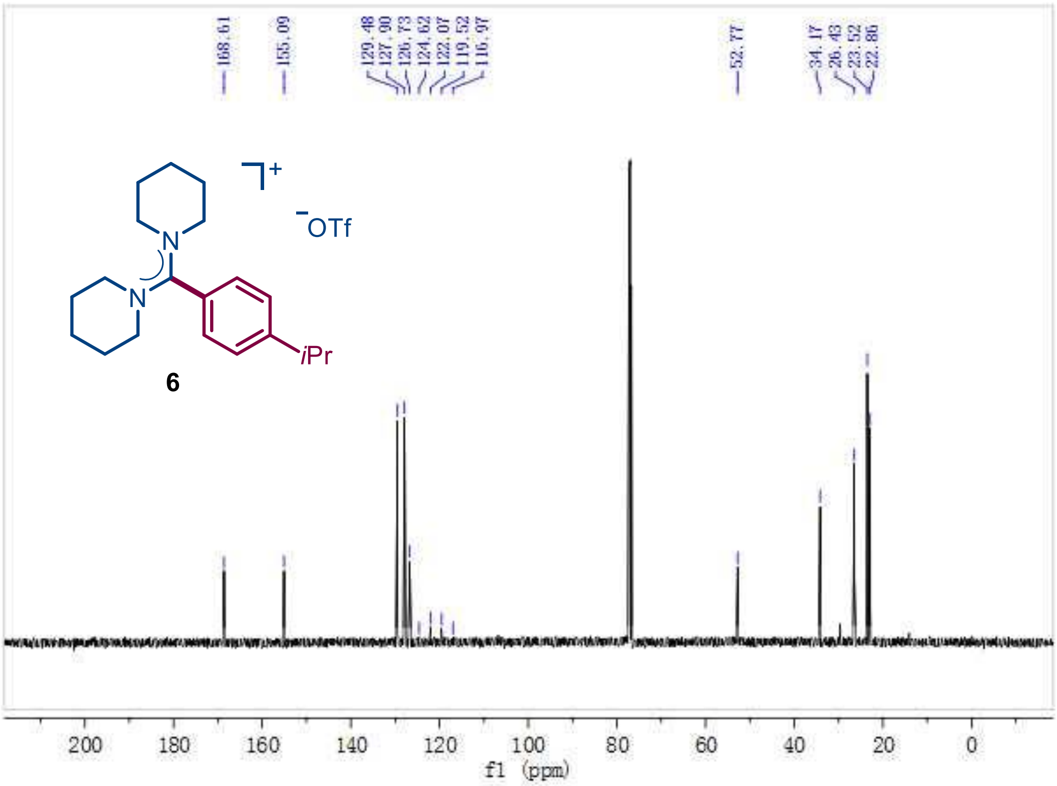

Supplementary Figure 24. ^{13}C NMR Spectrum of Compound **6**

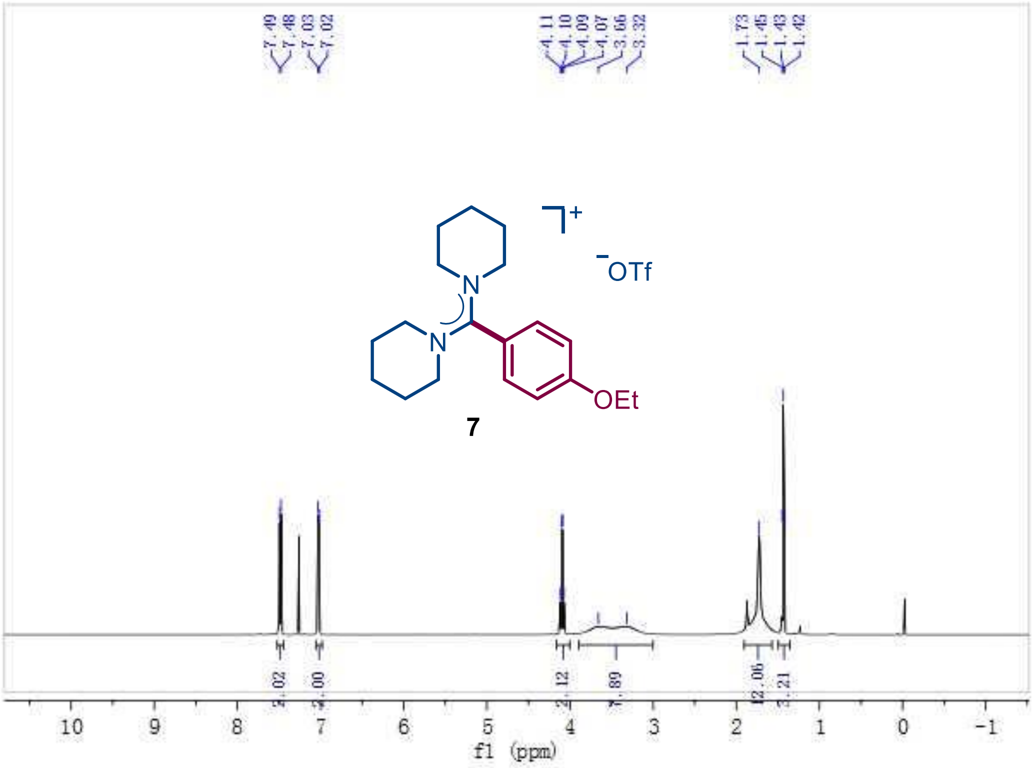

Supplementary Figure 25. ^{1}H NMR Spectrum of Compound **7**

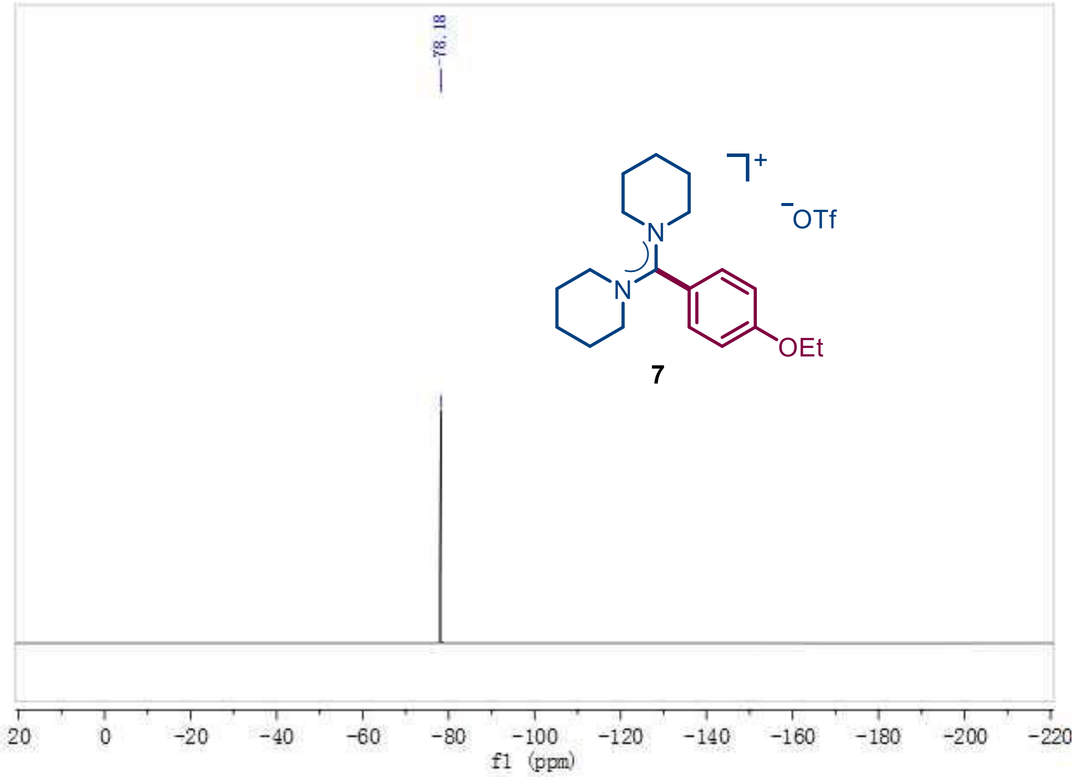

Supplementary Figure 26. ^{19}F NMR Spectrum of Compound **7**

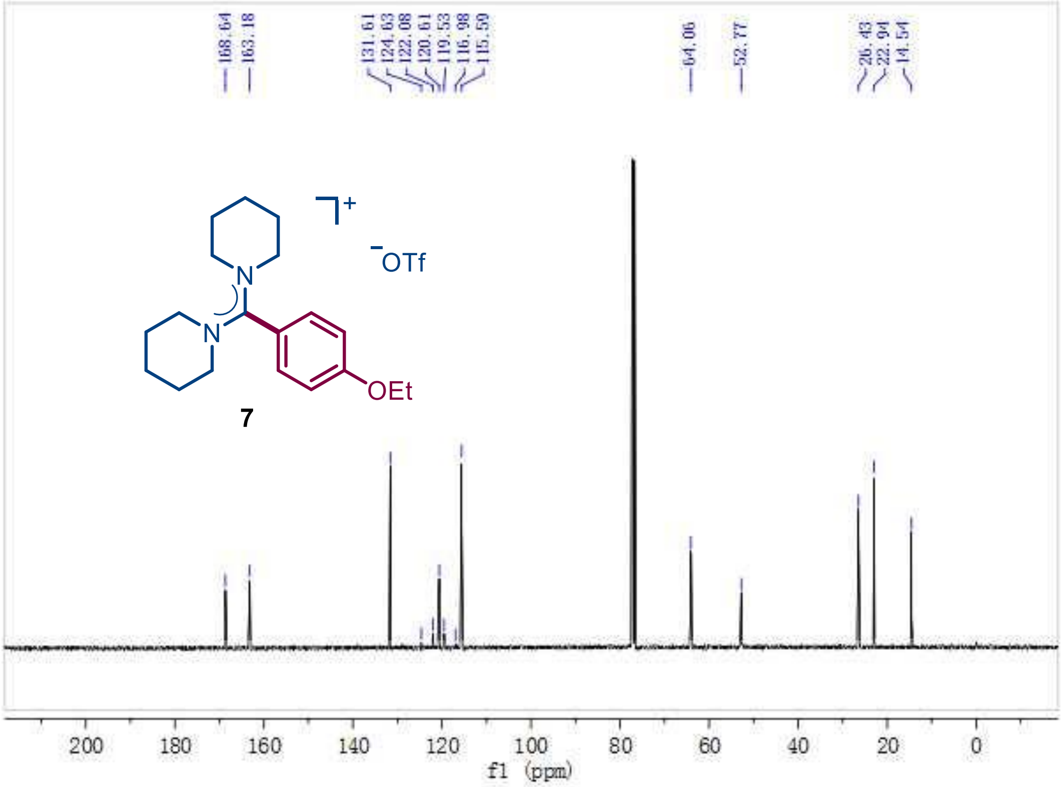

Supplementary Figure 27. ^{13}C NMR Spectrum of Compound **7**

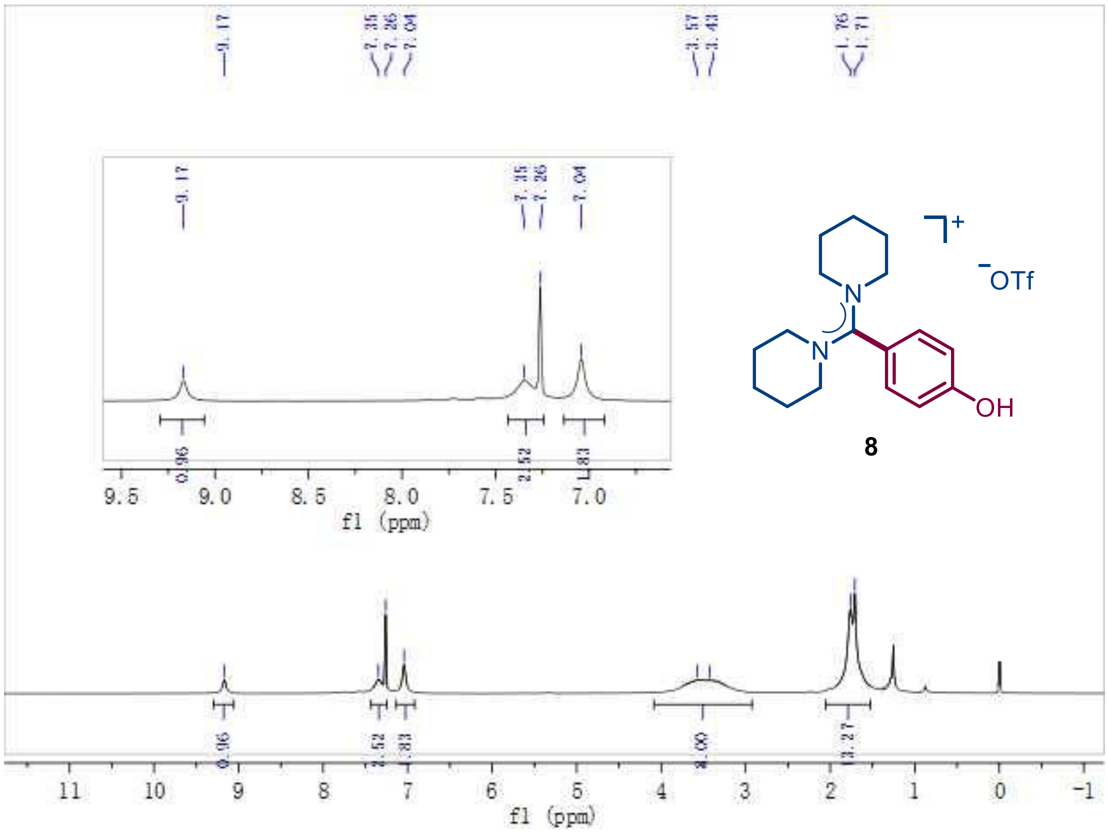

Supplementary Figure 28. ^{1}H NMR Spectrum of Compound **8**

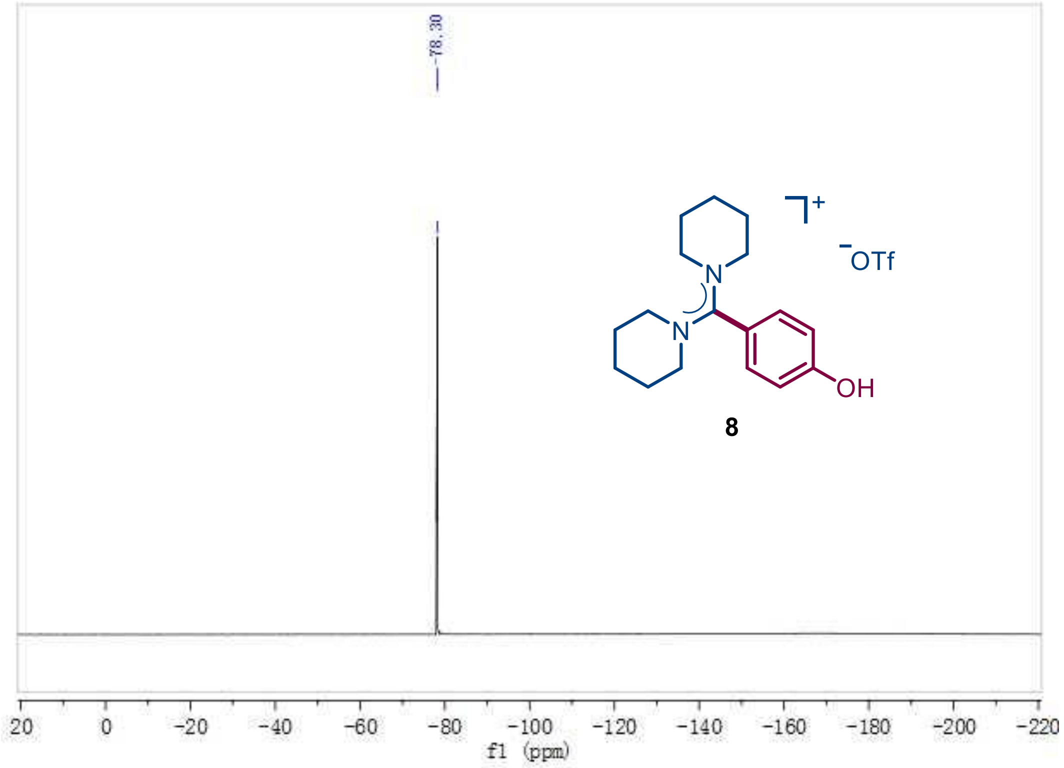

Supplementary Figure 29. ^{19}F NMR Spectrum of Compound **8**

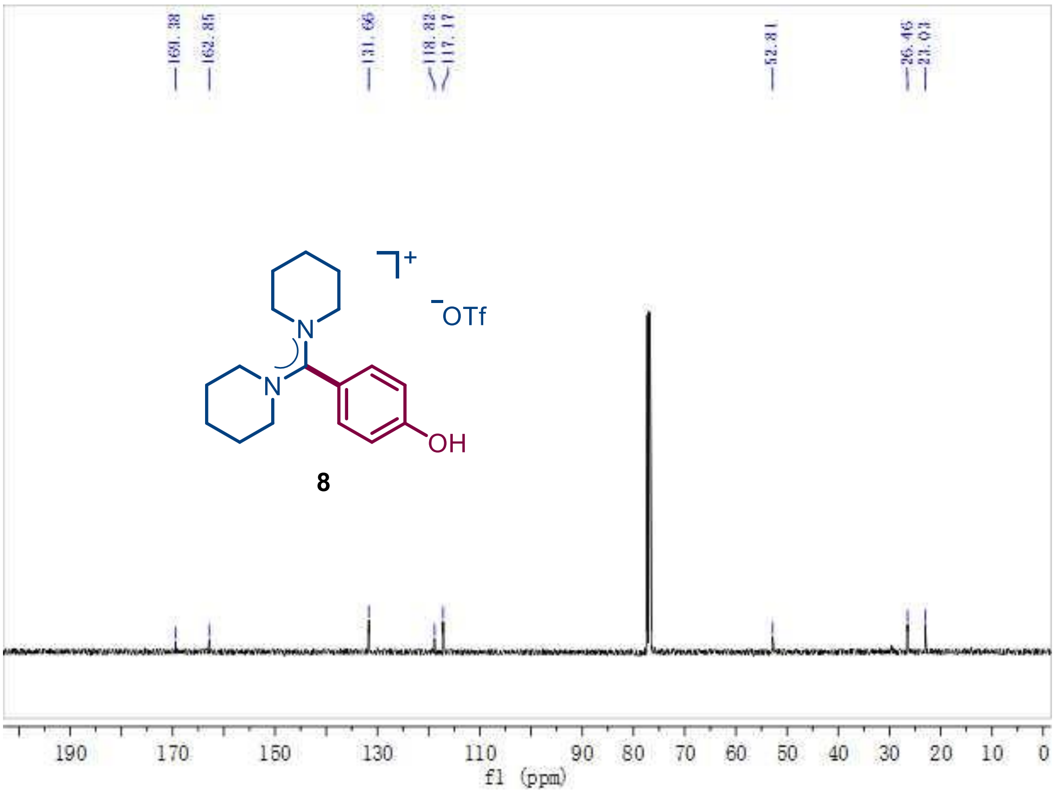

Supplementary Figure 30. ^{13}C NMR Spectrum of Compound **8**

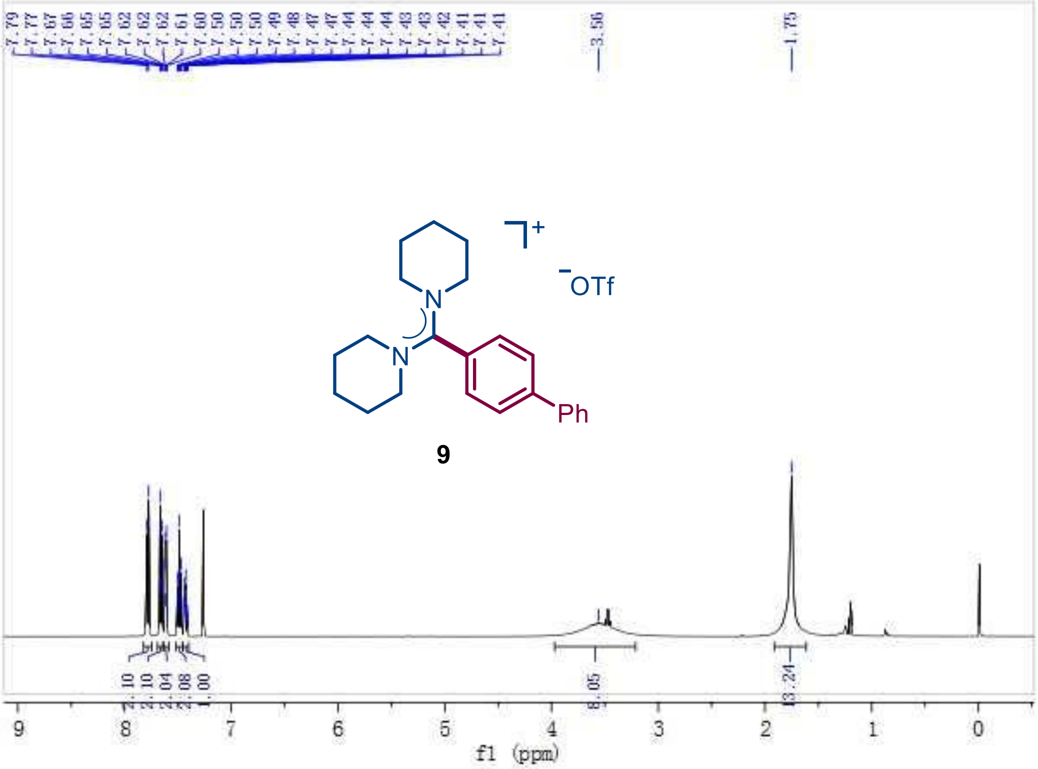

Supplementary Figure 31. ^{1}H NMR Spectrum of Compound **9**

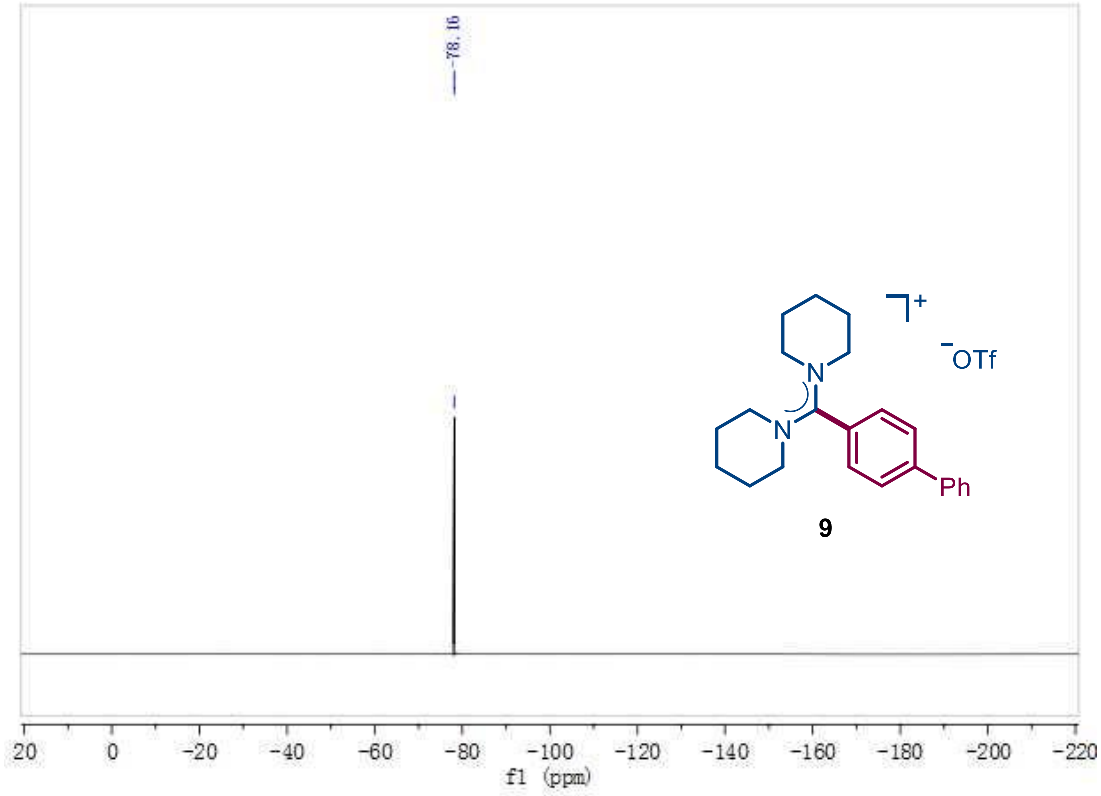

Supplementary Figure 32. ^{19}F NMR Spectrum of Compound **9**

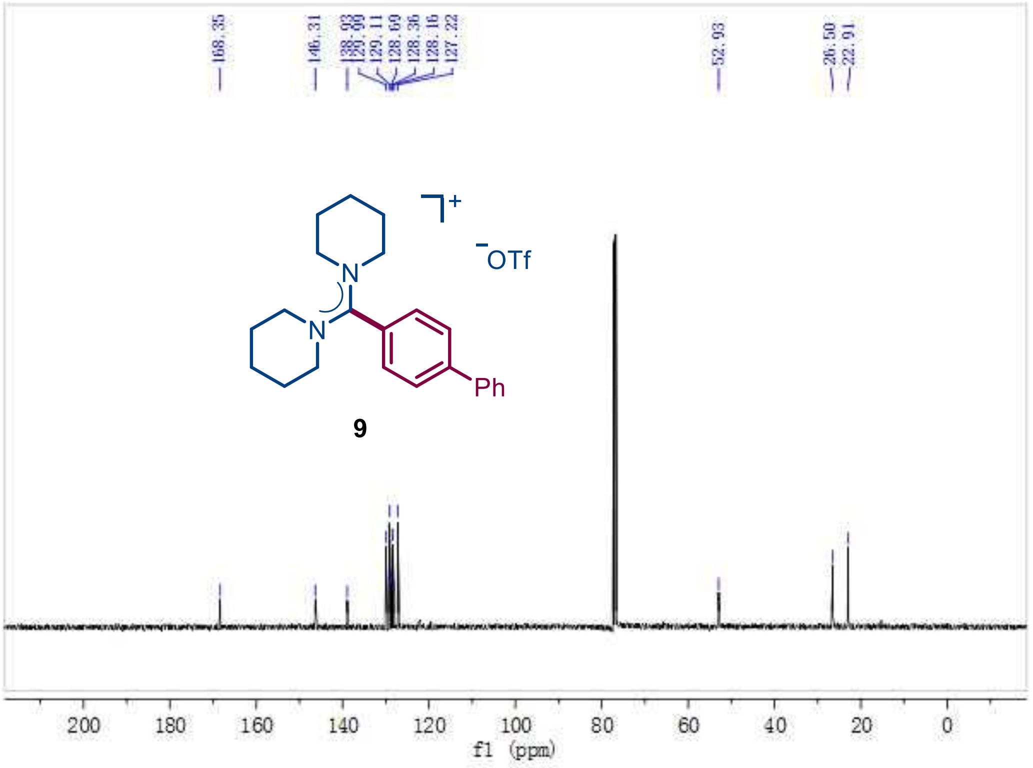

Supplementary Figure 33. ^{13}C NMR Spectrum of Compound **9**

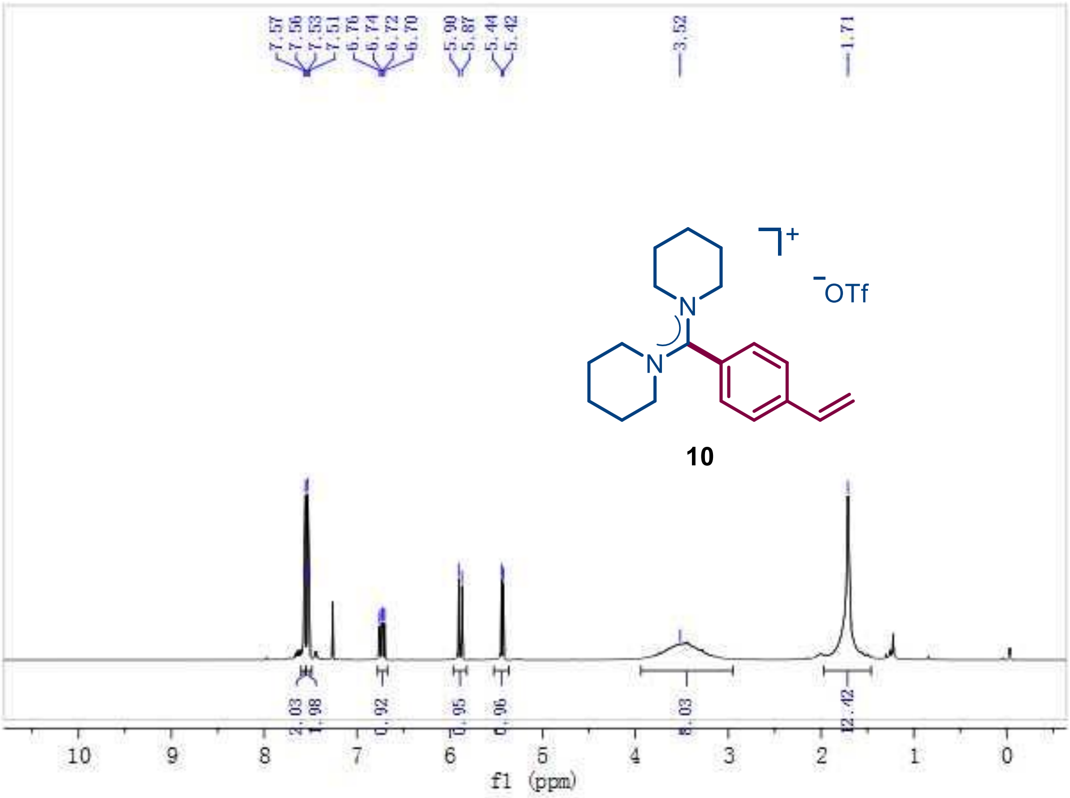

Supplementary Figure 34. ^{1}H NMR Spectrum of Compound **10**

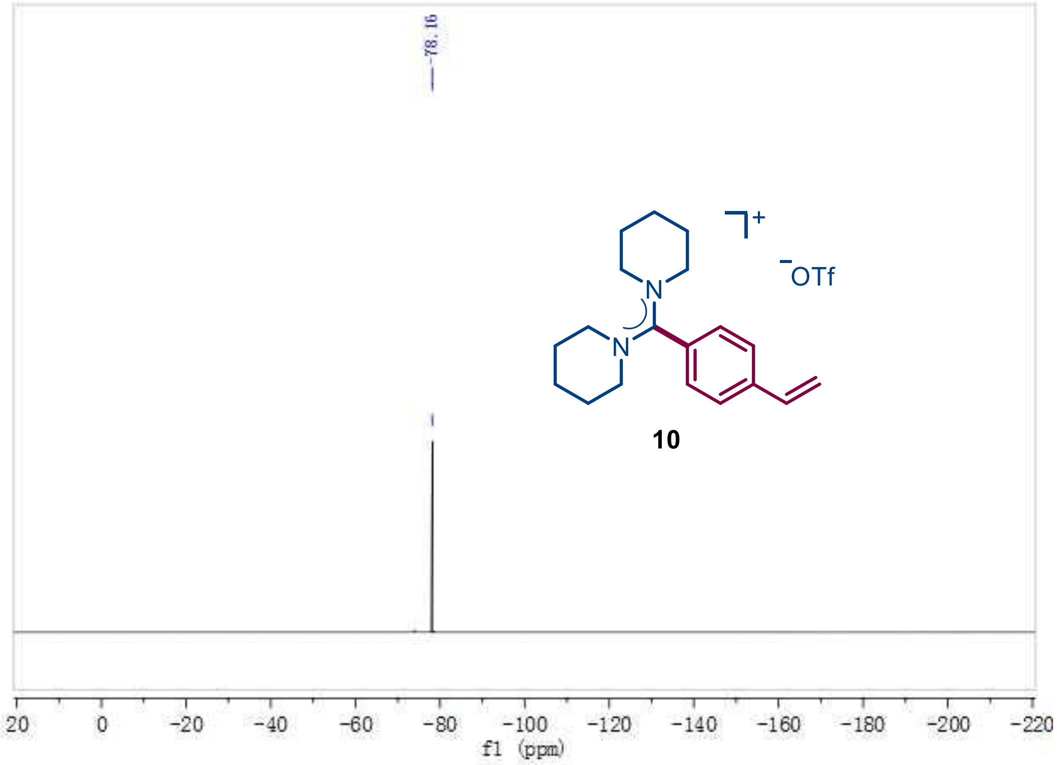

Supplementary Figure 35. ^{19}F NMR Spectrum of Compound **10**

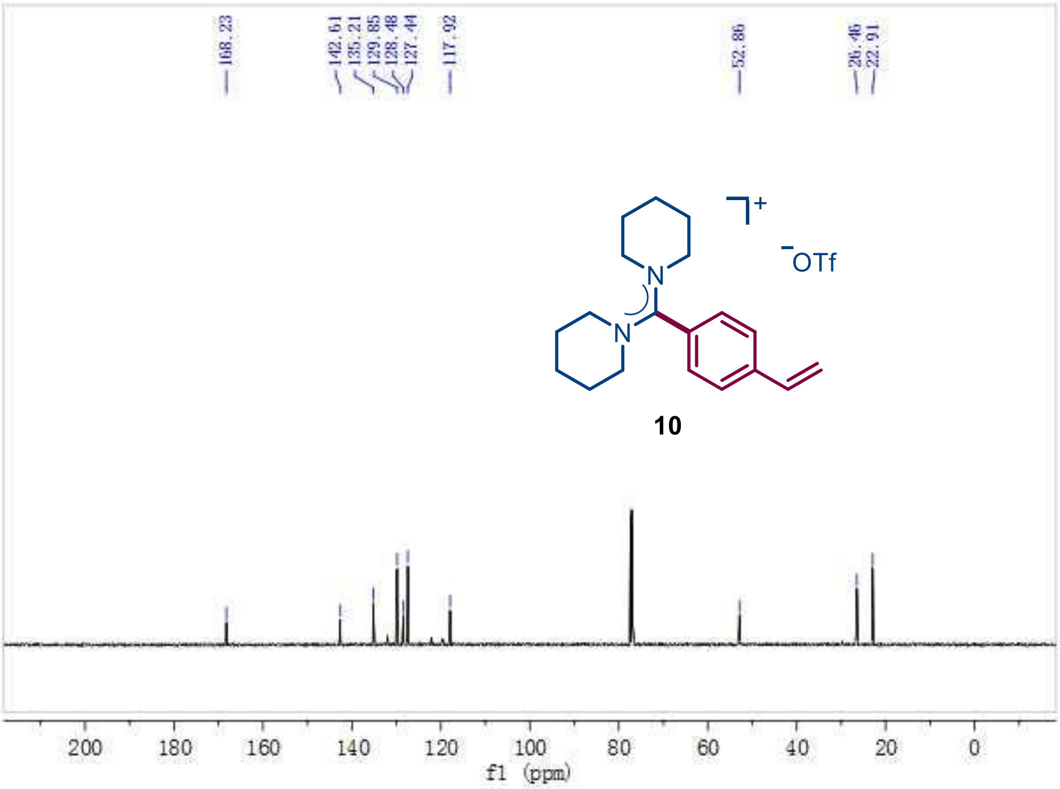

Supplementary Figure 36. ^{13}C NMR Spectrum of Compound **10**

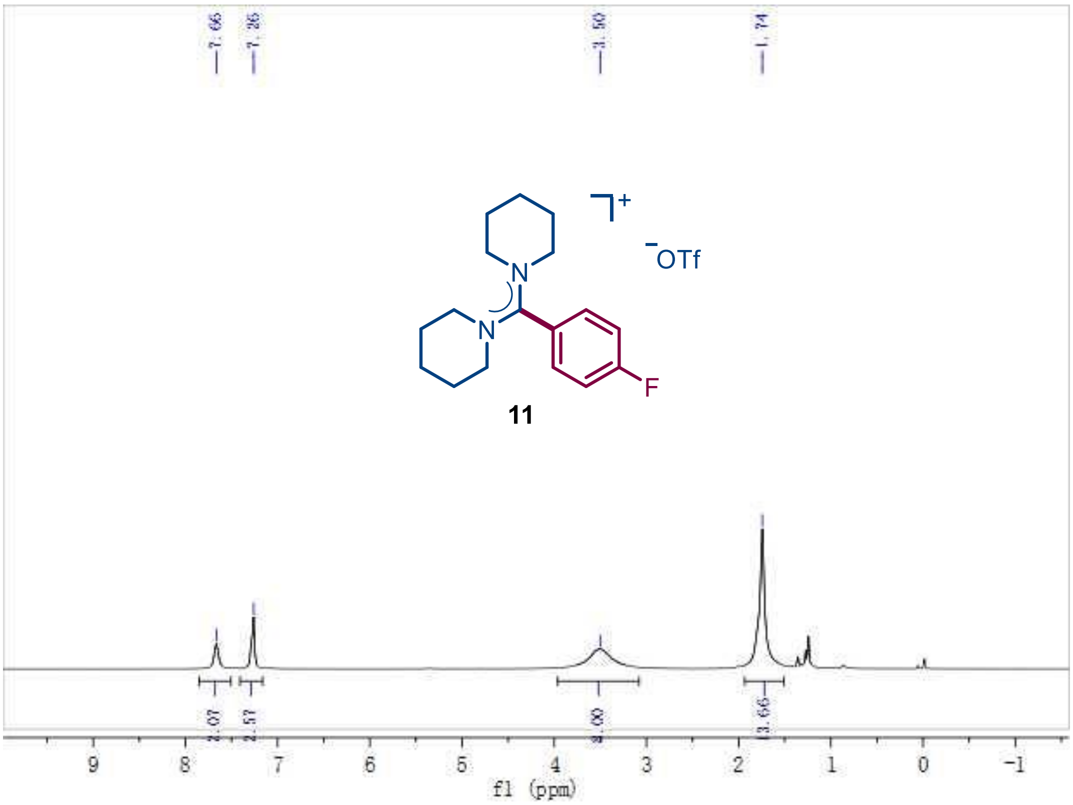

Supplementary Figure 37. ^{1}H NMR Spectrum of Compound **11**

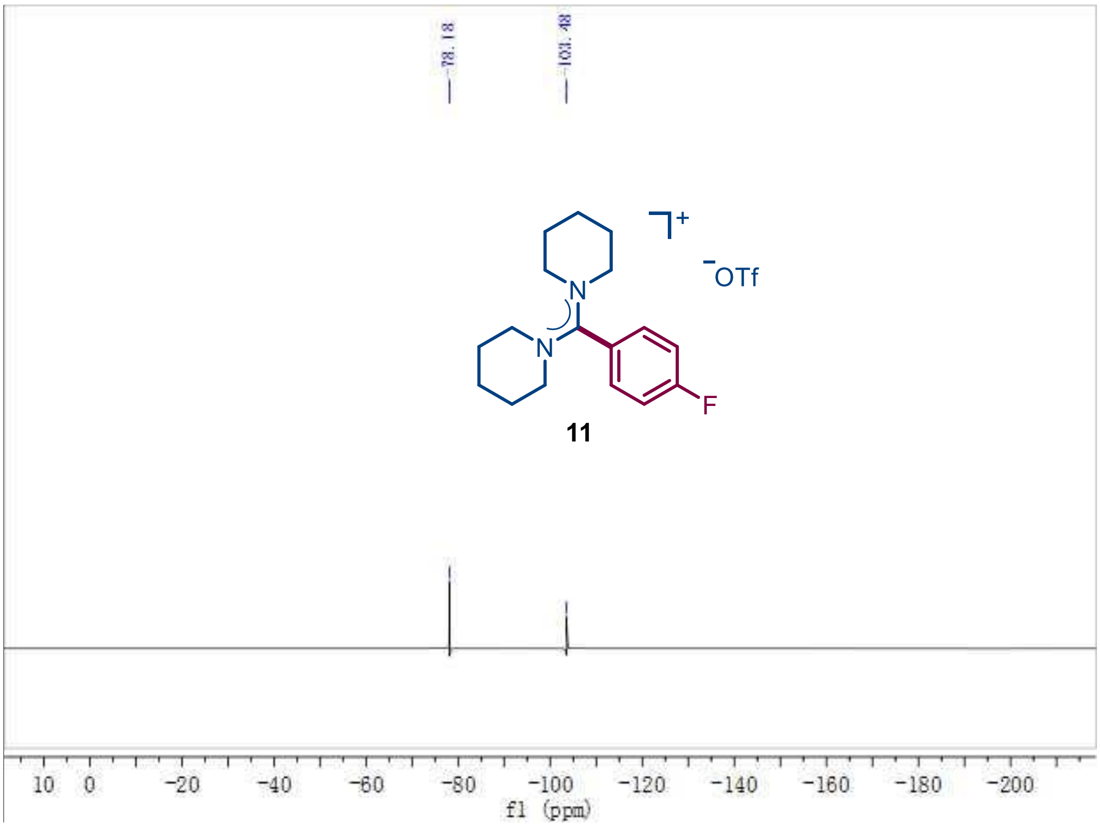

Supplementary Figure 38. ^{19}F NMR Spectrum of Compound **11**

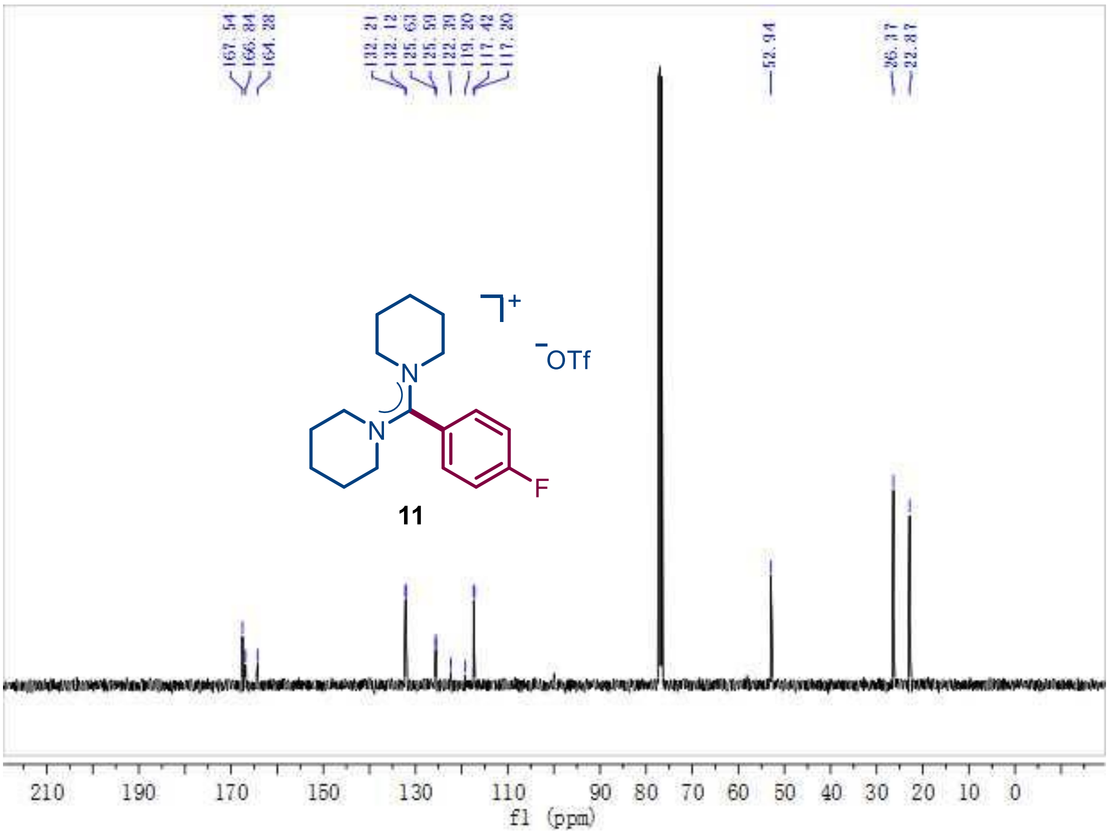

Supplementary Figure 39. ^{13}C NMR Spectrum of Compound **11**

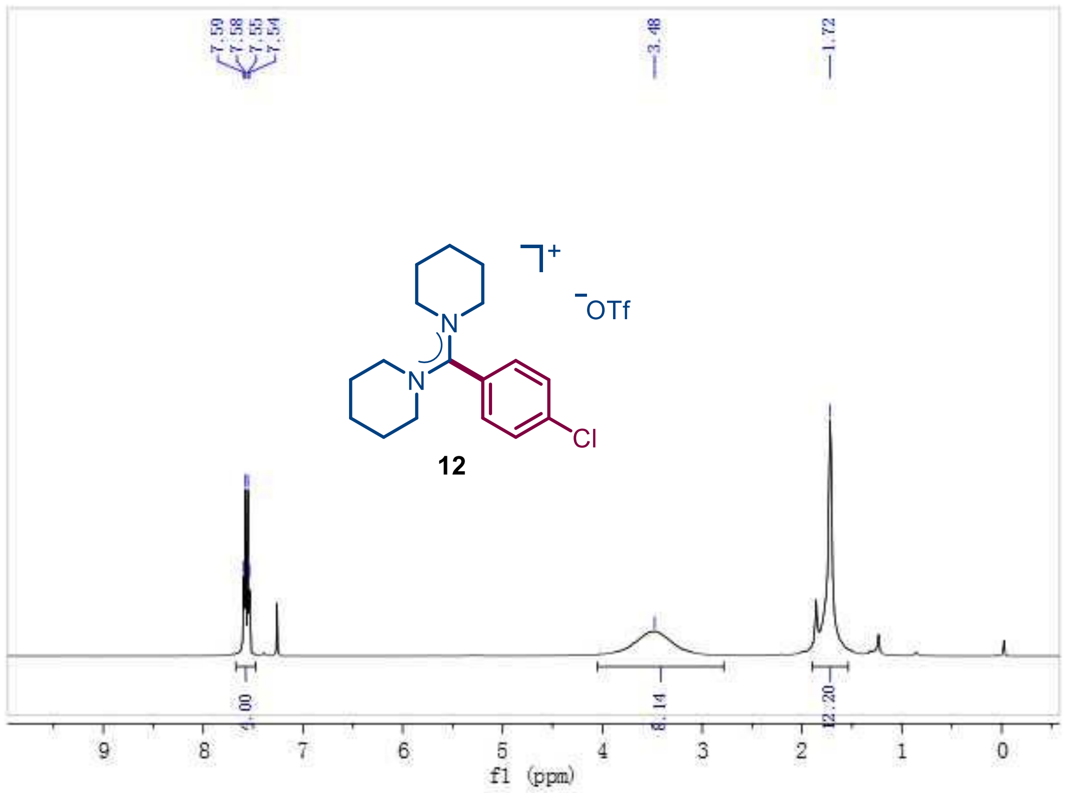

Supplementary Figure 40. ^{1}H NMR Spectrum of Compound **12**

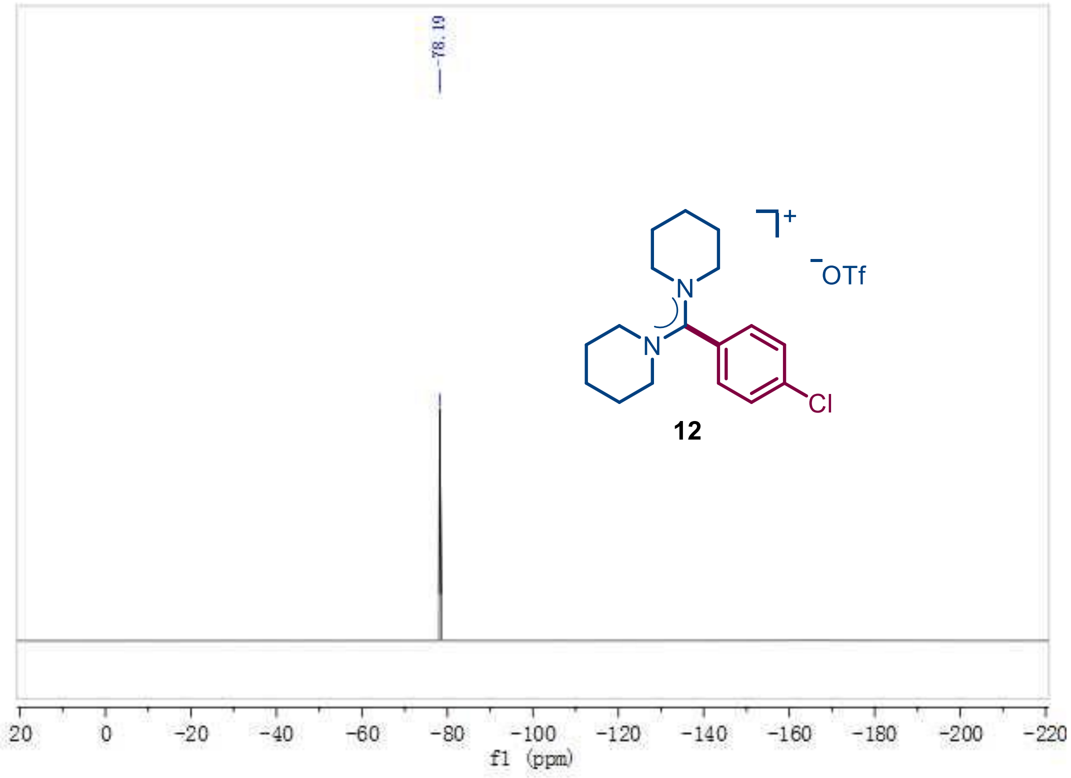

Supplementary Figure 41. ^{19}F NMR Spectrum of Compound **12**

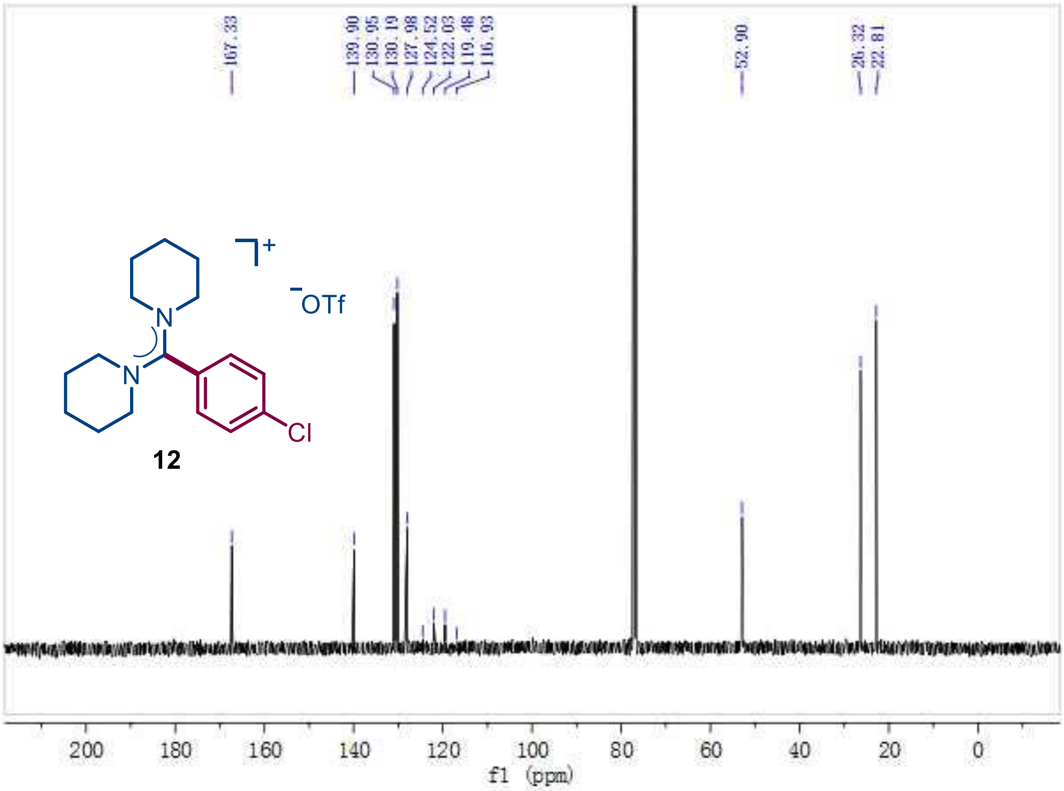

Supplementary Figure 42. ^{13}C NMR Spectrum of Compound **12**

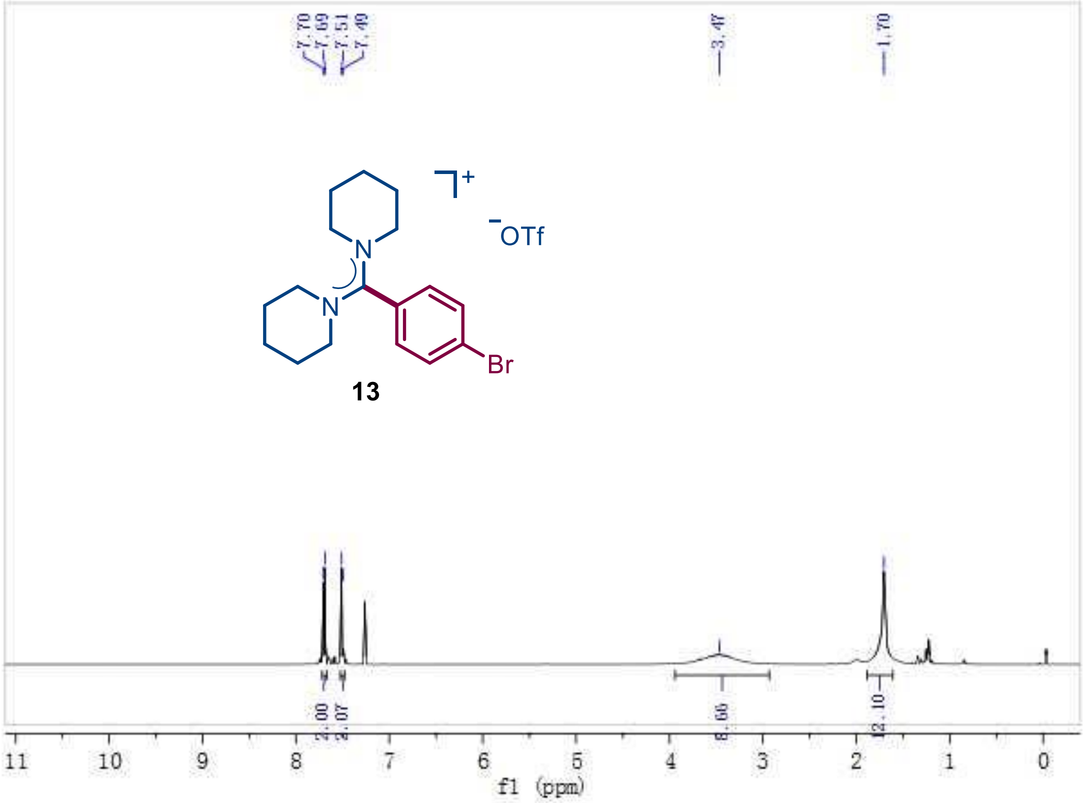

Supplementary Figure 43. ^{1}H NMR Spectrum of Compound **13**

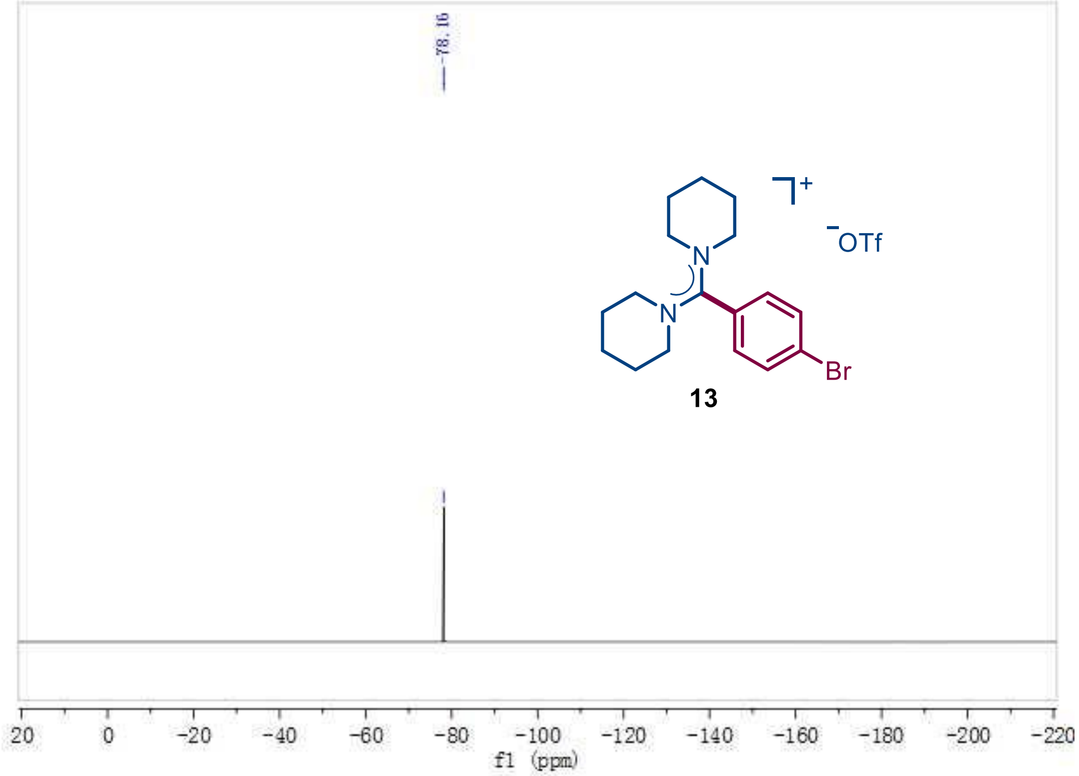

Supplementary Figure 44. ^{19}F NMR Spectrum of Compound **13**

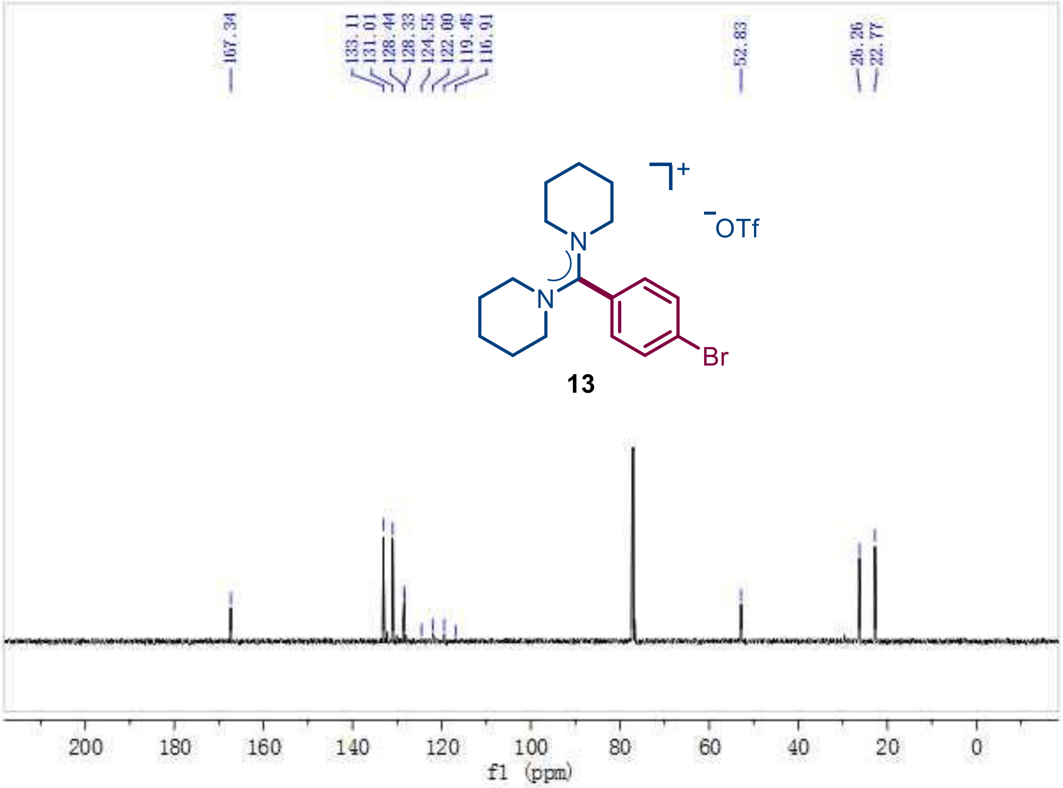

Supplementary Figure 45. ^{13}C NMR Spectrum of Compound **13**

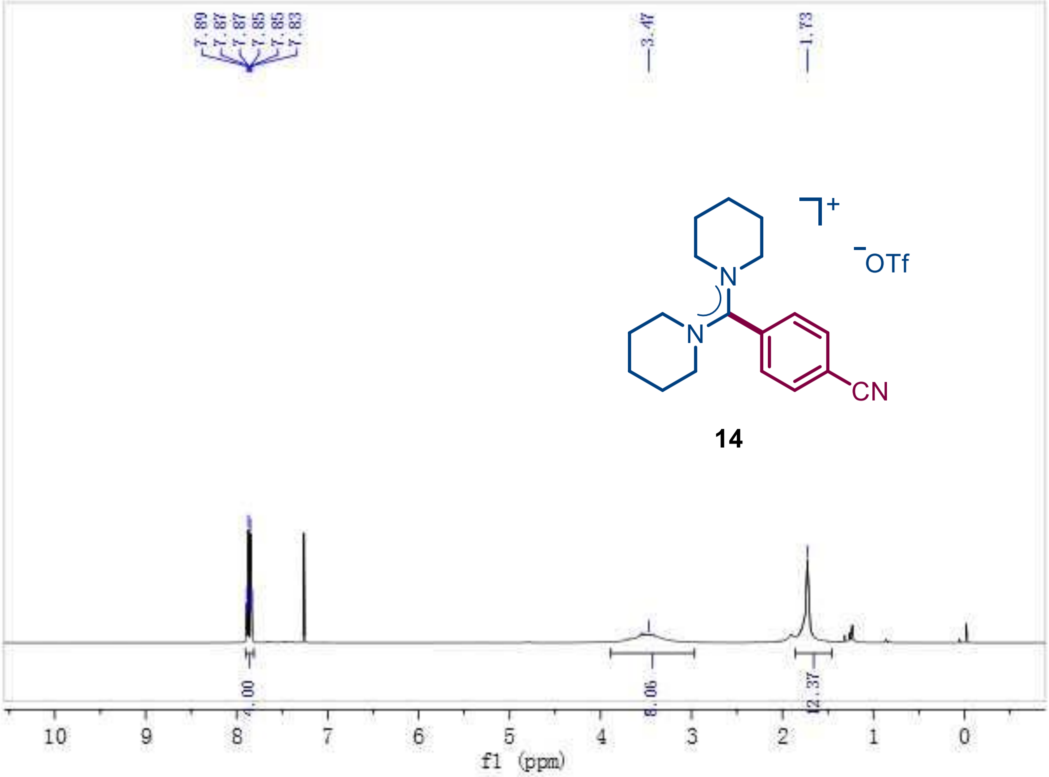

Supplementary Figure 46. ^{1}H NMR Spectrum of Compound **14**

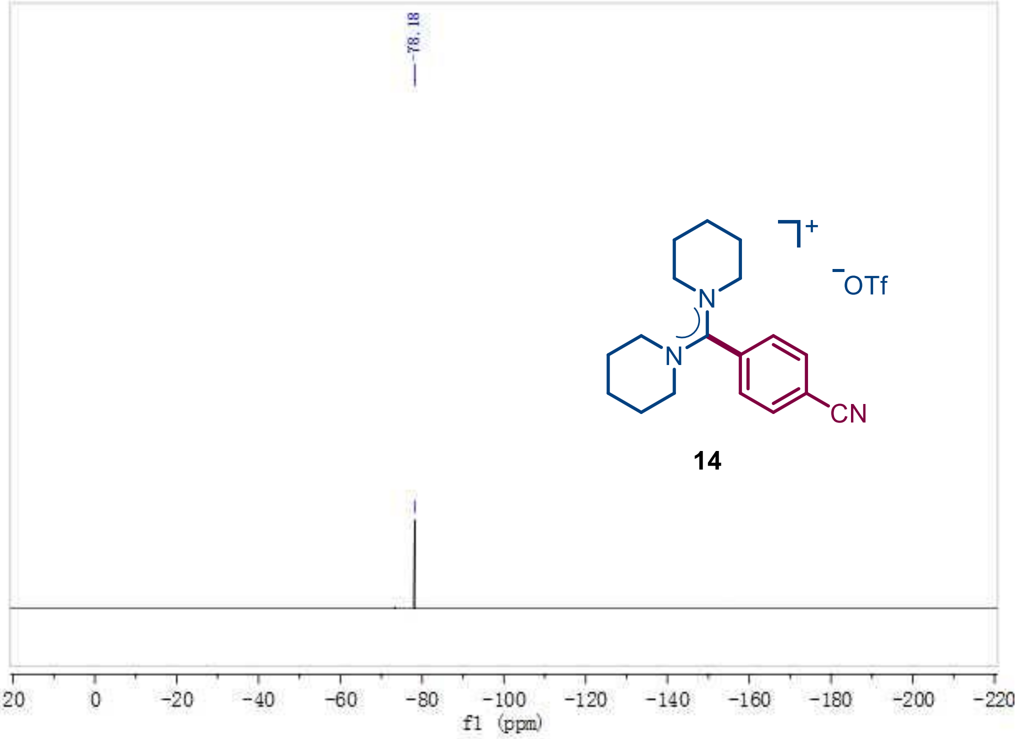

Supplementary Figure 47. ^{19}F NMR Spectrum of Compound **14**

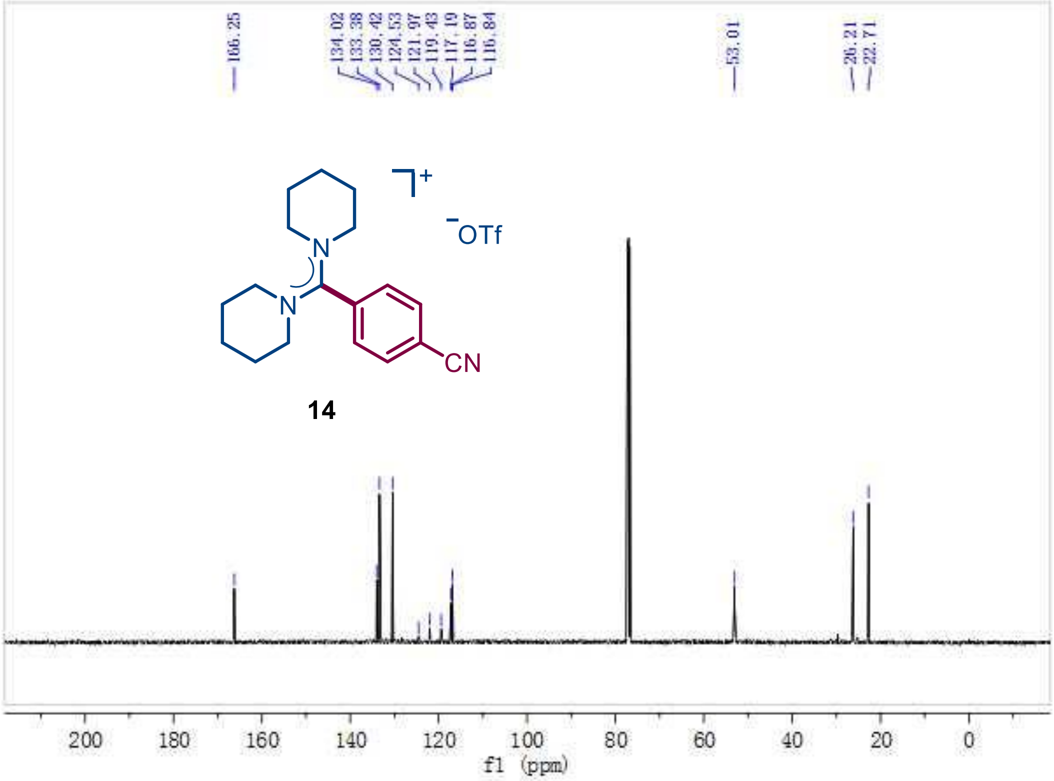

Supplementary Figure 48. ^{13}C NMR Spectrum of Compound **14**

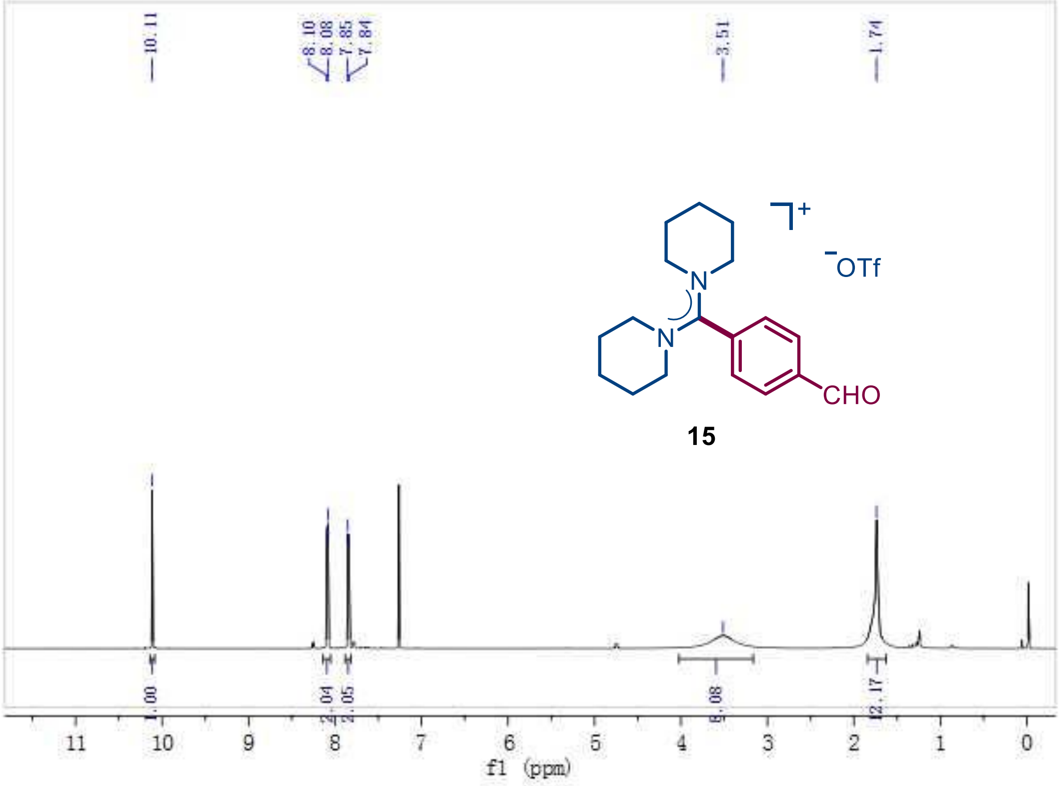

Supplementary Figure 49. ^{1}H NMR Spectrum of Compound **15**

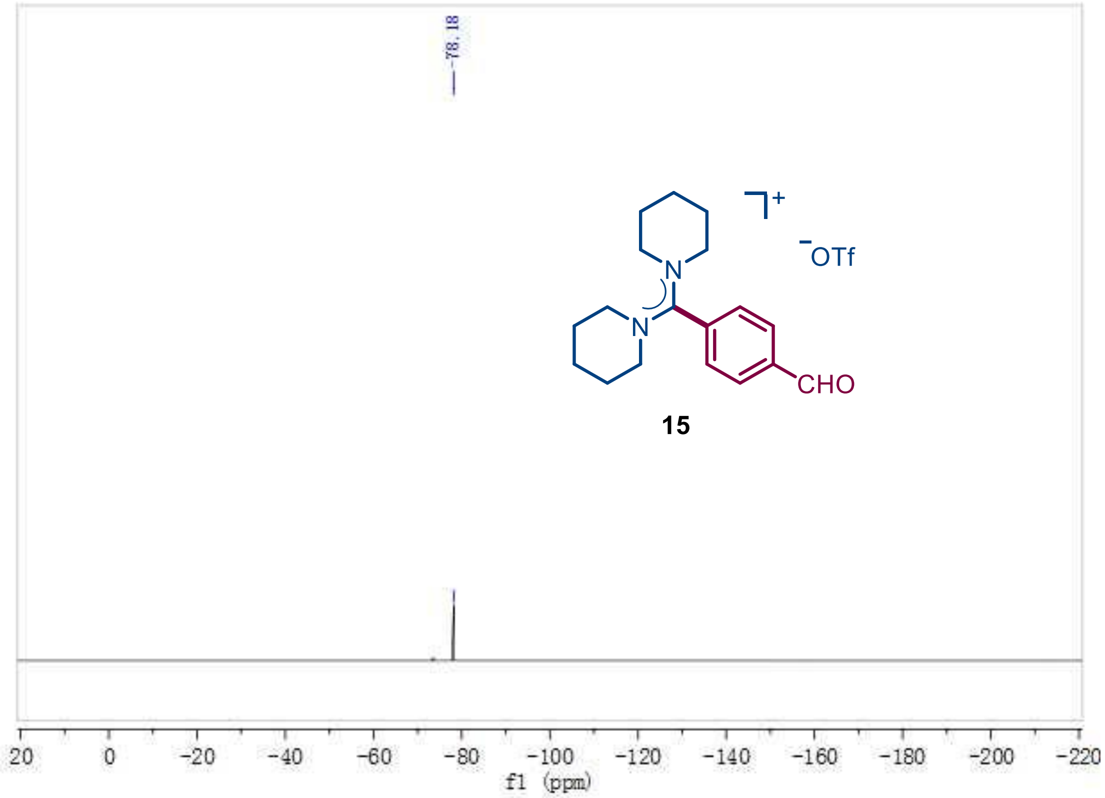

Supplementary Figure 50. ^{19}F NMR Spectrum of Compound **15**

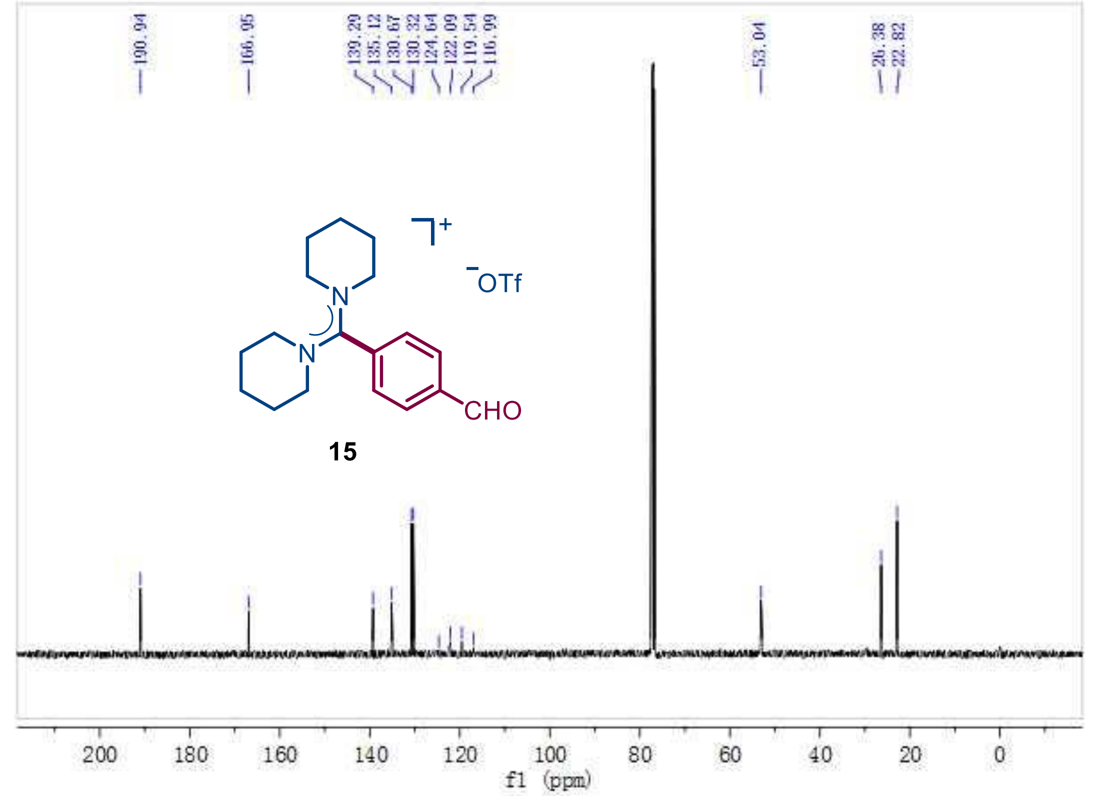

Supplementary Figure 51. ^{13}C NMR Spectrum of Compound **15**

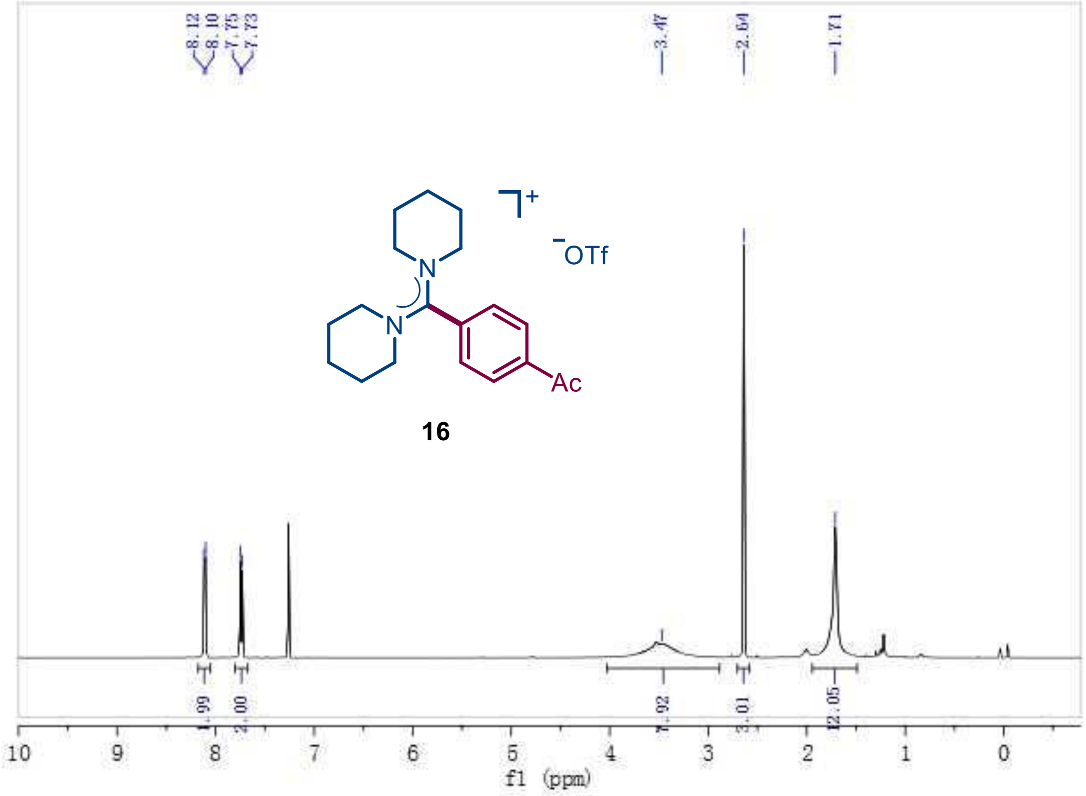

Supplementary Figure 52. ^{1}H NMR Spectrum of Compound **16**

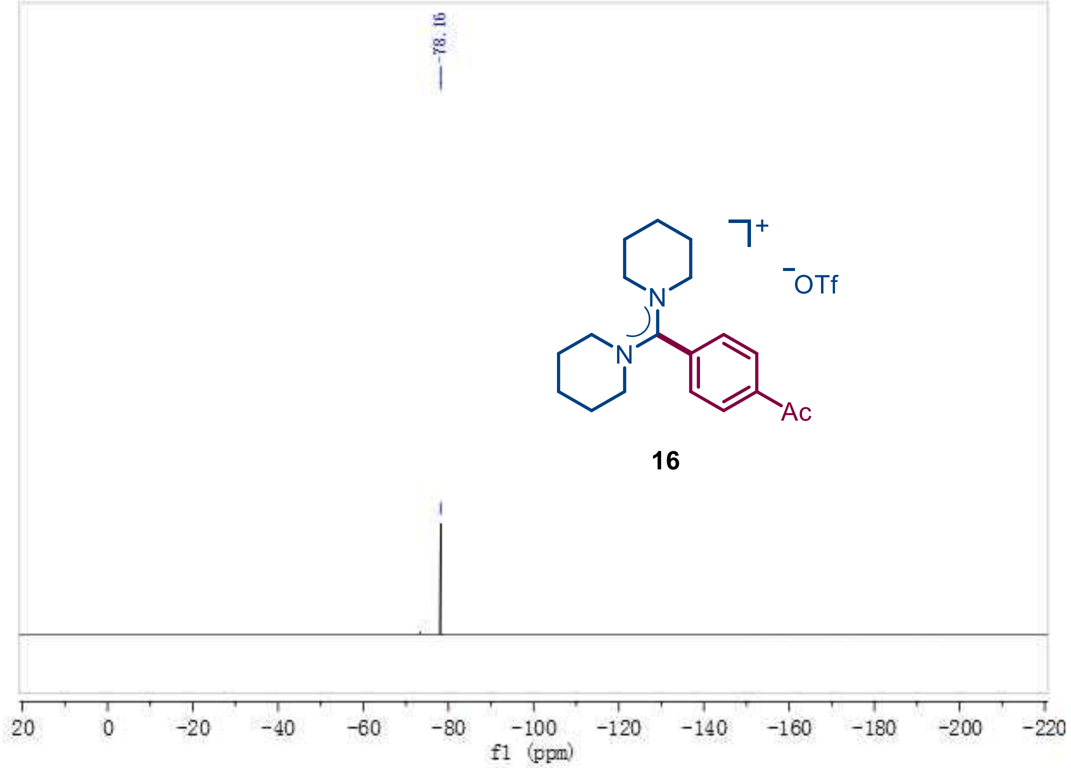

Supplementary Figure 53. ^{19}F NMR Spectrum of Compound **16**

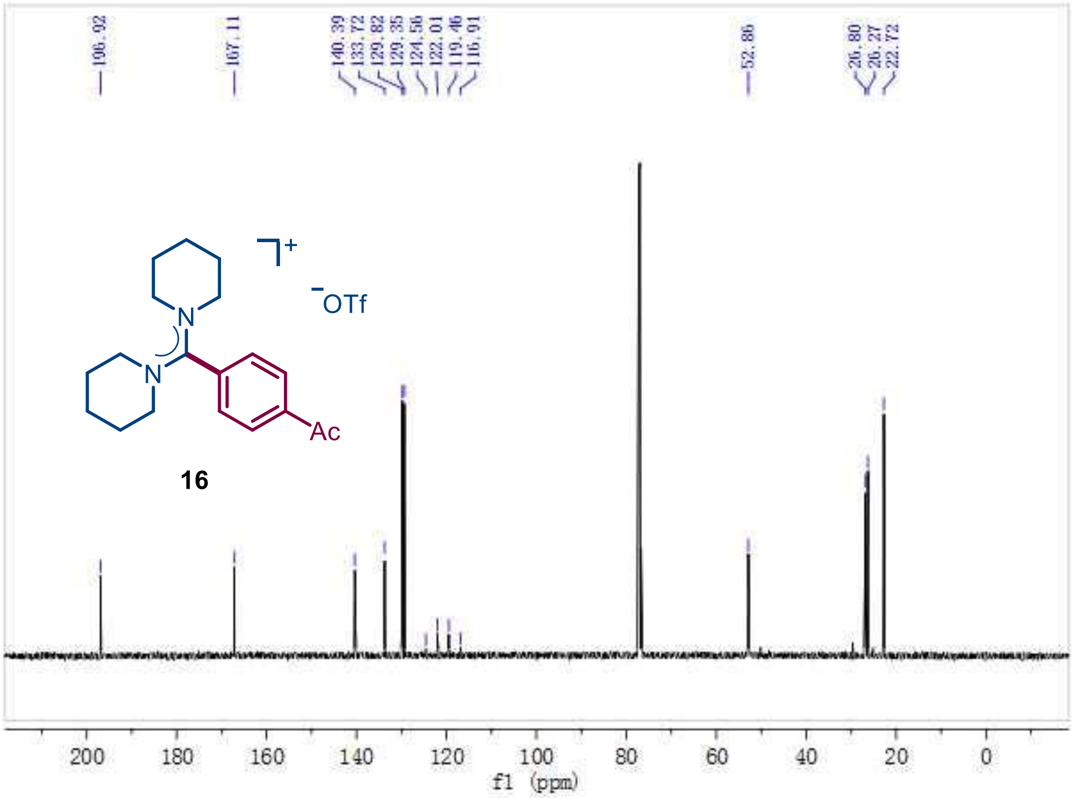

Supplementary Figure 54. ^{13}C NMR Spectrum of Compound **16**

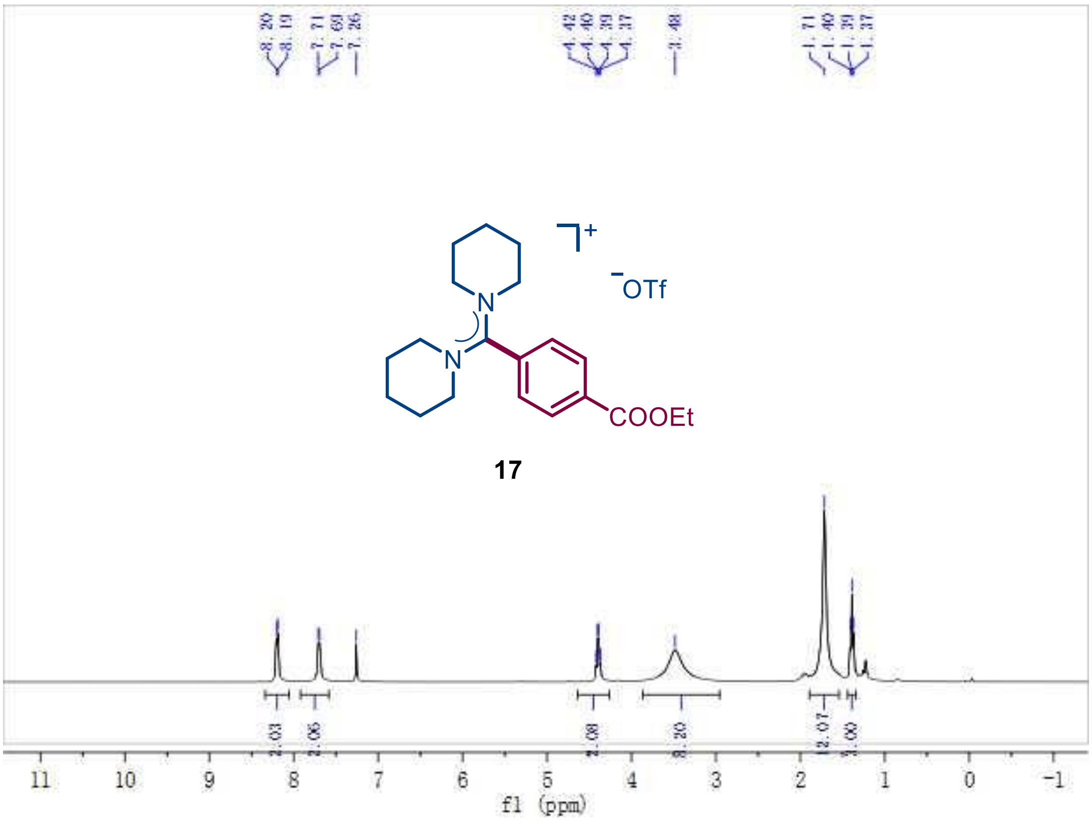

Supplementary Figure 55. ^{1}H NMR Spectrum of Compound **17**

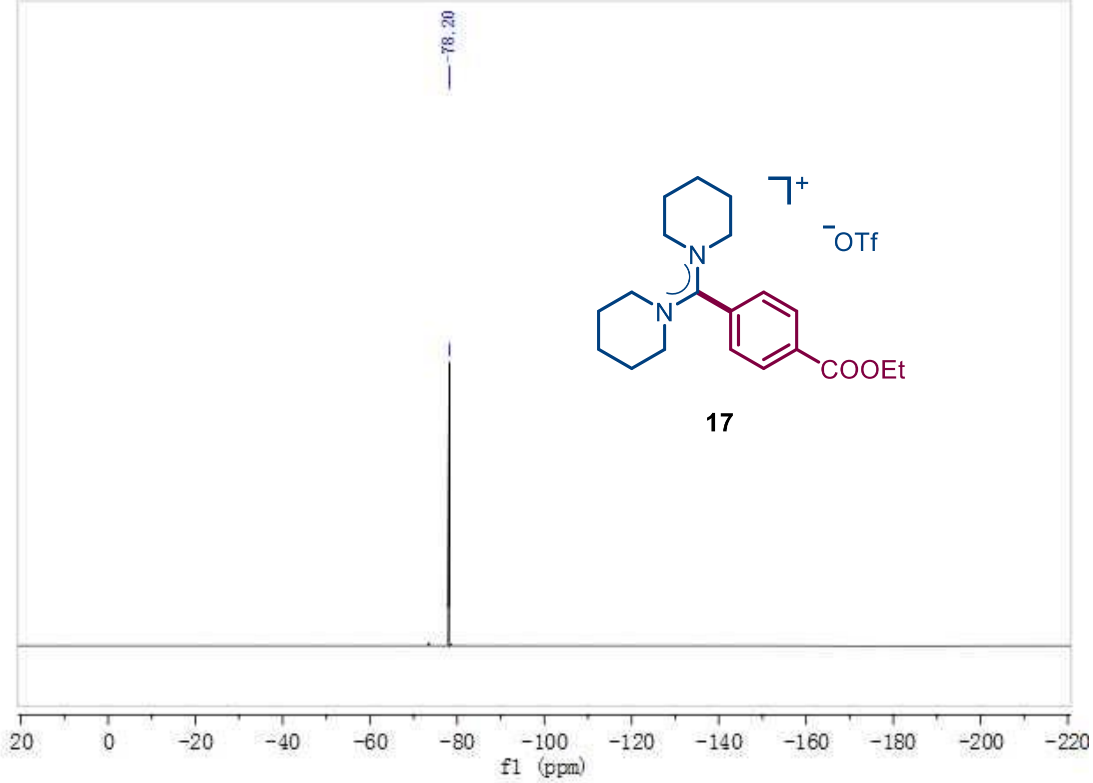

Supplementary Figure 56. ^{19}F NMR Spectrum of Compound **17**

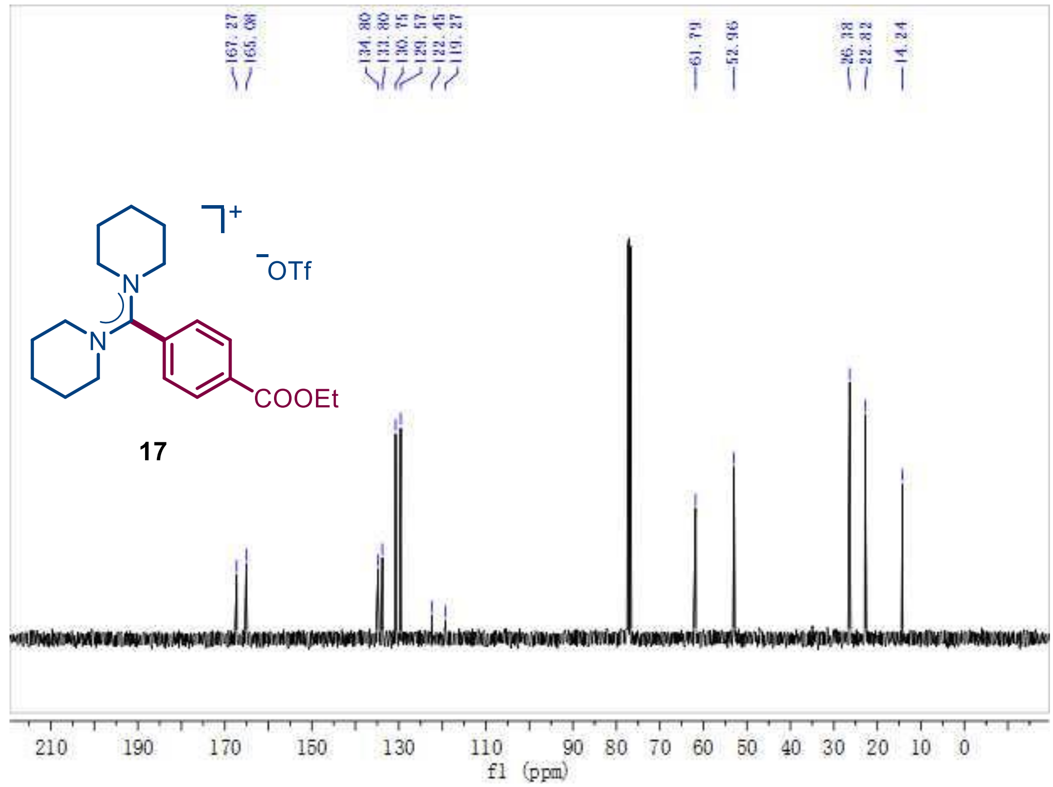

Supplementary Figure 57. ^{13}C NMR Spectrum of Compound **17**

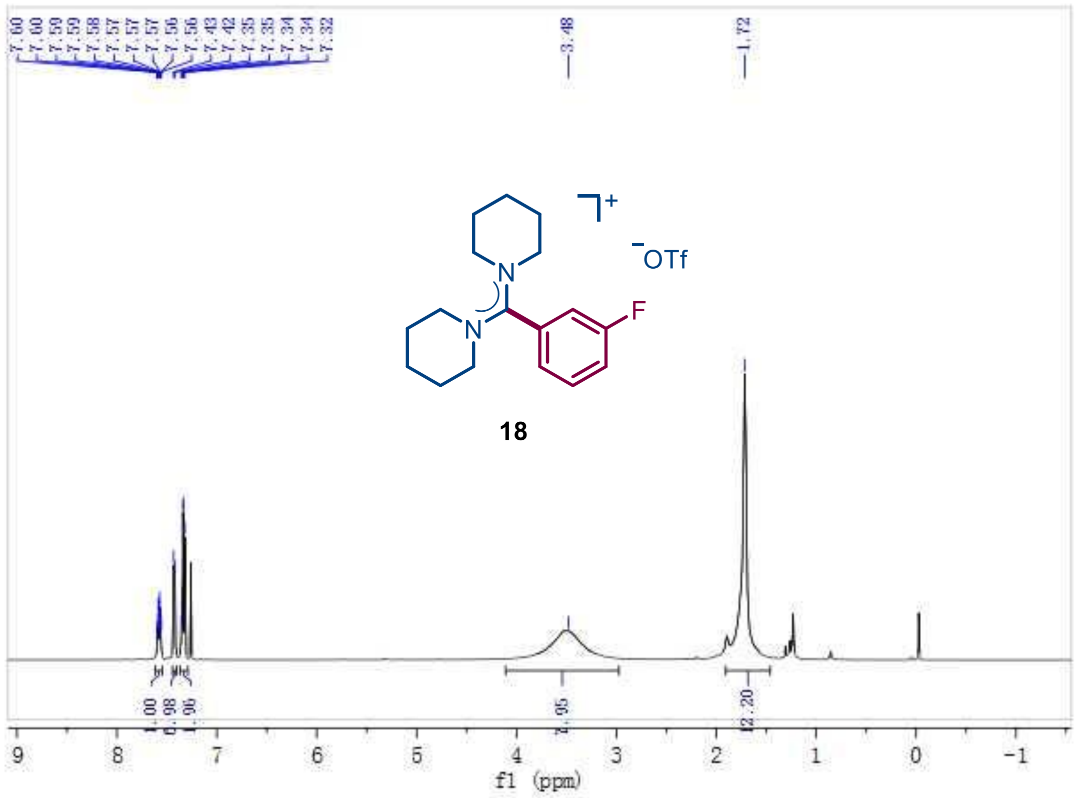

Supplementary Figure 58. ^{1}H NMR Spectrum of Compound **18**

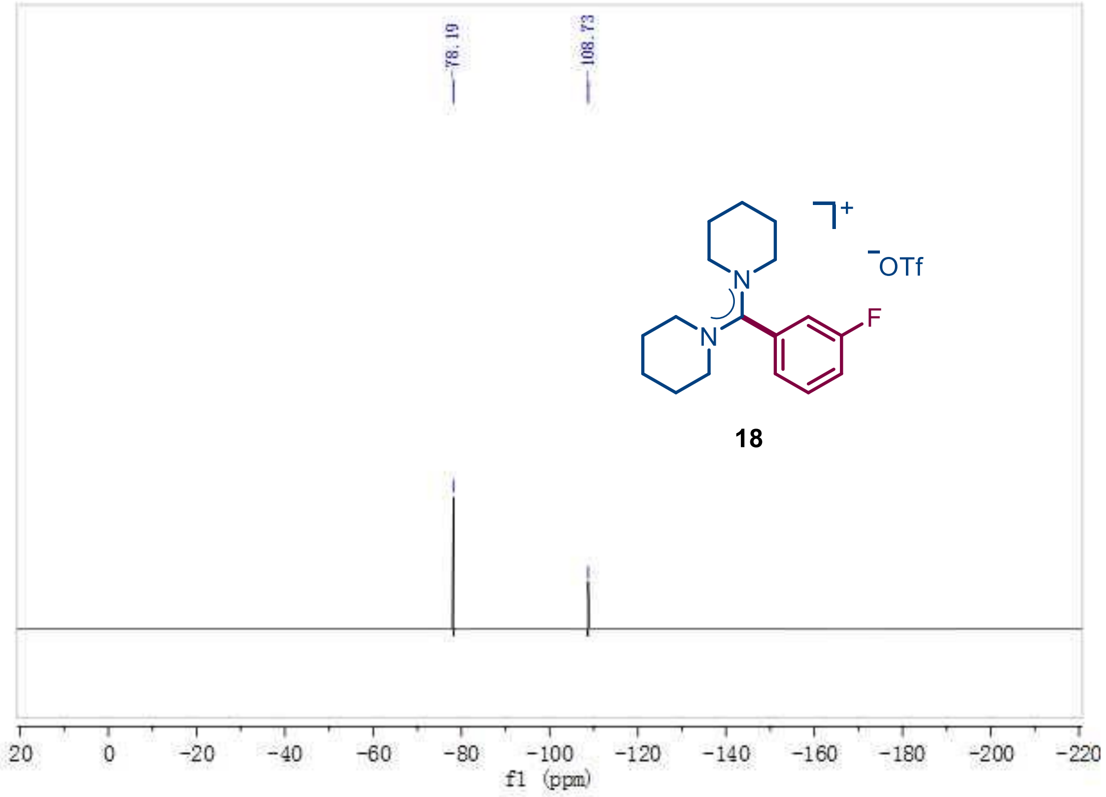

Supplementary Figure 59. ^{19}F NMR Spectrum of Compound **18**

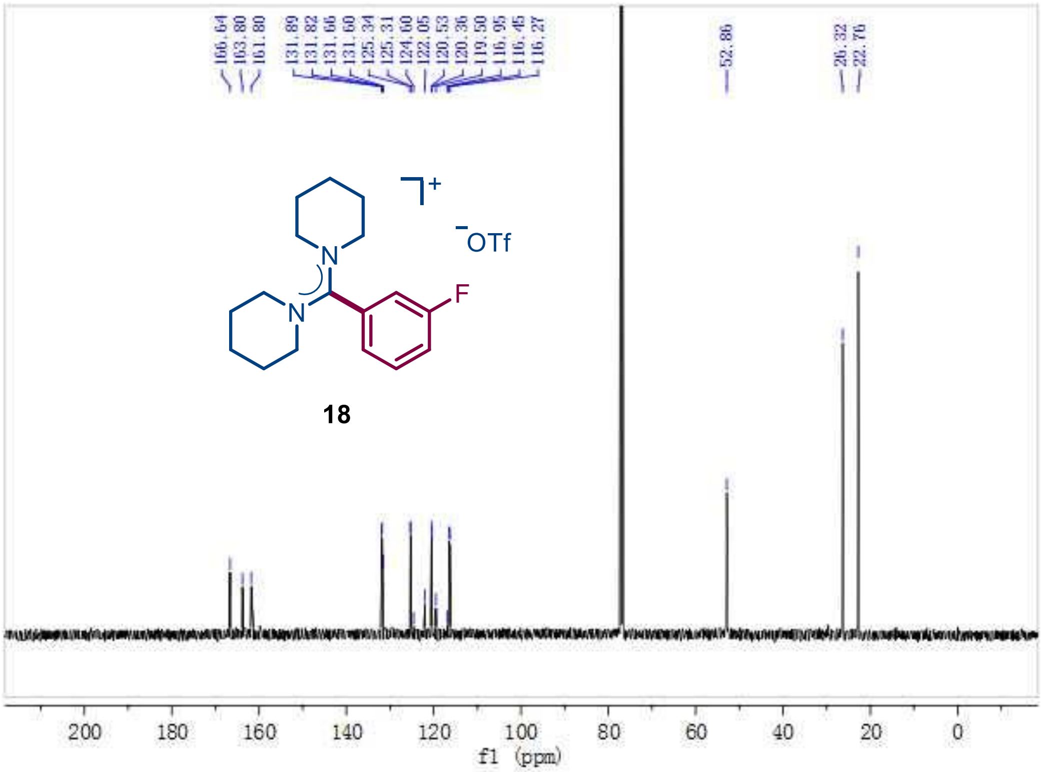

Supplementary Figure 60. ^{13}C NMR Spectrum of Compound **18**

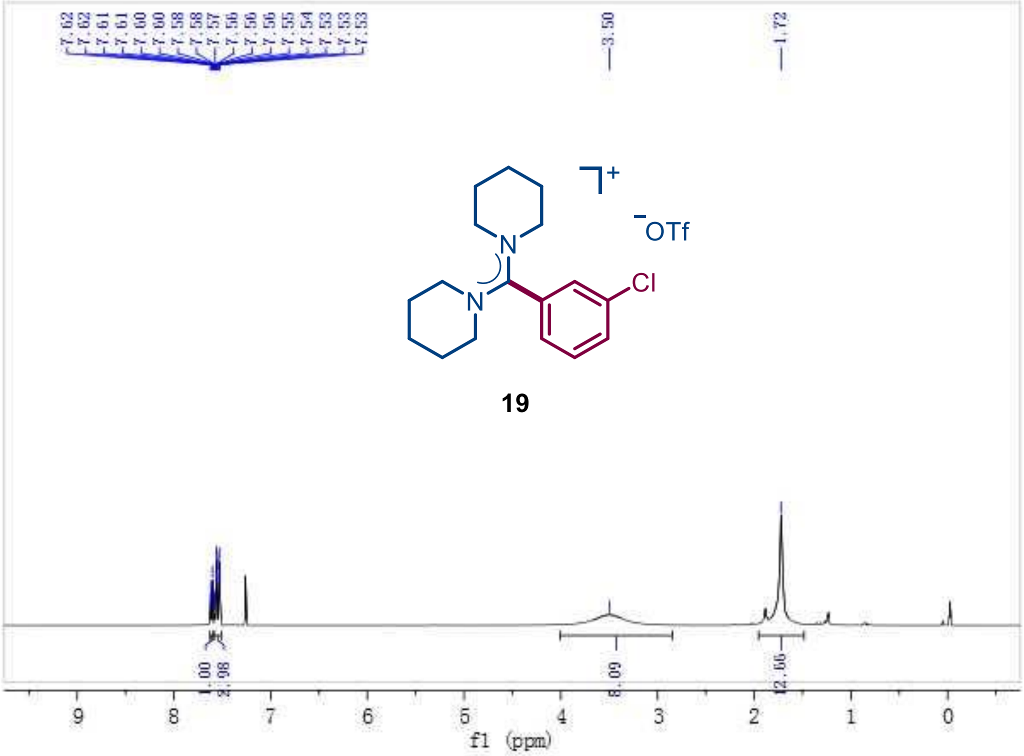

Supplementary Figure 61. ^{1}H NMR Spectrum of Compound **19**

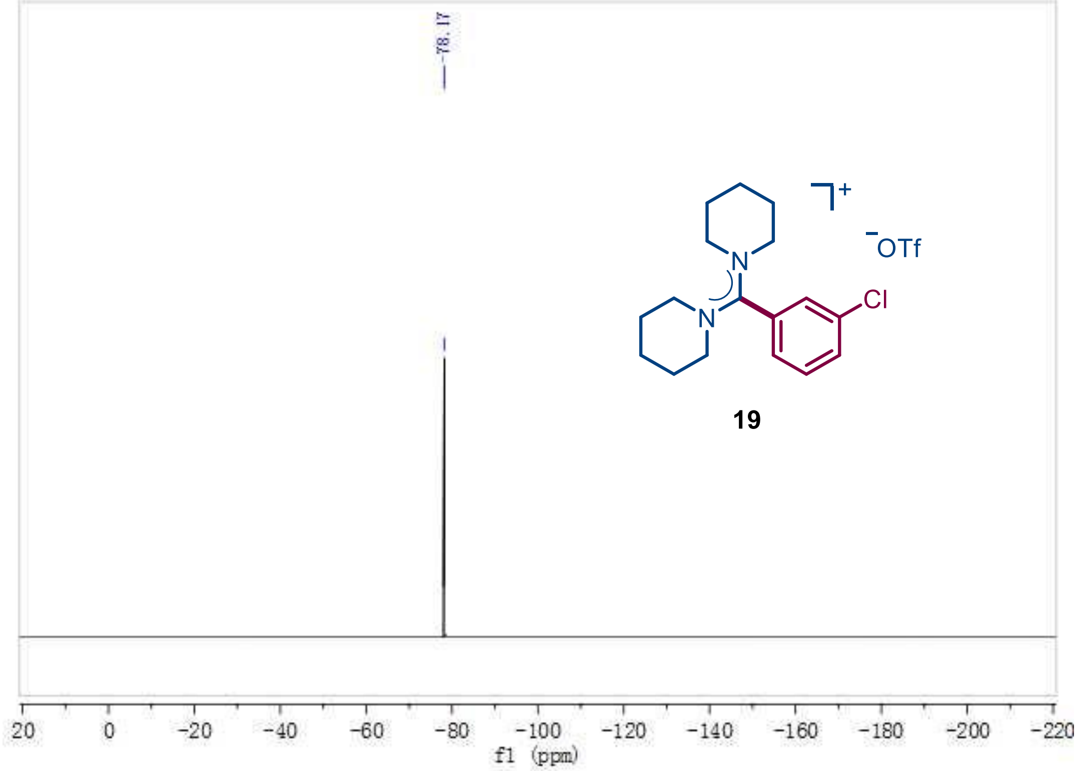

Supplementary Figure 62. ^{19}F NMR Spectrum of Compound **19**

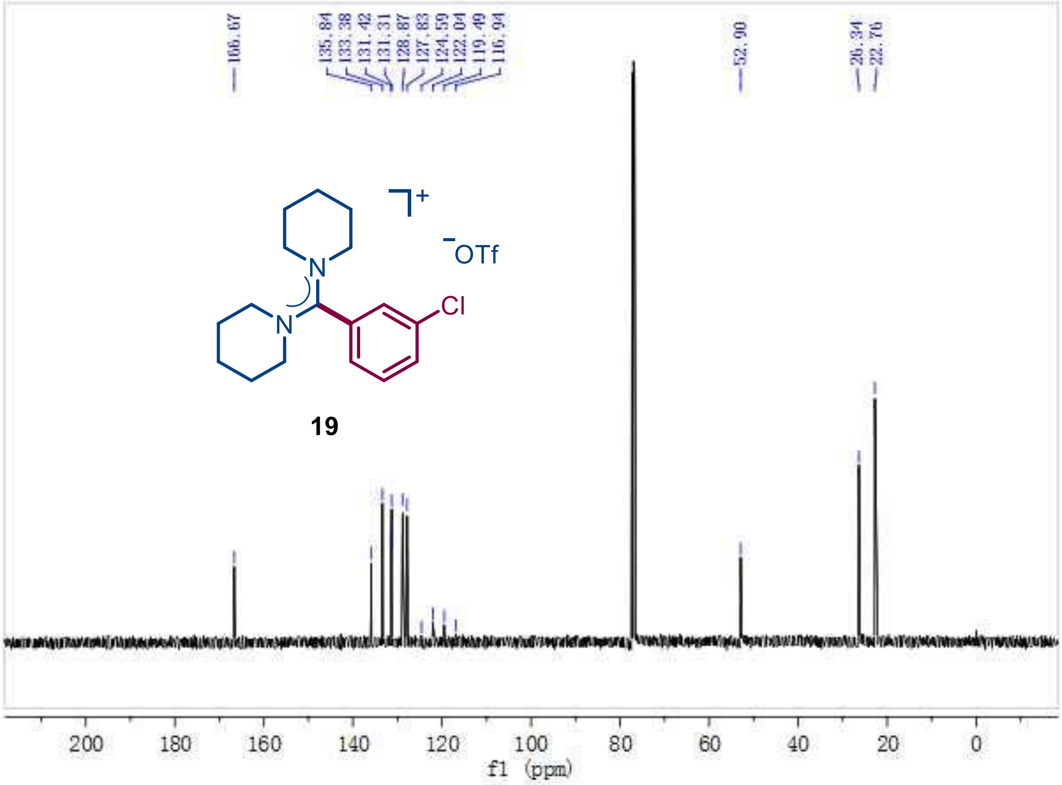

Supplementary Figure 63. ^{13}C NMR Spectrum of Compound **19**

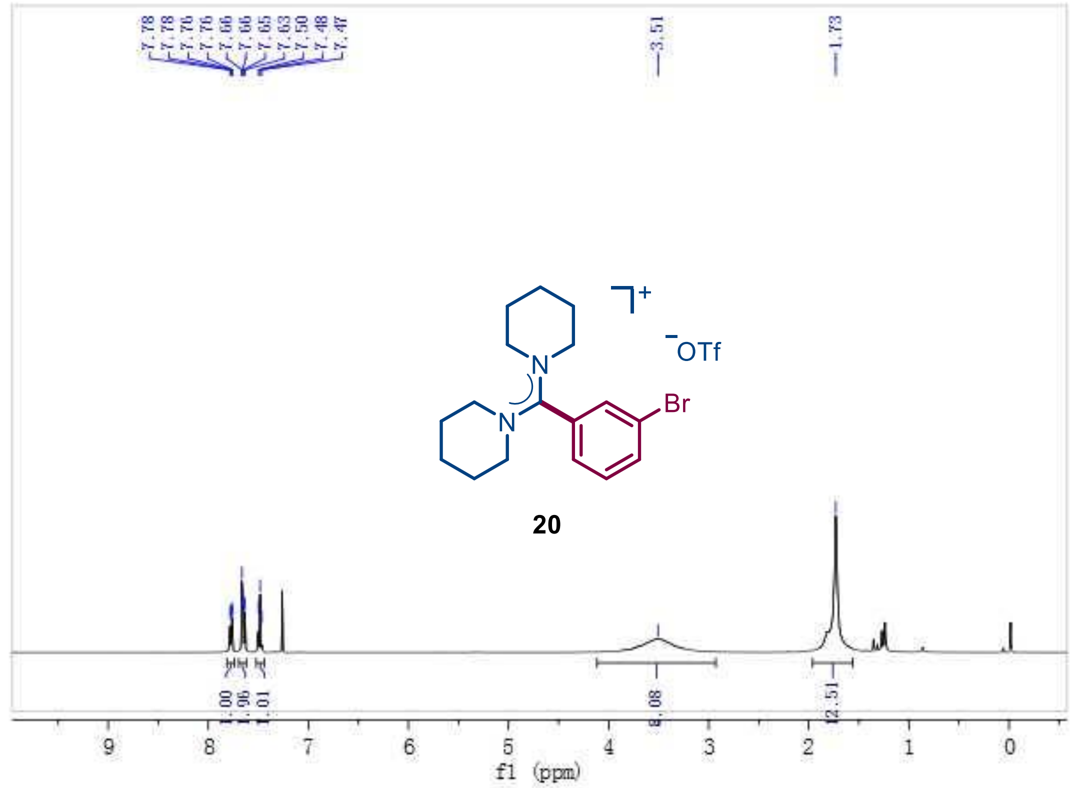

Supplementary Figure 64. ^{1}H NMR Spectrum of Compound **20**

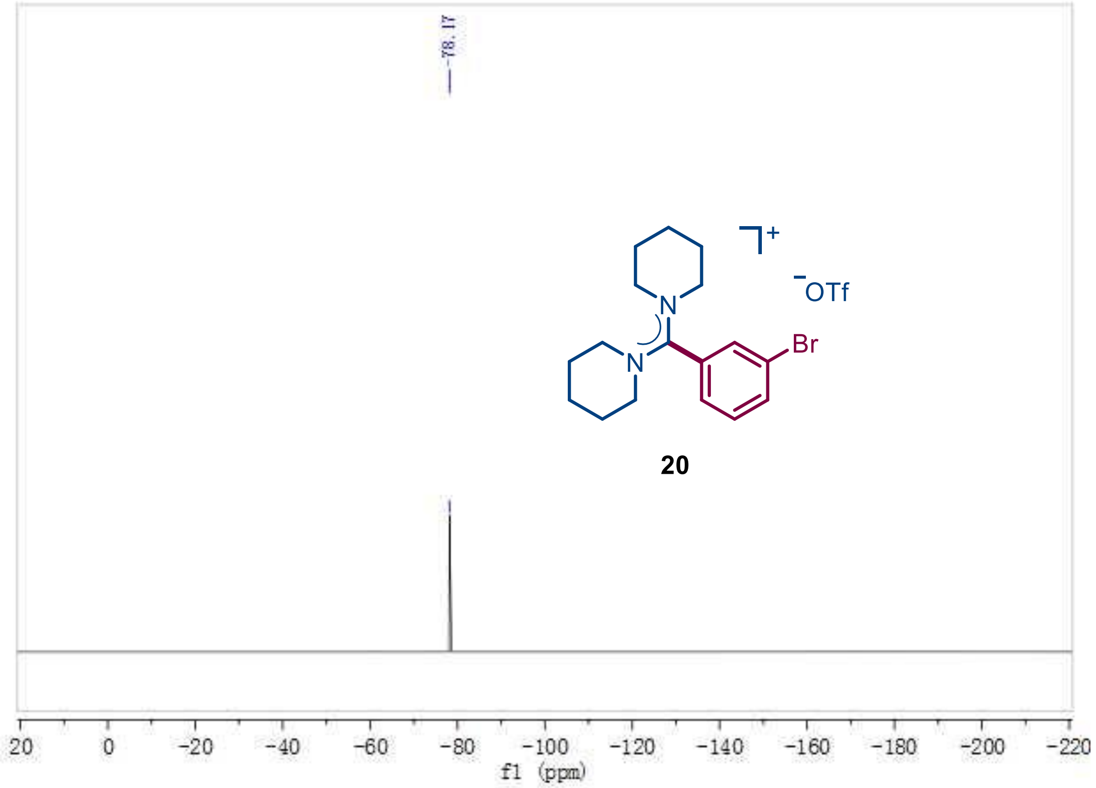

Supplementary Figure 65. ^{19}F NMR Spectrum of Compound **20**

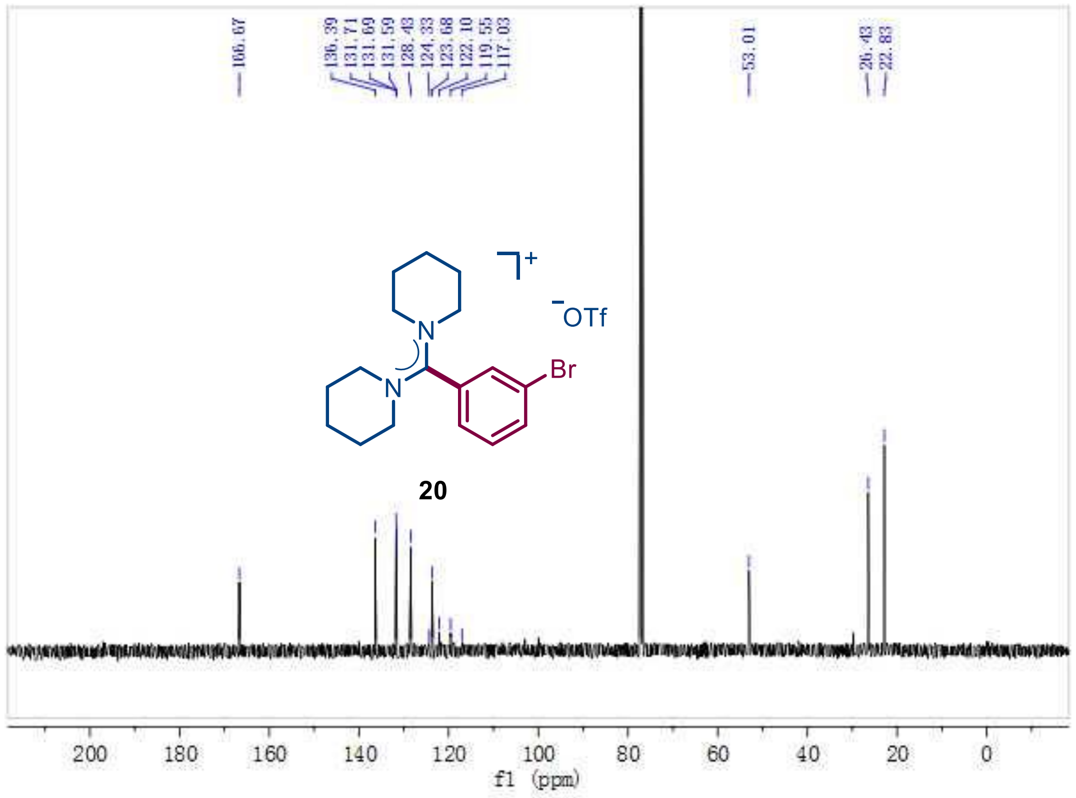

Supplementary Figure 66. ^{13}C NMR Spectrum of Compound **20**

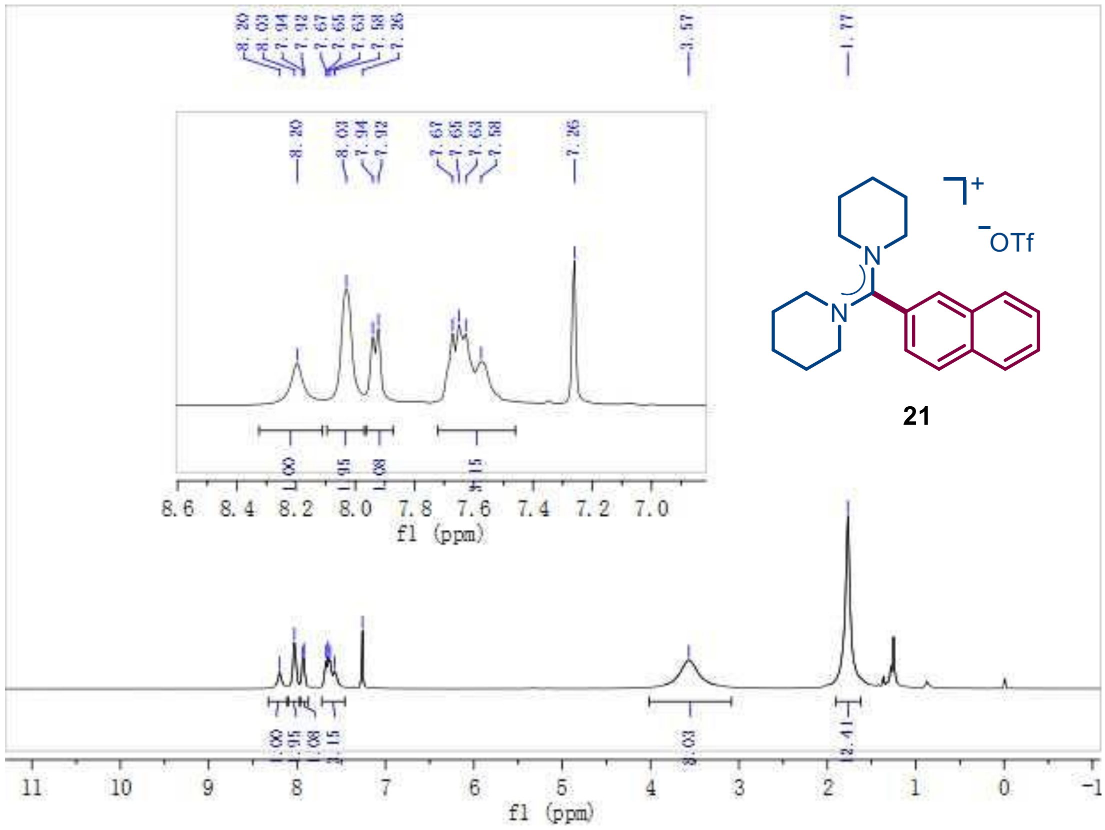

Supplementary Figure 67. ^{1}H NMR Spectrum of Compound **21**

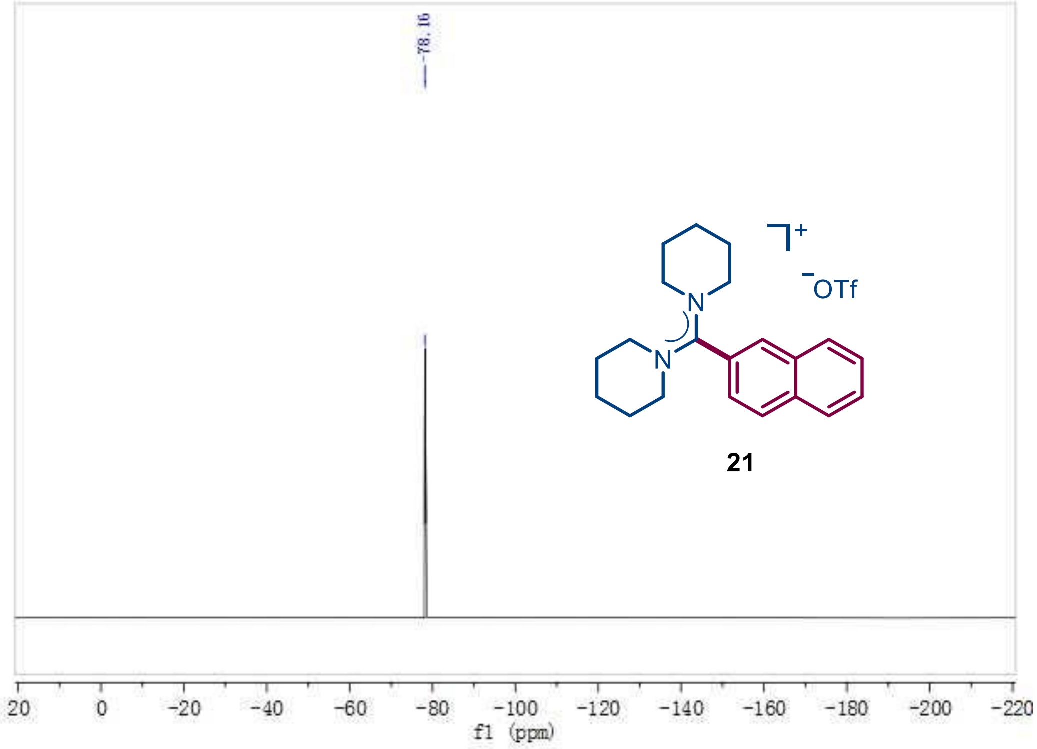

Supplementary Figure 68. ^{19}F NMR Spectrum of Compound **21**

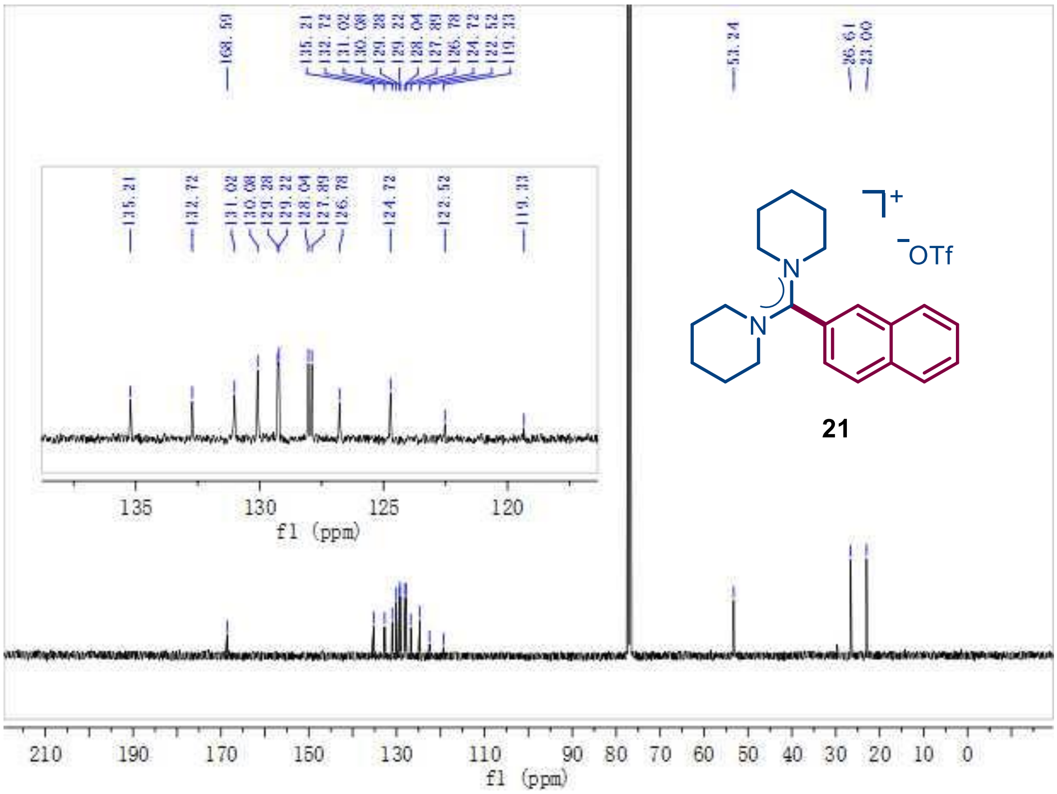

Supplementary Figure 69. ^{13}C NMR Spectrum of Compound **21**

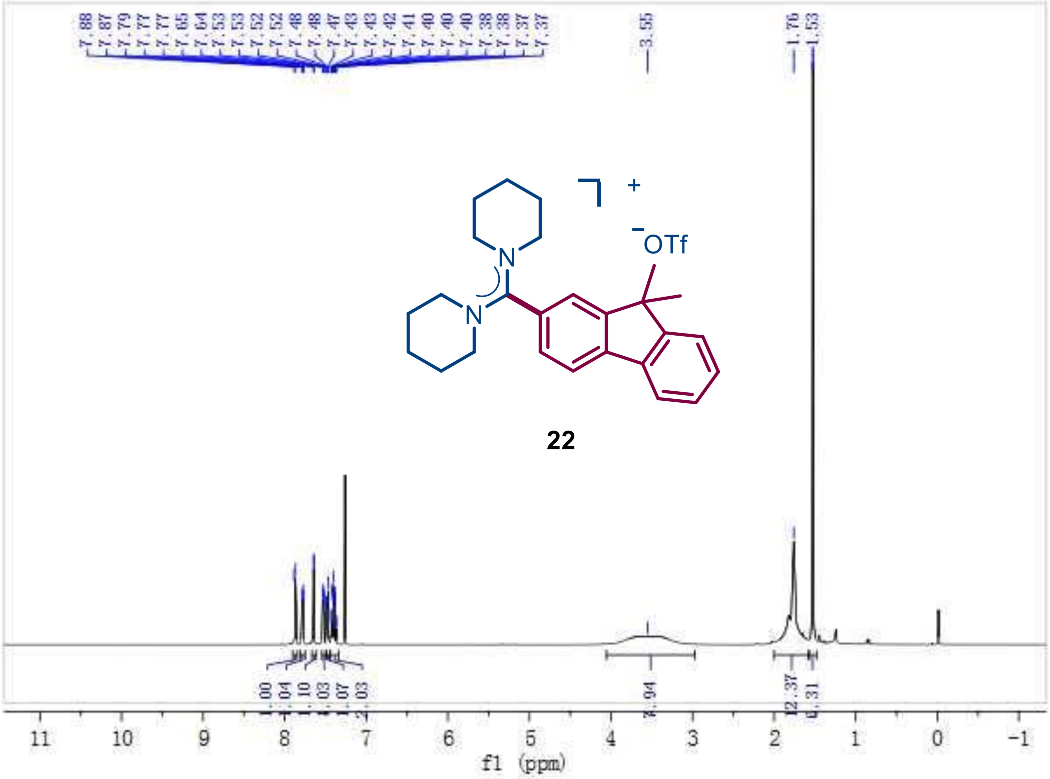

Supplementary Figure 70. ^{1}H NMR Spectrum of Compound **22**

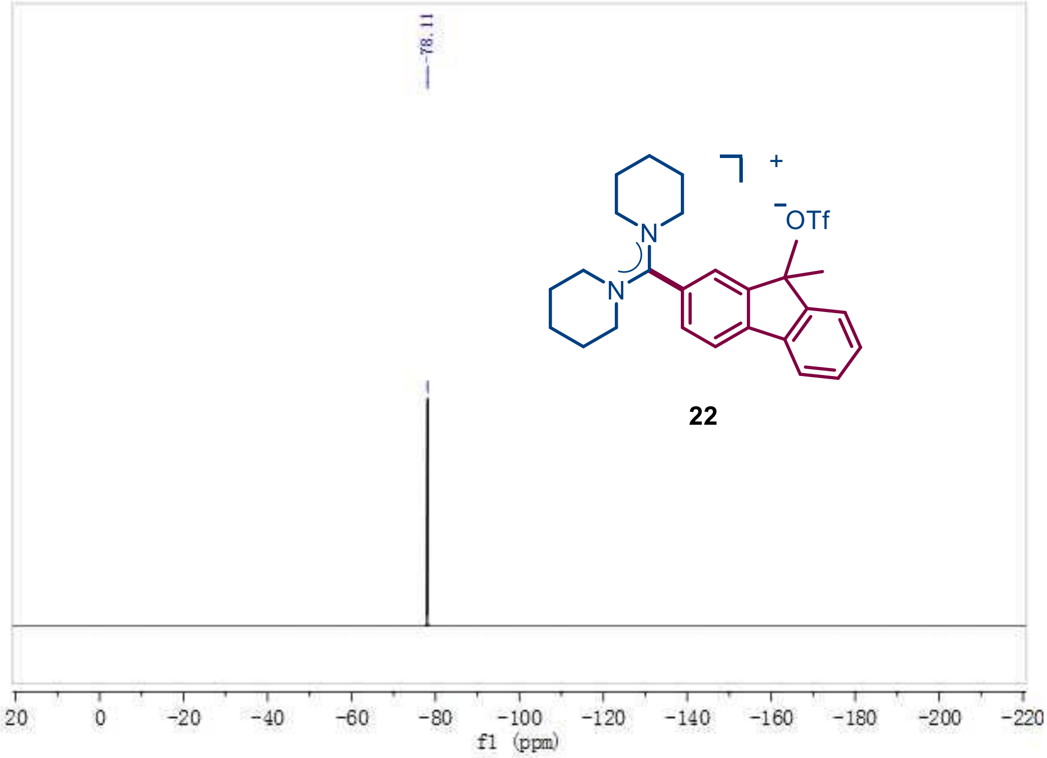

Supplementary Figure 71. ^{19}F NMR Spectrum of Compound **22**

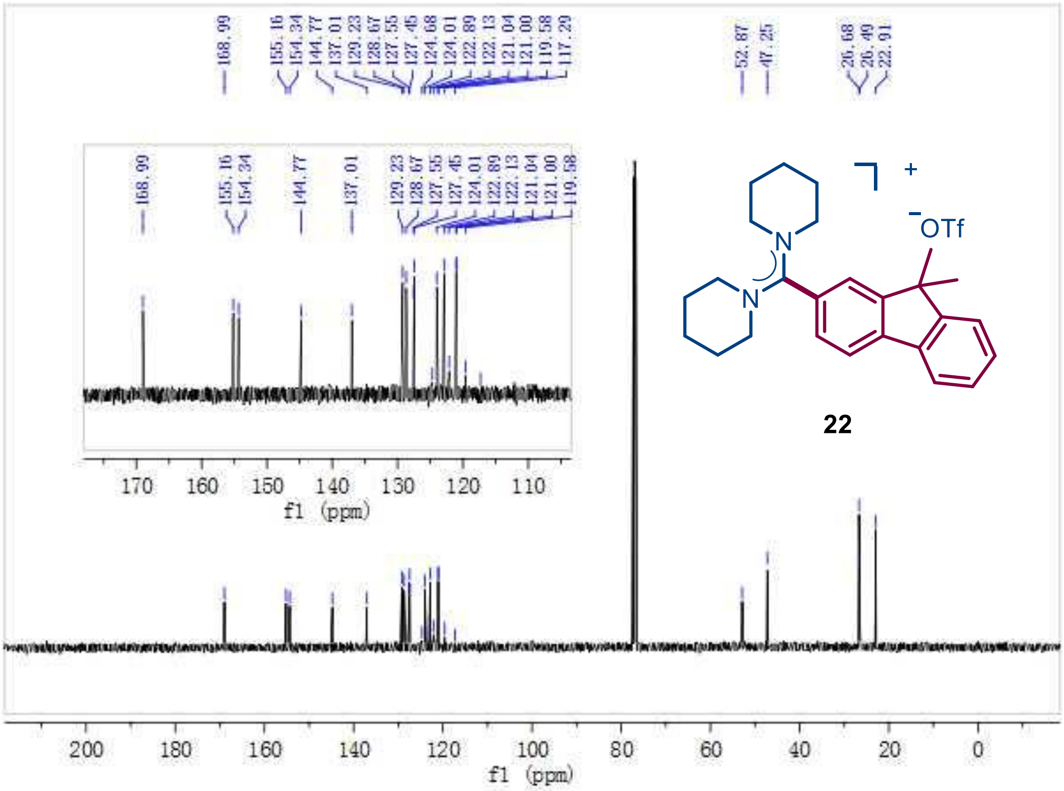

Supplementary Figure 72. ^{13}C NMR Spectrum of Compound **22**

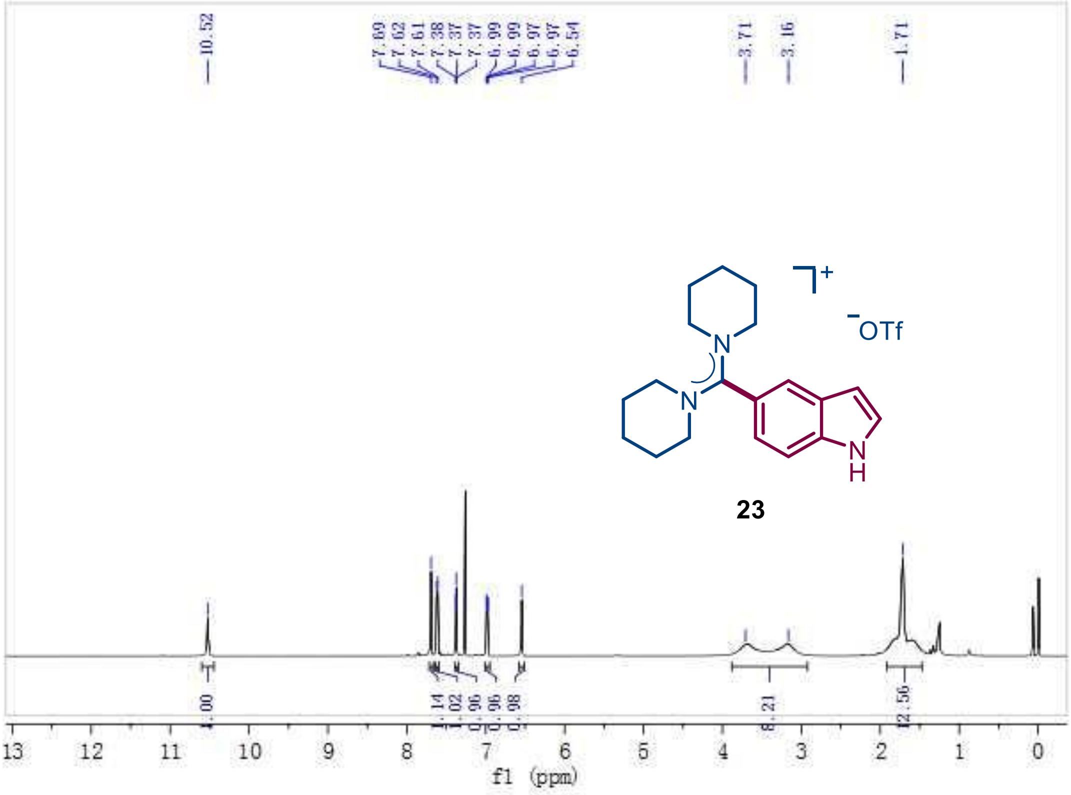

Supplementary Figure 73. ^{1}H NMR Spectrum of Compound **23**

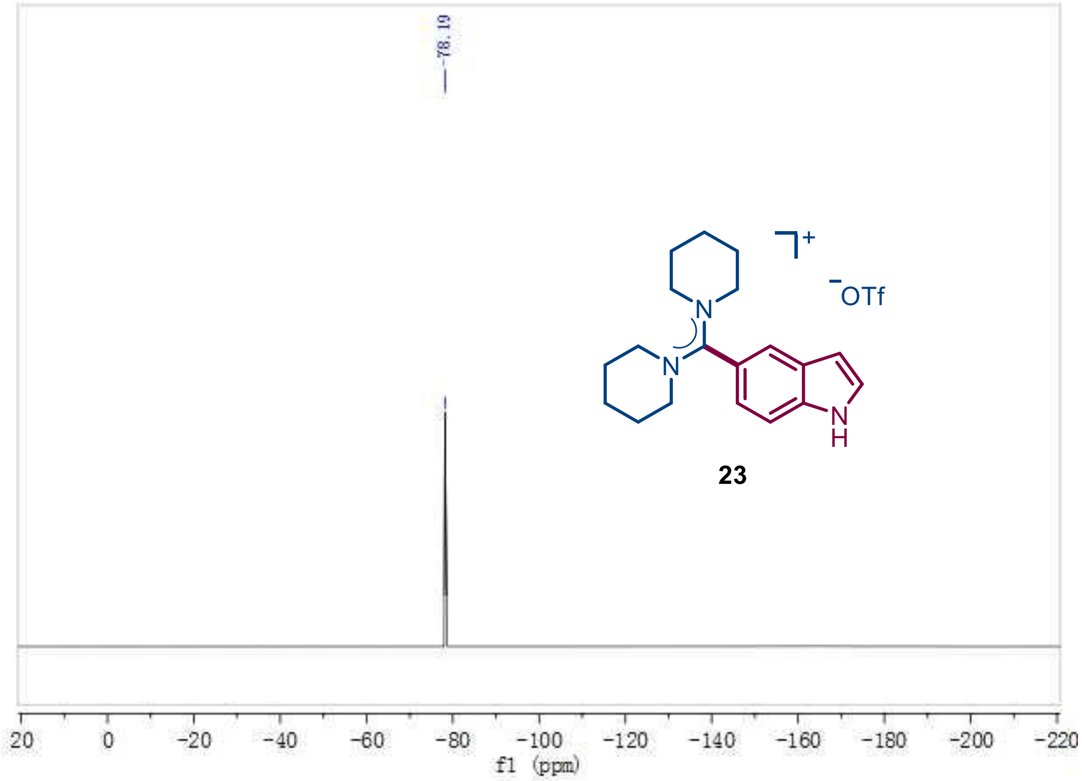

Supplementary Figure 74. ^{19}F NMR Spectrum of Compound **23**

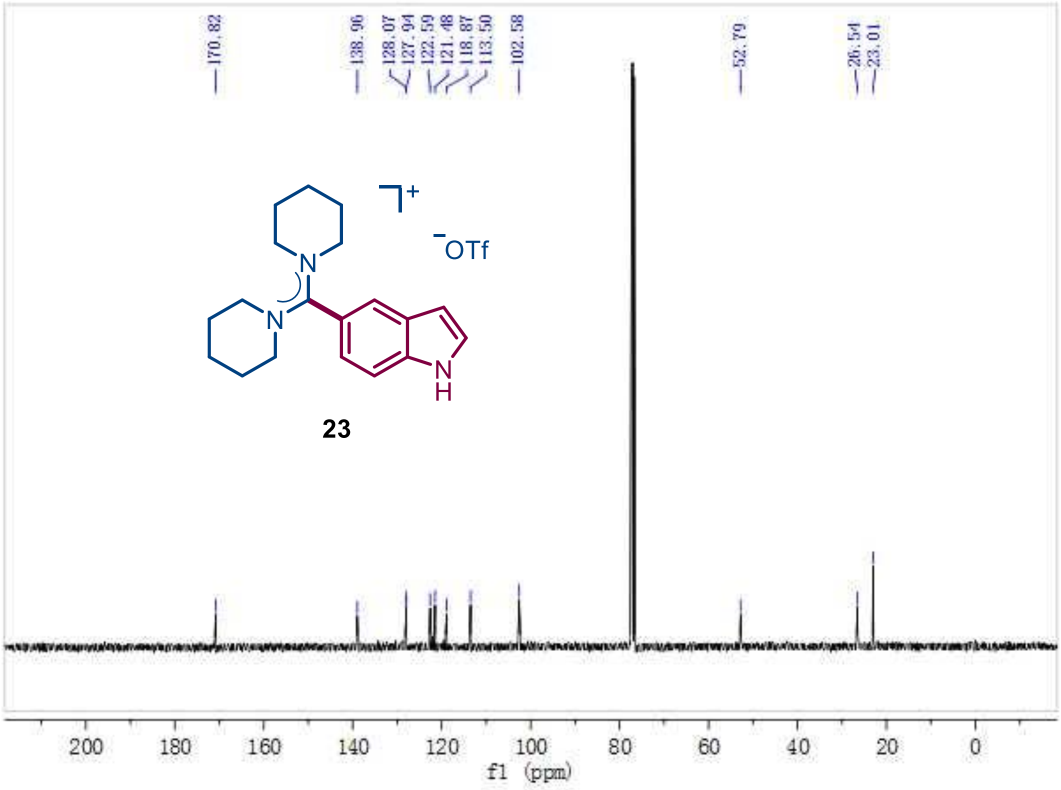

Supplementary Figure 75. ^{13}C NMR Spectrum of Compound **23**

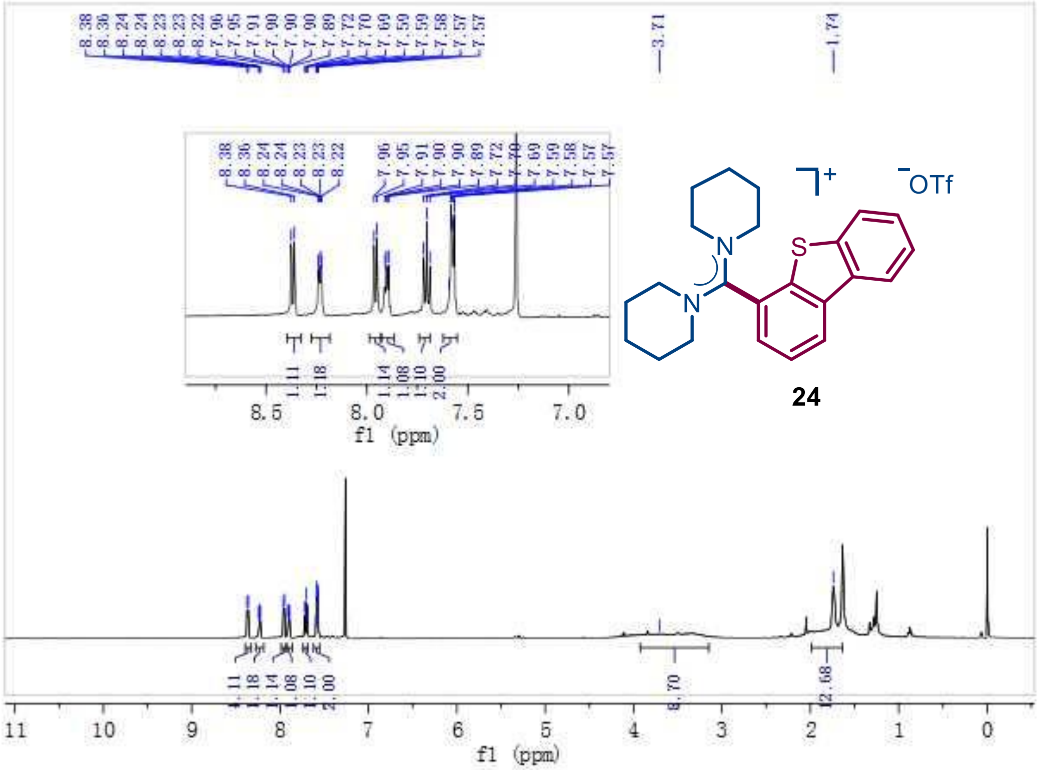

Supplementary Figure 76. ^{1}H NMR Spectrum of Compound **24**

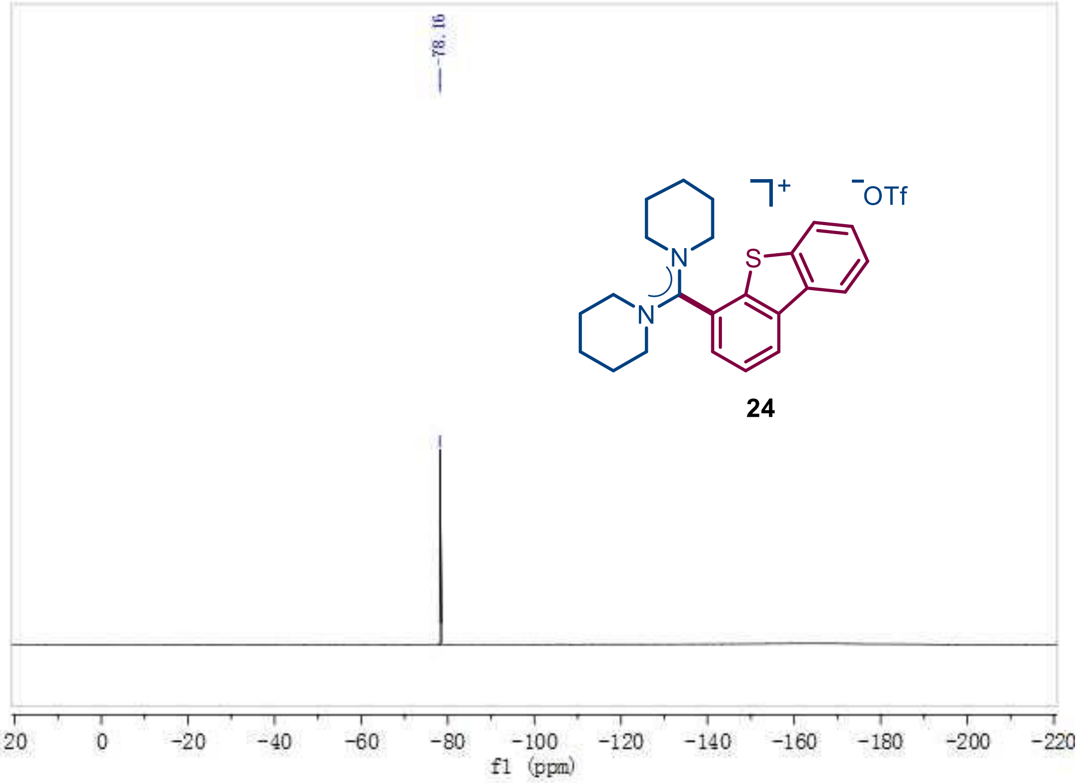

Supplementary Figure 77. ^{19}F NMR Spectrum of Compound **24**

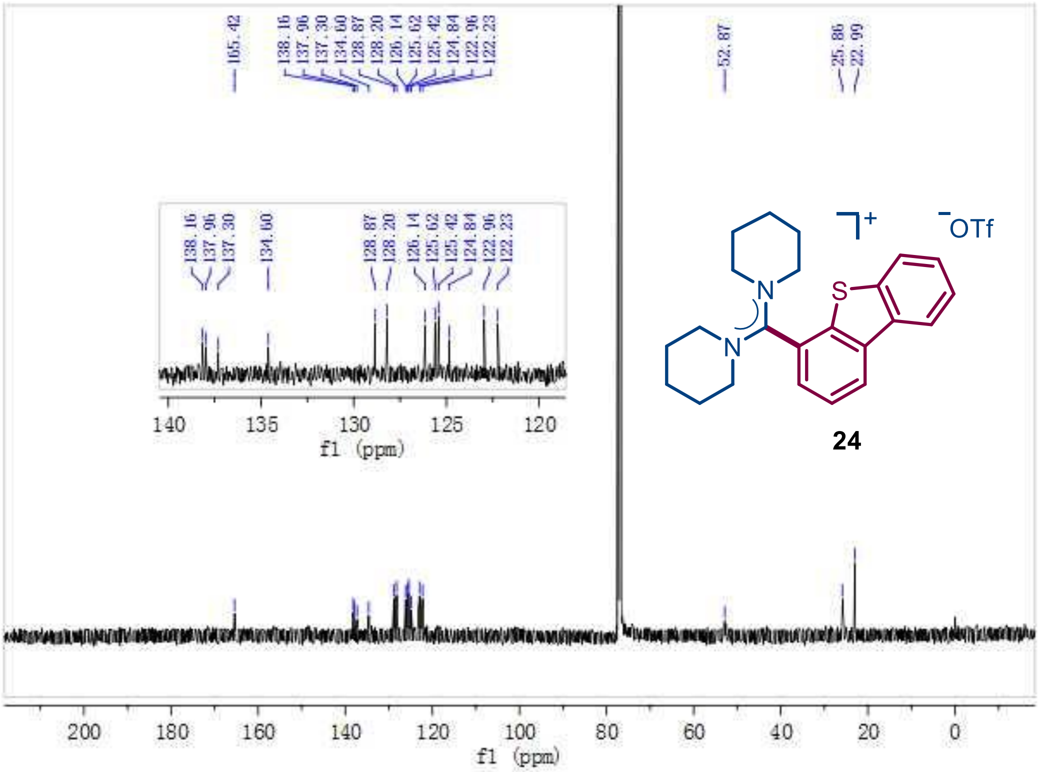

Supplementary Figure 78. ^{13}C NMR Spectrum of Compound **24**

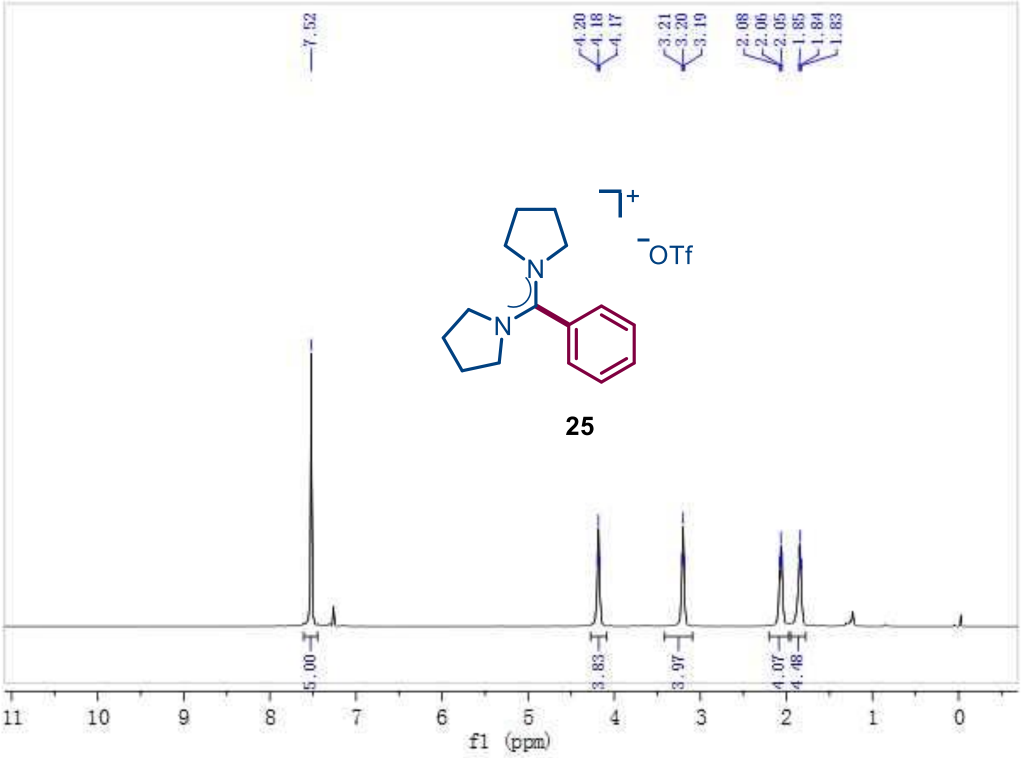

Supplementary Figure 79. ^{1}H NMR Spectrum of Compound **25**

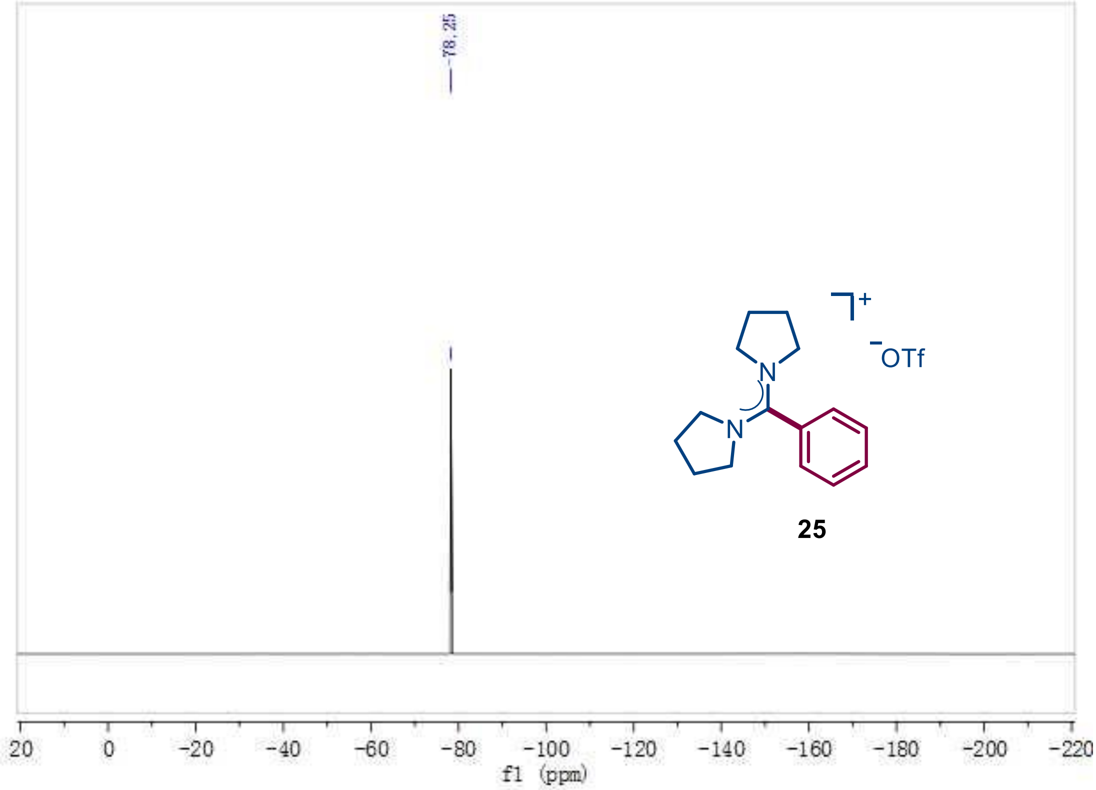

Supplementary Figure 80. ^{19}F NMR Spectrum of Compound **25**

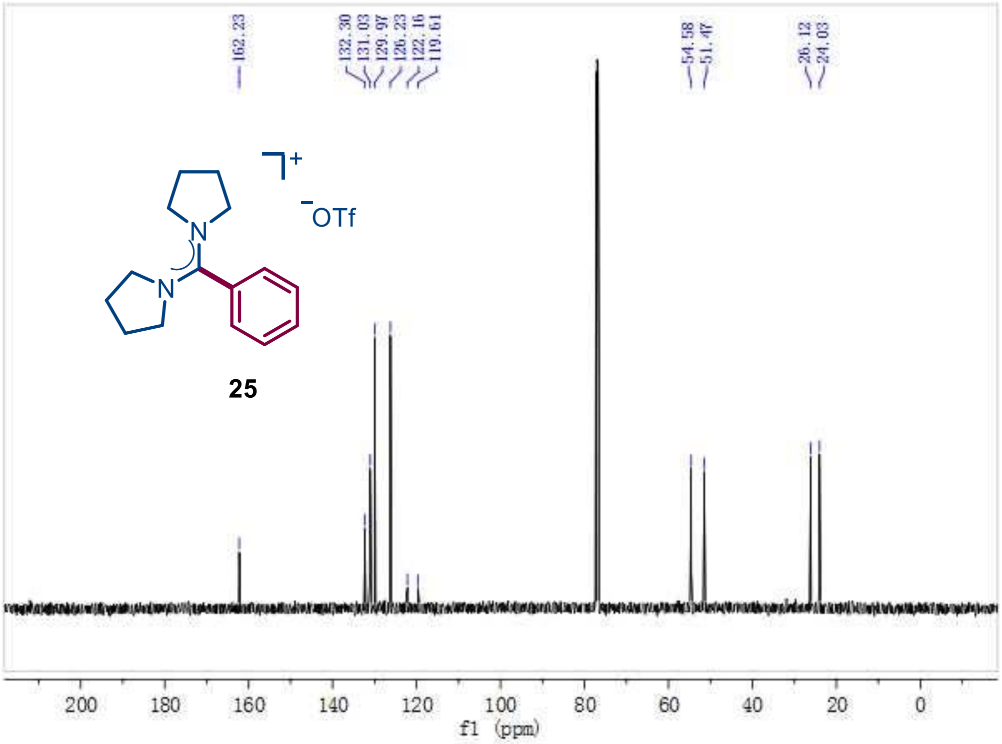

Supplementary Figure 81. ^{13}C NMR Spectrum of Compound **25**

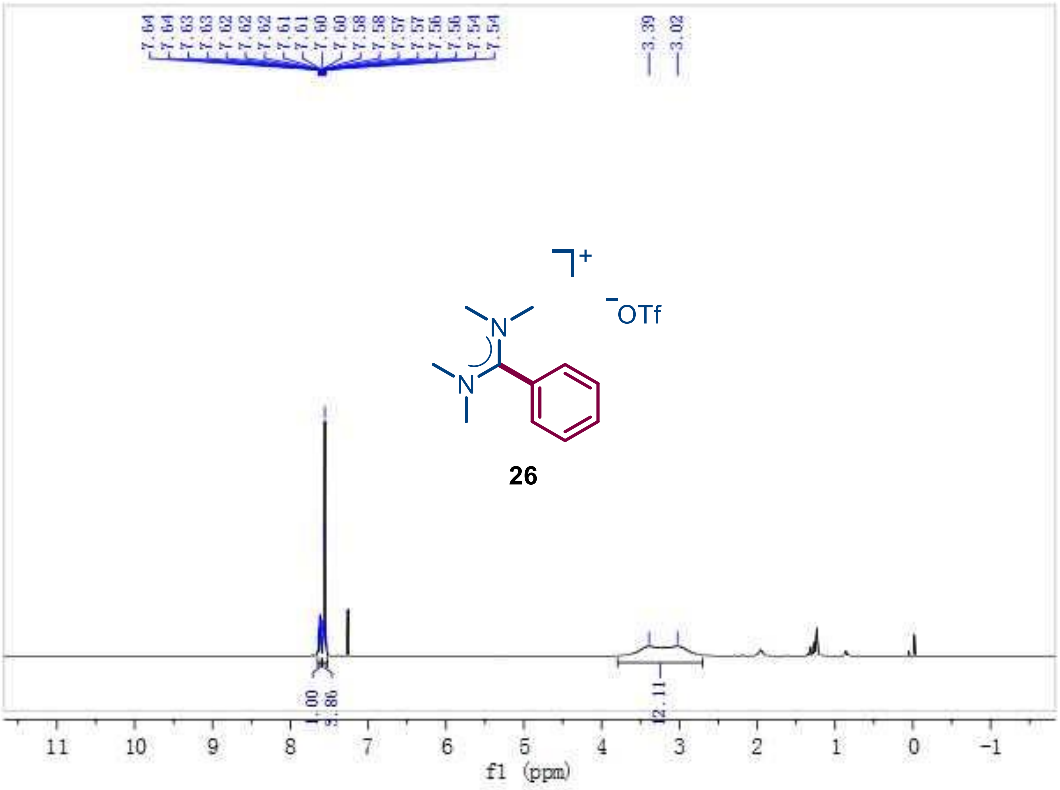

Supplementary Figure 82. ^{1}H NMR Spectrum of Compound **26**

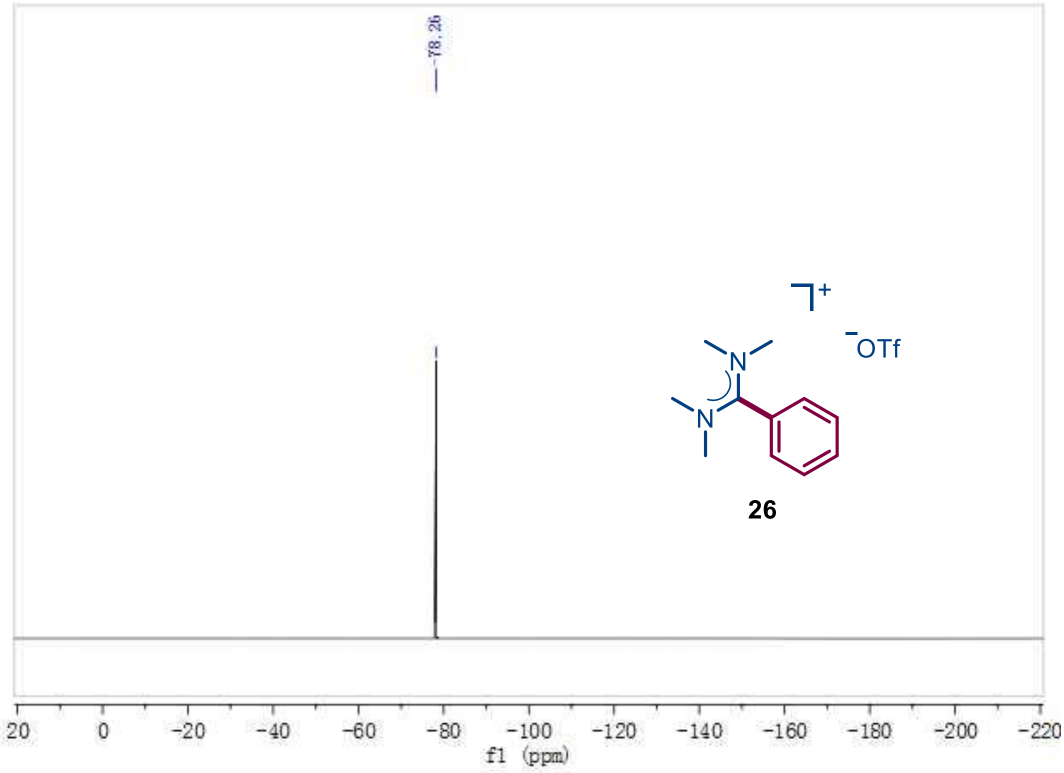

Supplementary Figure 83. ^{19}F NMR Spectrum of Compound **26**

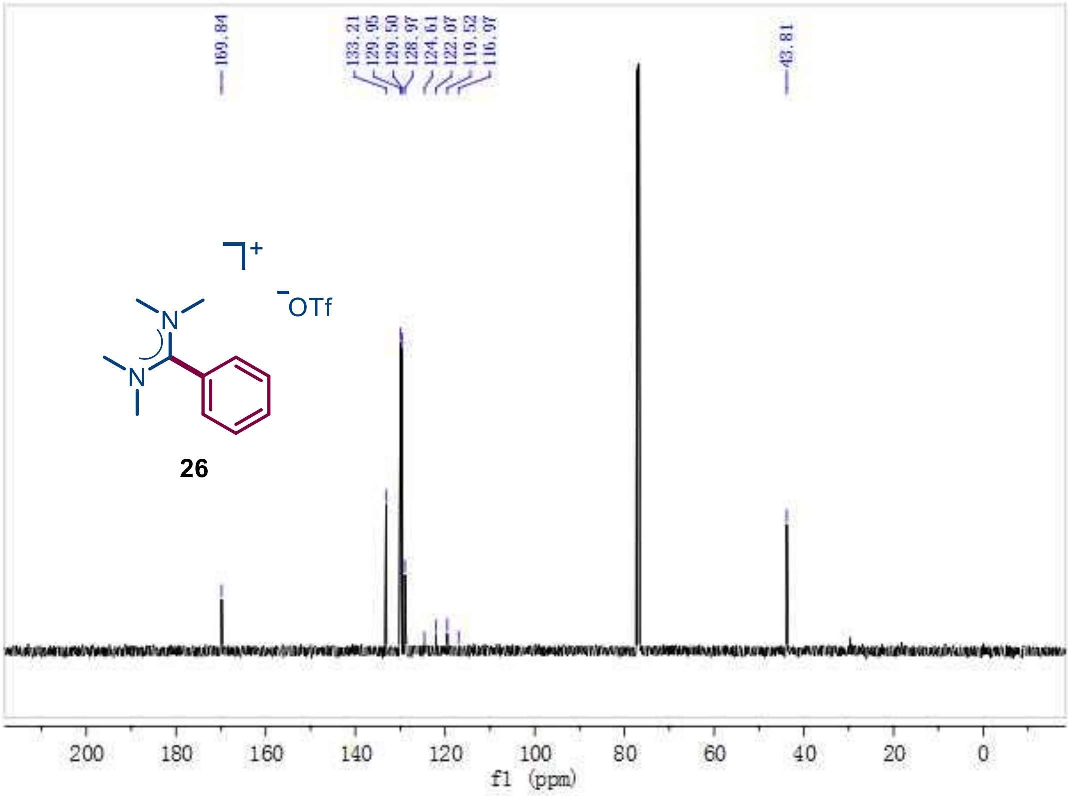

Supplementary Figure 84. ^{13}C NMR Spectrum of Compound **26**

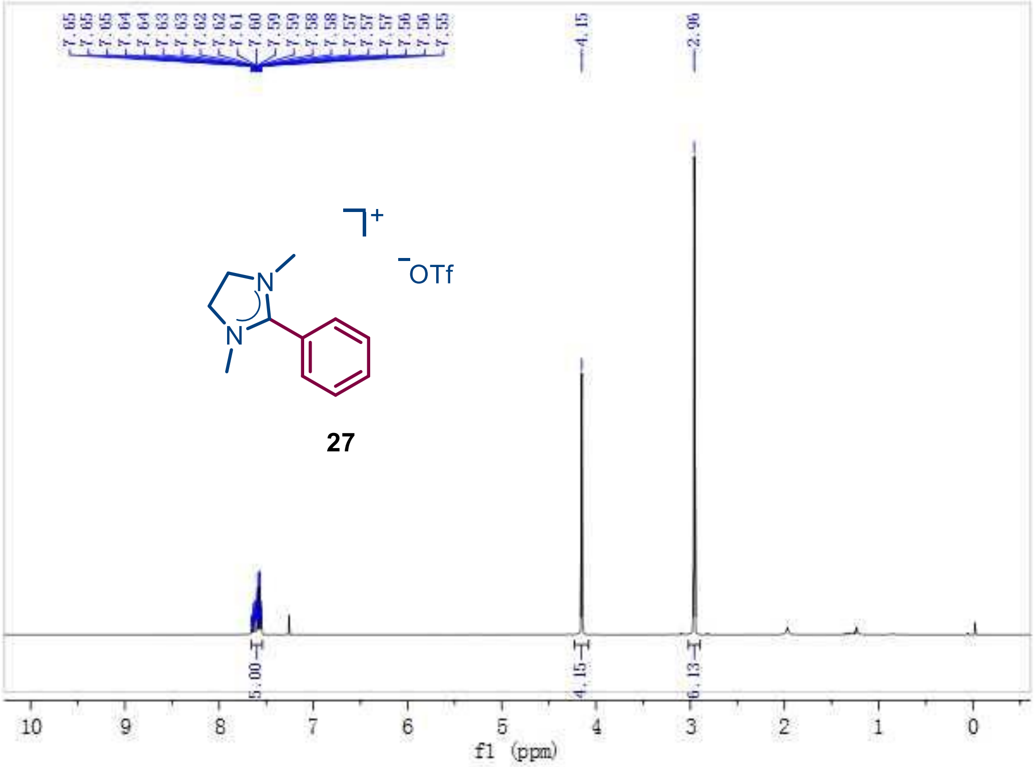

Supplementary Figure 85. ^{1}H NMR Spectrum of Compound **27**

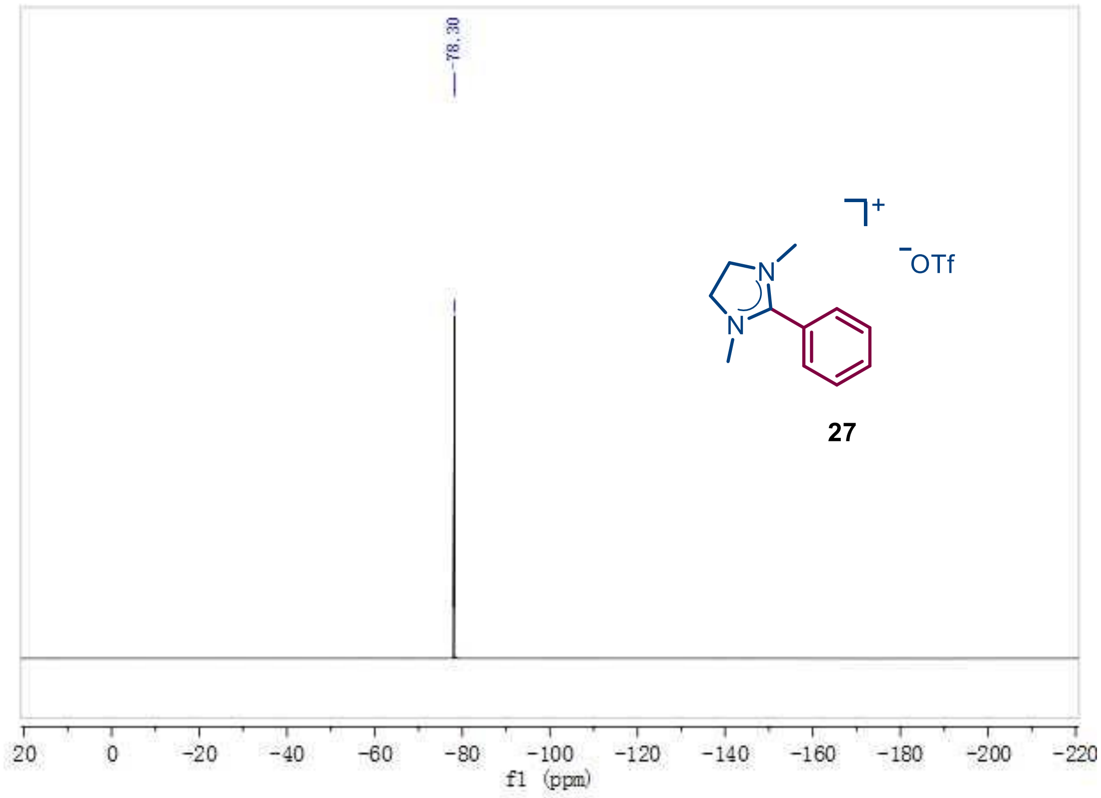

Supplementary Figure 86. ^{19}F NMR Spectrum of Compound **27**

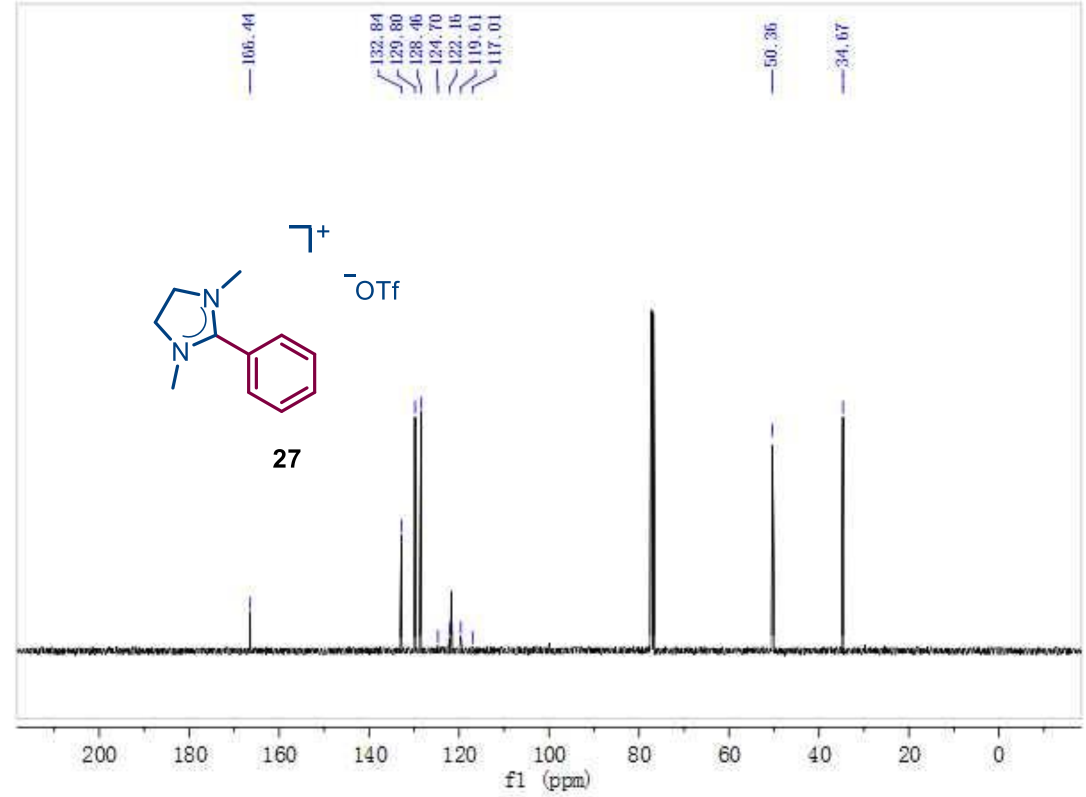

Supplementary Figure 87. ^{13}C NMR Spectrum of Compound **27**

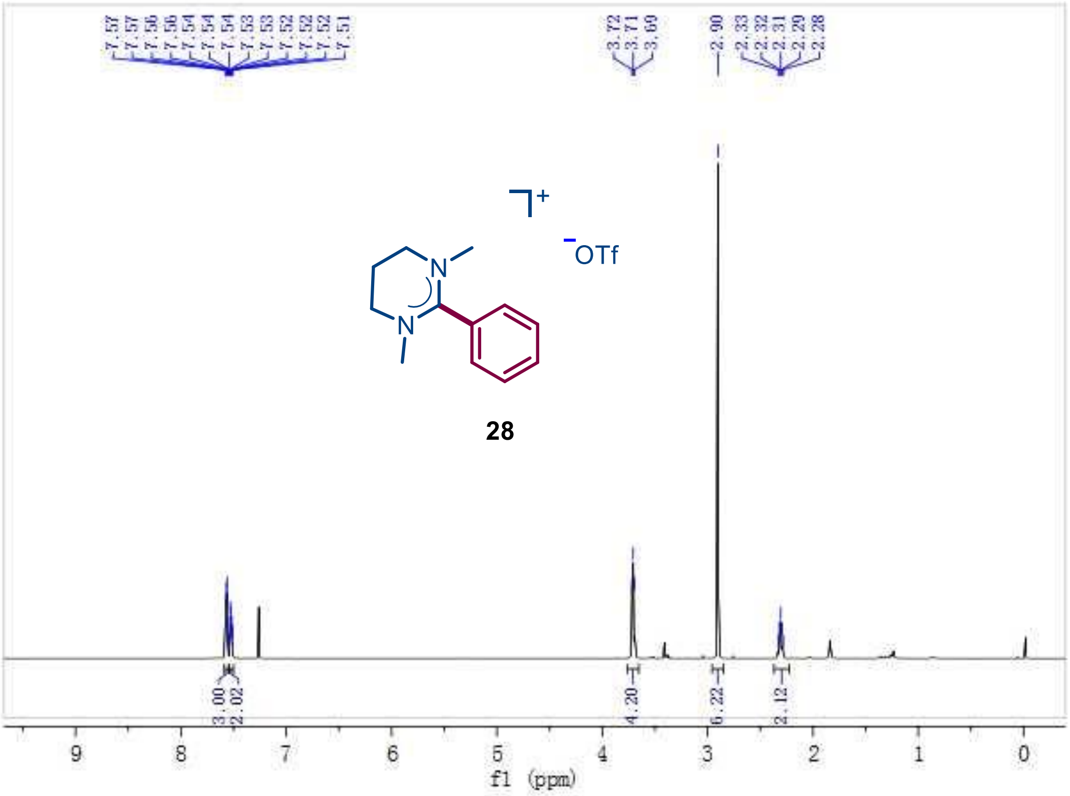

Supplementary Figure 88. ^{1}H NMR Spectrum of Compound **28**

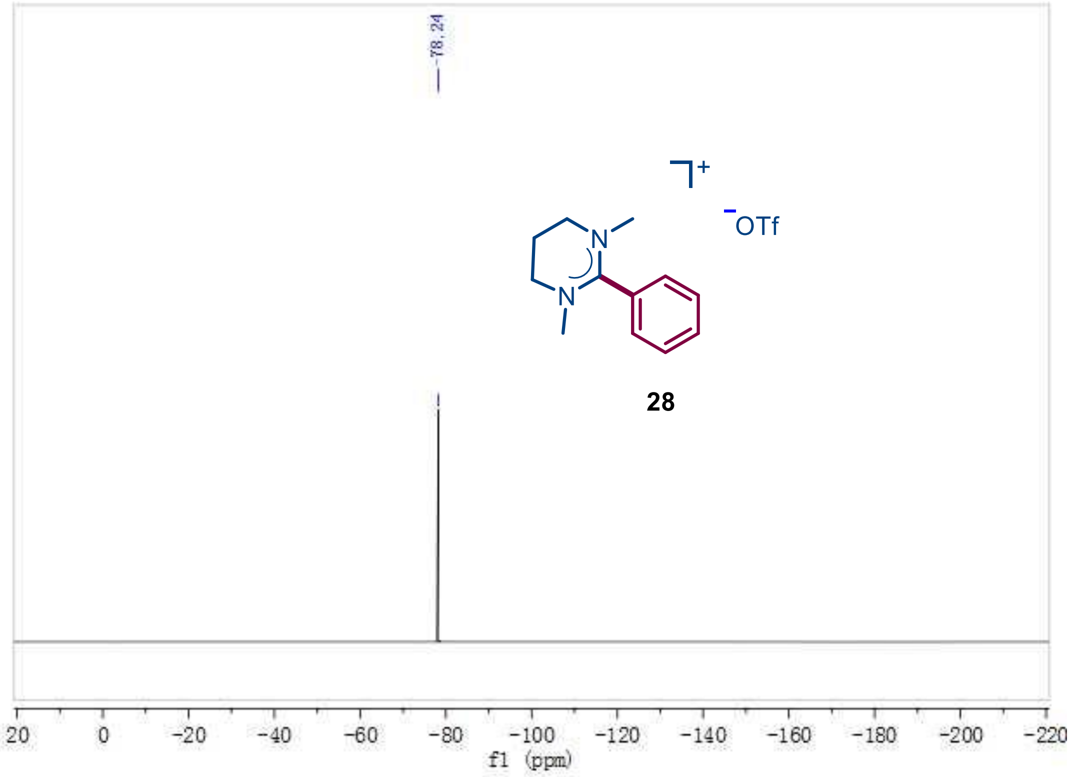

Supplementary Figure 89. ^{19}F NMR Spectrum of Compound **28**

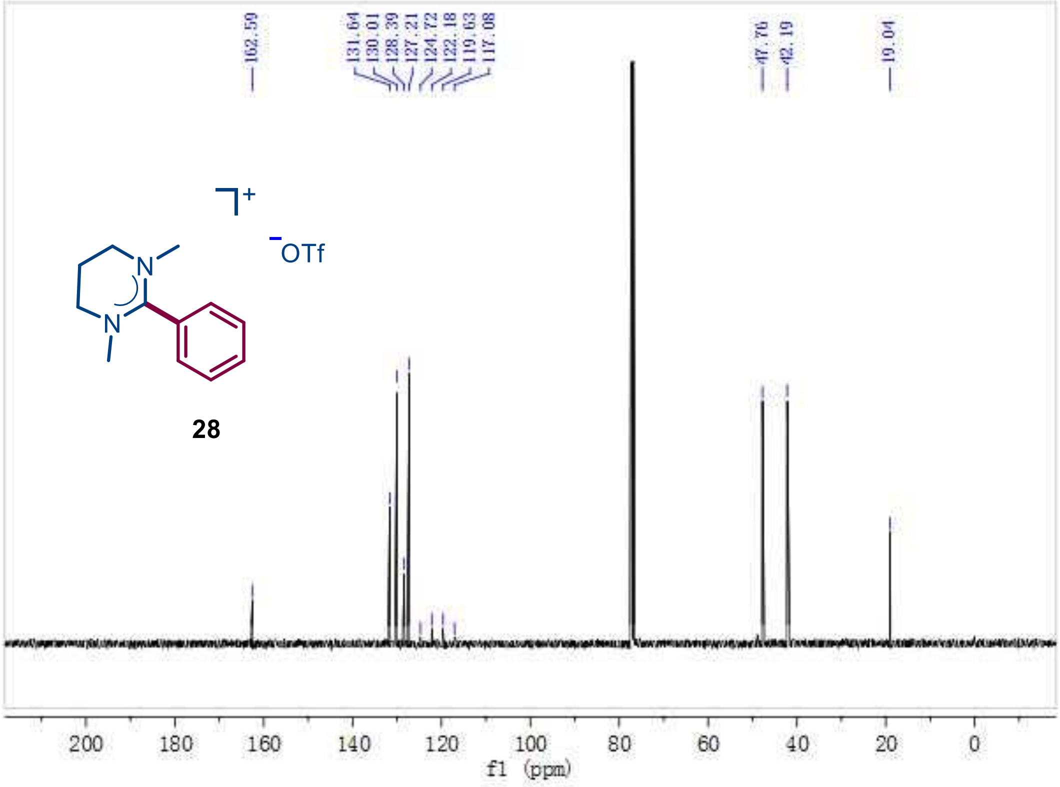

Supplementary Figure 90. ^{13}C NMR Spectrum of Compound **28**

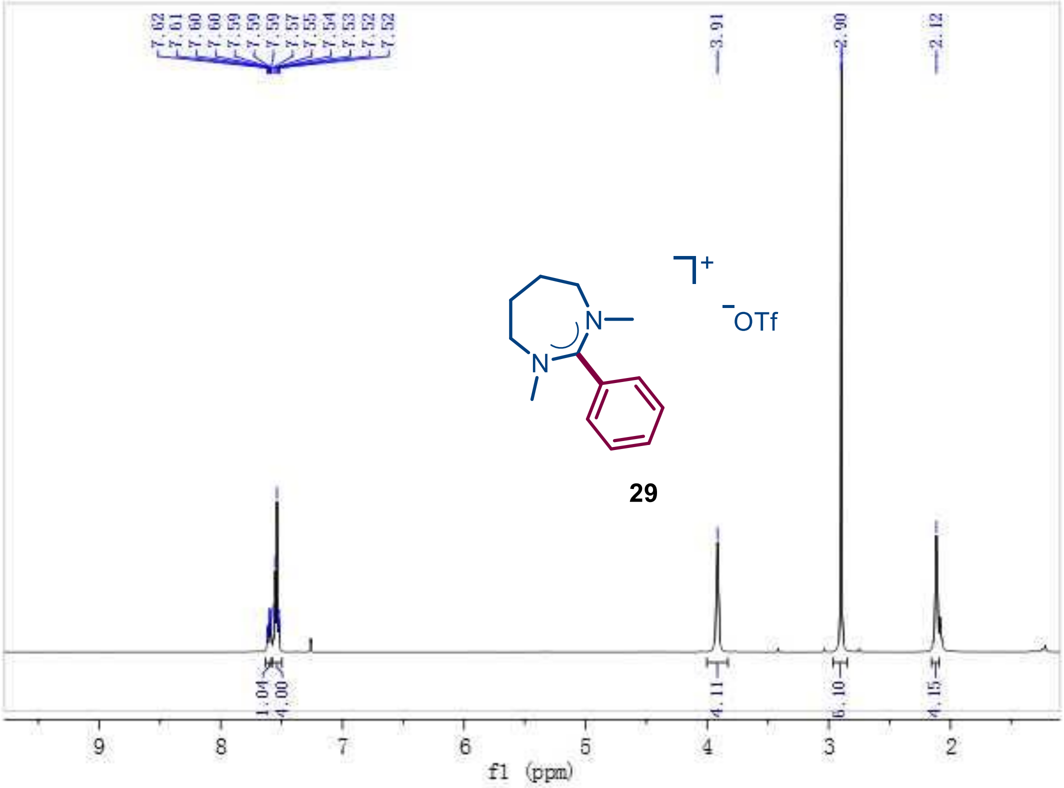

Supplementary Figure 91. ^{1}H NMR Spectrum of Compound **29**

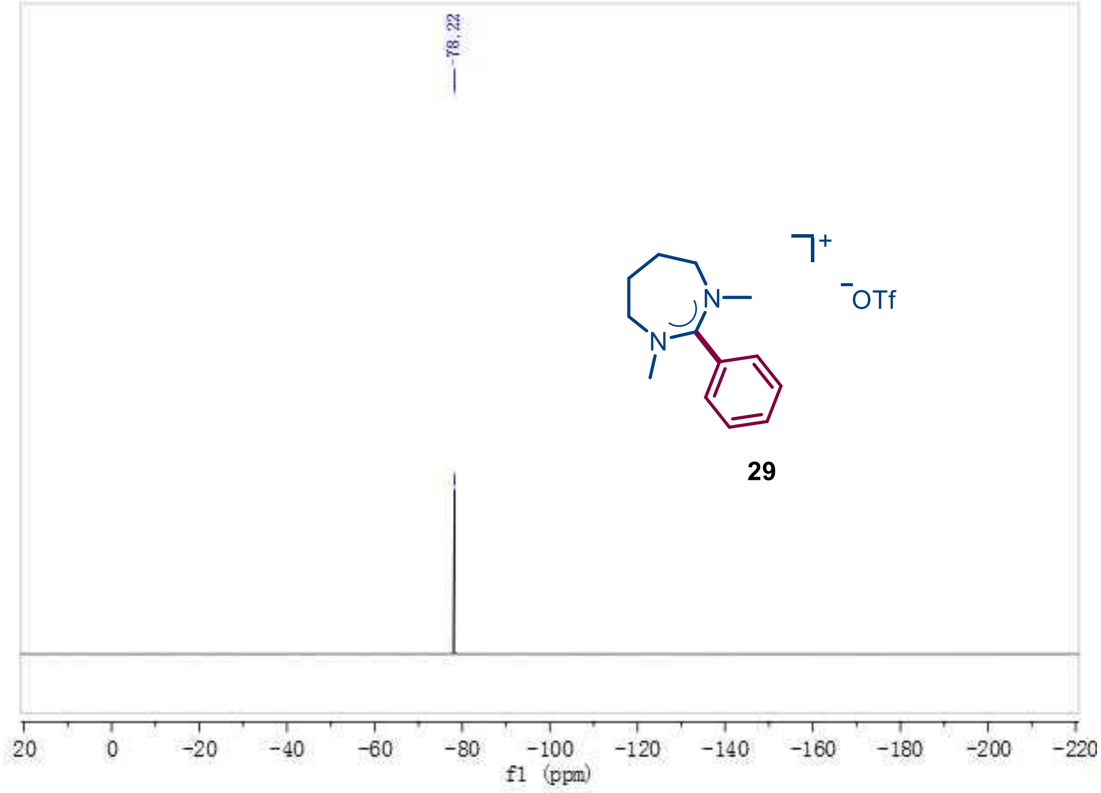

Supplementary Figure 92. ^{19}F NMR Spectrum of Compound **29**

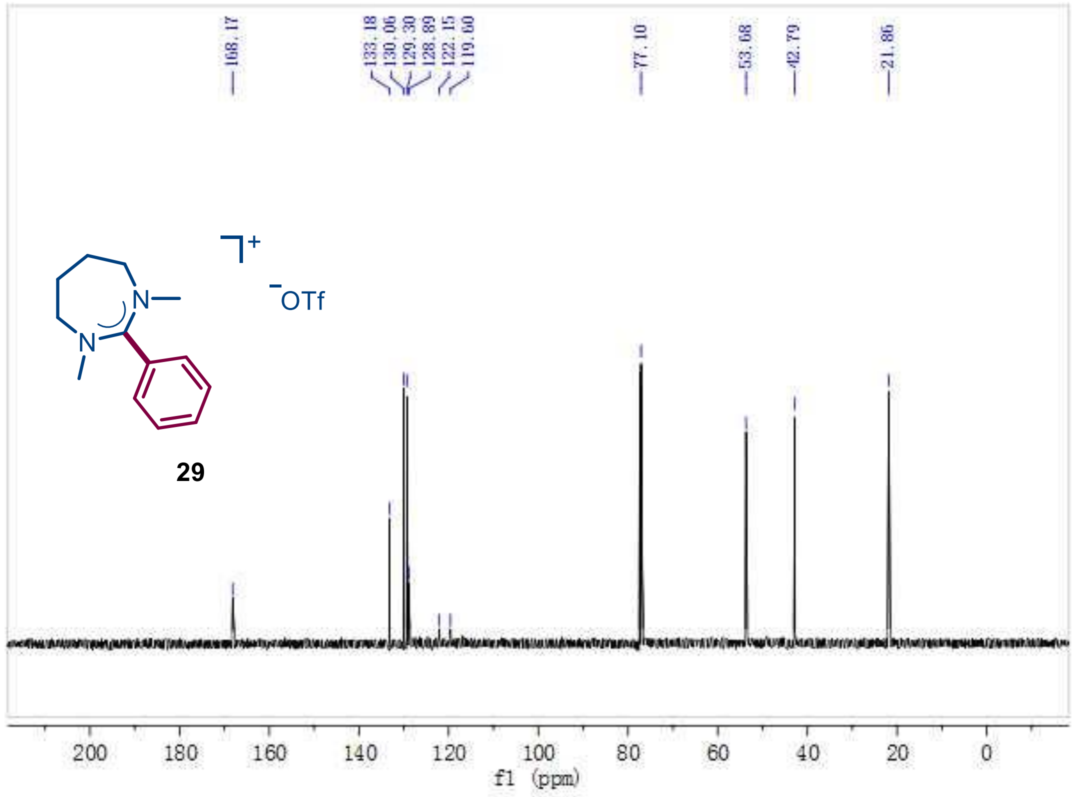

Supplementary Figure 93. ^{13}C NMR Spectrum of Compound **29**

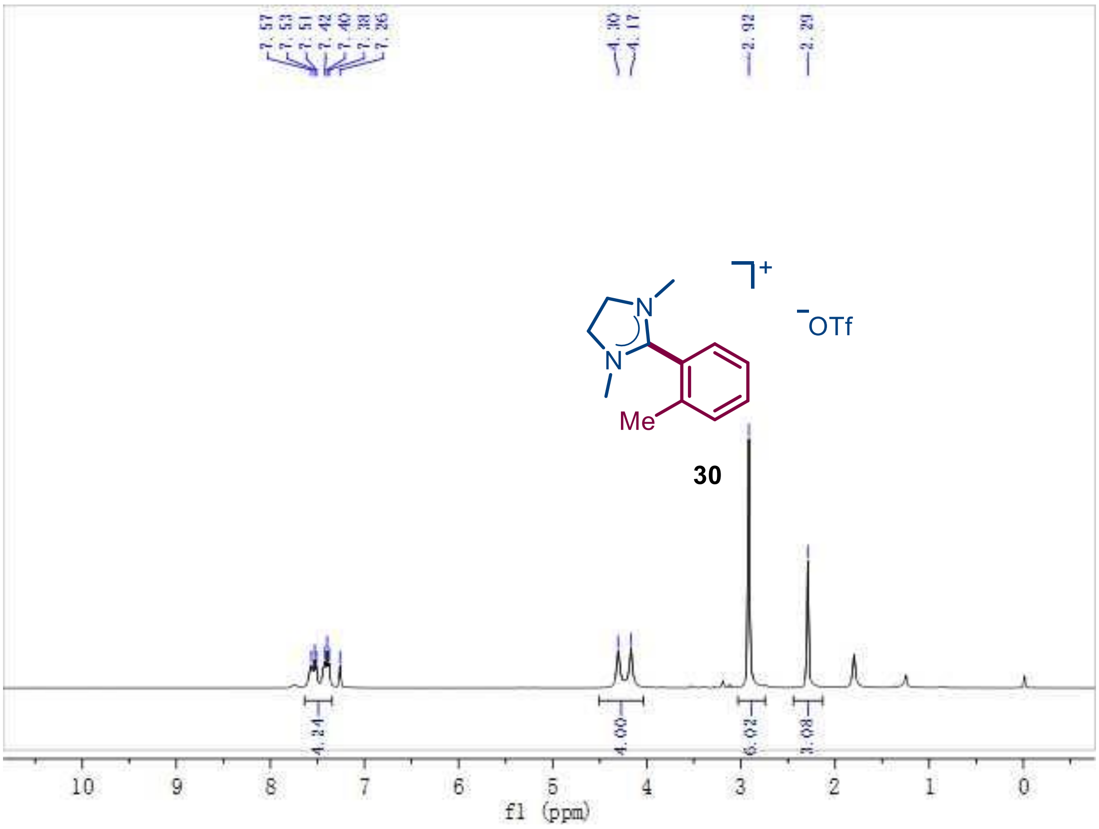

Supplementary Figure 94. ^{1}H NMR Spectrum of Compound **30**

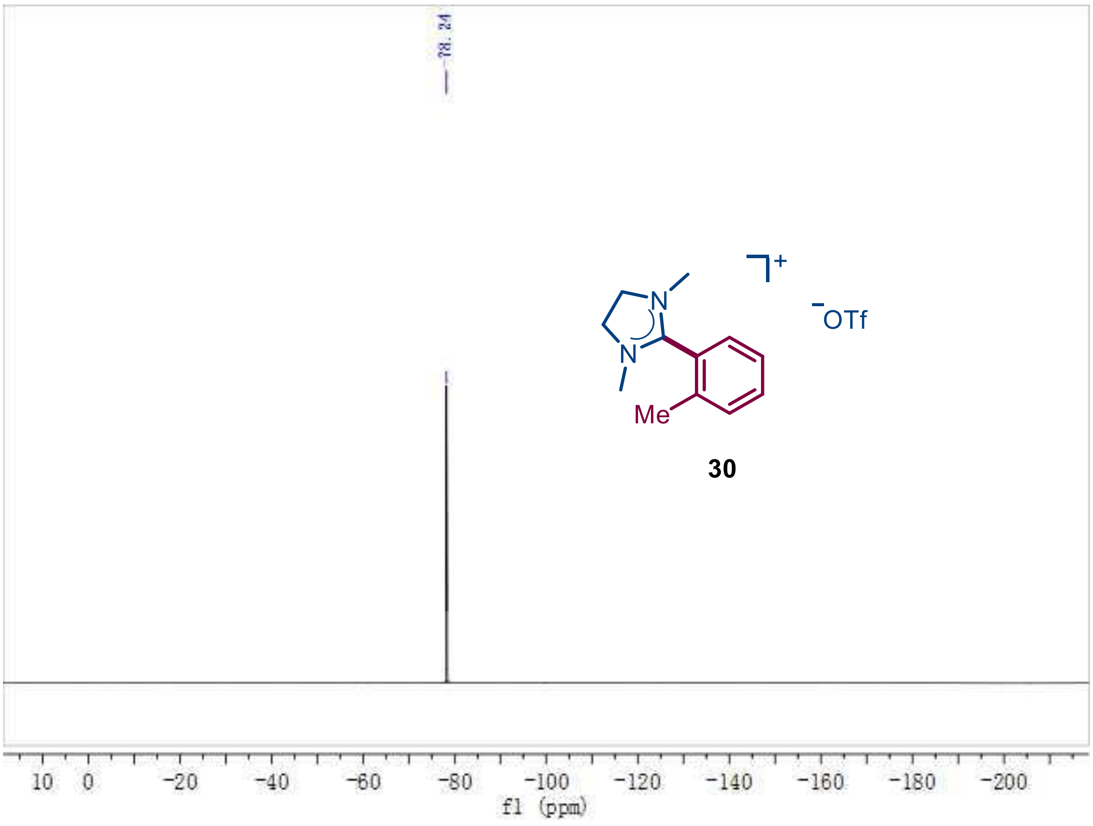

Supplementary Figure 95. ^{19}F NMR Spectrum of Compound **30**

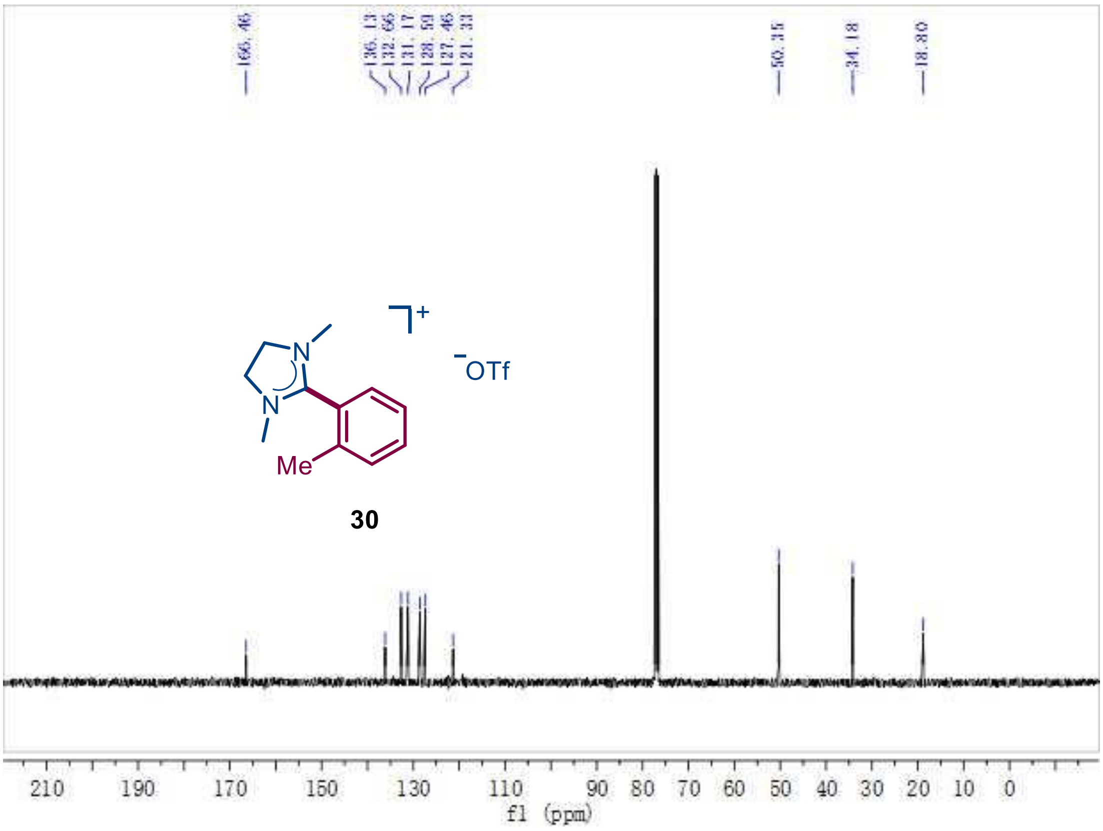

Supplementary Figure 96. ^{13}C NMR Spectrum of Compound **30**

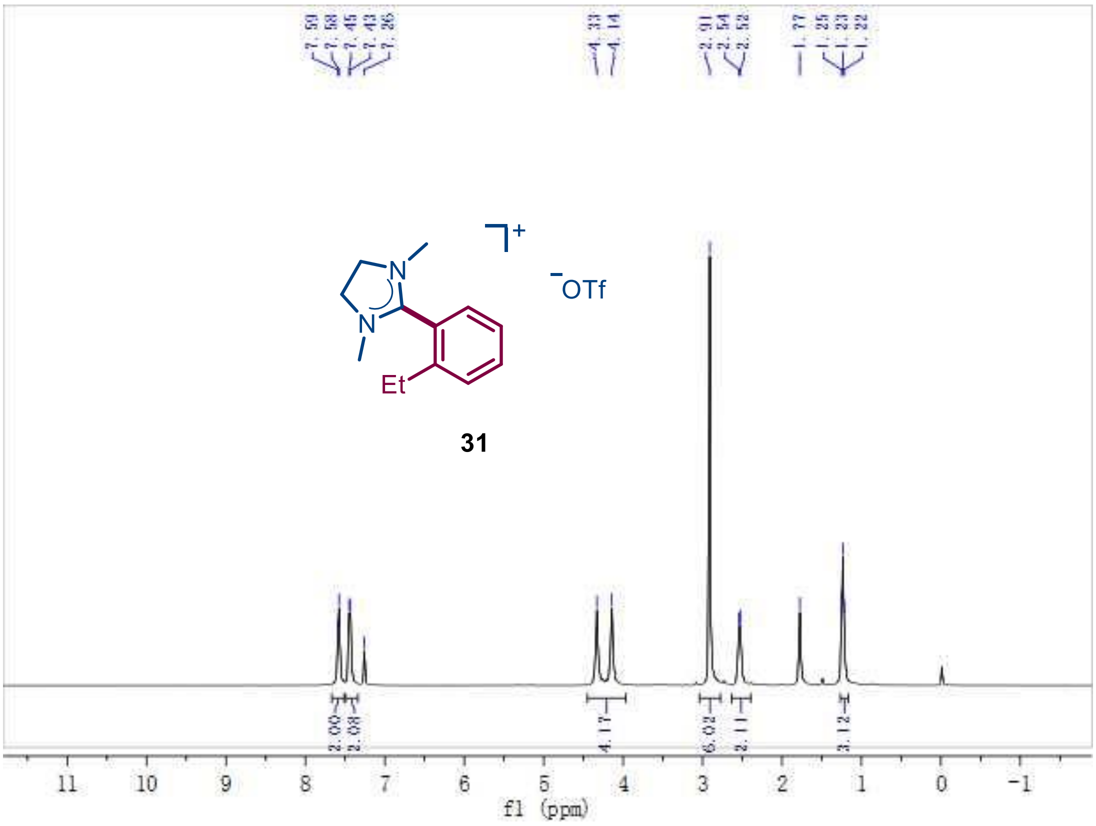

Supplementary Figure 97. ^{1}H NMR Spectrum of Compound **31**

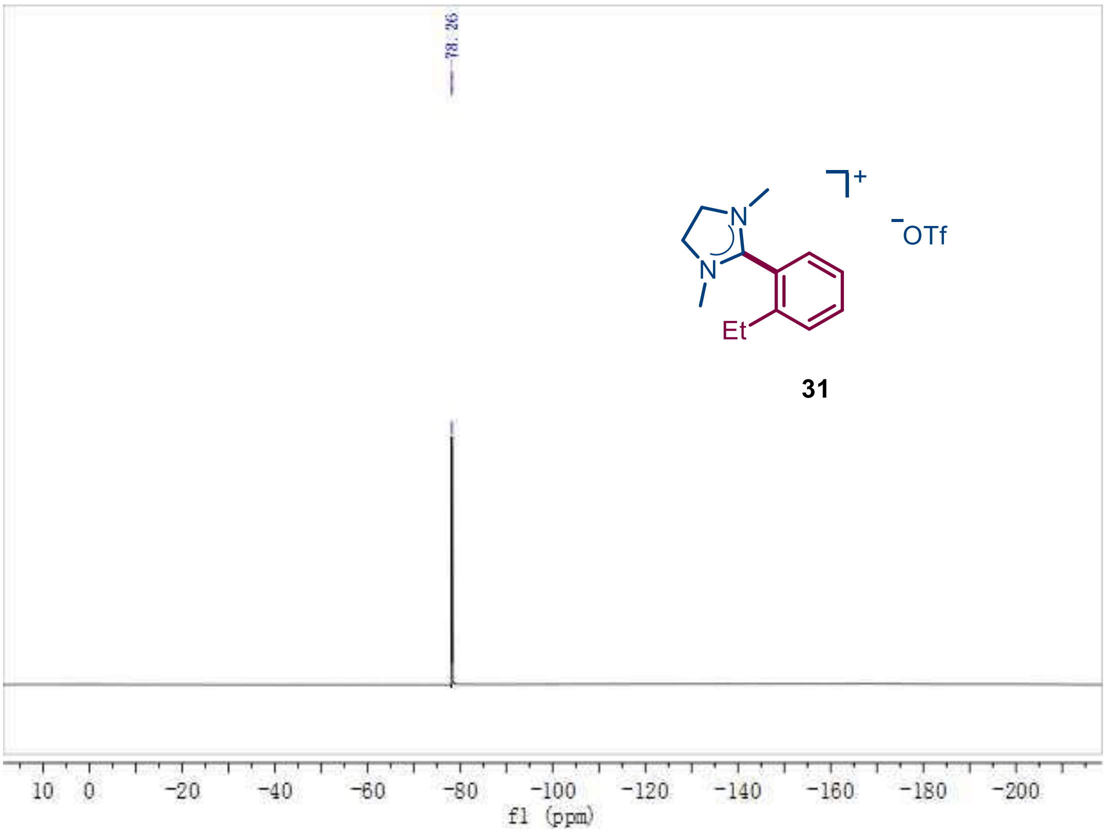

Supplementary Figure 98. ^{19}F NMR Spectrum of Compound **31**

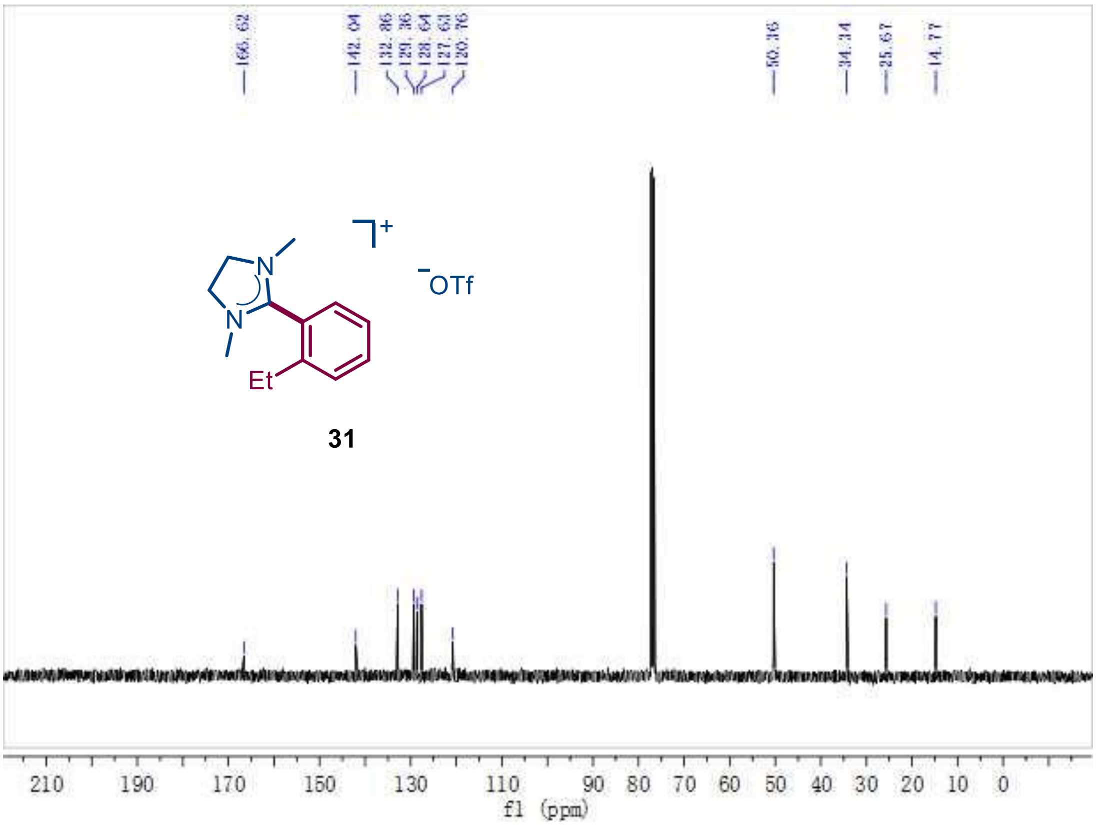

Supplementary Figure 99. ^{13}C NMR Spectrum of Compound **31**

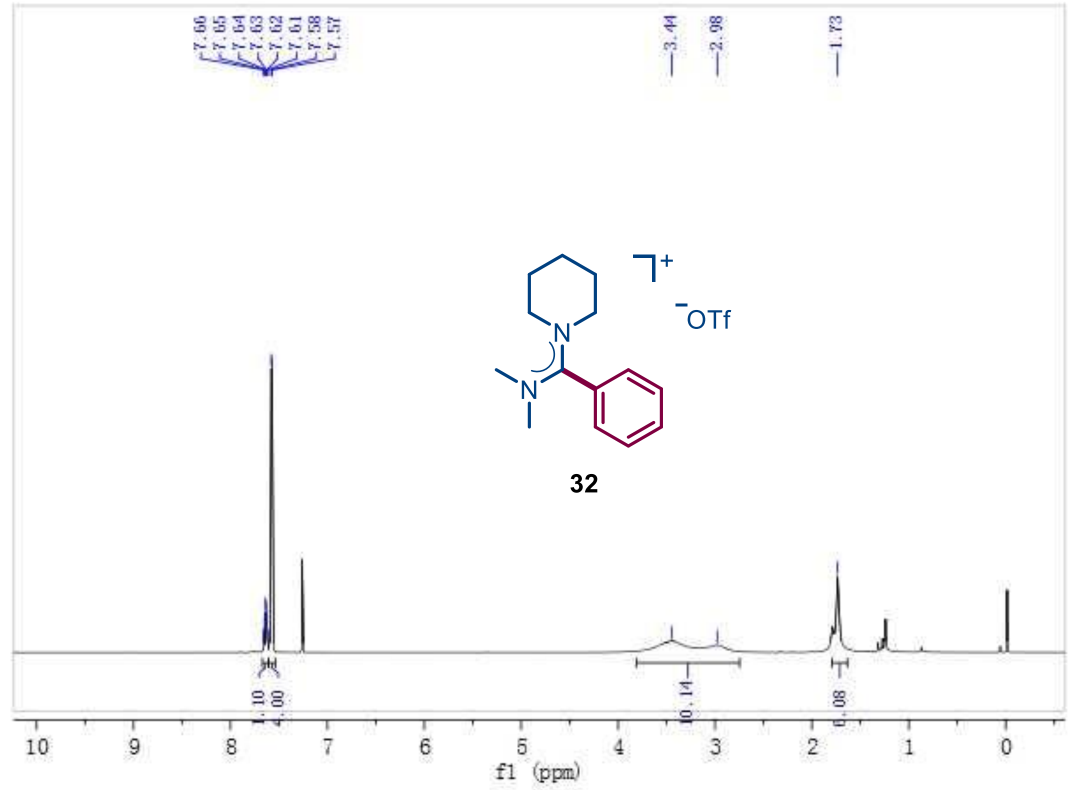

Supplementary Figure 100. ^{1}H NMR Spectrum of Compound **32**

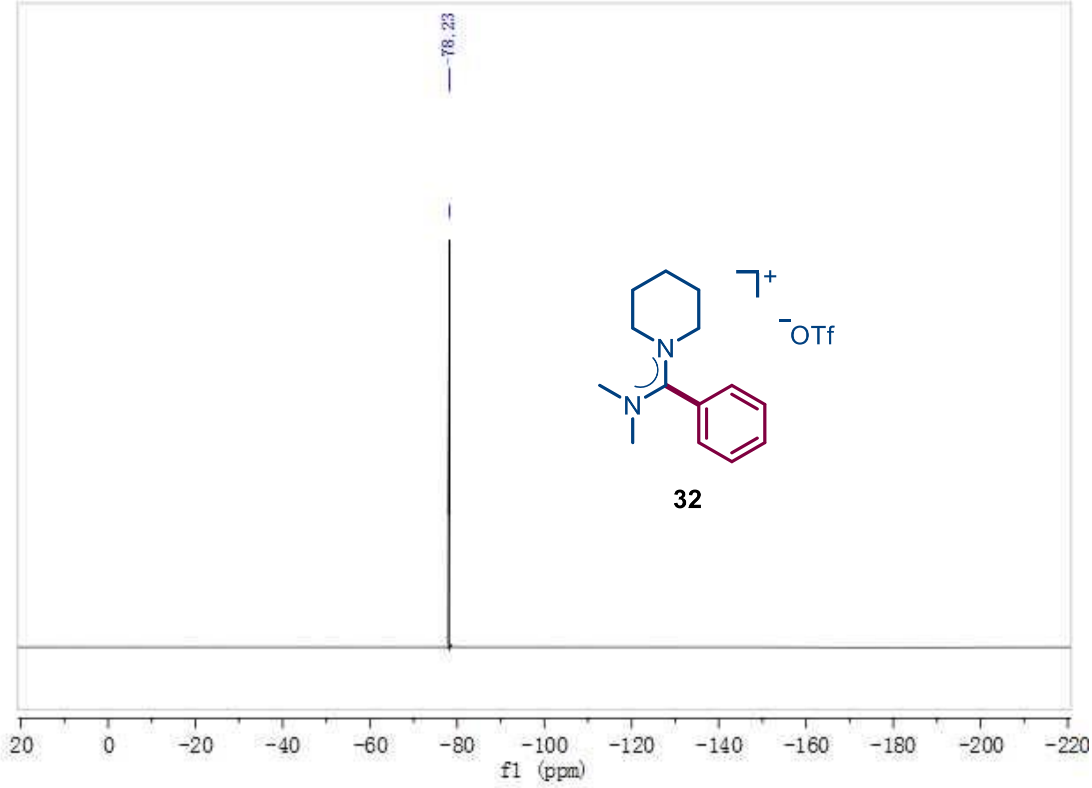

Supplementary Figure 101. ^{19}F NMR Spectrum of Compound **32**

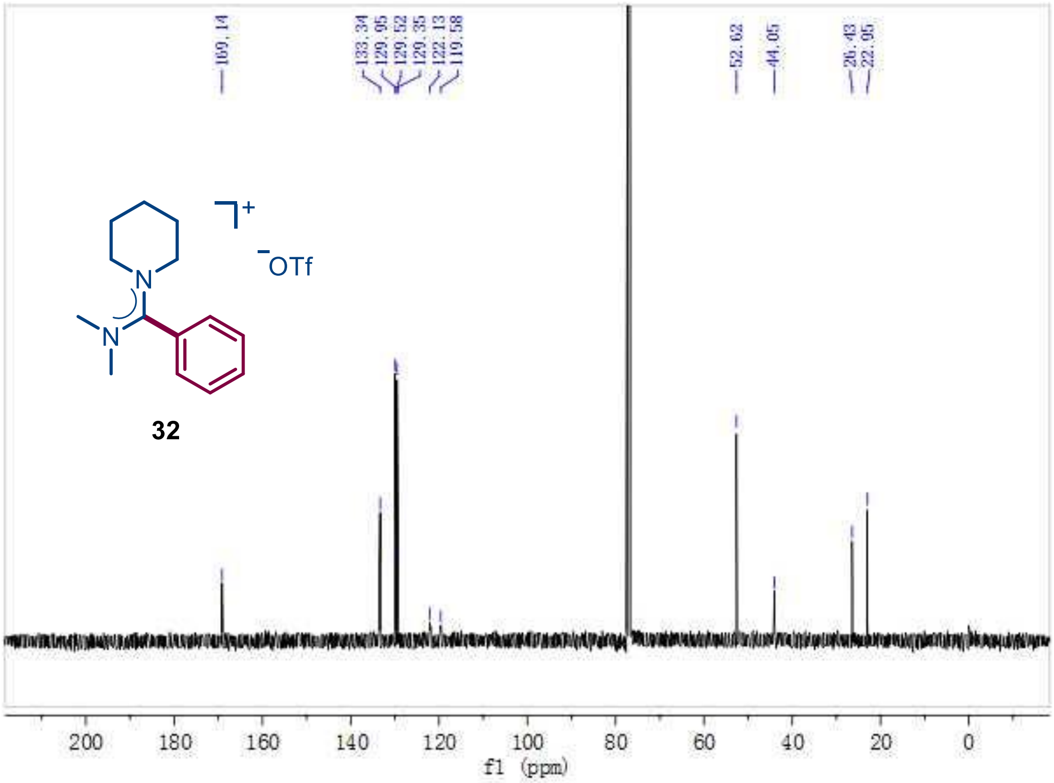

Supplementary Figure 102. ^{13}C NMR Spectrum of Compound **32**

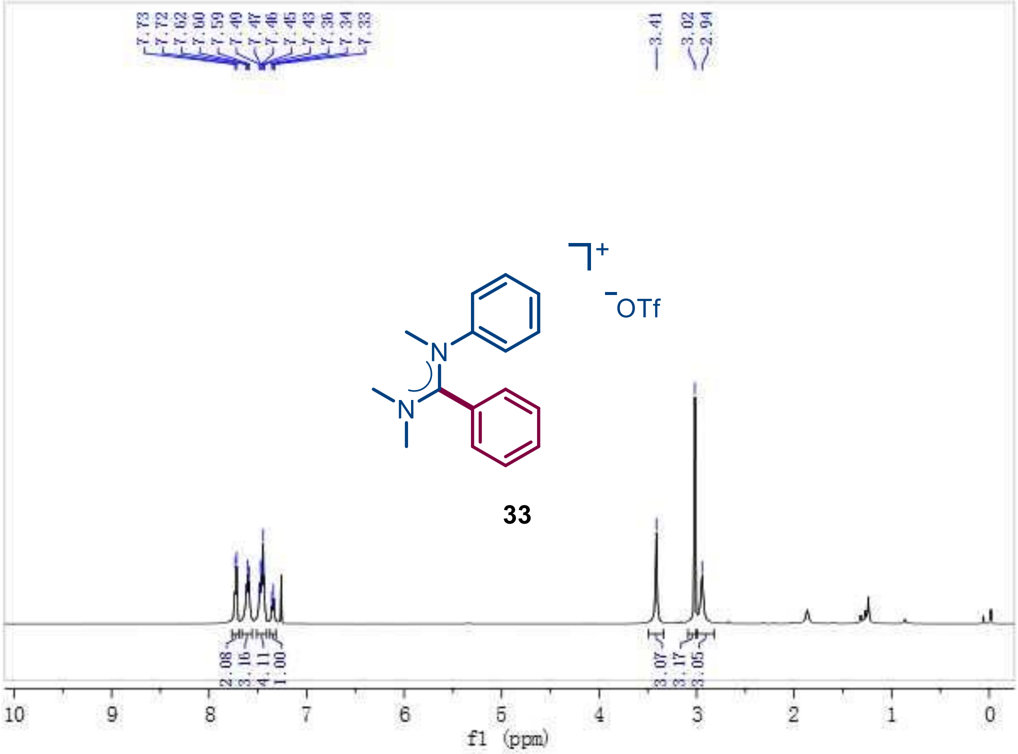

Supplementary Figure 103. ^{1}H NMR Spectrum of Compound **33**

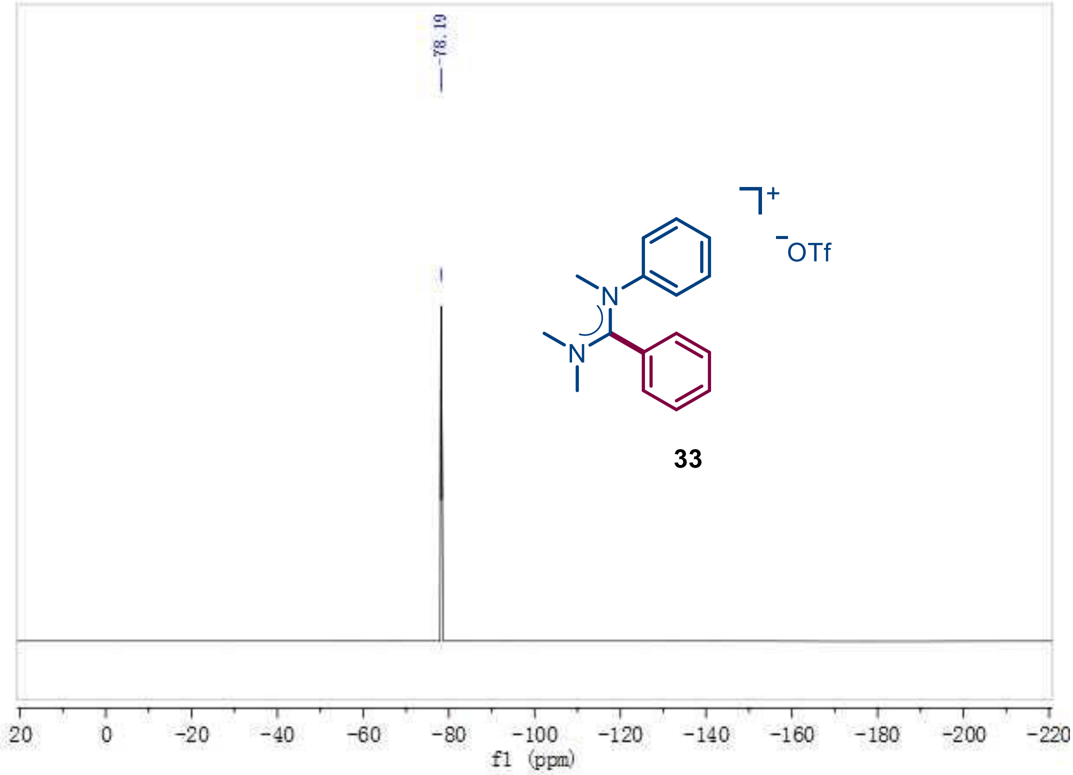

Supplementary Figure 104. ^{19}F NMR Spectrum of Compound **33**

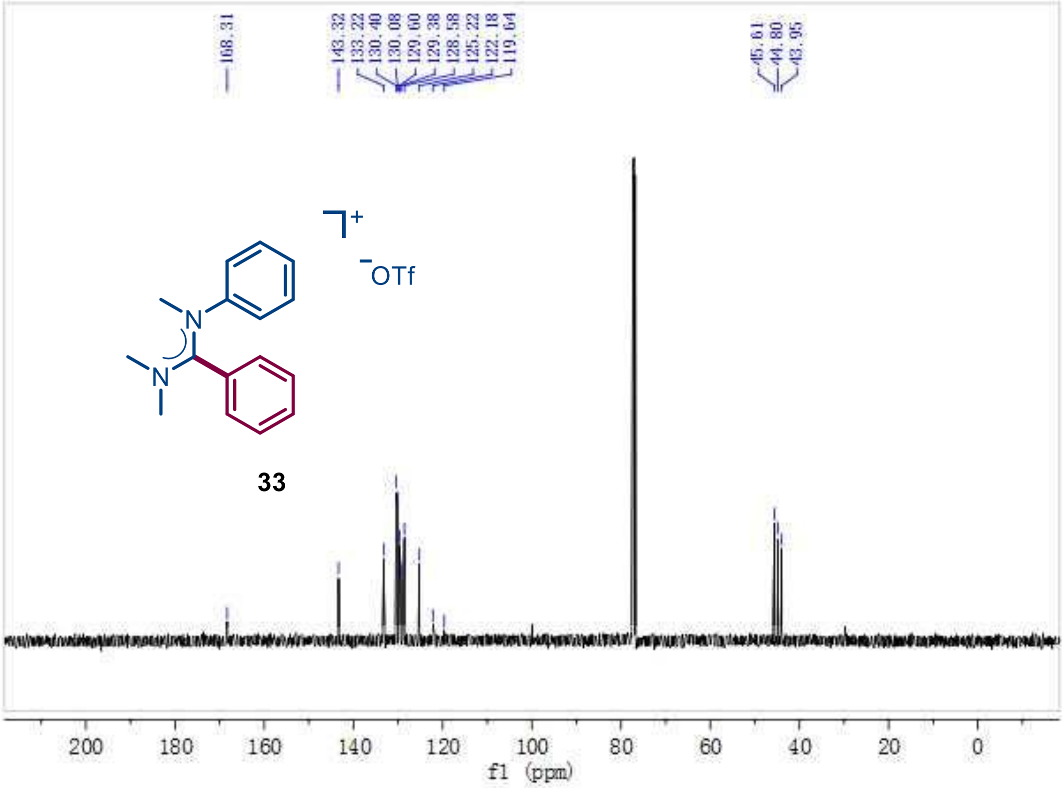

Supplementary Figure 105. ^{13}C NMR Spectrum of Compound **33**

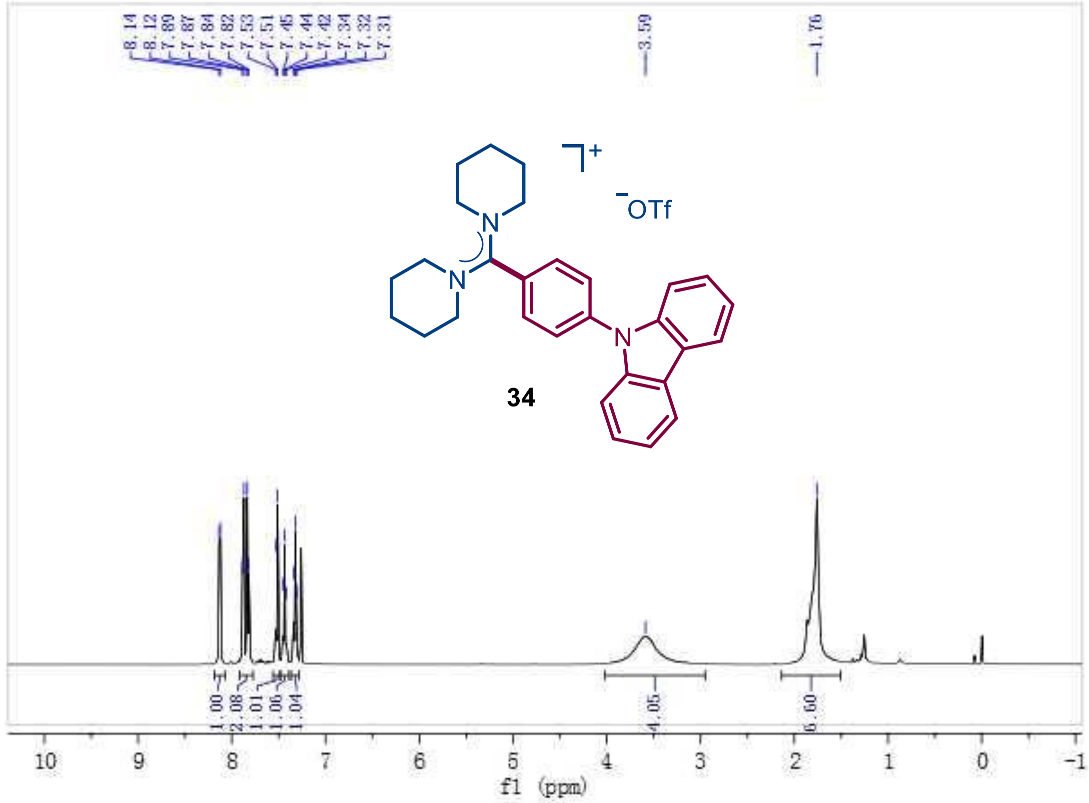

Supplementary Figure 106. ^{1}H NMR Spectrum of Compound **34**

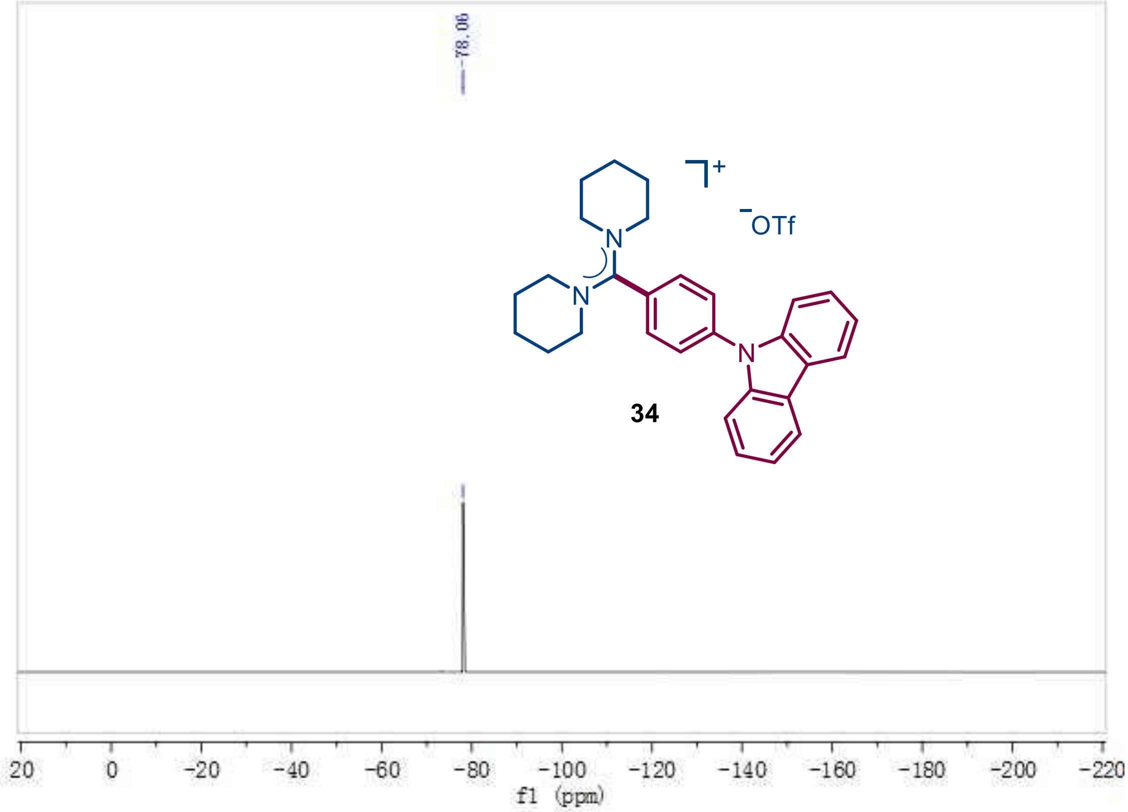

Supplementary Figure 107. ^{19}F NMR Spectrum of Compound **34**

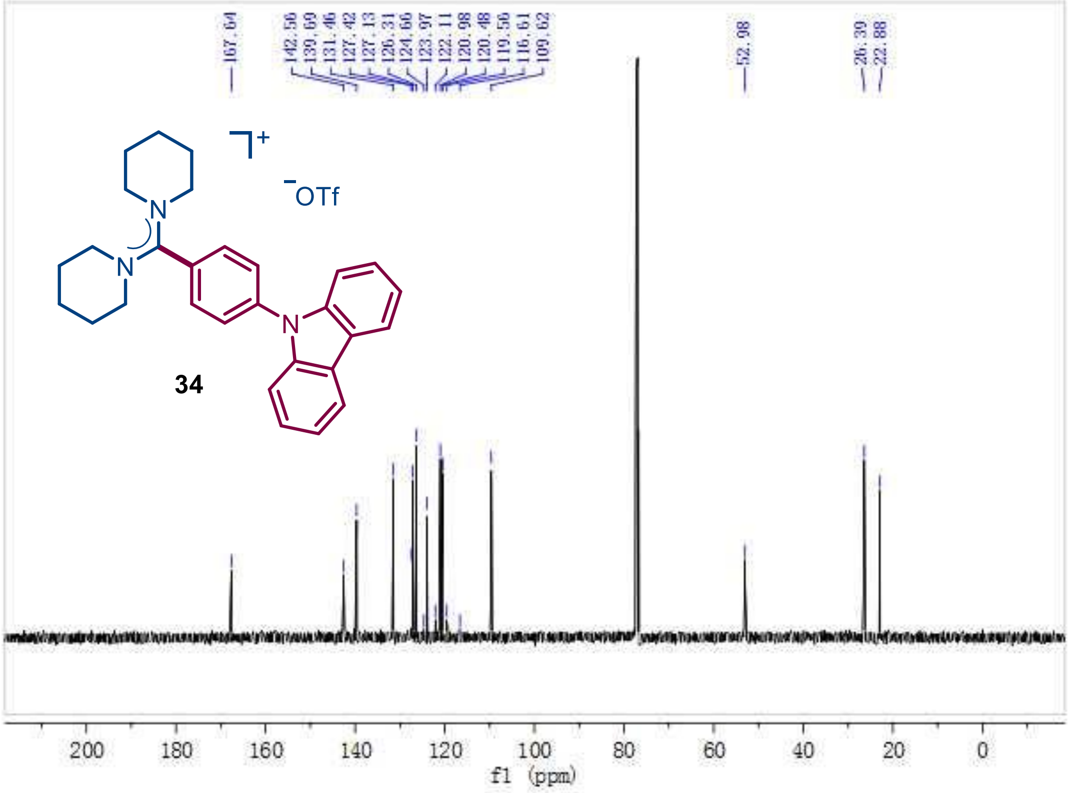

Supplementary Figure 108. ^{13}C NMR Spectrum of Compound **34**

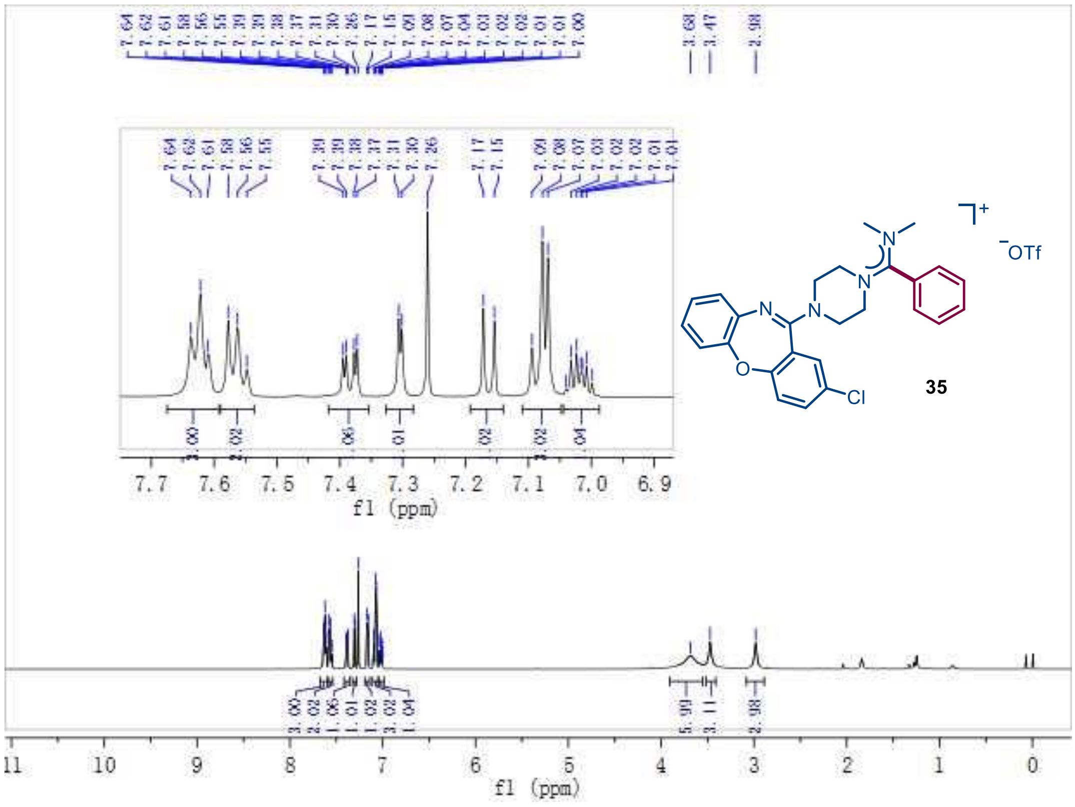

Supplementary Figure 109. ^{1}H NMR Spectrum of Compound **35**

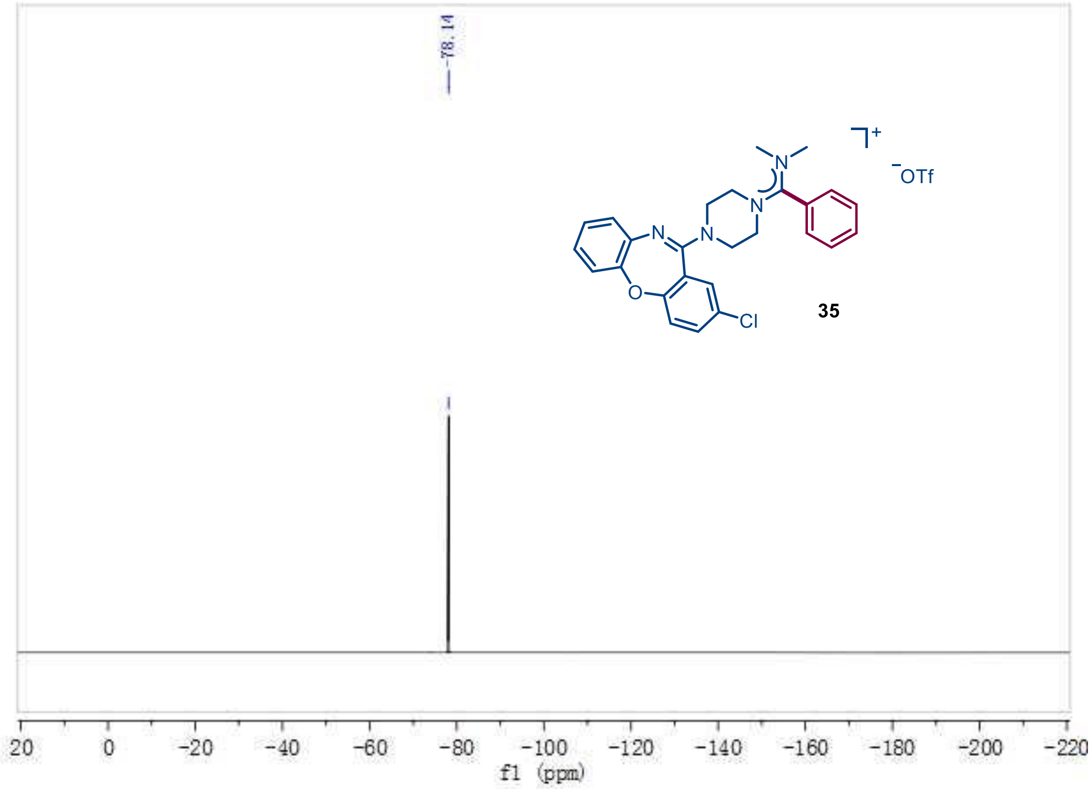

Supplementary Figure 110. ^{19}F NMR Spectrum of Compound **35**

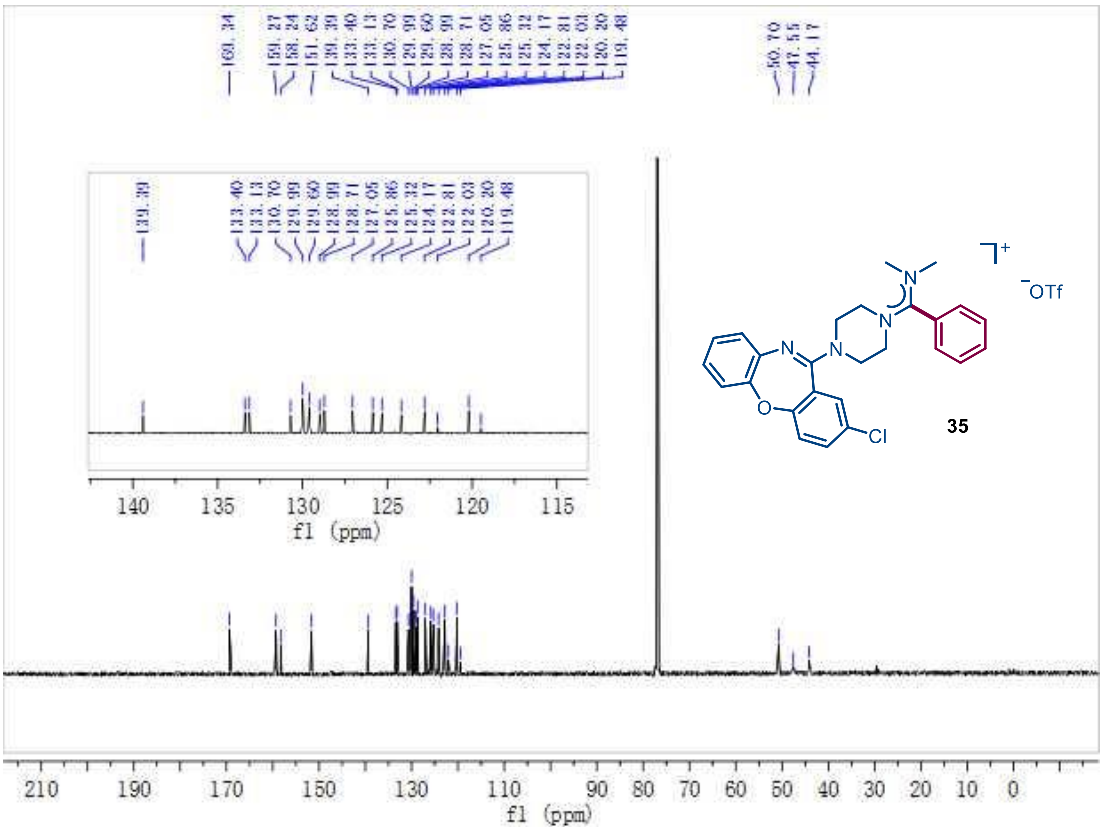

Supplementary Figure 111. ^{13}C NMR Spectrum of Compound **35**

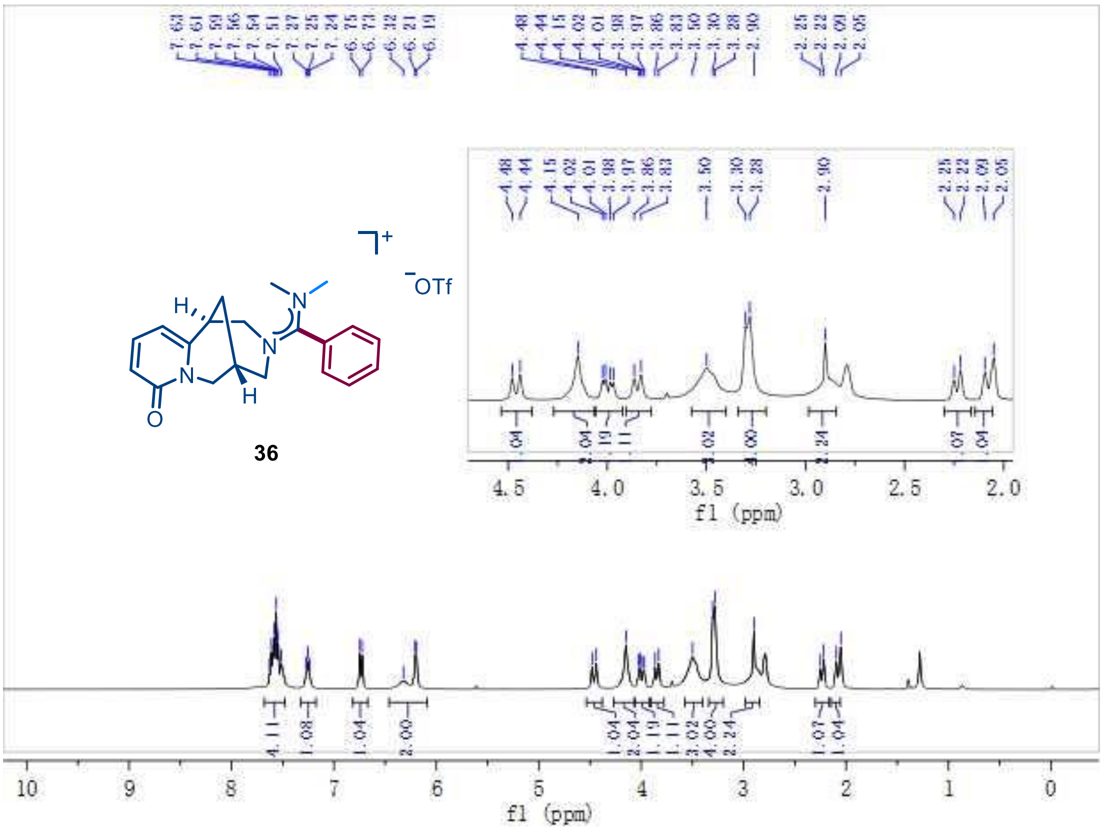

Supplementary Figure 112. ^{1}H NMR Spectrum of Compound **36**

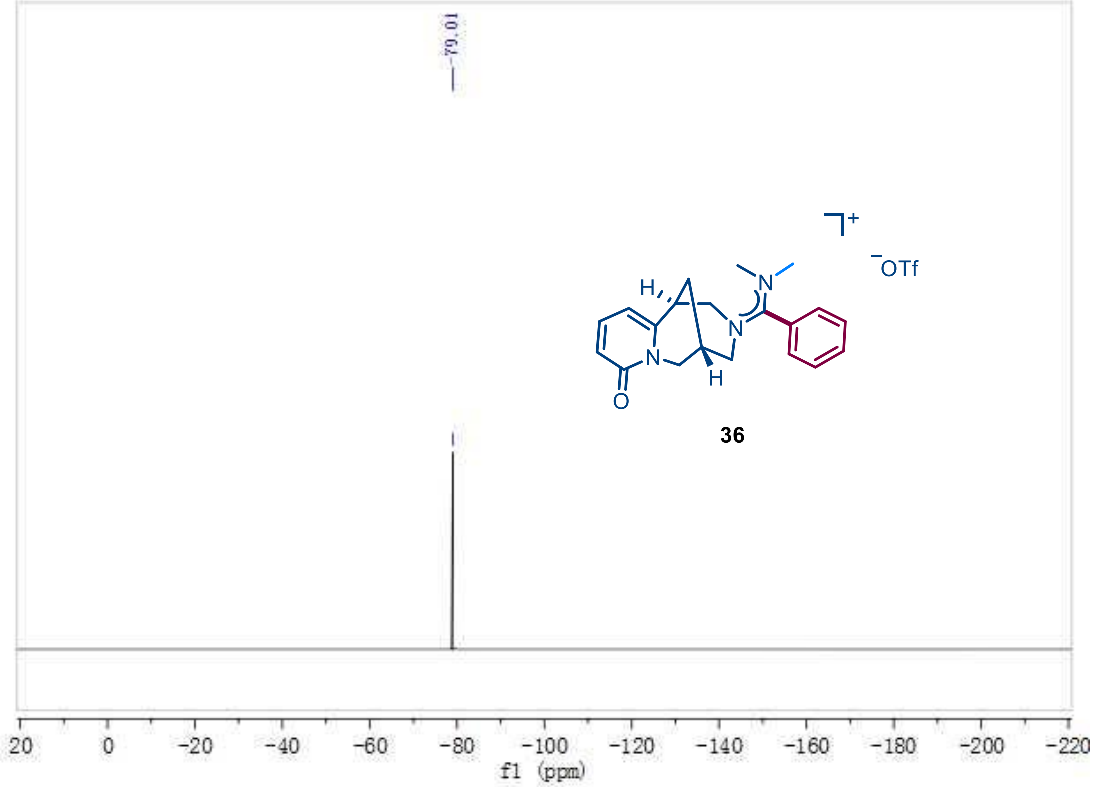

Supplementary Figure 113. ^{19}F NMR Spectrum of Compound **36**

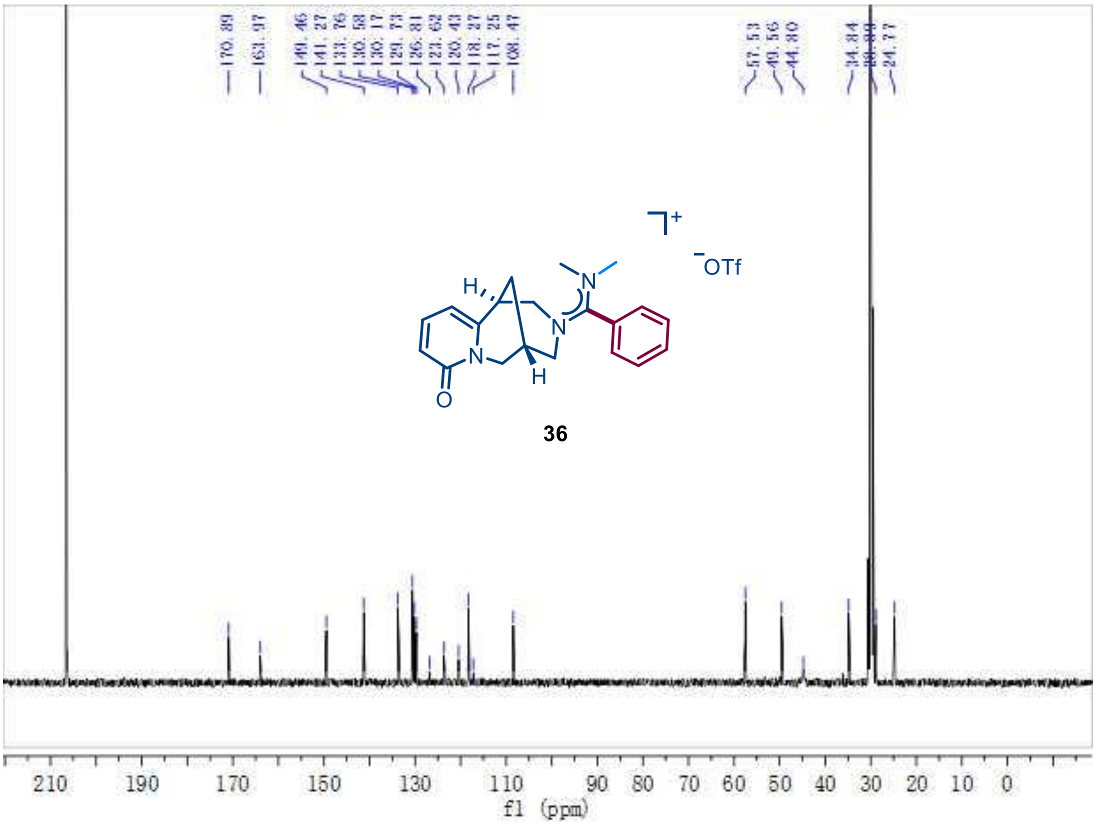

Supplementary Figure 114. ^{13}C NMR Spectrum of Compound **36**

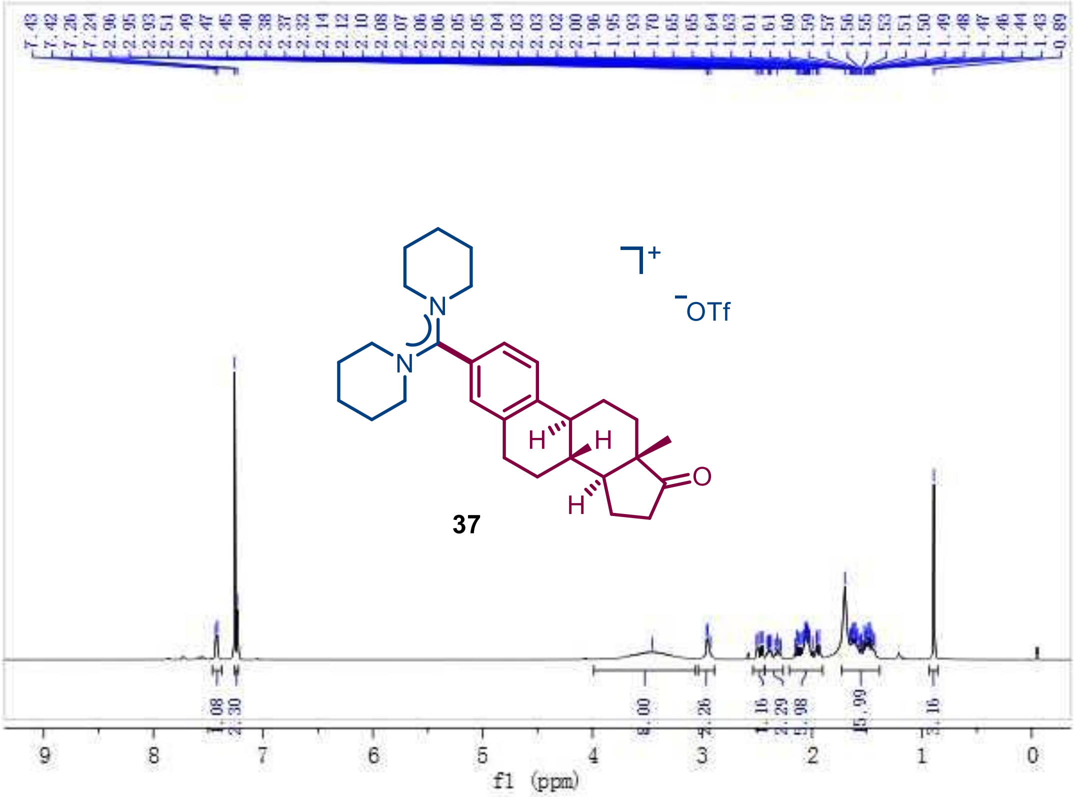

Supplementary Figure 115. ^{1}H NMR Spectrum of Compound **37**

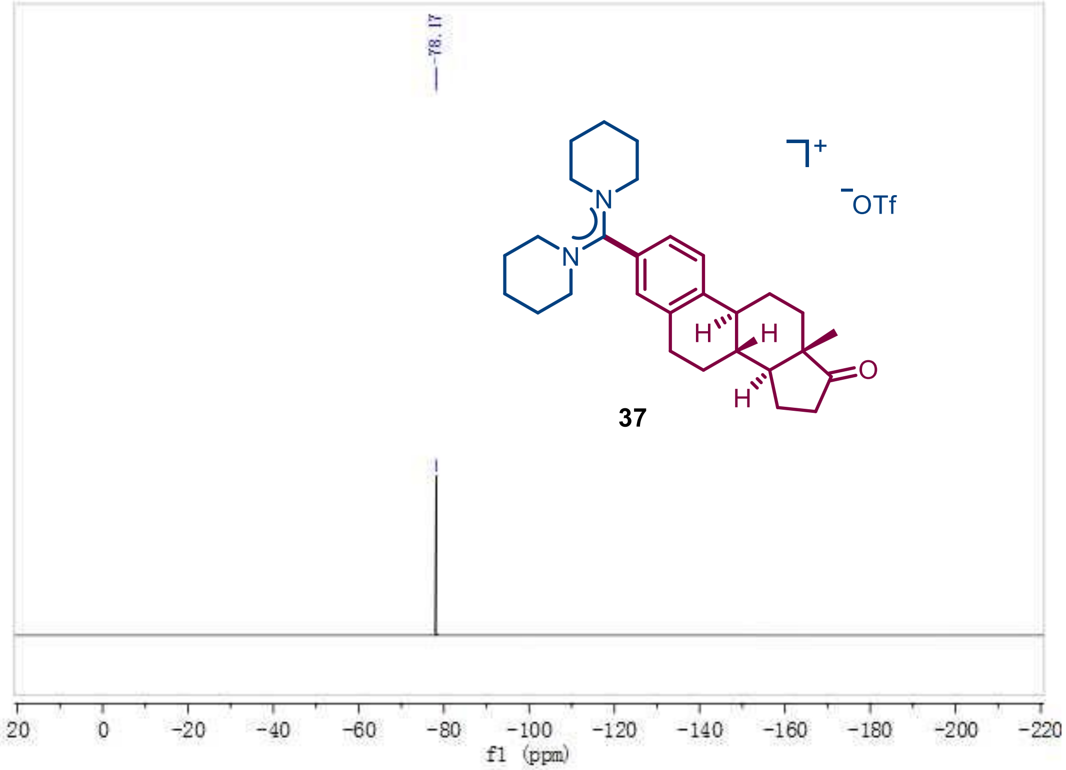

Supplementary Figure 116. ^{19}F NMR Spectrum of Compound **37**

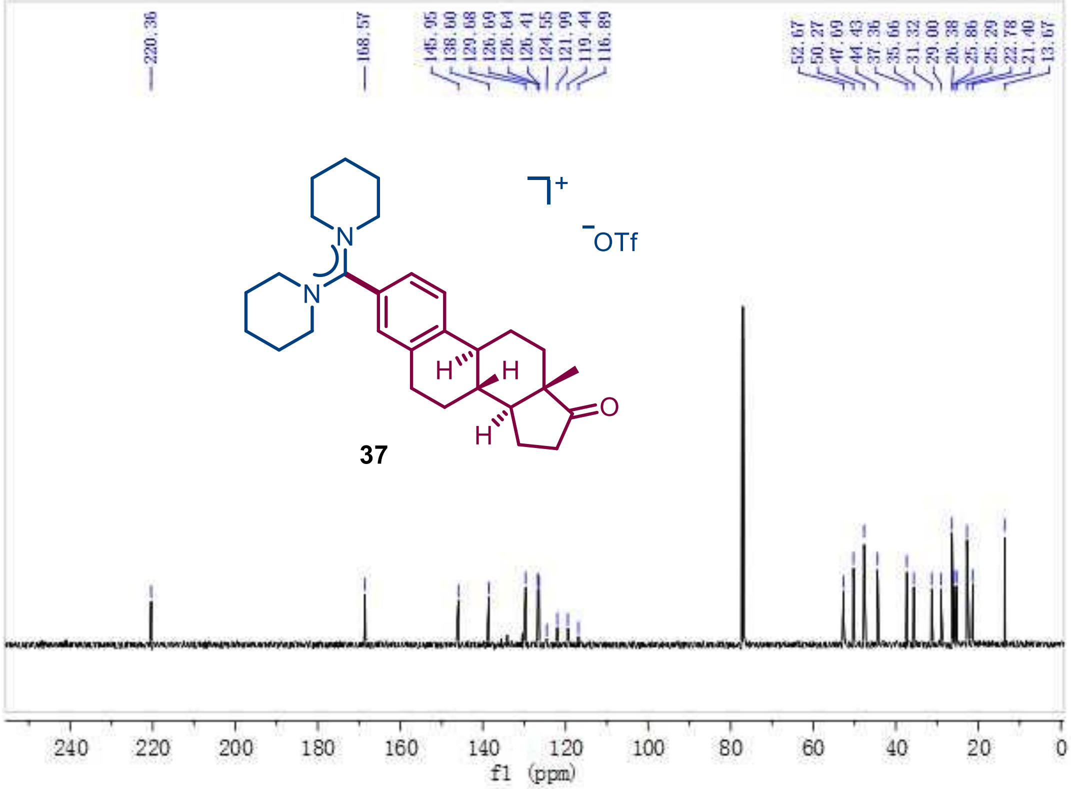

Supplementary Figure 117. ^{13}C NMR Spectrum of Compound **37**

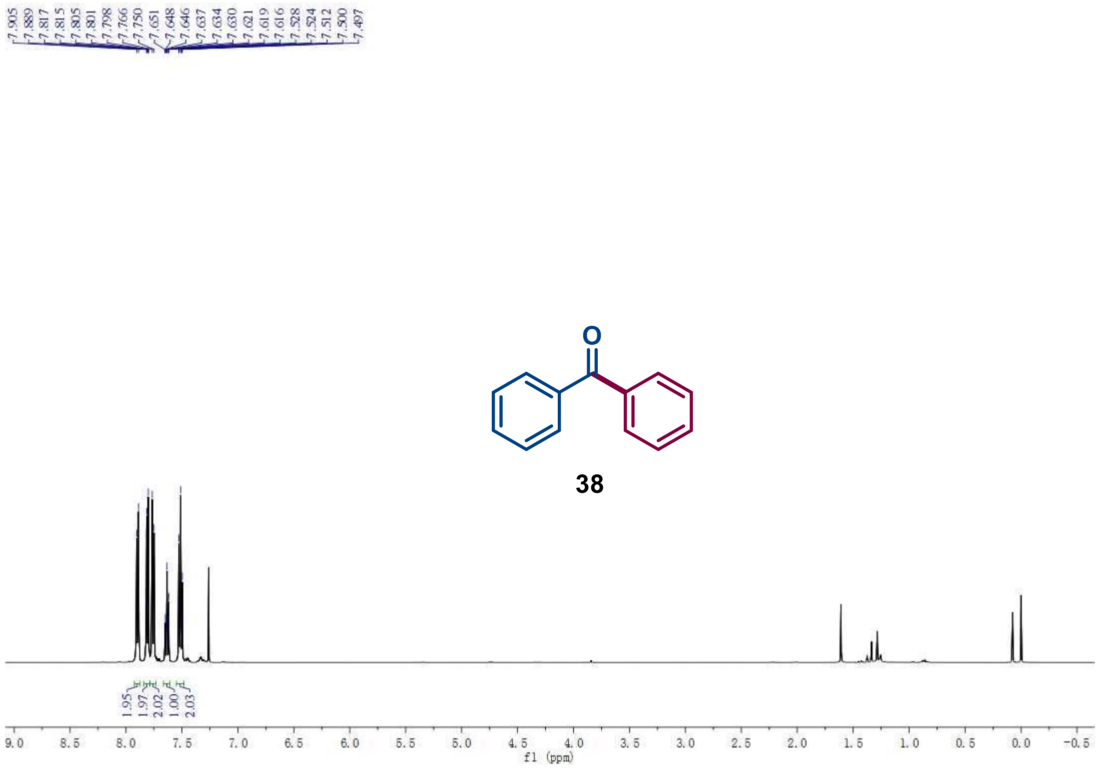

Supplementary Figure 118. ^{1}H NMR Spectrum of Compound **38**

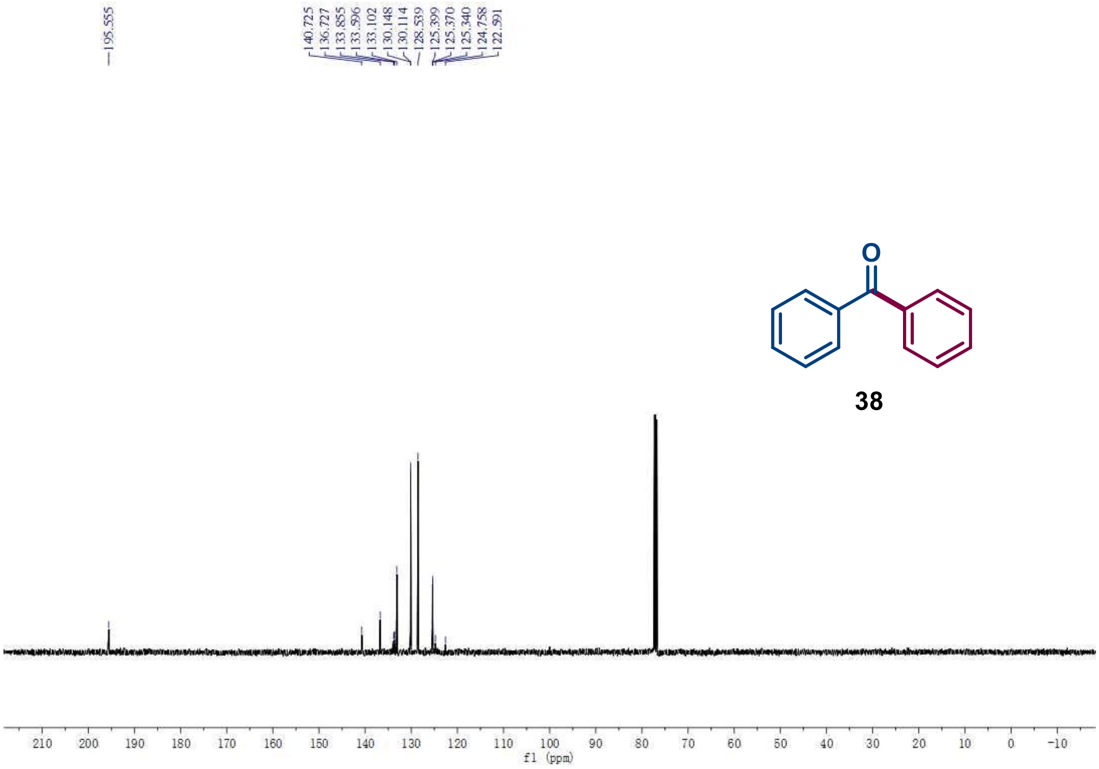

Supplementary Figure 119. ^{13}C NMR Spectrum of Compound **38**

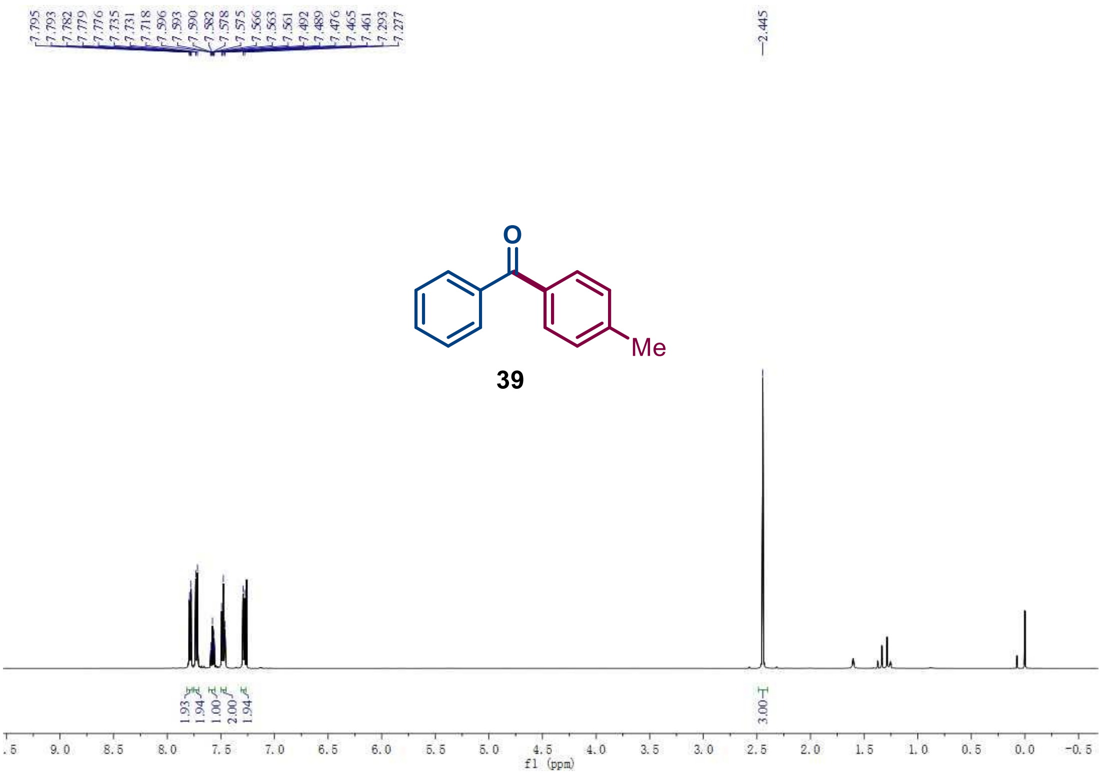

Supplementary Figure 120. ^{1}H NMR Spectrum of Compound **39**

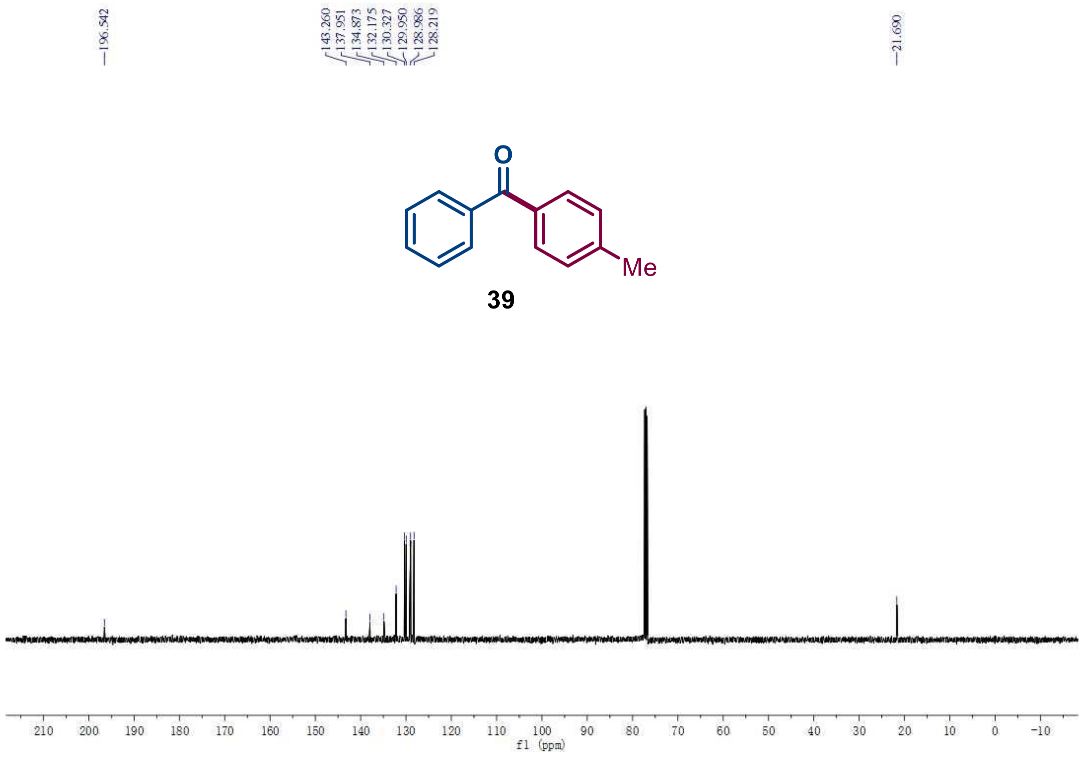

Supplementary Figure 121. ^{13}C NMR Spectrum of Compound **39**

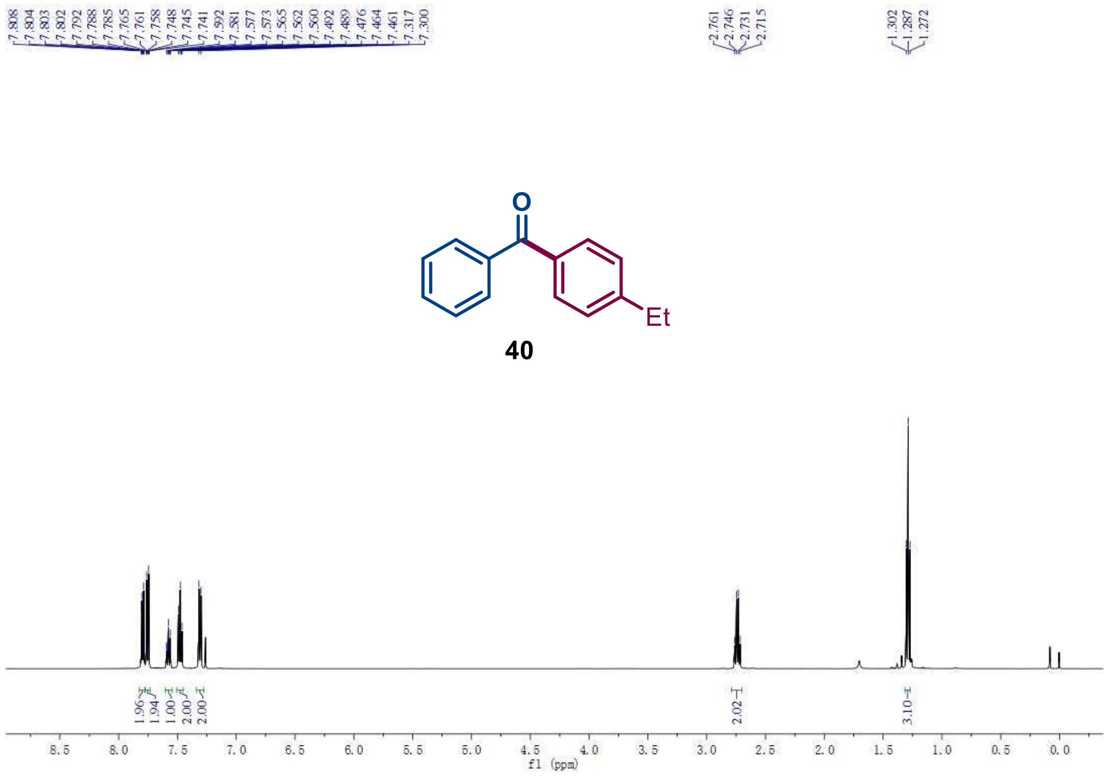

Supplementary Figure 122. ^{1}H NMR Spectrum of Compound **40**

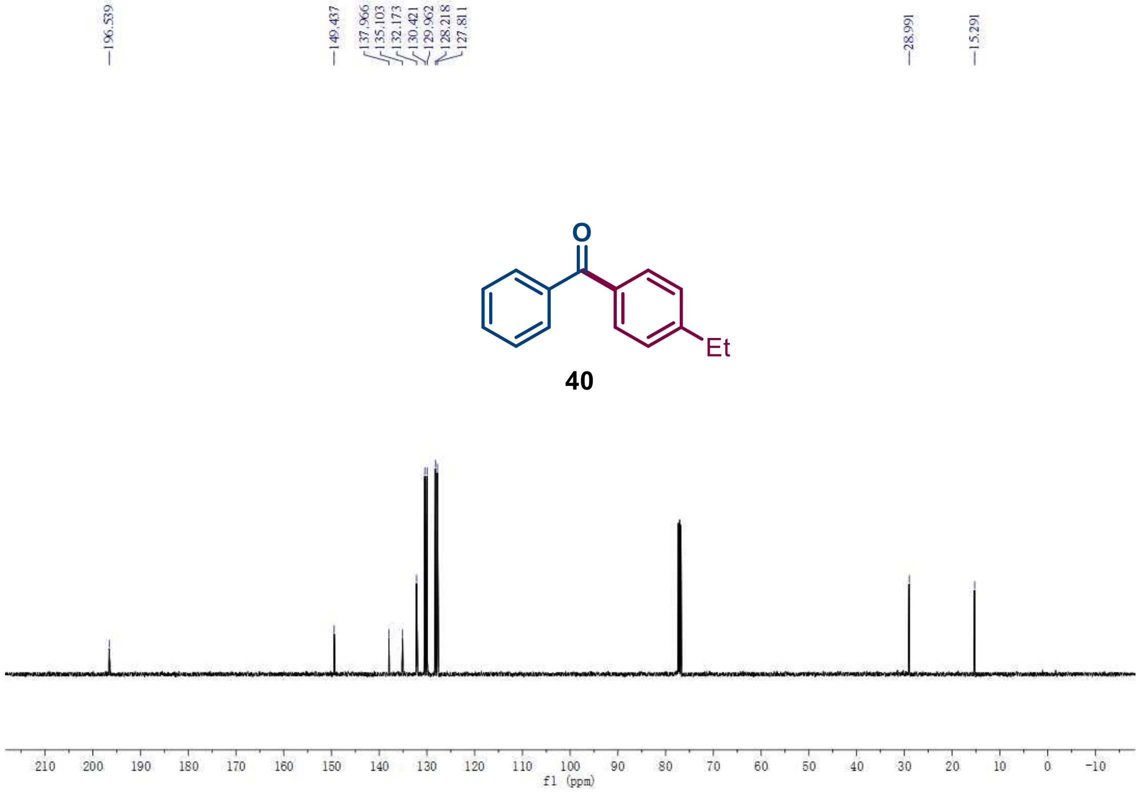

Supplementary Figure 123. ^{13}C NMR Spectrum of Compound **40**

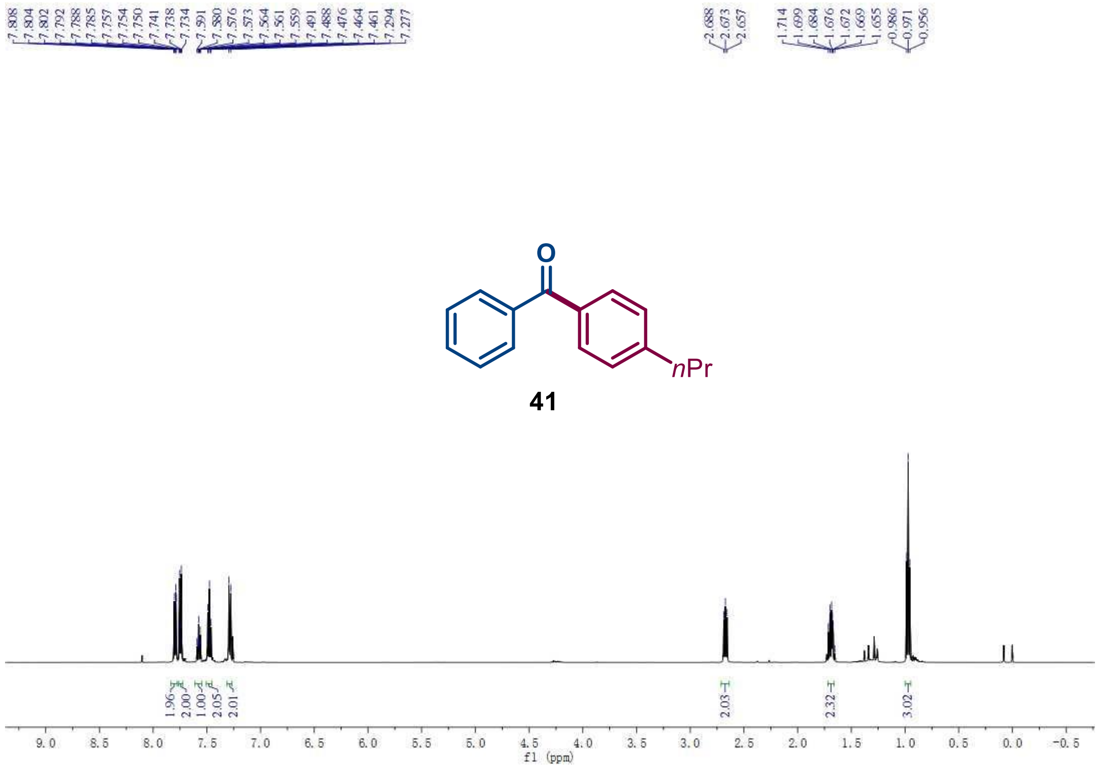

Supplementary Figure 124. ^{1}H NMR Spectrum of Compound **41**

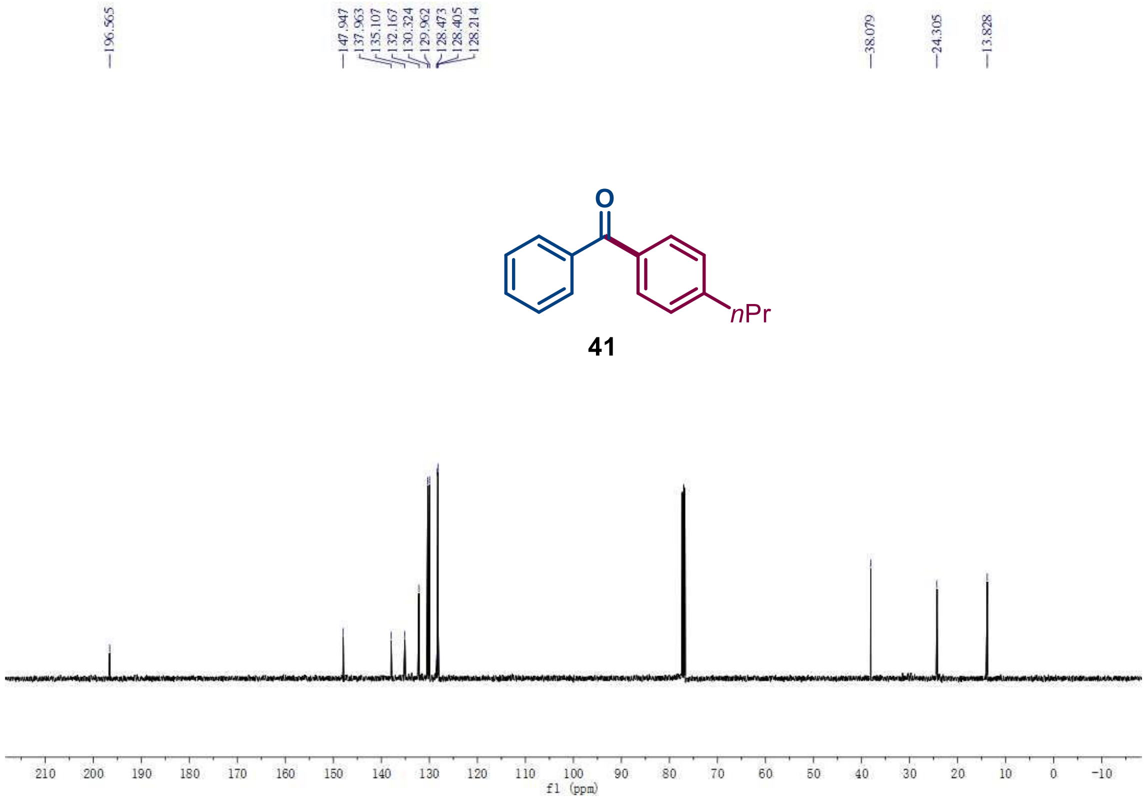

Supplementary Figure 125. ^{13}C NMR Spectrum of Compound **41**

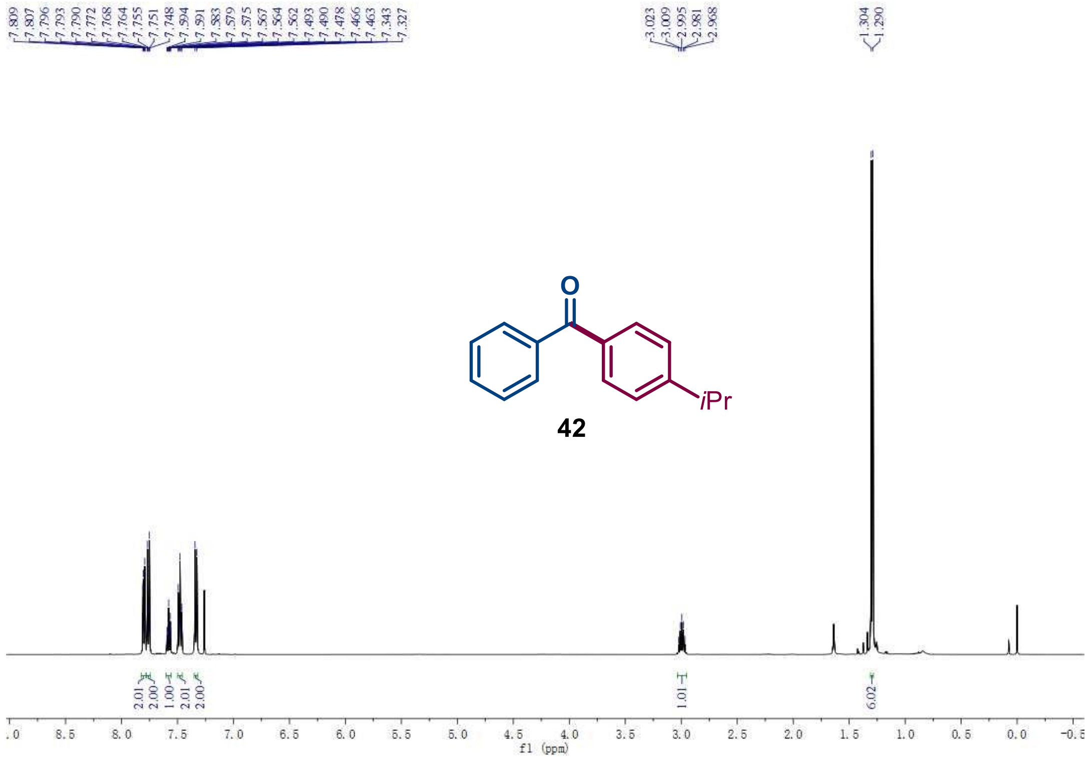

Supplementary Figure 126. ^{1}H NMR Spectrum of Compound **42**

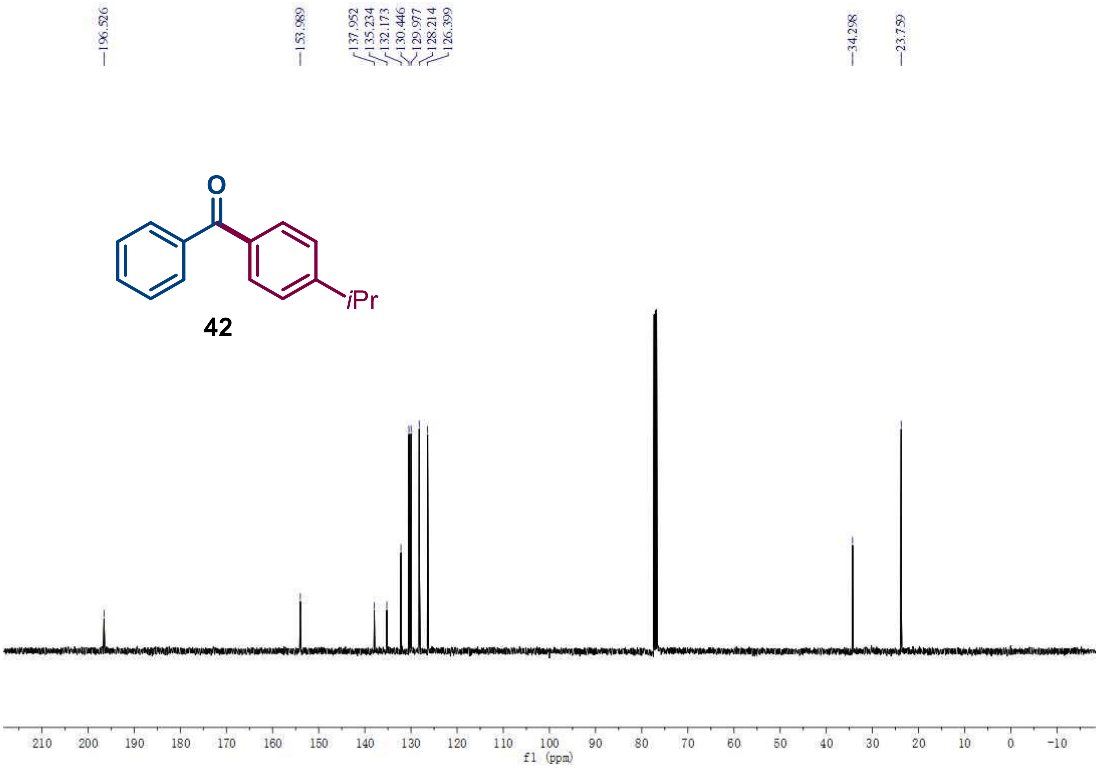

Supplementary Figure 127. ^{13}C NMR Spectrum of Compound **42**

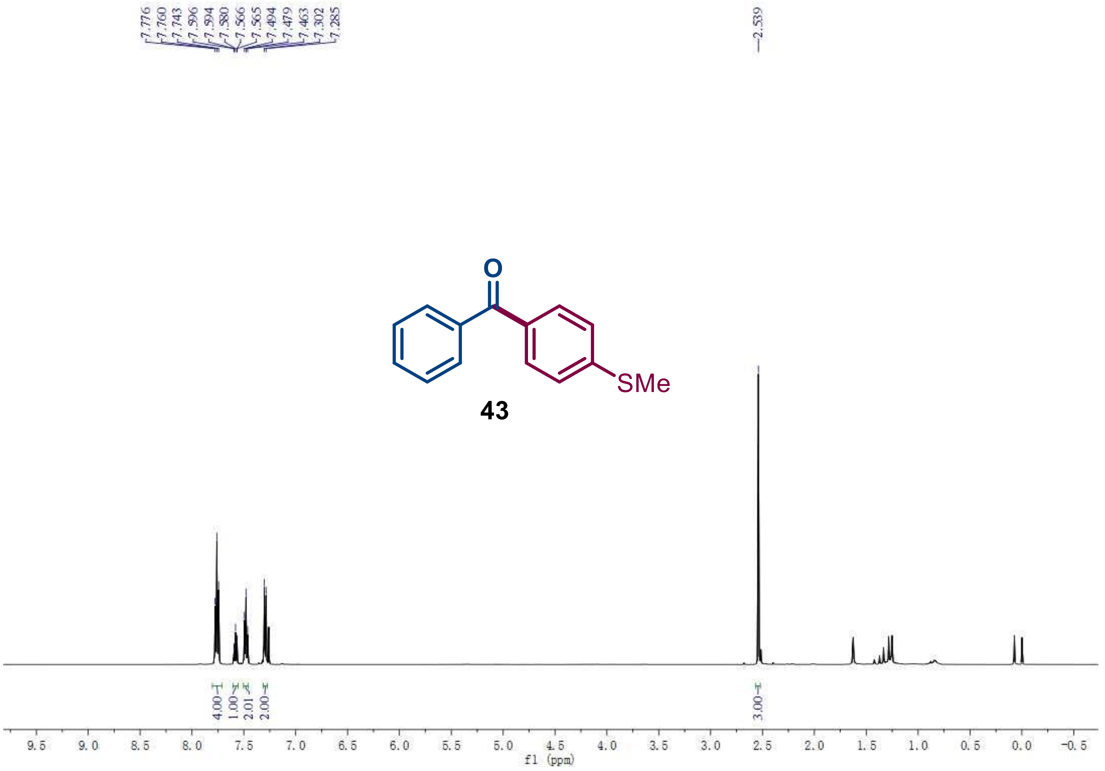

Supplementary Figure 128. ^{1}H NMR Spectrum of Compound **43**

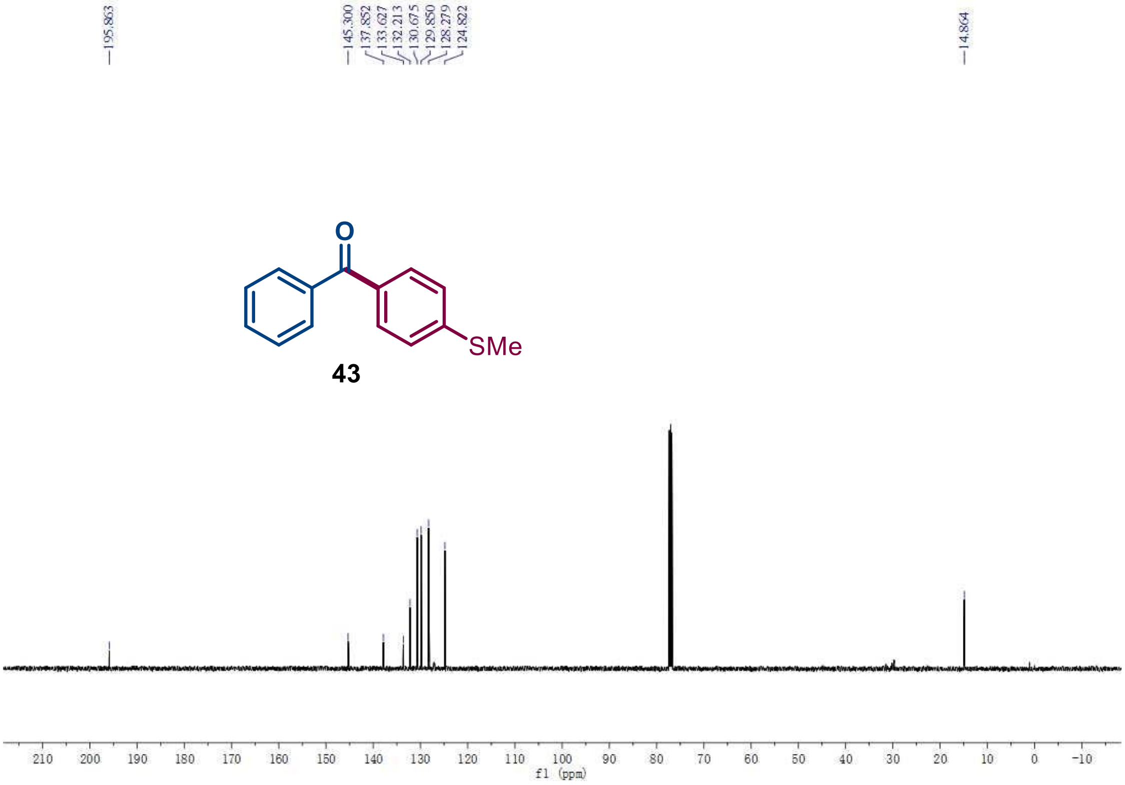

Supplementary Figure 129. ^{13}C NMR Spectrum of Compound **43**

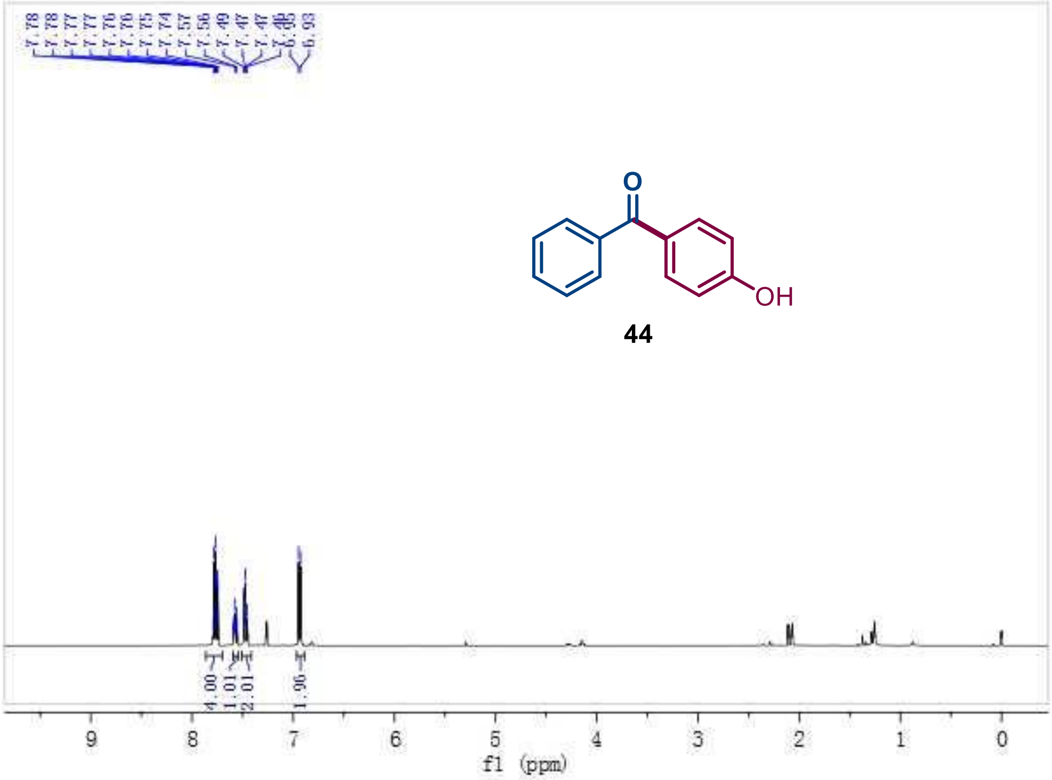

Supplementary Figure 130. ^{1}H NMR Spectrum of Compound **44**

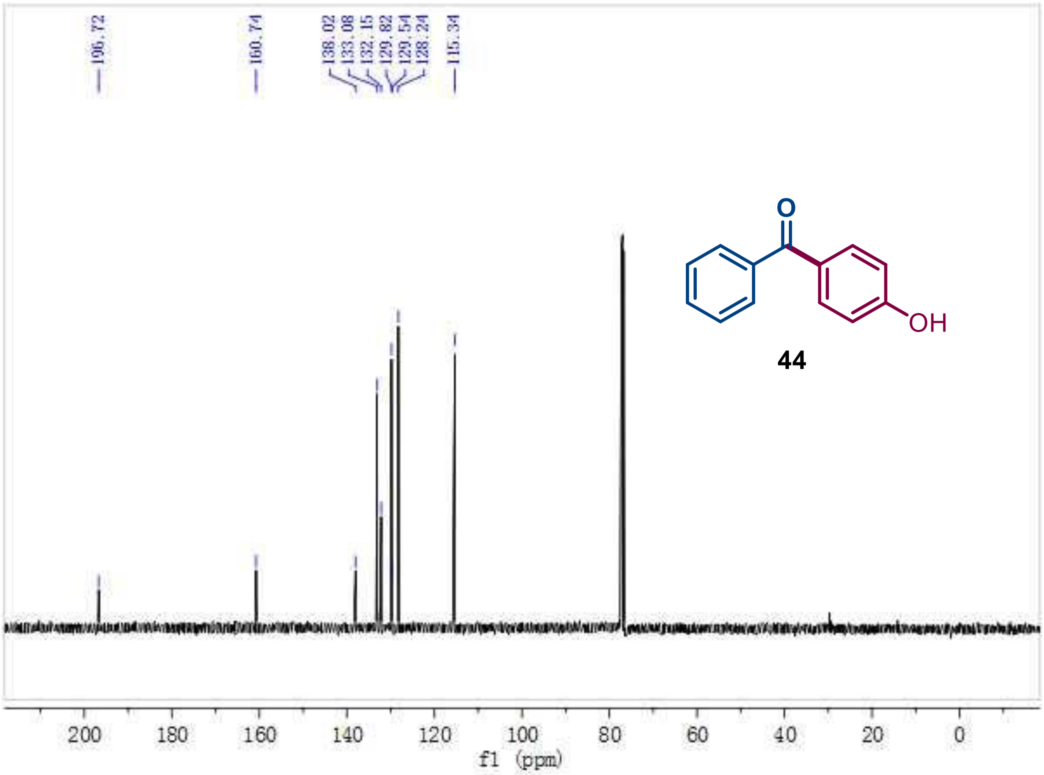

Supplementary Figure 131. ^{13}C NMR Spectrum of Compound **44**

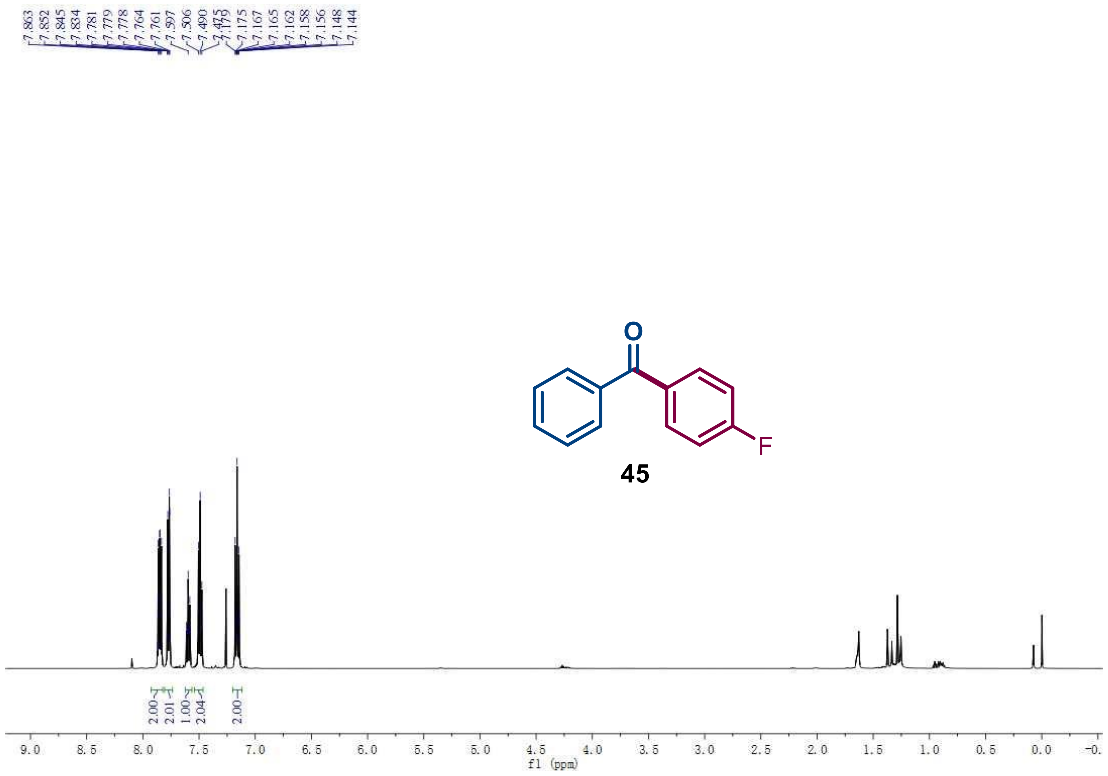

Supplementary Figure 132. ^{1}H NMR Spectrum of Compound **45**

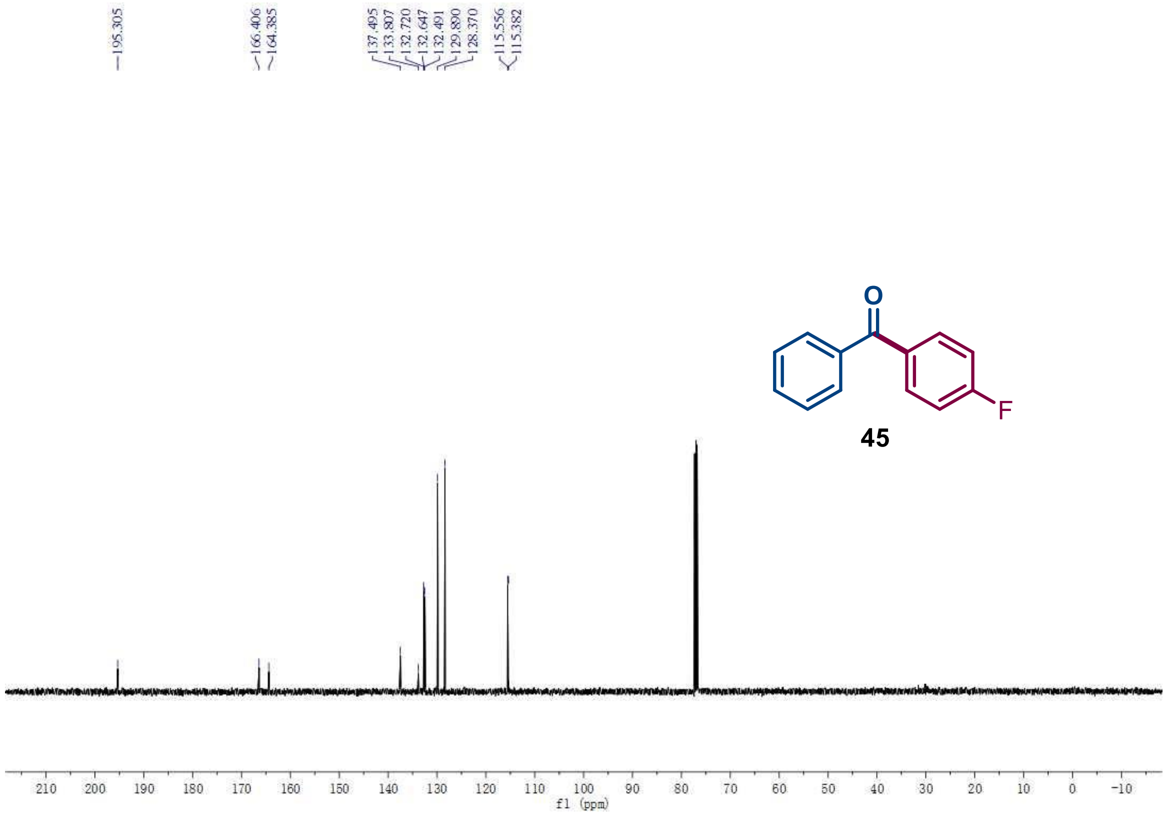

Supplementary Figure 133. ^{13}C NMR Spectrum of Compound **45**

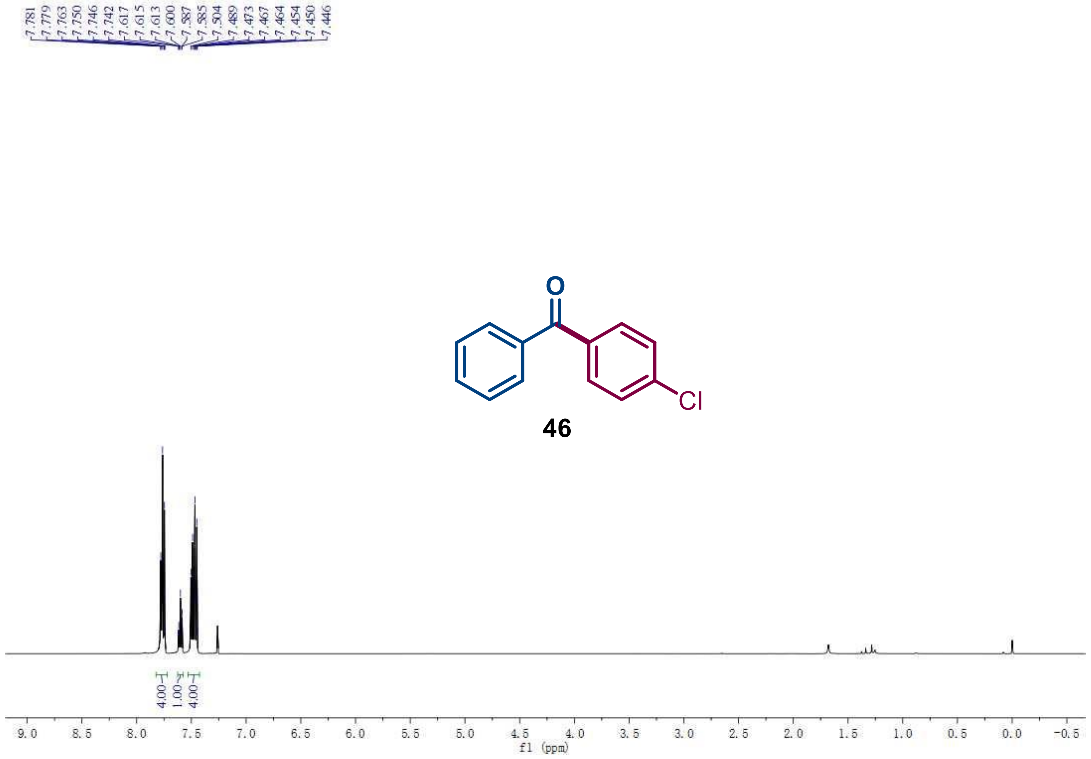

Supplementary Figure 134. ^{1}H NMR Spectrum of Compound **46**

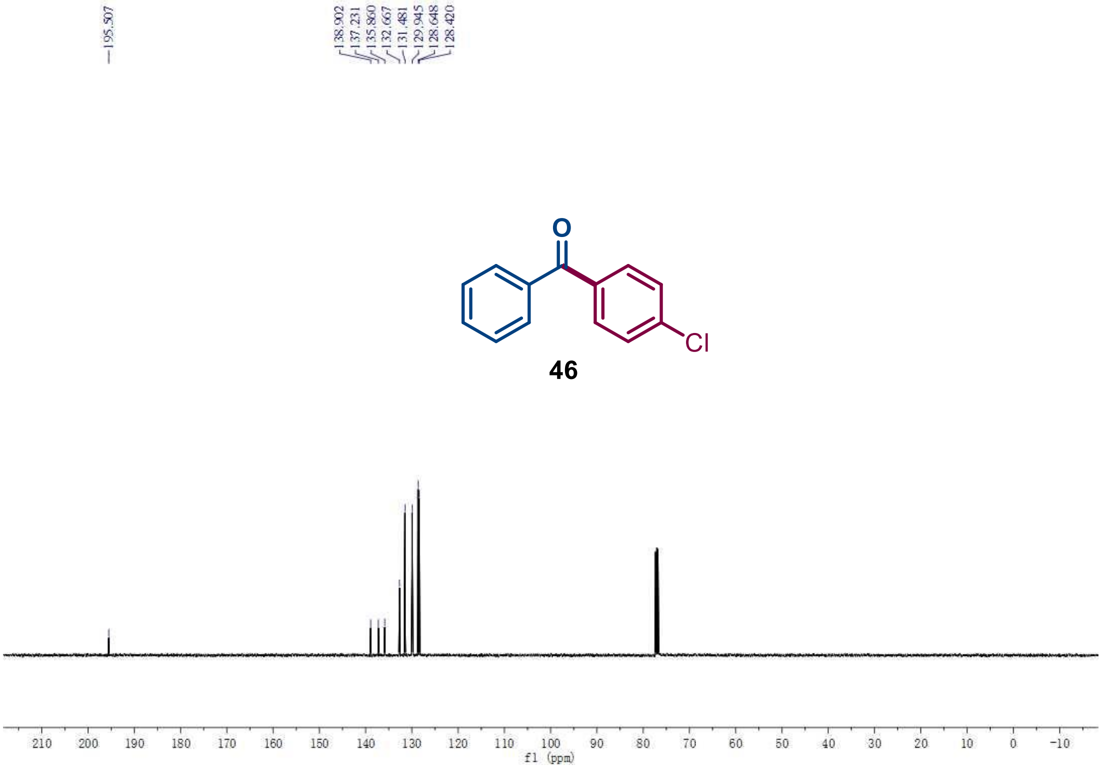

Supplementary Figure 135. ^{13}C NMR Spectrum of Compound **46**

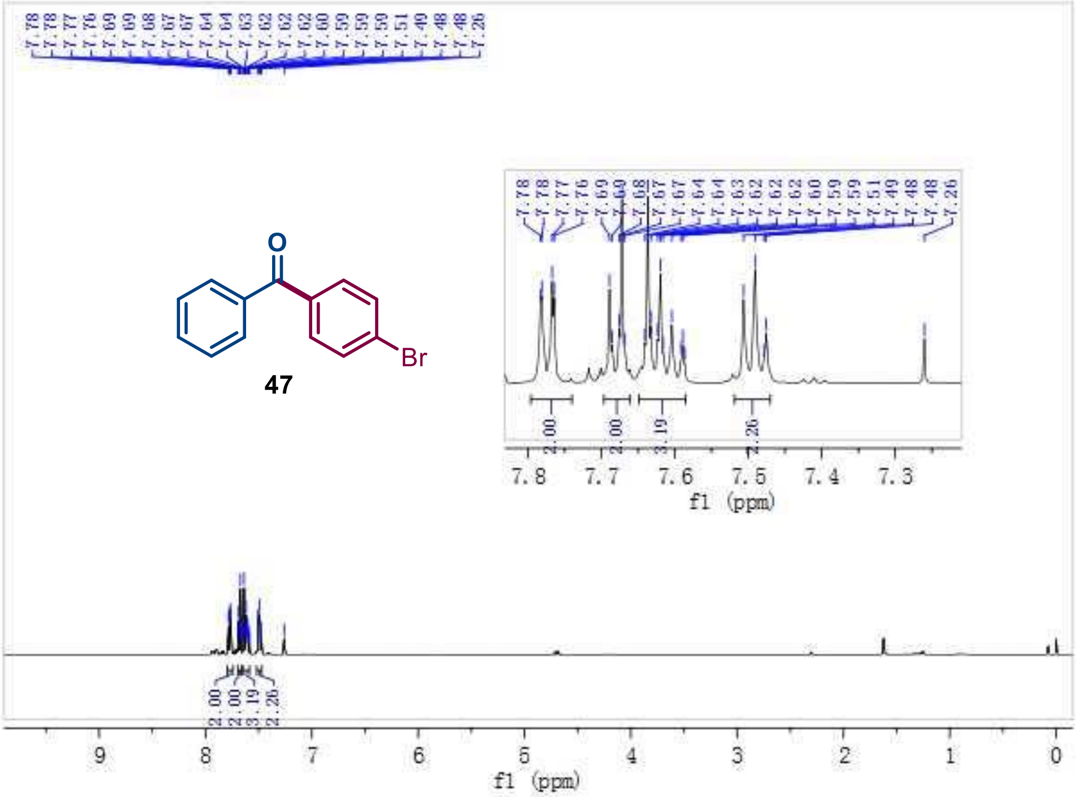

Supplementary Figure 136. ^{1}H NMR Spectrum of Compound **47**

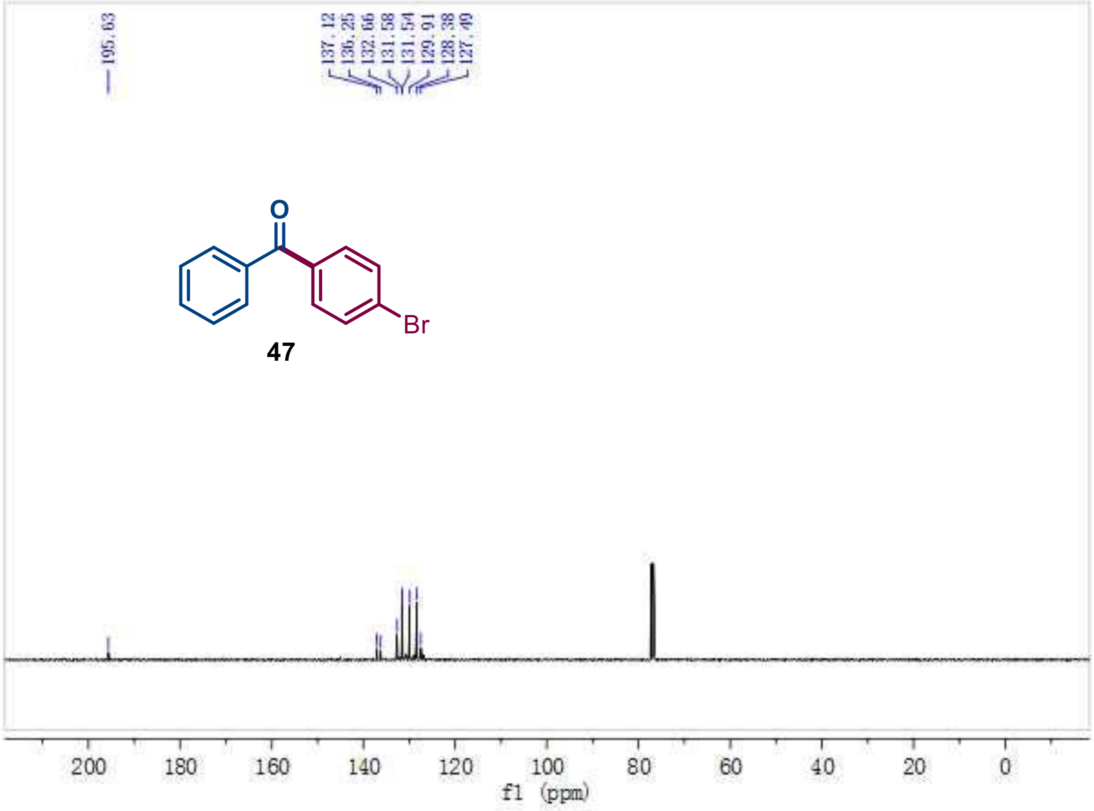

Supplementary Figure 137. ^{13}C NMR Spectrum of Compound **47**

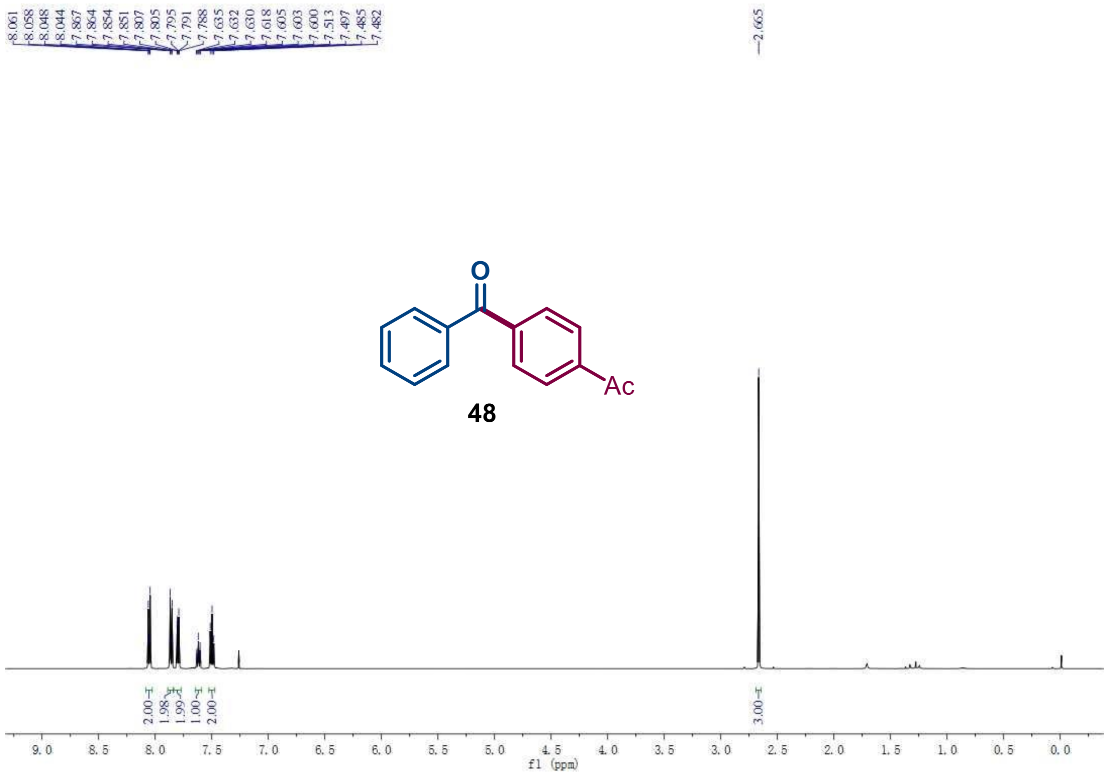

Supplementary Figure 138. ^{1}H NMR Spectrum of Compound **48**

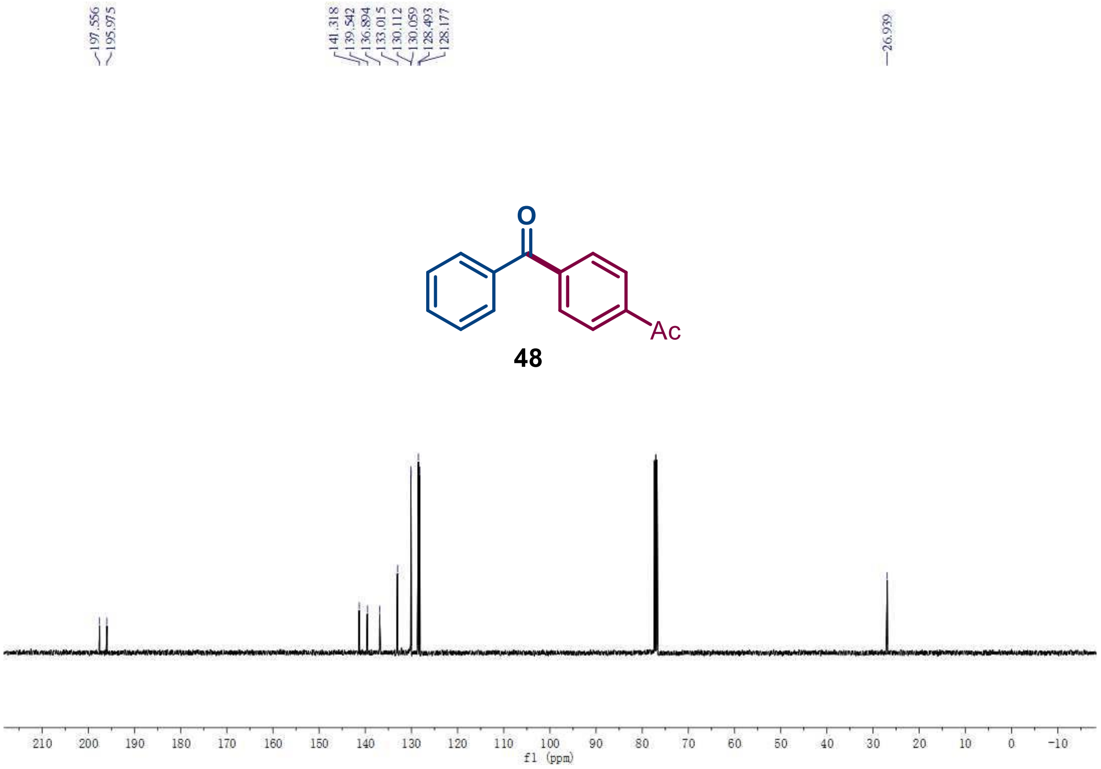

Supplementary Figure 139. ^{13}C NMR Spectrum of Compound **48**

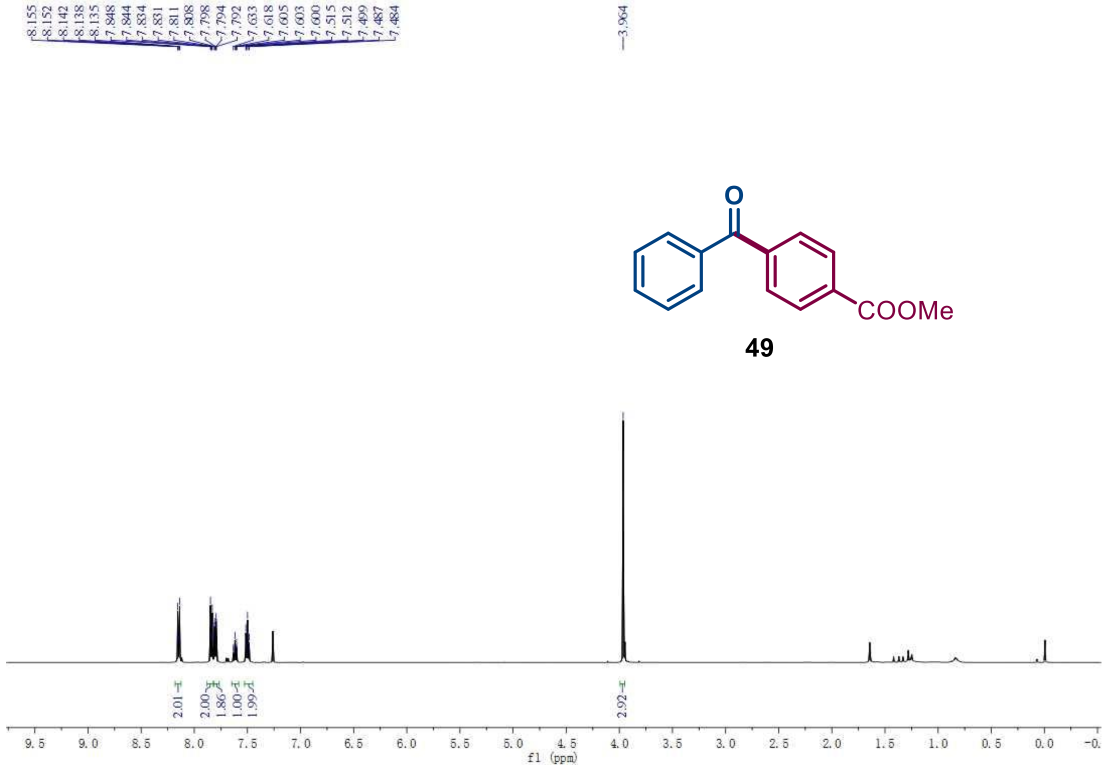

Supplementary Figure 140. ^{1}H NMR Spectrum of Compound **49**

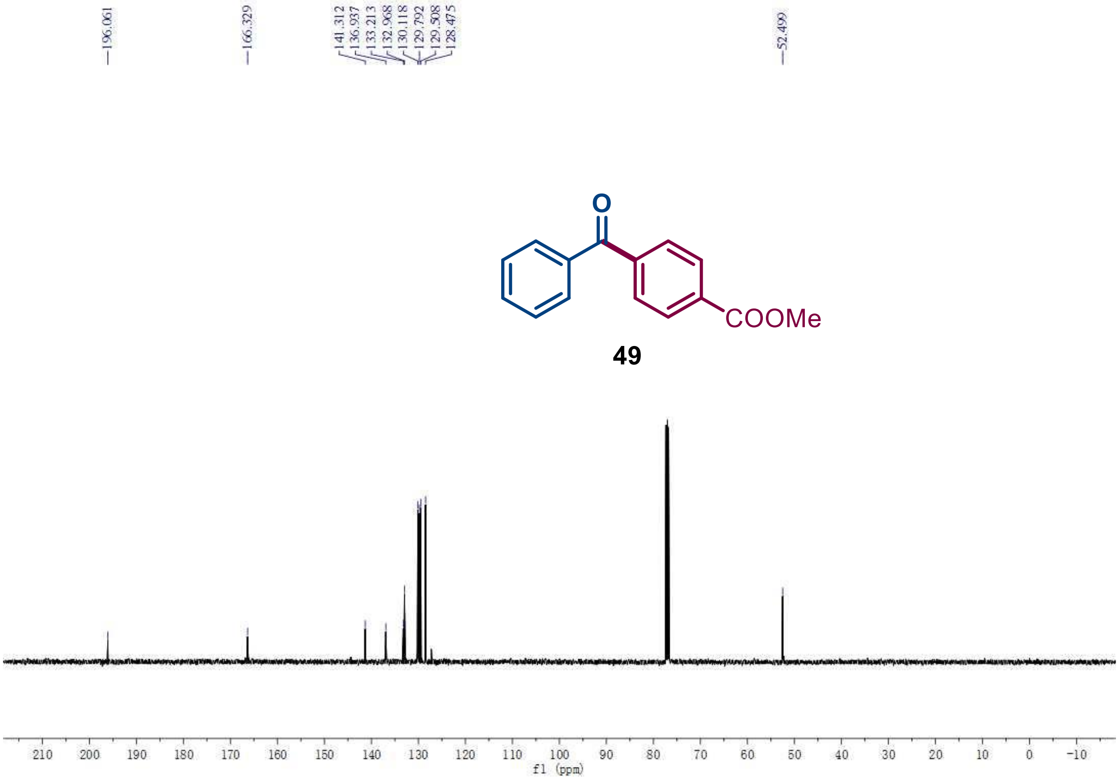

Supplementary Figure 141. ^{13}C NMR Spectrum of Compound **49**

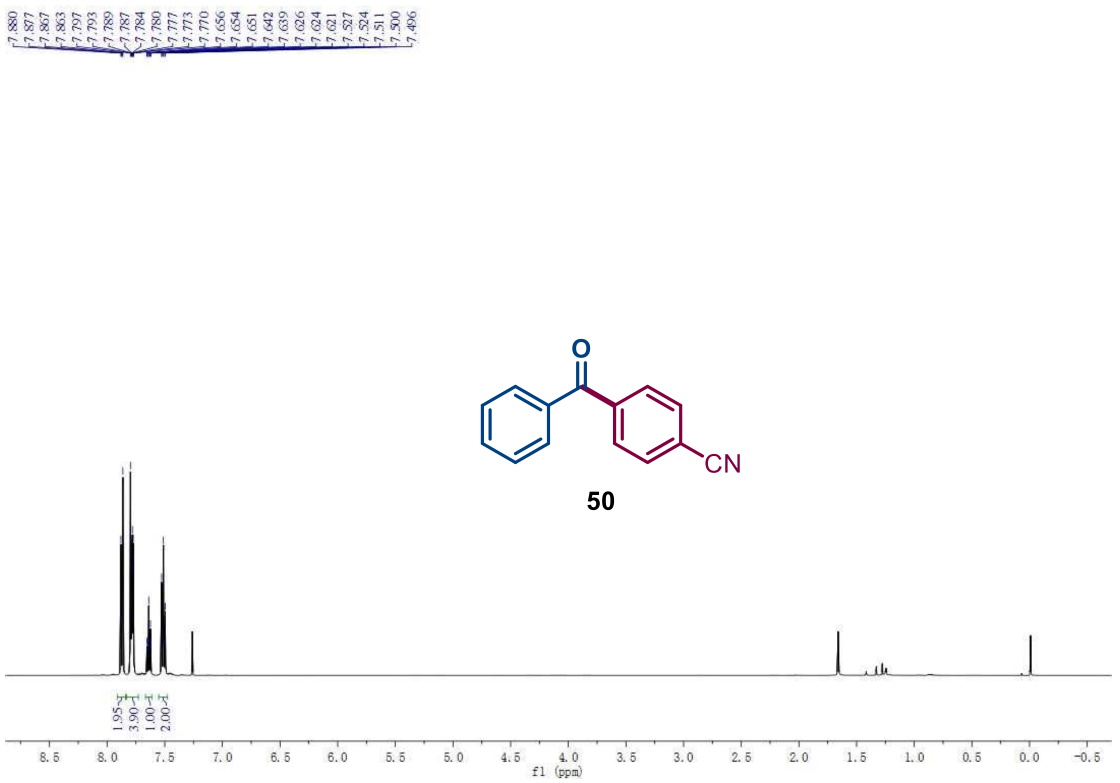

Supplementary Figure 142. ^{1}H NMR Spectrum of Compound **50**

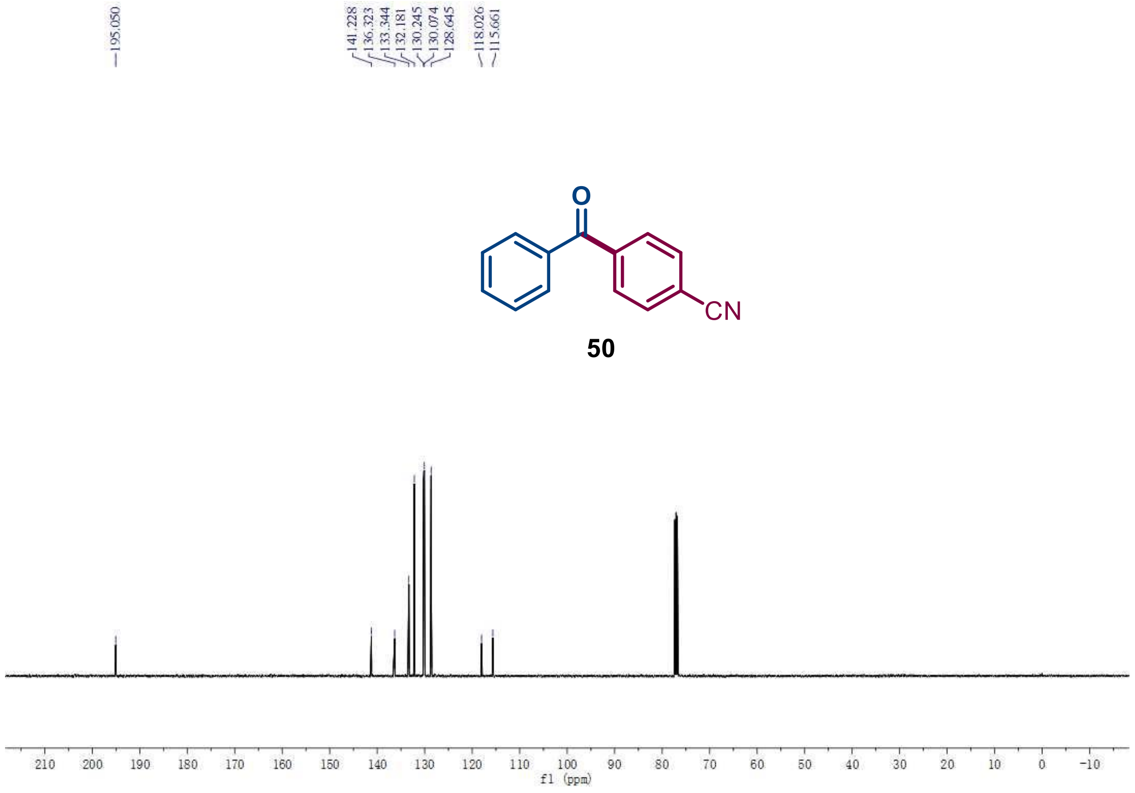

Supplementary Figure 143. ^{13}C NMR Spectrum of Compound **50**

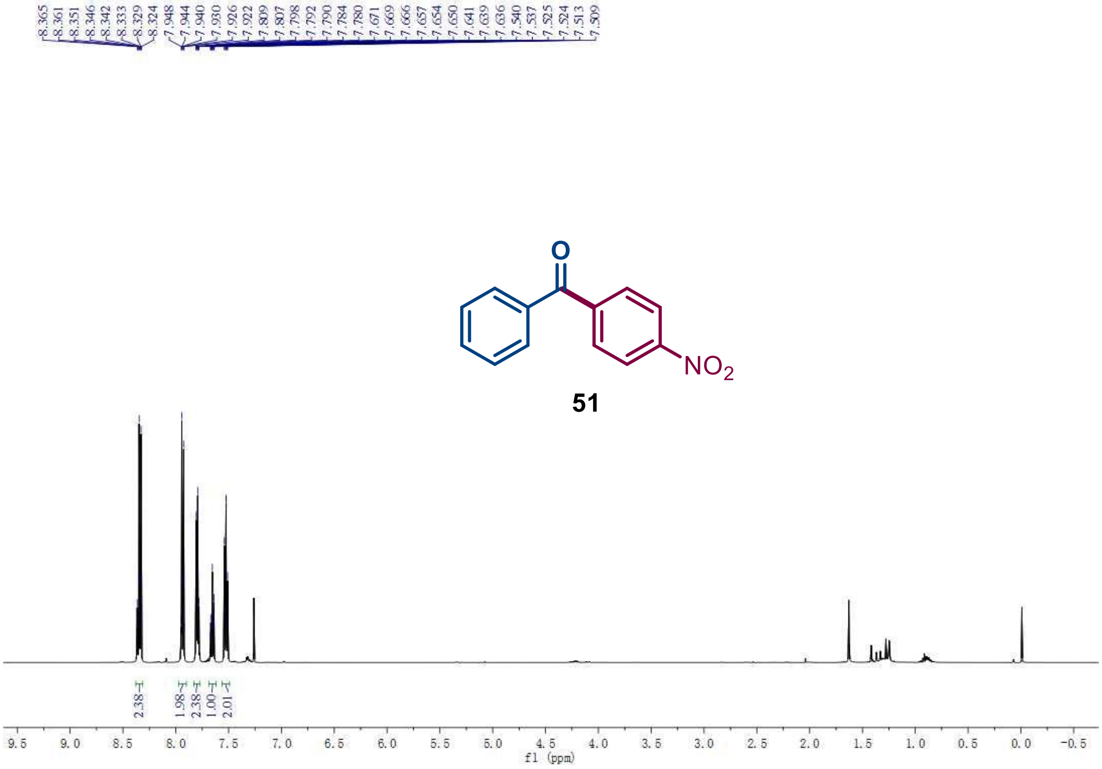

Supplementary Figure 144. ^{1}H NMR Spectrum of Compound **51**

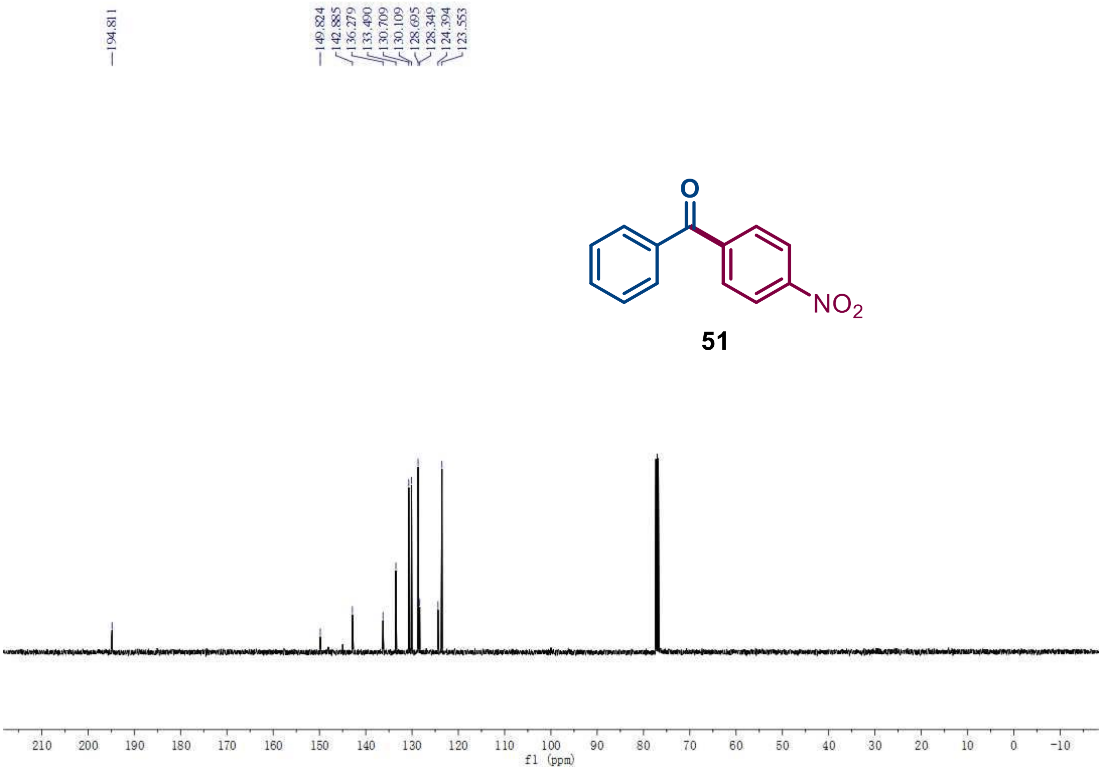

Supplementary Figure 145. ^{13}C NMR Spectrum of Compound **51**

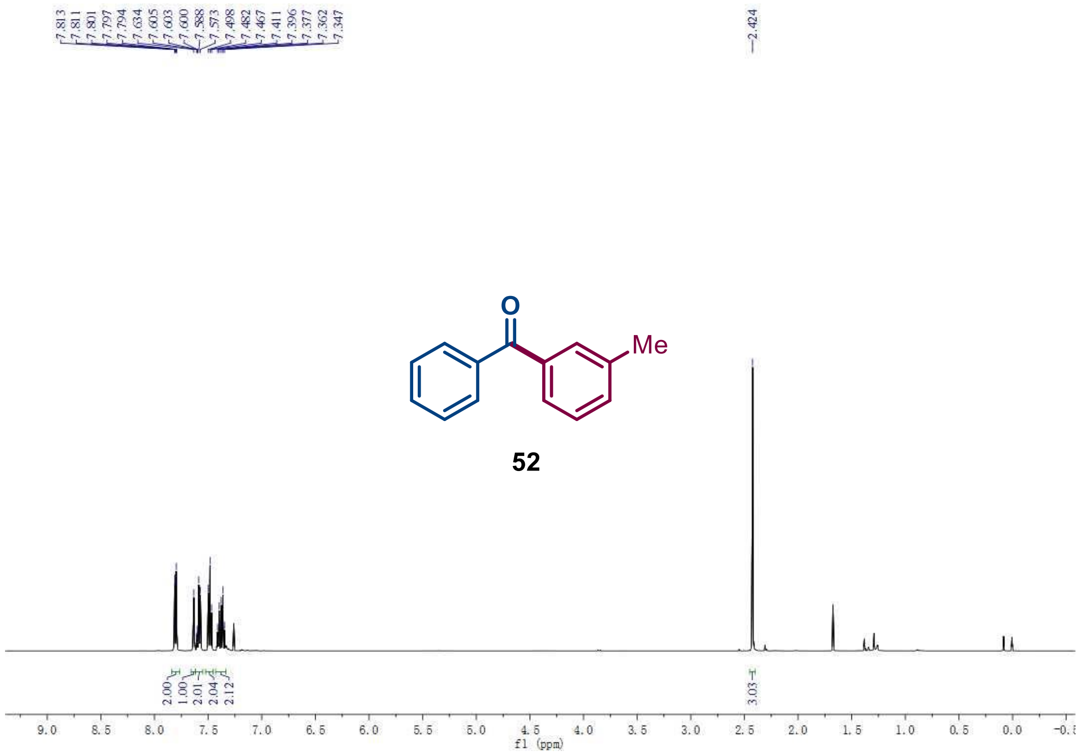

Supplementary Figure 146. ^{1}H NMR Spectrum of Compound **52**

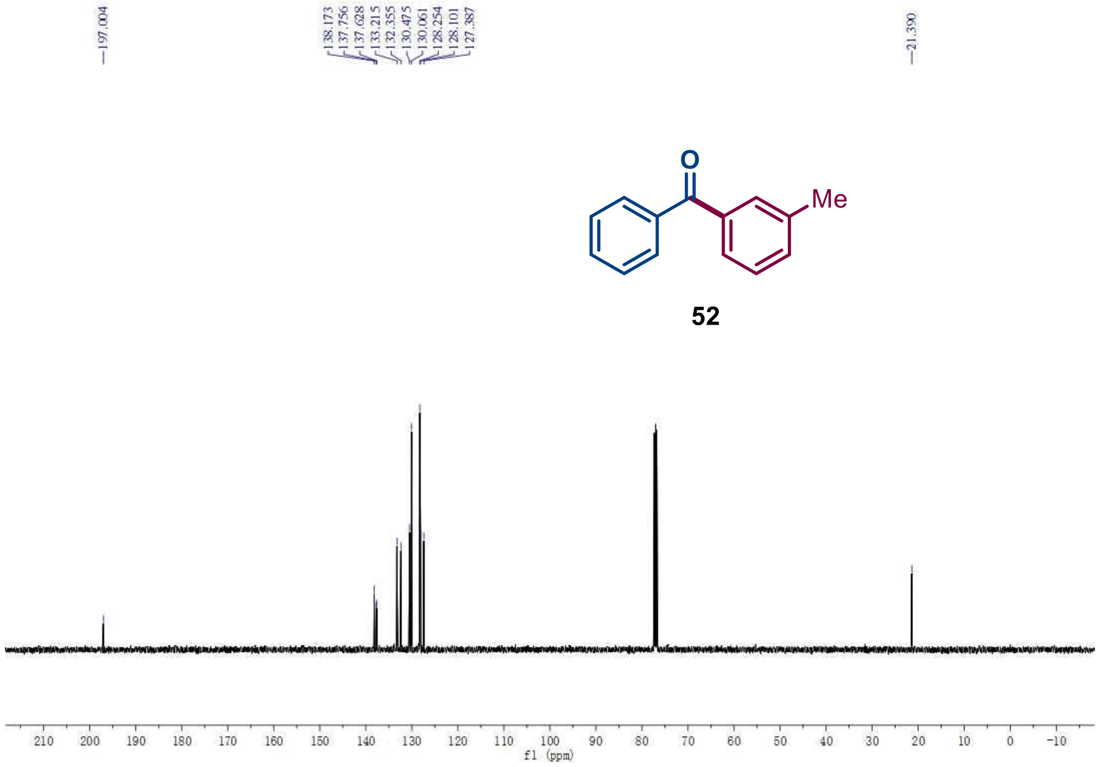

Supplementary Figure 147. ^{13}C NMR Spectrum of Compound**52**

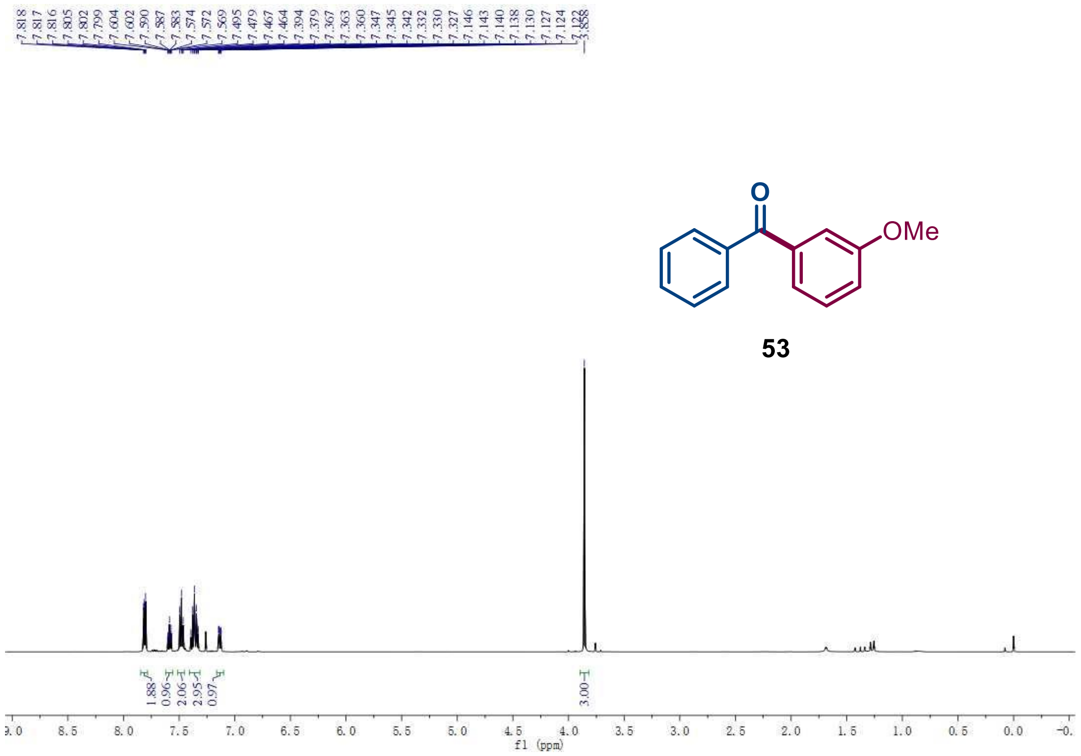

Supplementary Figure 148. ^{1}H NMR Spectrum of Compound **53**

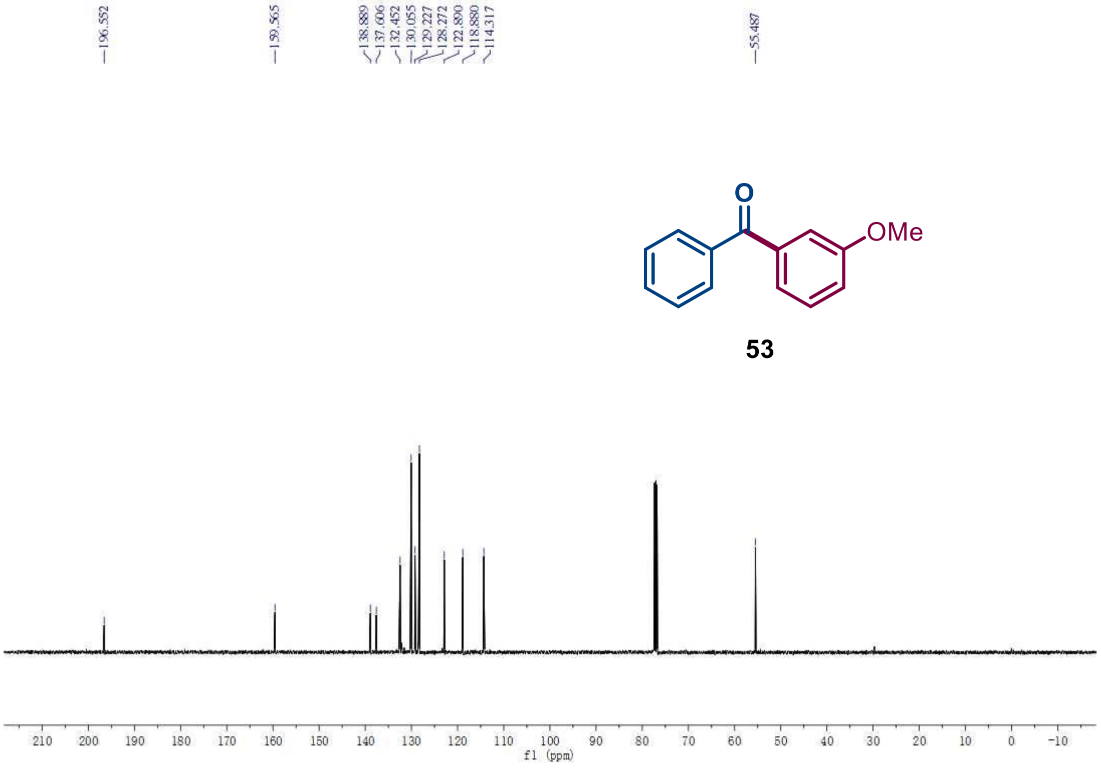

Supplementary Figure 149. ^{13}C NMR Spectrum of Compound **53**

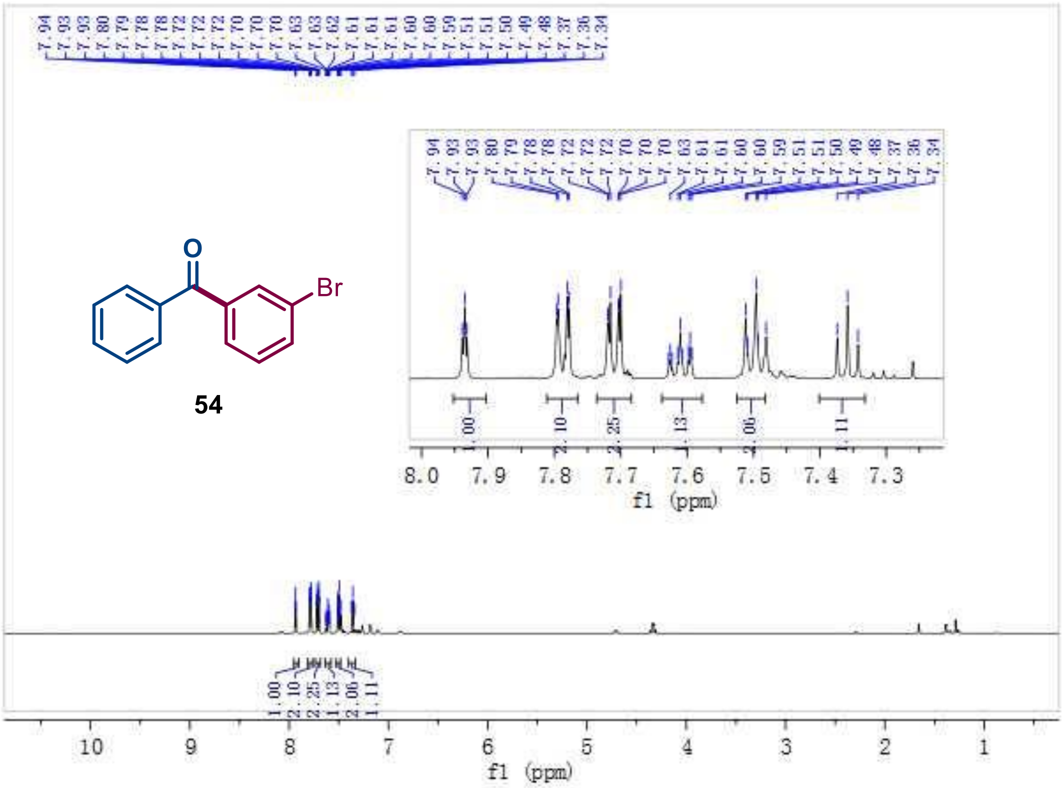

Supplementary Figure 150. ^{1}H NMR Spectrum of Compound **54**

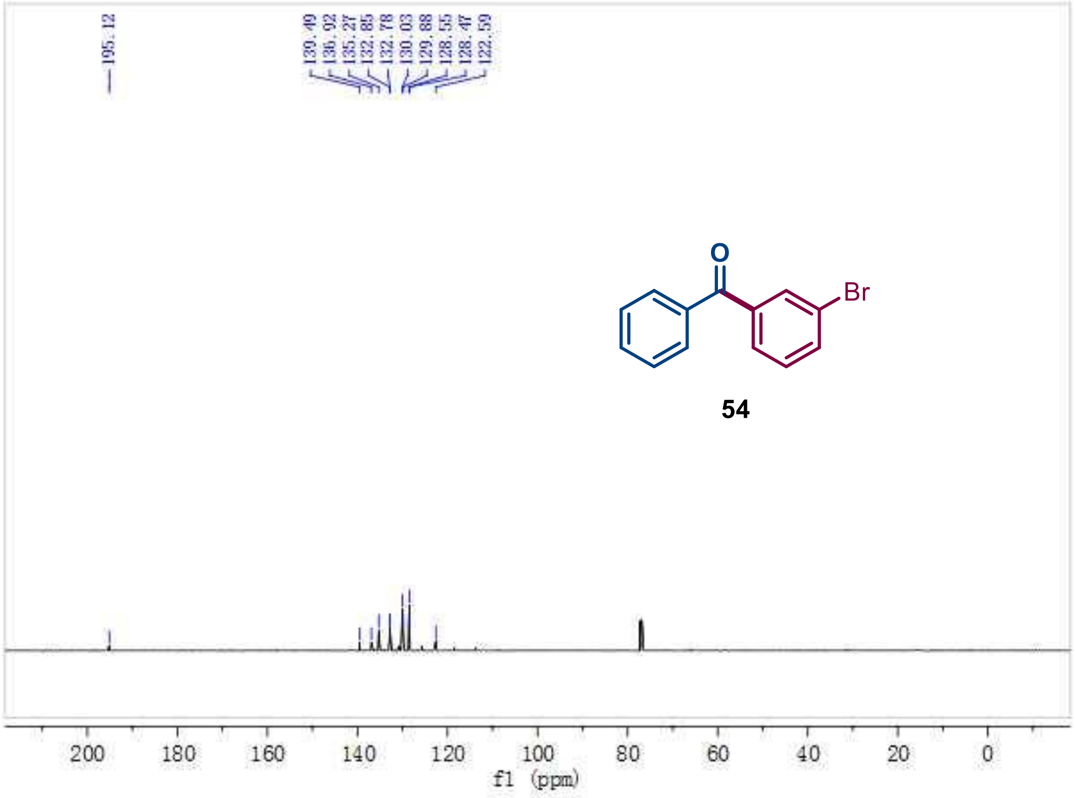

Supplementary Figure 151. ^{13}C NMR Spectrum of Compound **54**

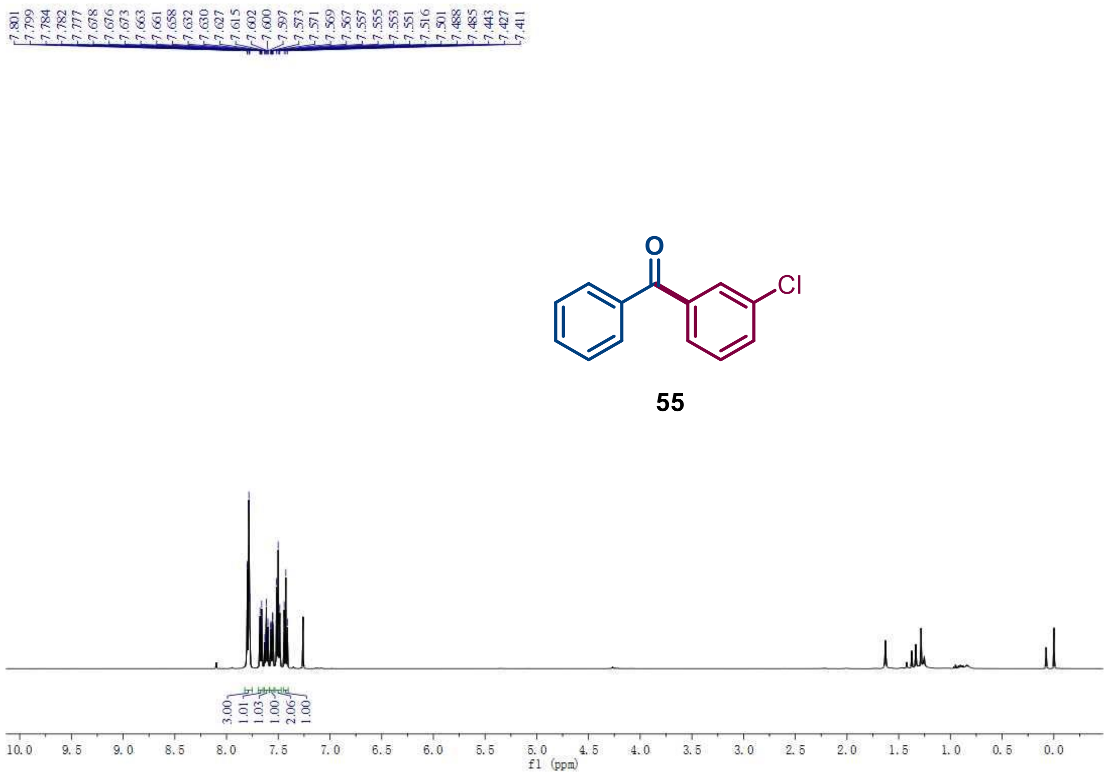

Supplementary Figure 152. ^{1}H NMR Spectrum of Compound **55**

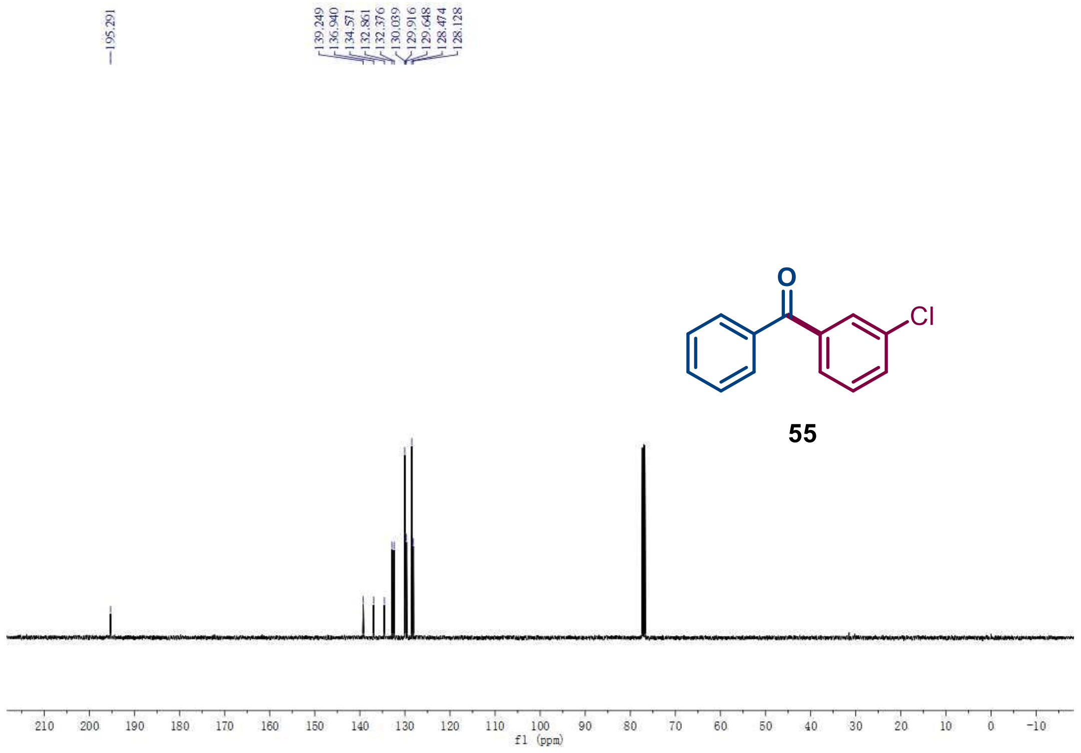

Supplementary Figure 153. ^{13}C NMR Spectrum of Compound **55**

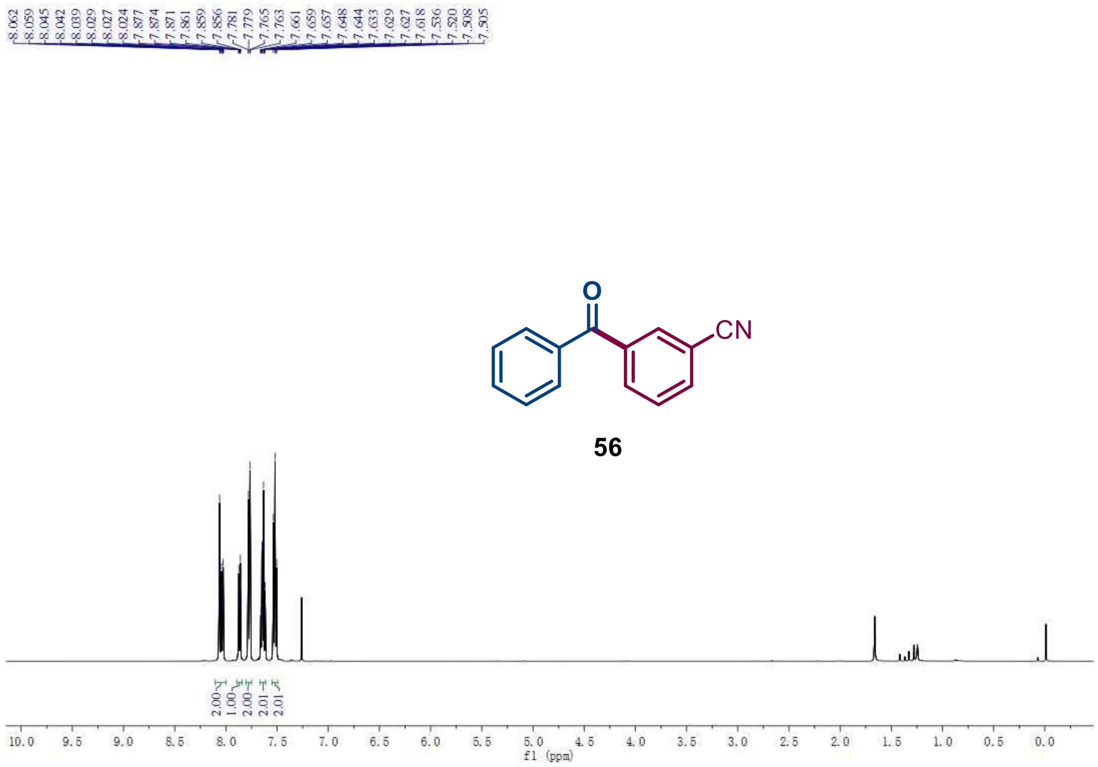

Supplementary Figure 154. ^{1}H NMR Spectrum of Compound **56**

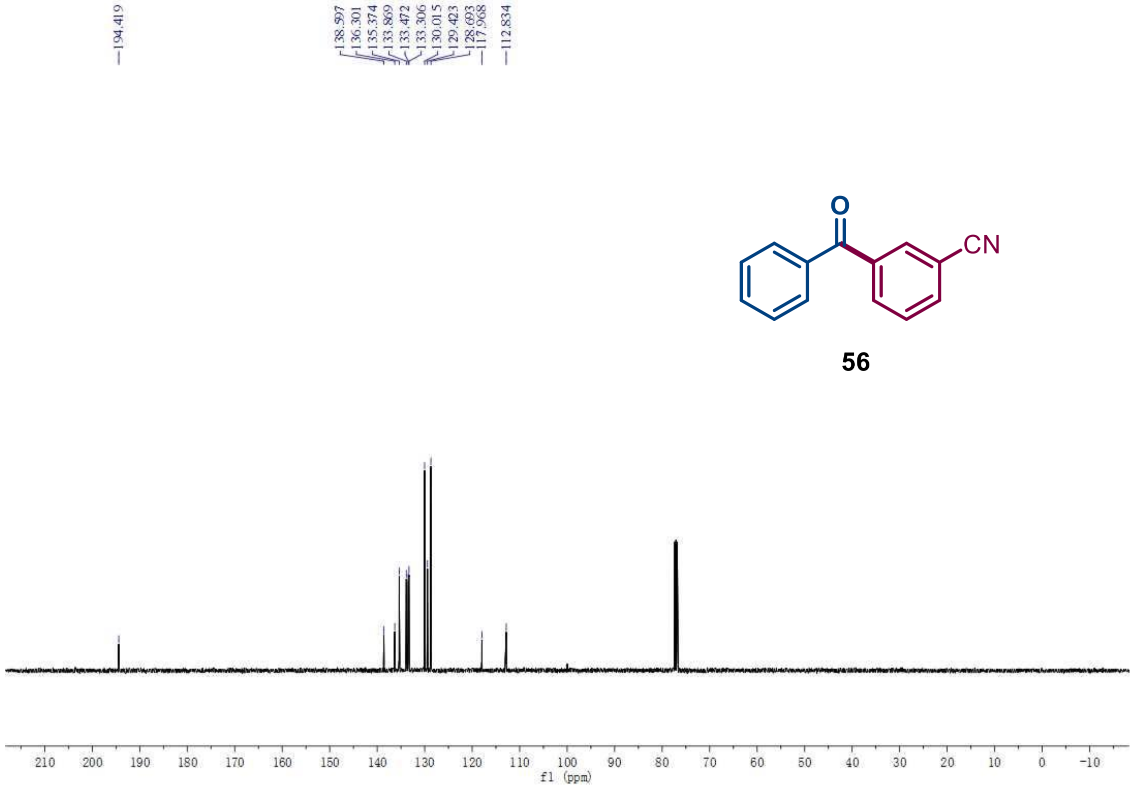

Supplementary Figure 155. ^{13}C NMR Spectrum of Compound **56**

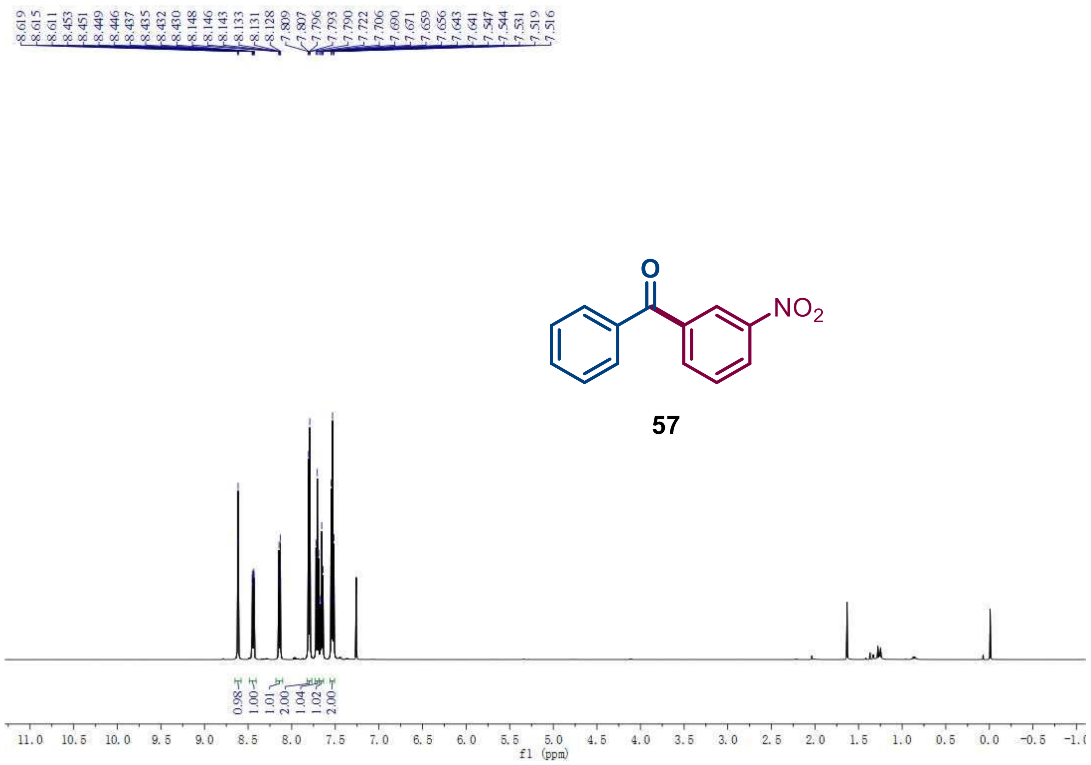

Supplementary Figure 156. ^{1}H NMR Spectrum of Compound **57**

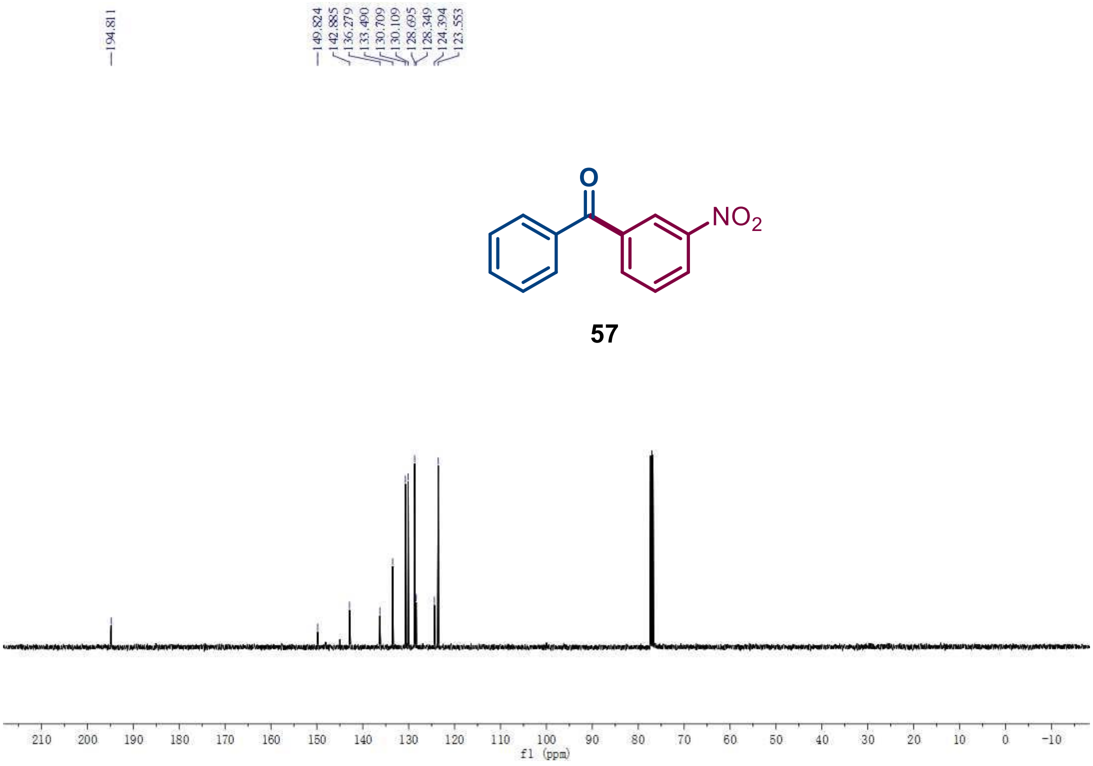

Supplementary Figure 157. ^{13}C NMR Spectrum of Compound **57**

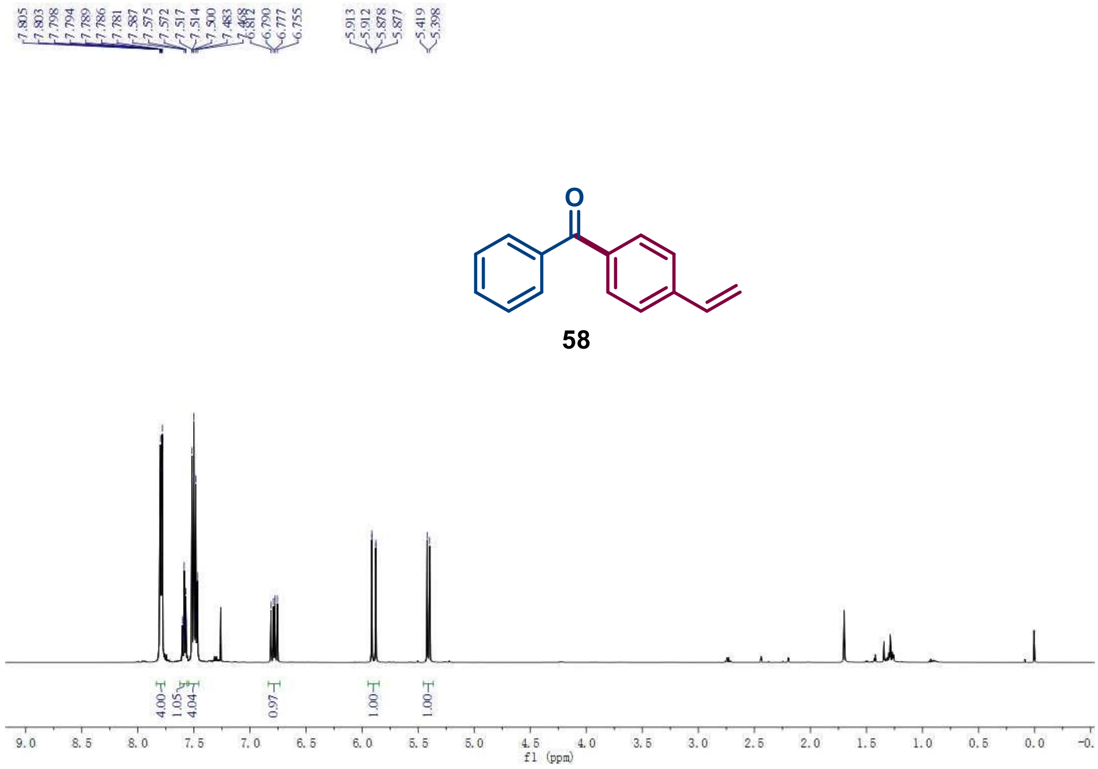

Supplementary Figure 158. ^{1}H NMR Spectrum of Compound **58**

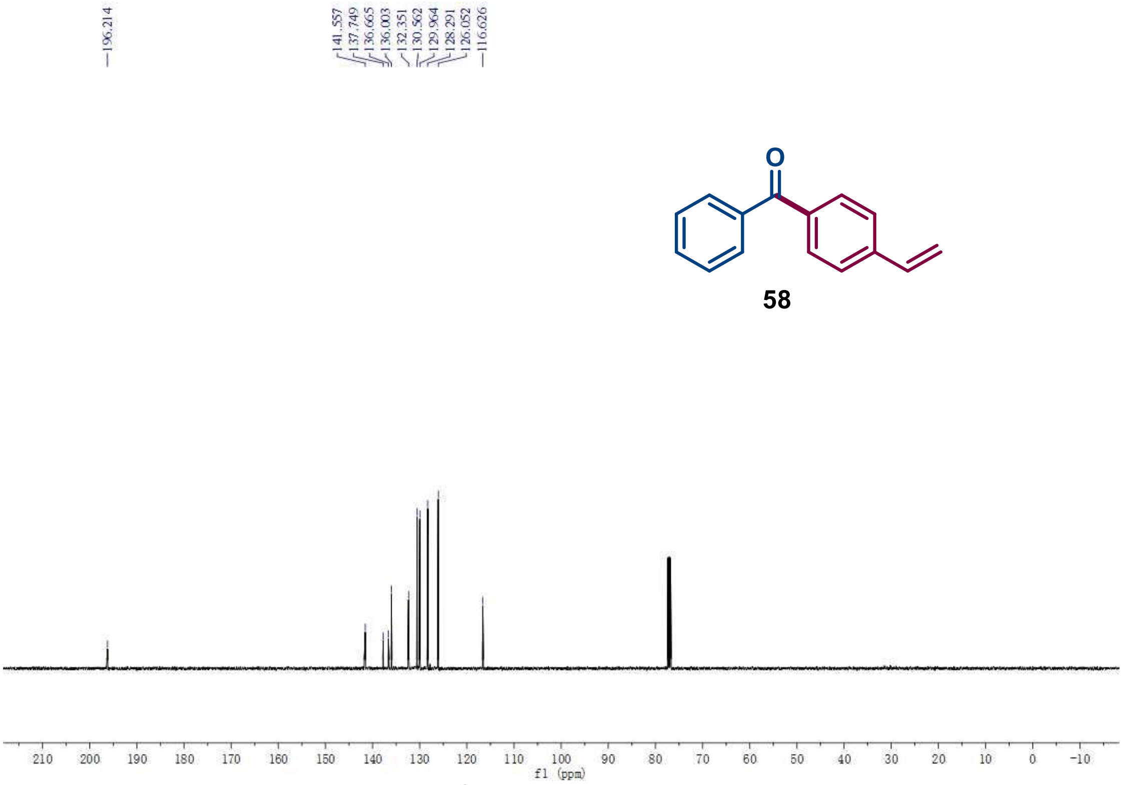

Supplementary Figure 159. ^{13}C NMR Spectrum of Compound **58**

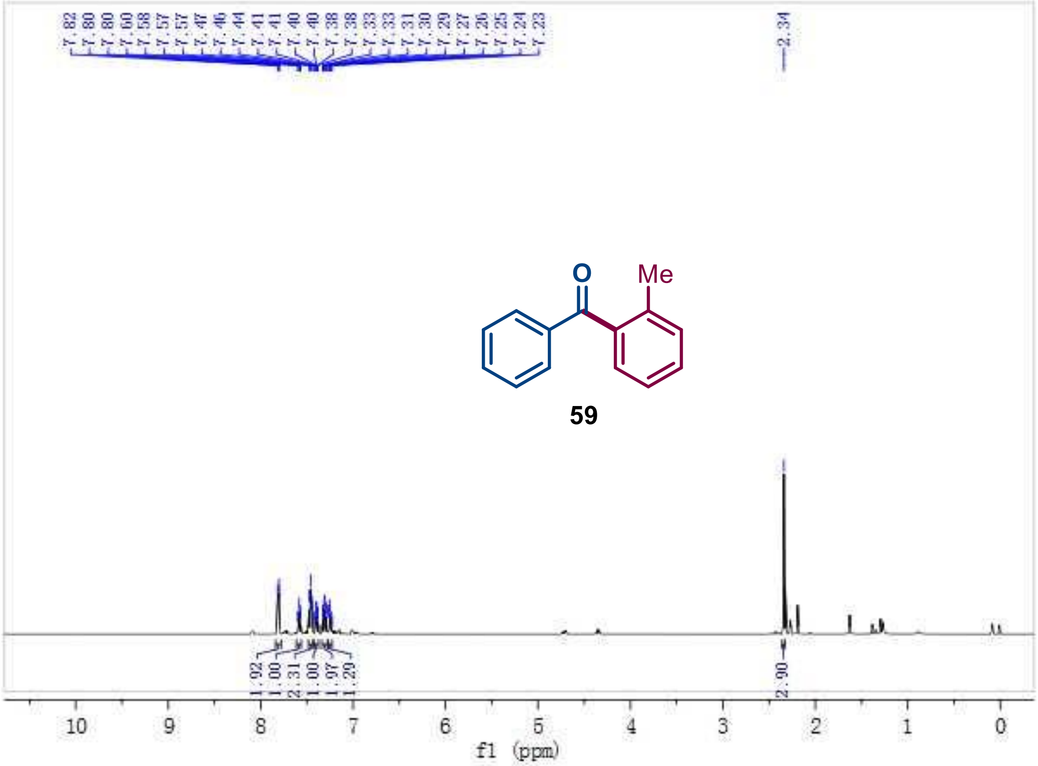

Supplementary Figure 160. ^{1}H NMR Spectrum of Compound **59**

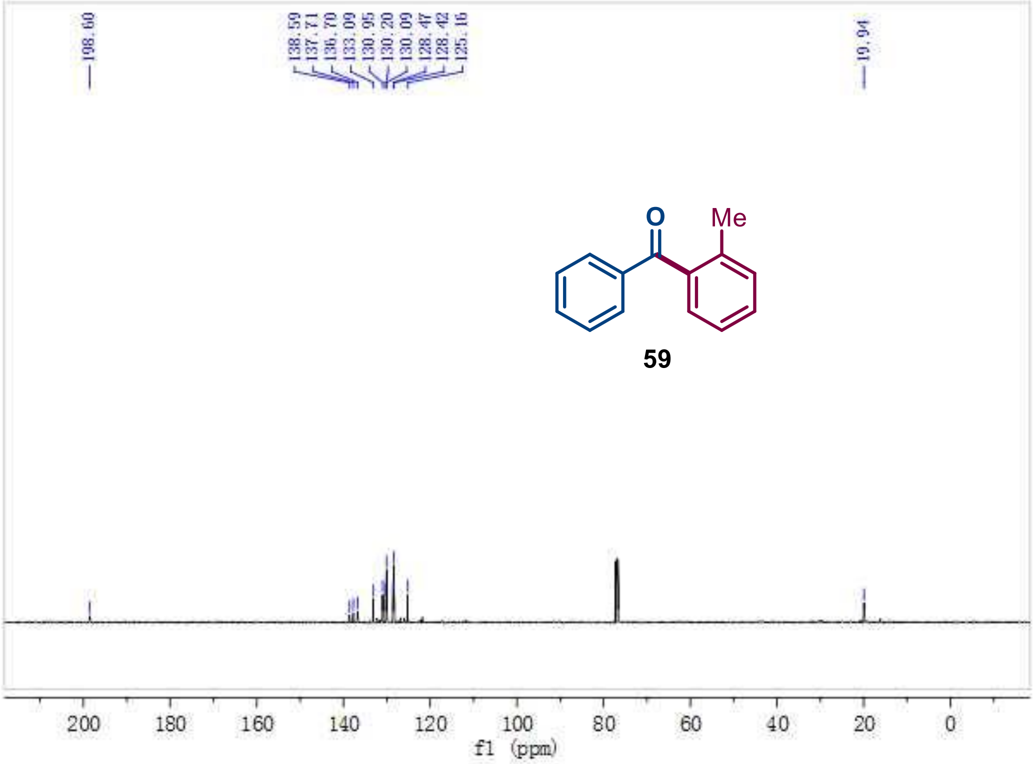

Supplementary Figure 161. ^{13}C NMR Spectrum of Compound **59**

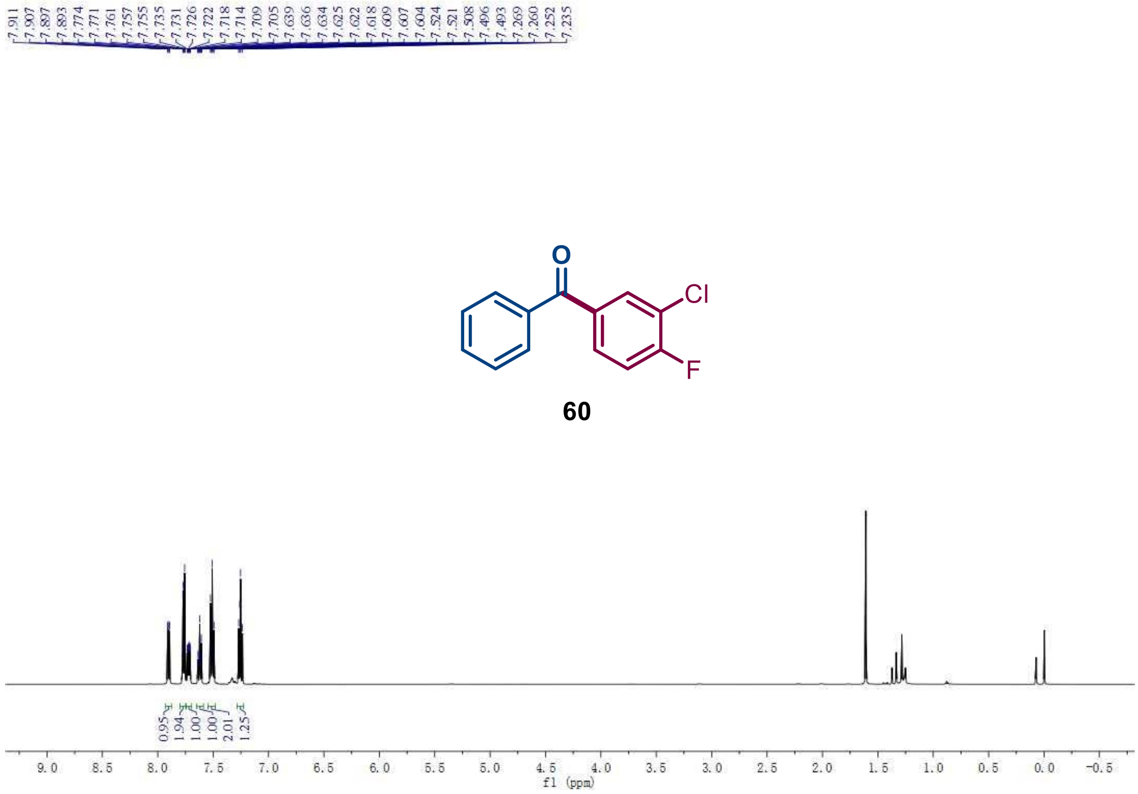

Supplementary Figure 162. ^{1}H NMR Spectrum of Compound **60**

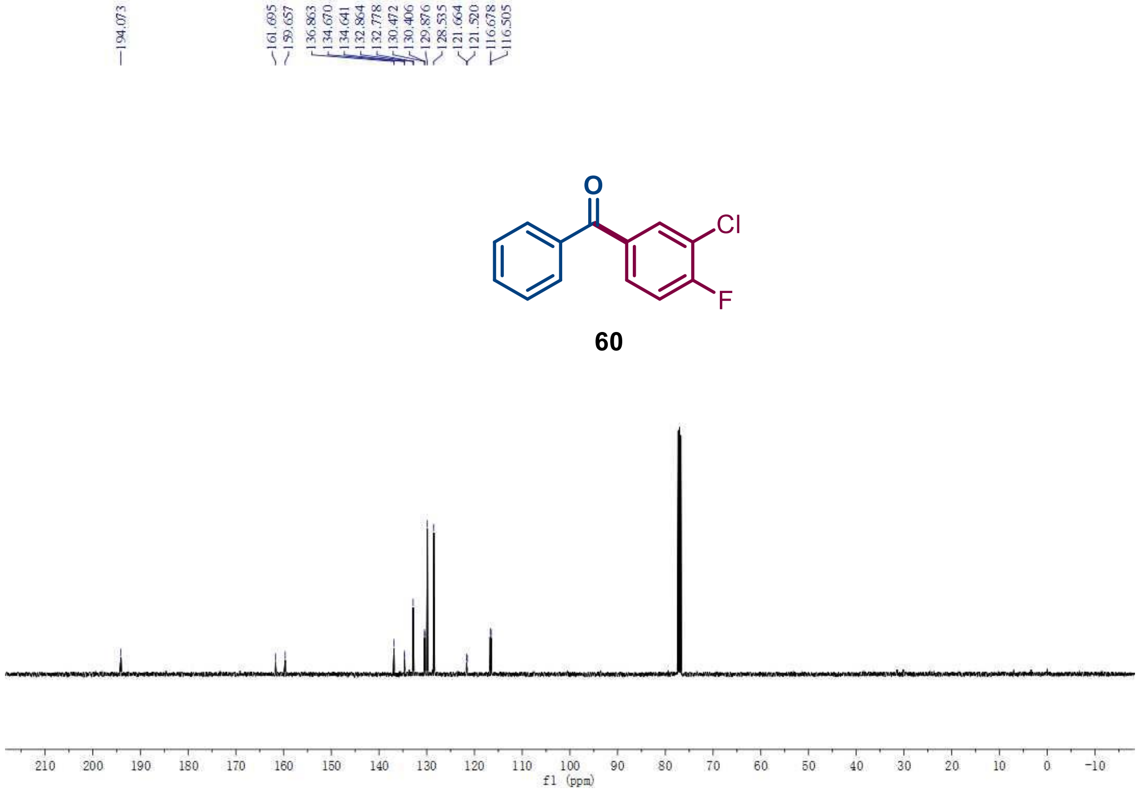

Supplementary Figure 163. ^{13}C NMR Spectrum of Compound **60**

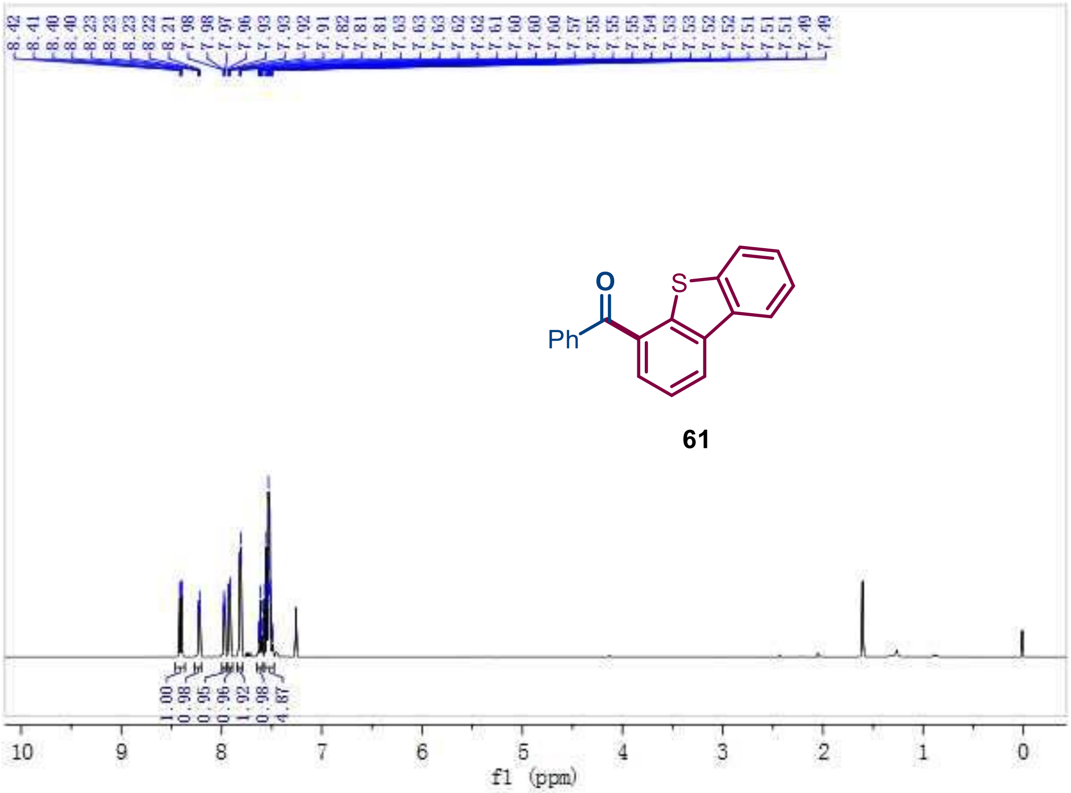

Supplementary Figure 164. ^{1}H NMR Spectrum of Compound **61**

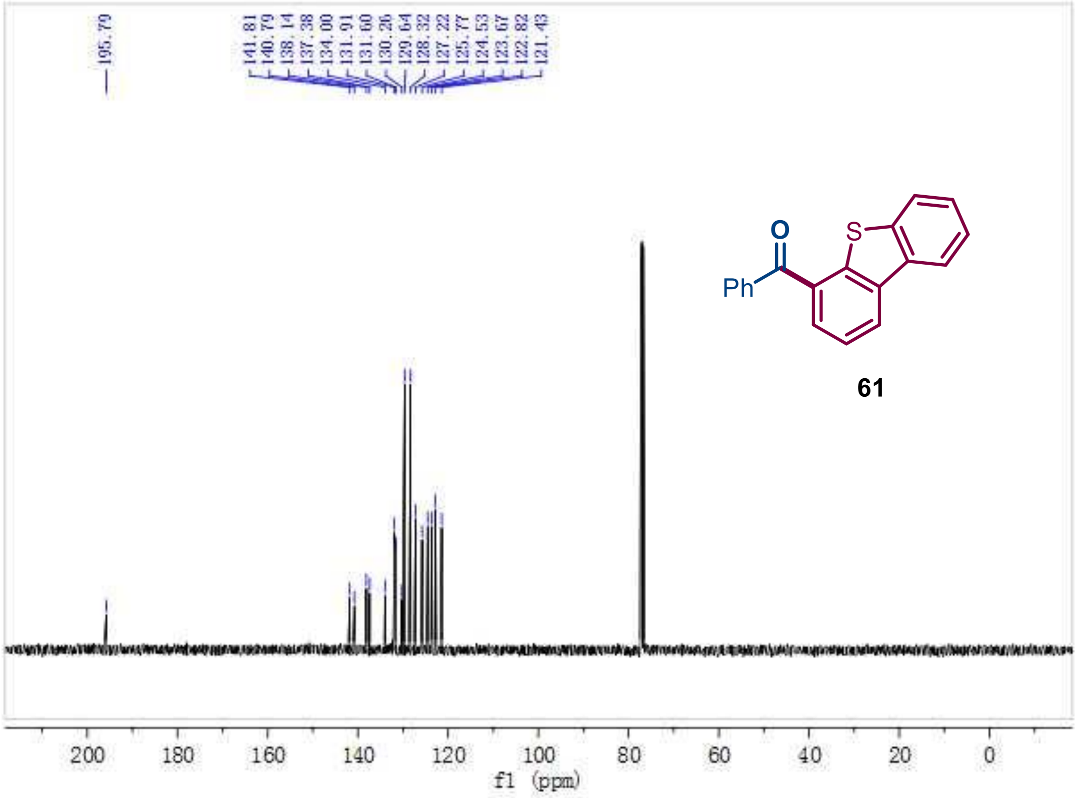

Supplementary Figure 165. ^{13}C NMR Spectrum of Compound **61**

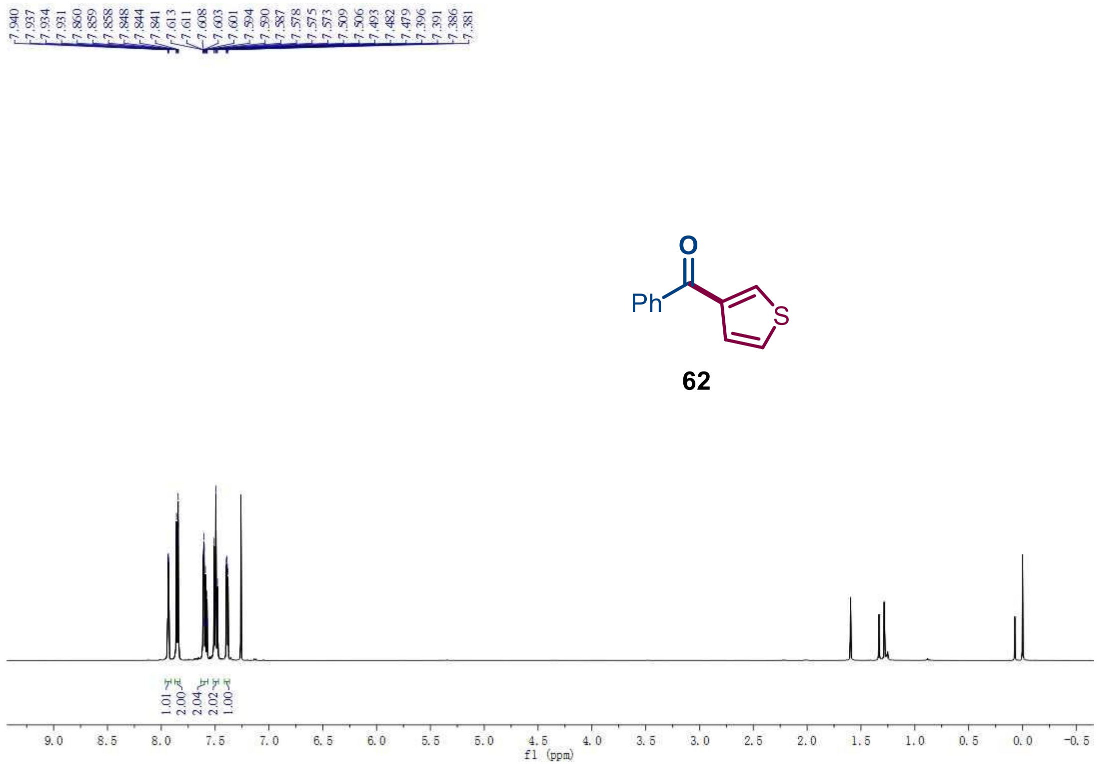

Supplementary Figure 166. ^{1}H NMR Spectrum of Compound **62**

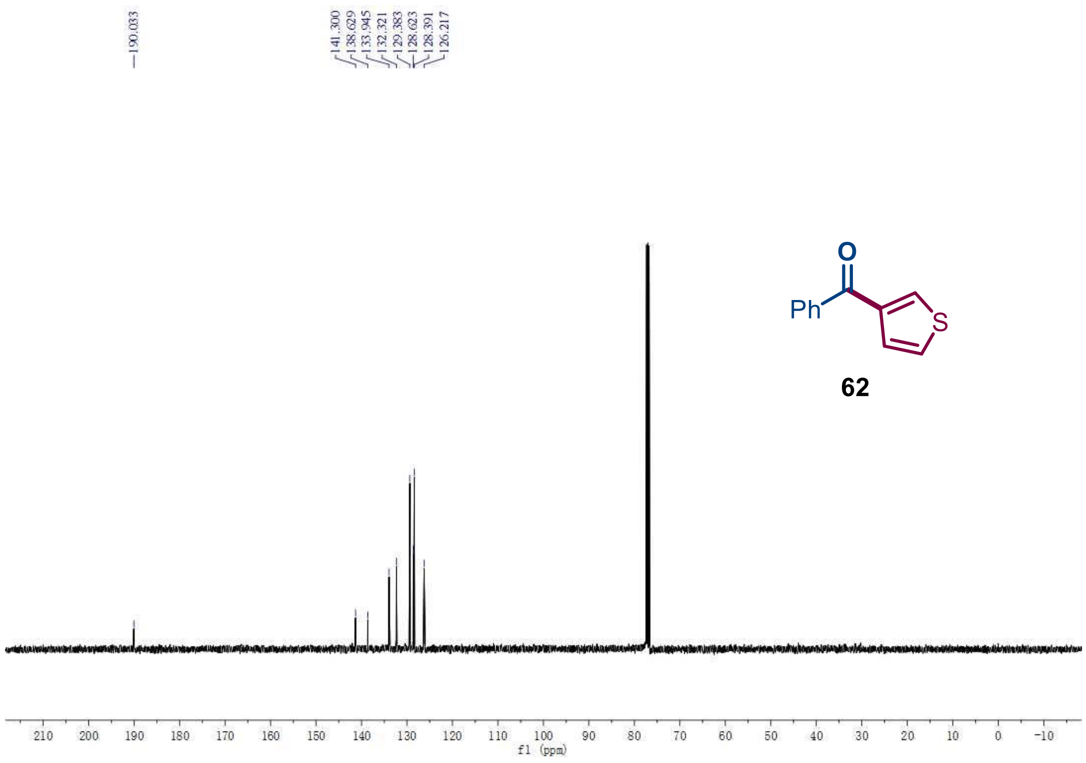

Supplementary Figure 167. ^{13}C NMR Spectrum of Compound **62**

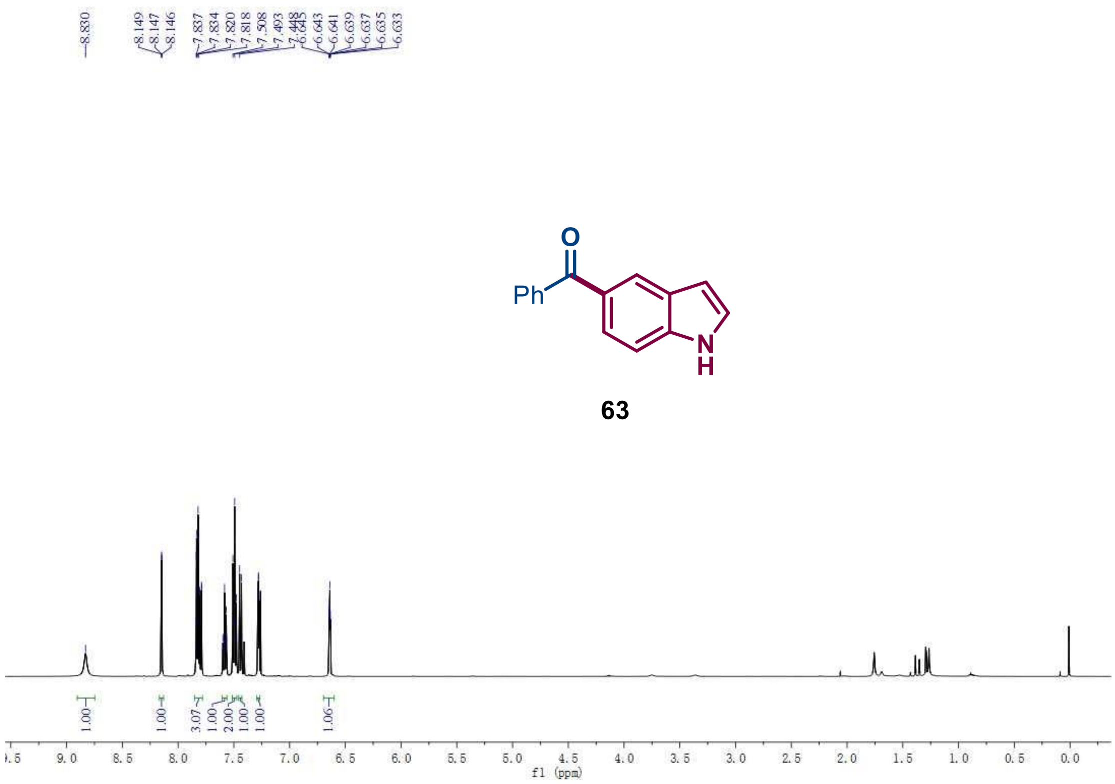

Supplementary Figure 168. ^{1}H NMR Spectrum of Compound **63**

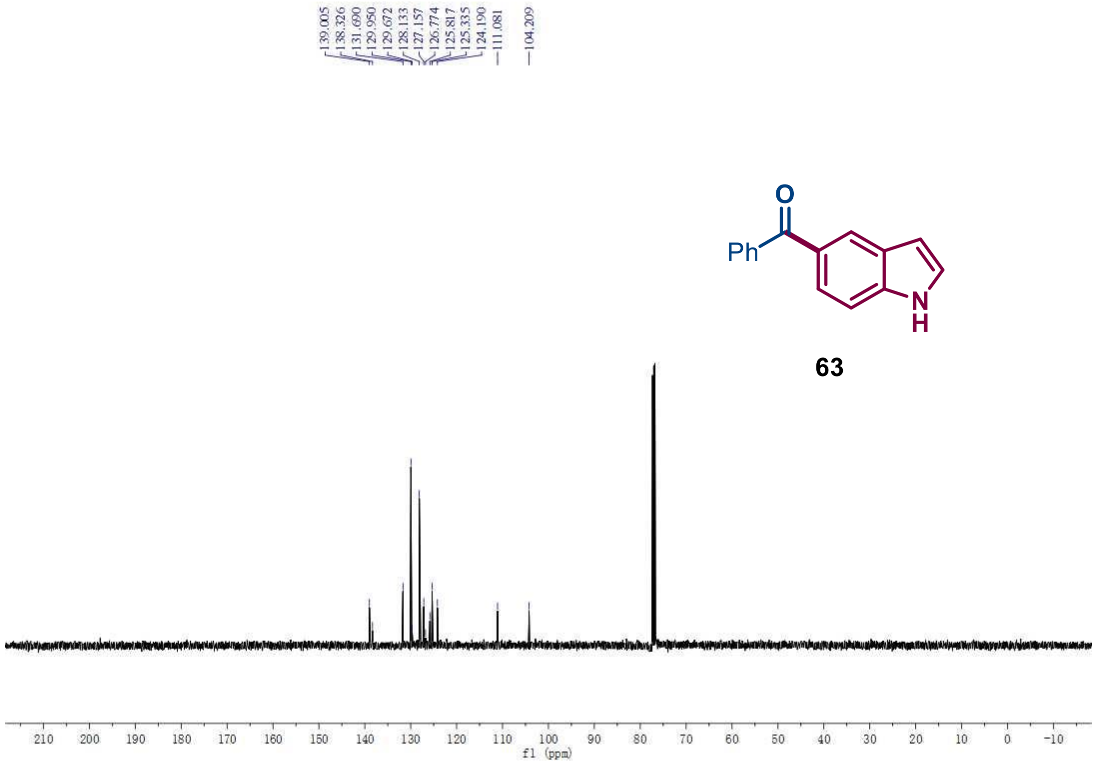

Supplementary Figure 169. ^{13}C NMR Spectrum of Compound **63**

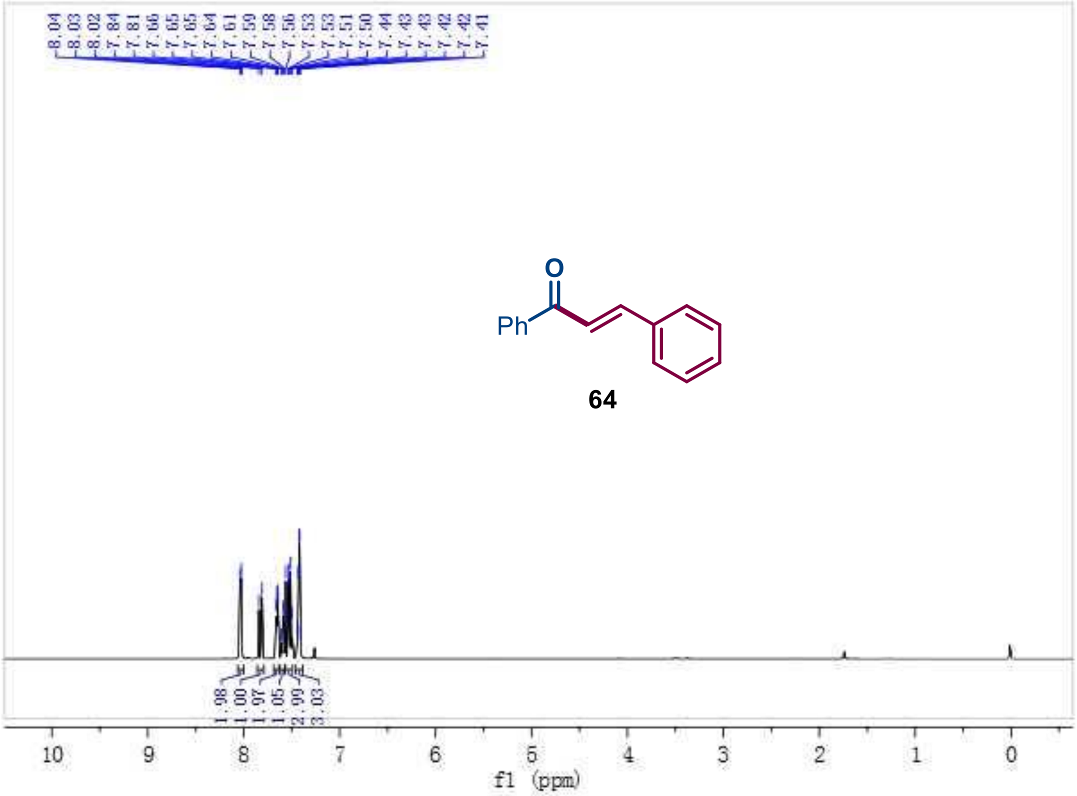

Supplementary Figure 170. ^{1}H NMR Spectrum of Compound **64**

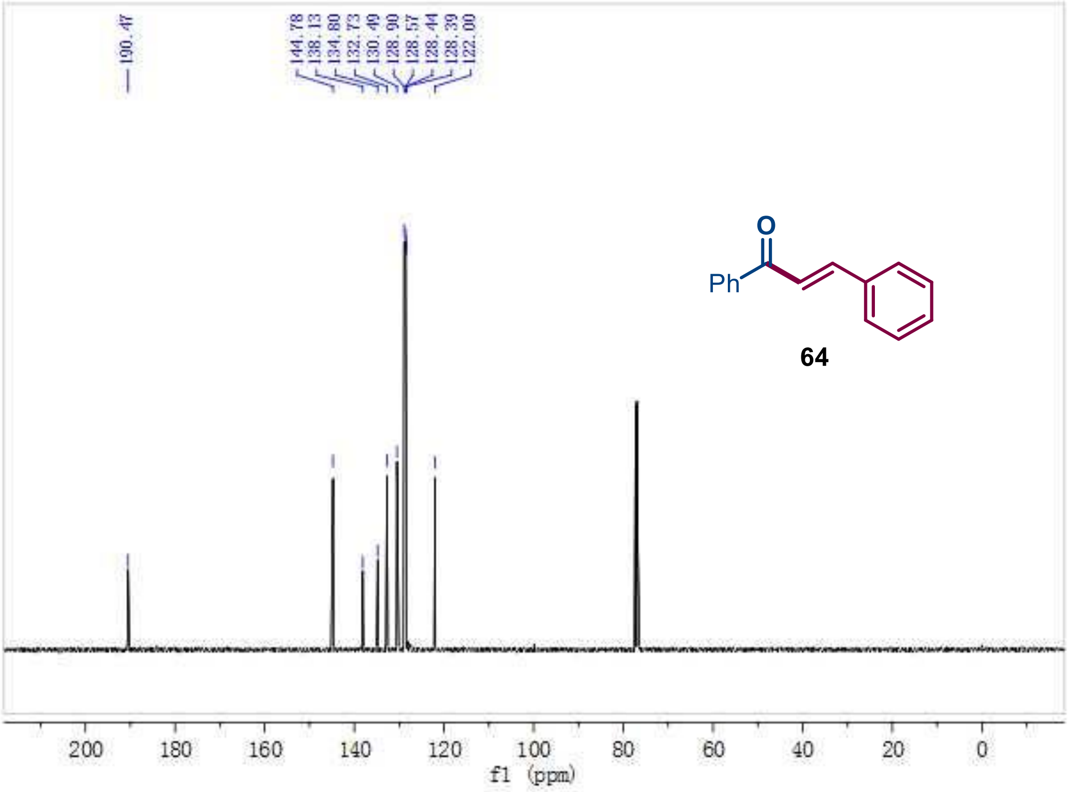

Supplementary Figure 171. ^{13}C NMR Spectrum of Compound **64**

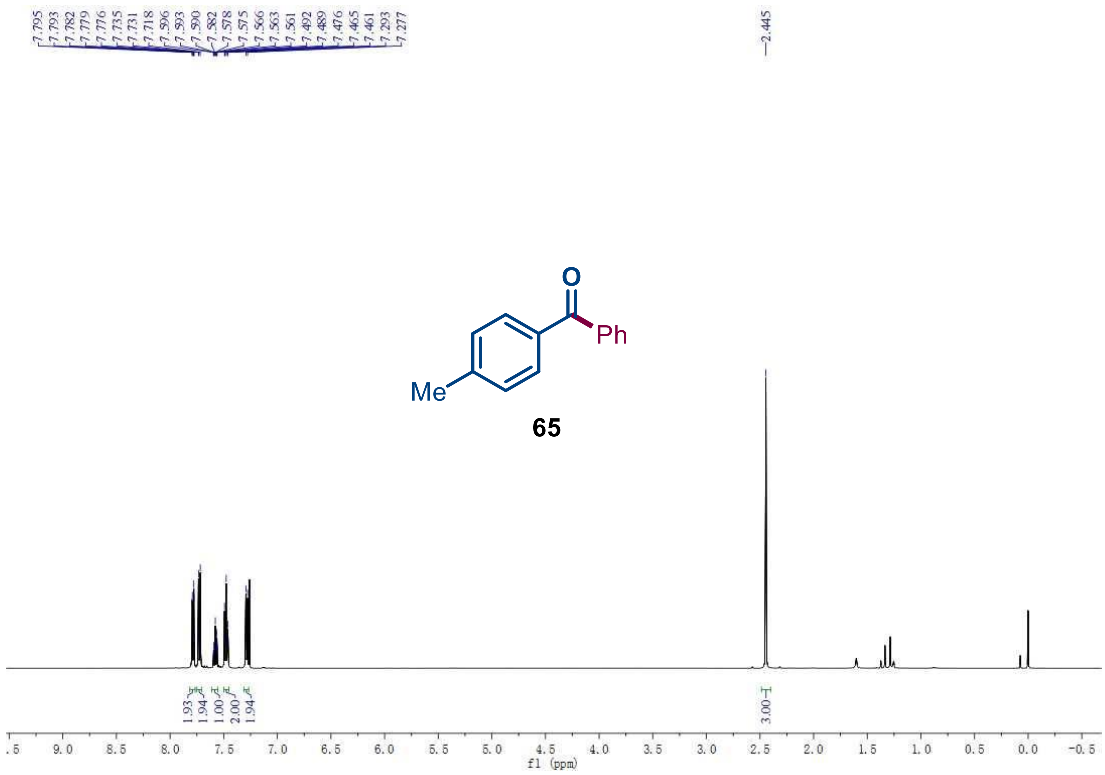

Supplementary Figure 172. ^{1}H NMR Spectrum of Compound **65**

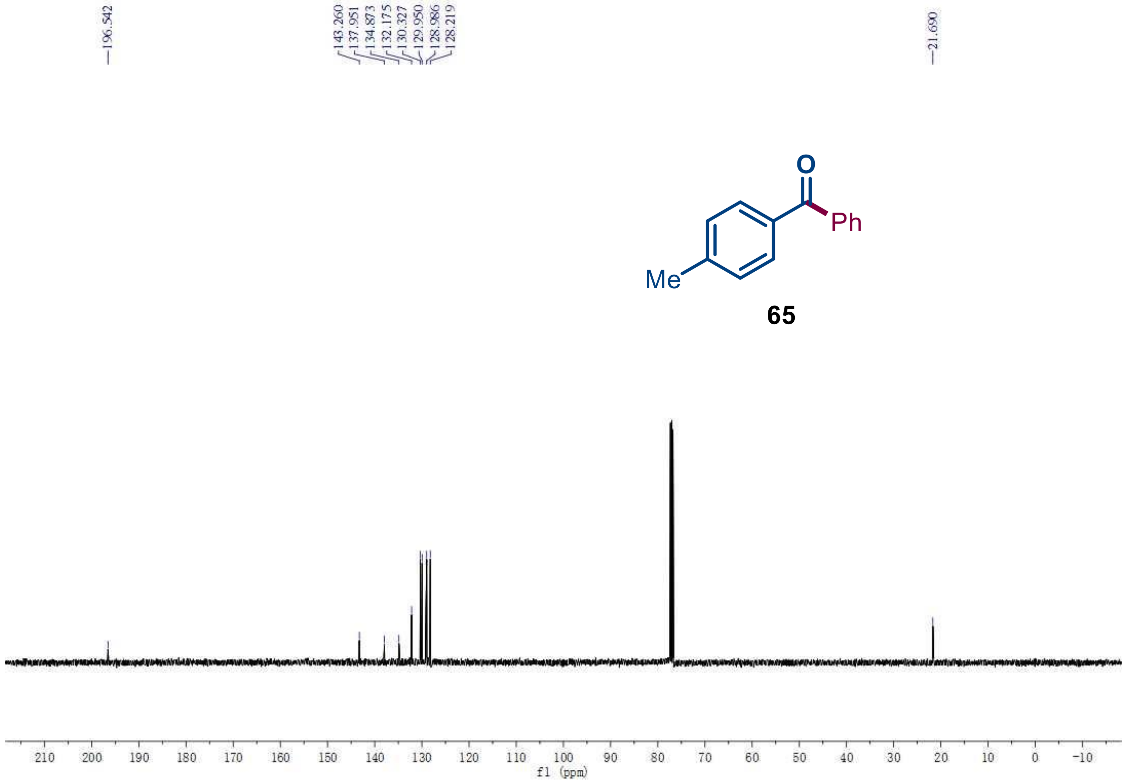

Supplementary Figure 173. ^{13}C NMR Spectrum of Compound **65**

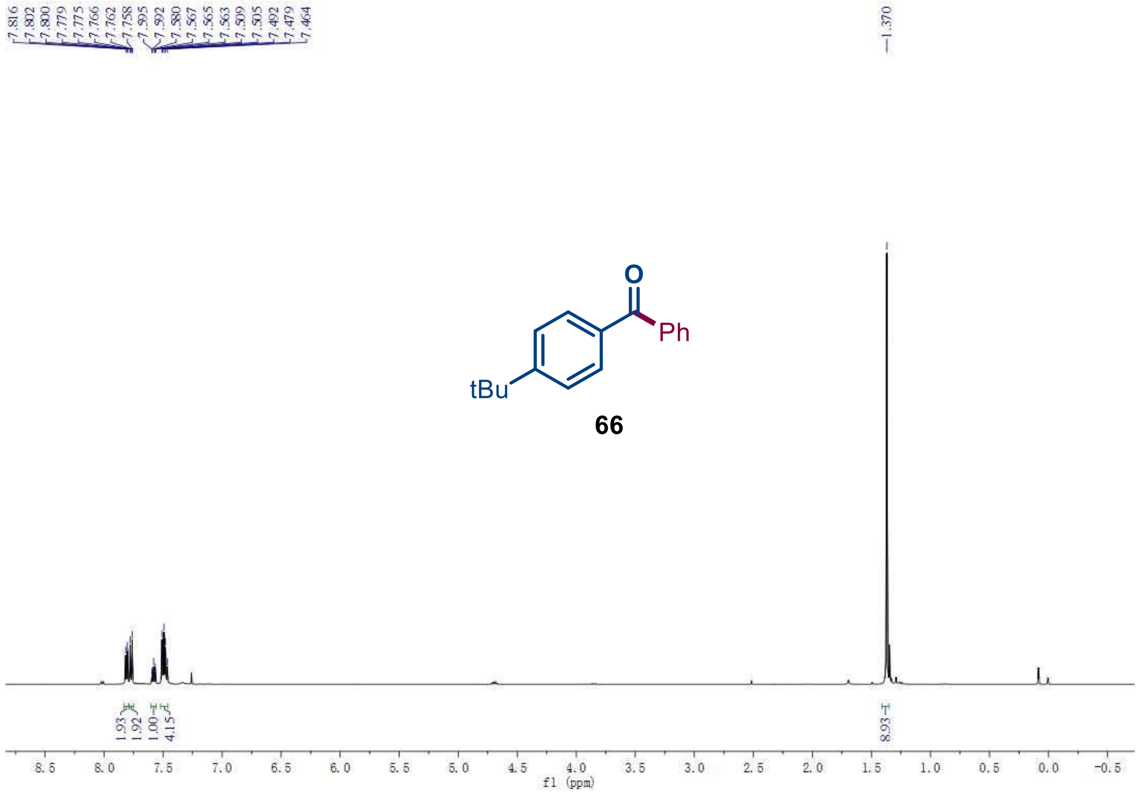

Supplementary Figure 174. ^{1}H NMR Spectrum of Compound **66**

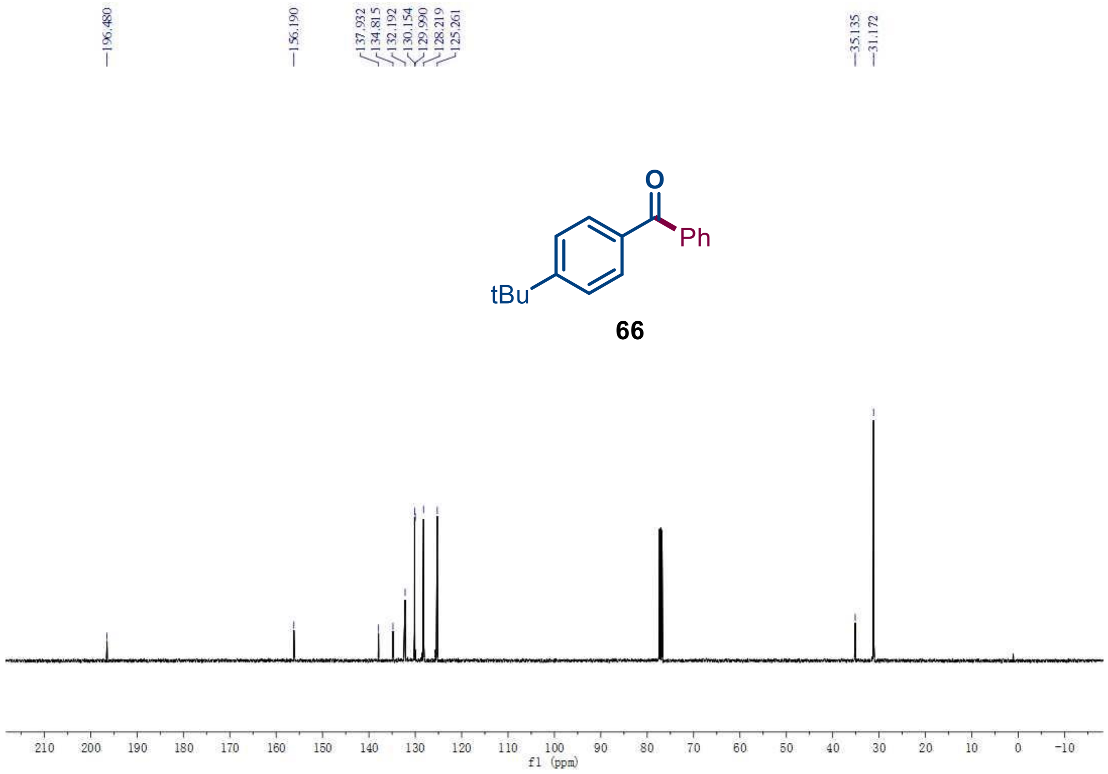

Supplementary Figure 175. ^{13}C NMR Spectrum of Compound **66**

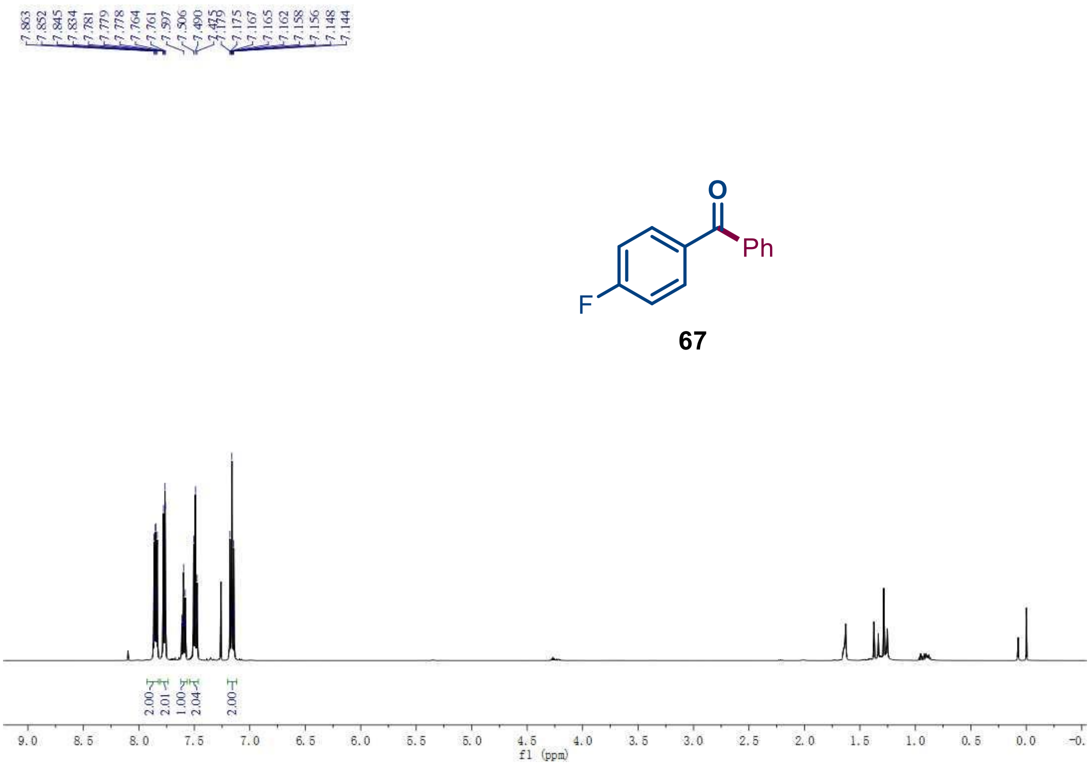

Supplementary Figure 176. ^{1}H NMR Spectrum of Compound **67**

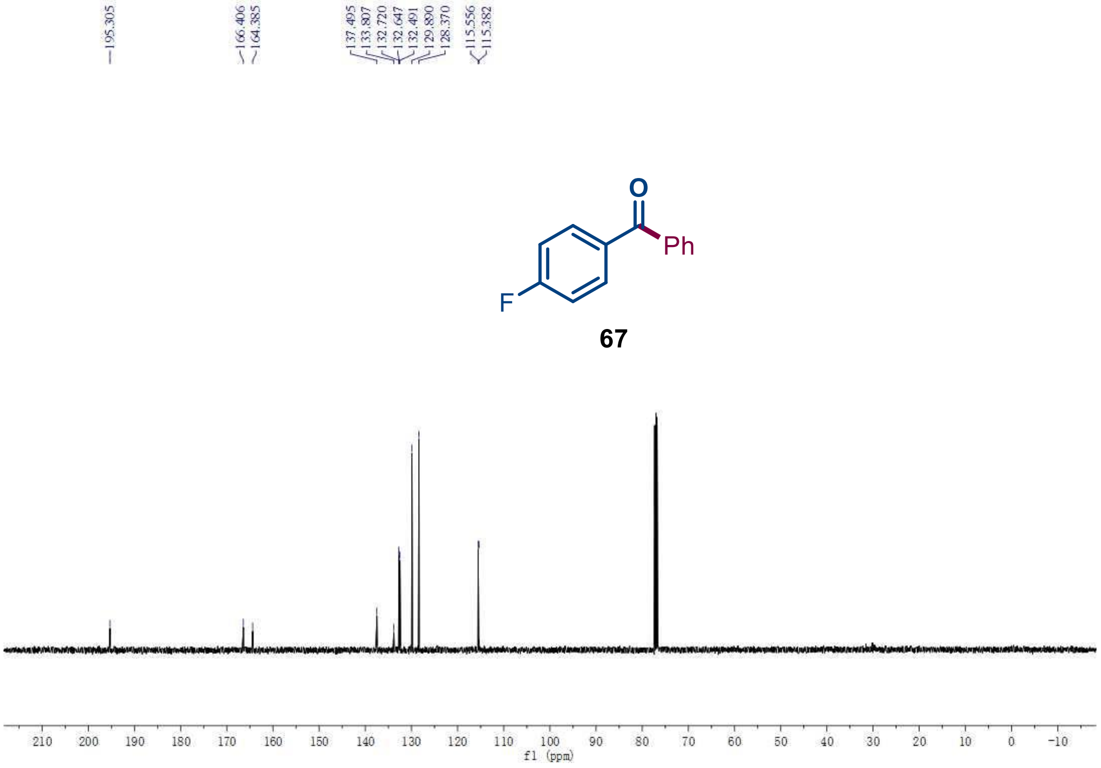

Supplementary Figure 177. ^{13}C NMR Spectrum of Compound **67**

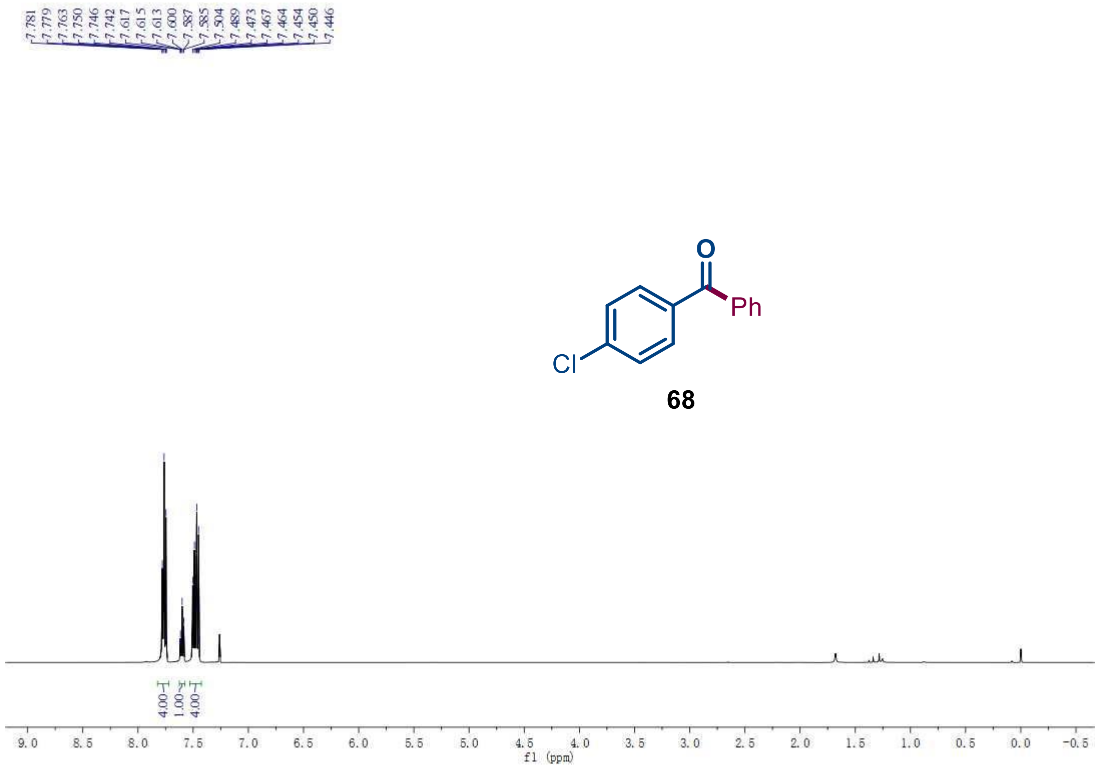

Supplementary Figure 178. ^{1}H NMR Spectrum of Compound **68**

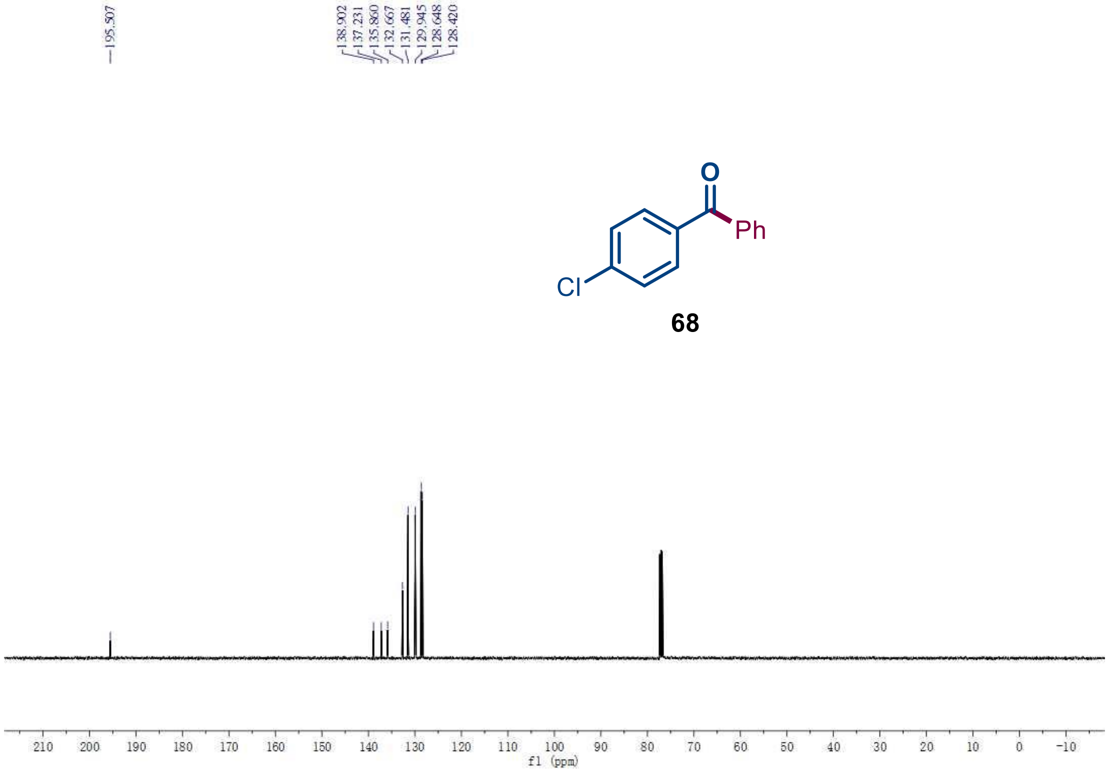

Supplementary Figure 179. ^{13}C NMR Spectrum of Compound **68**

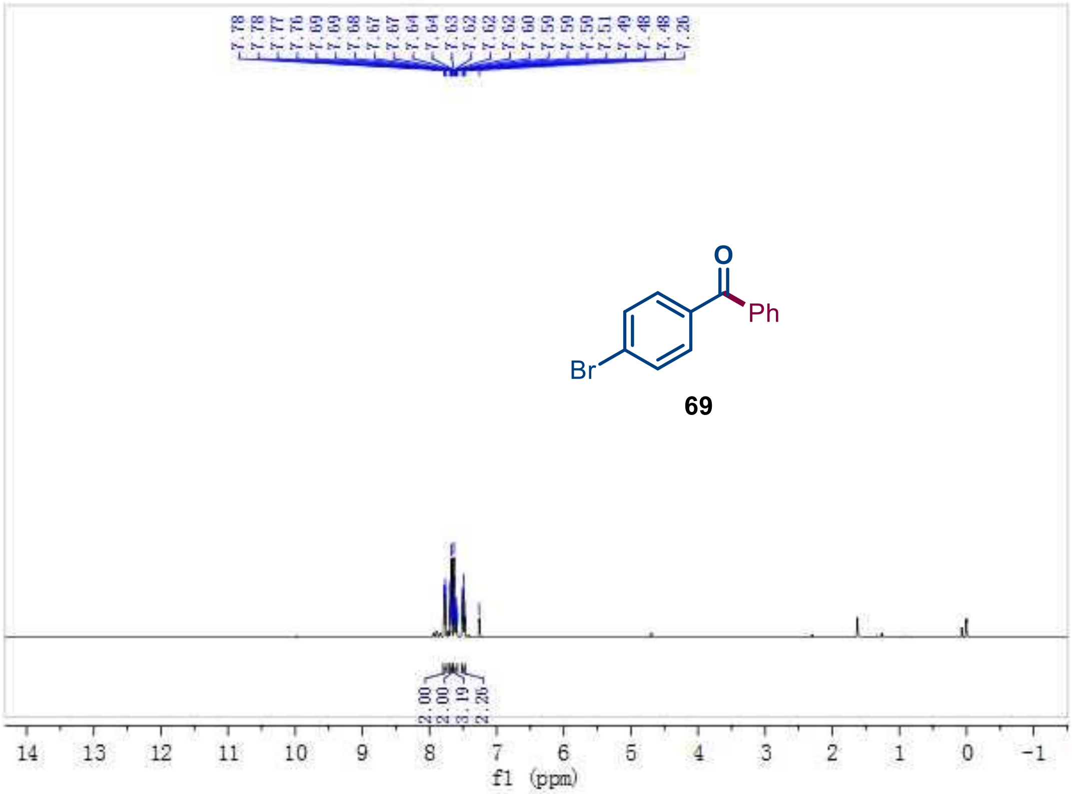

Supplementary Figure 180. ^{1}H NMR Spectrum of Compound **69**

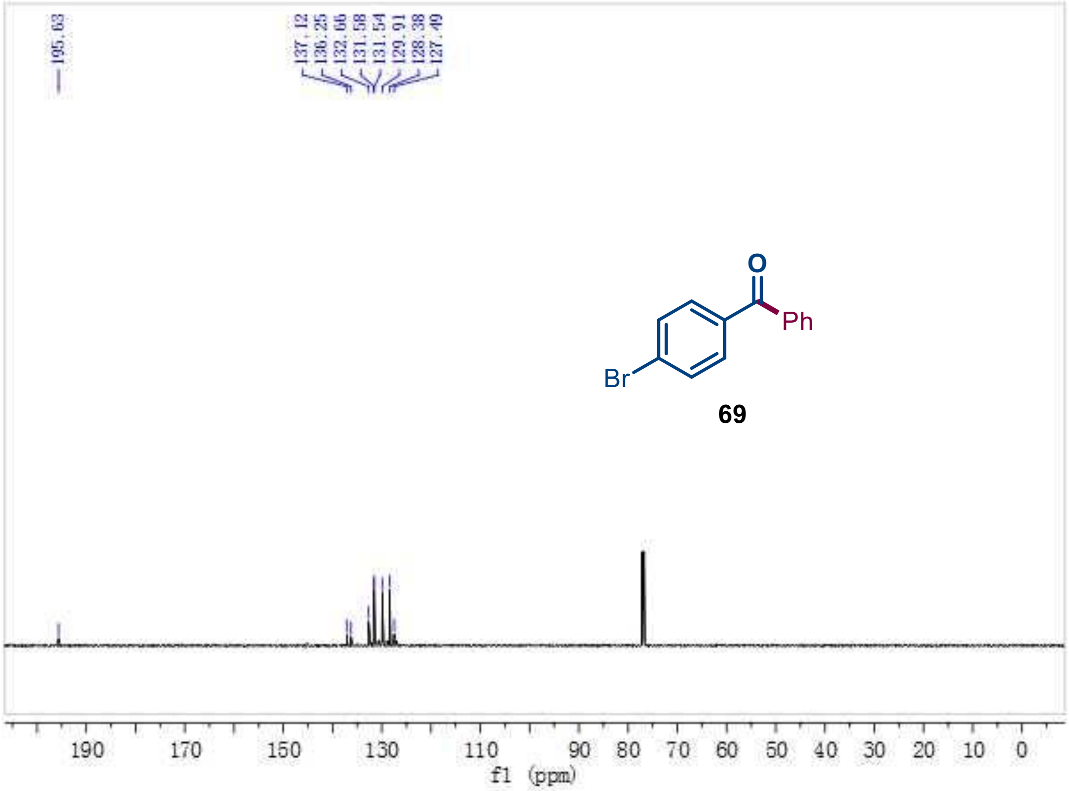

Supplementary Figure 181. ^{13}C NMR Spectrum of Compound **69**

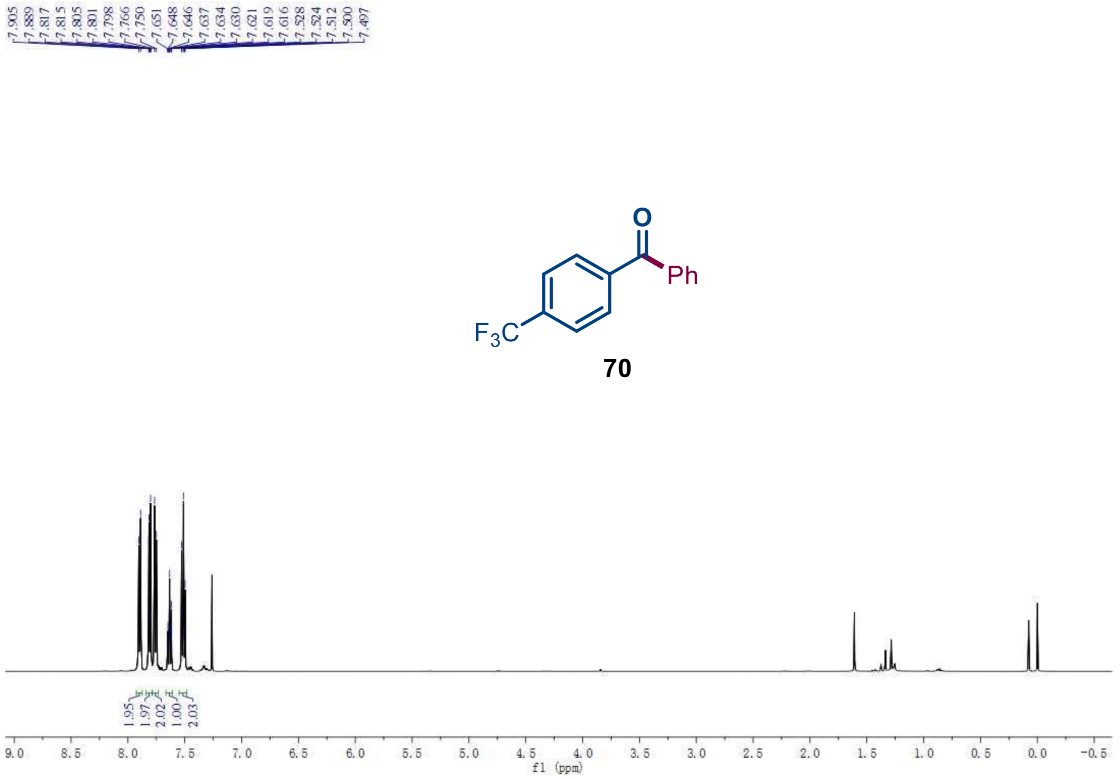

Supplementary Figure 182. ^{1}H NMR Spectrum of Compound **70**

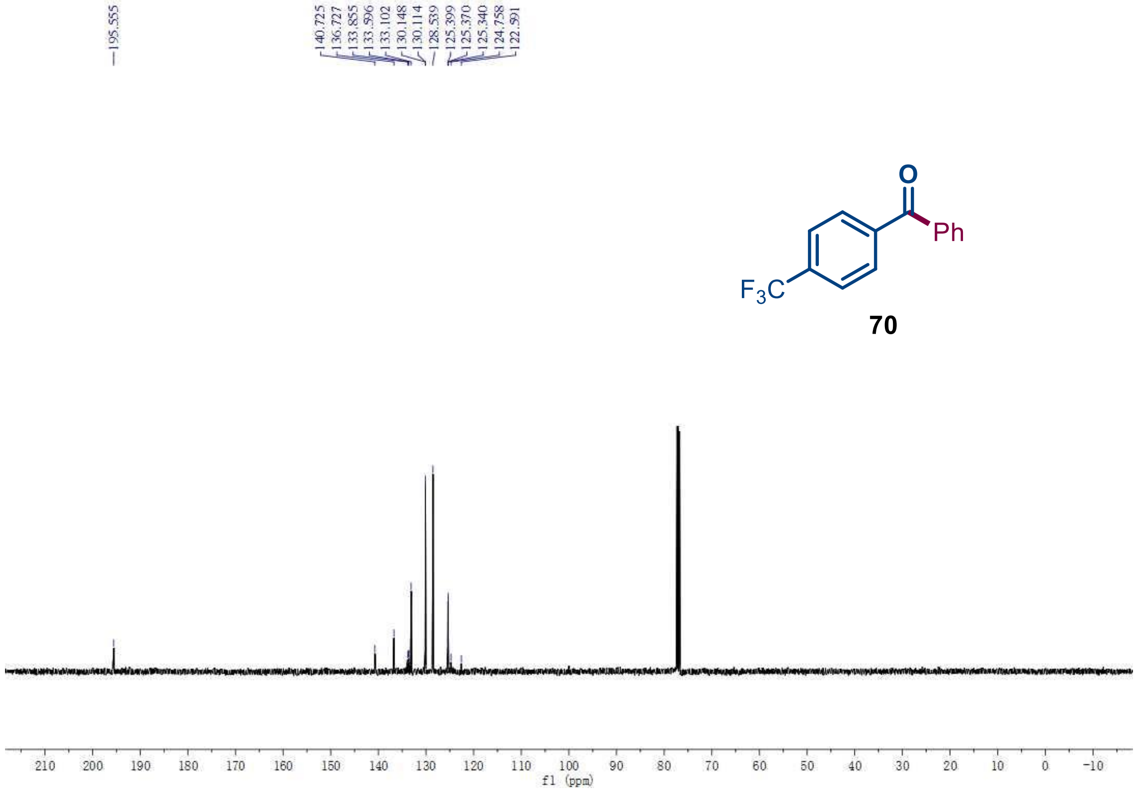

Supplementary Figure 183. ^{13}C NMR Spectrum of Compound **70**

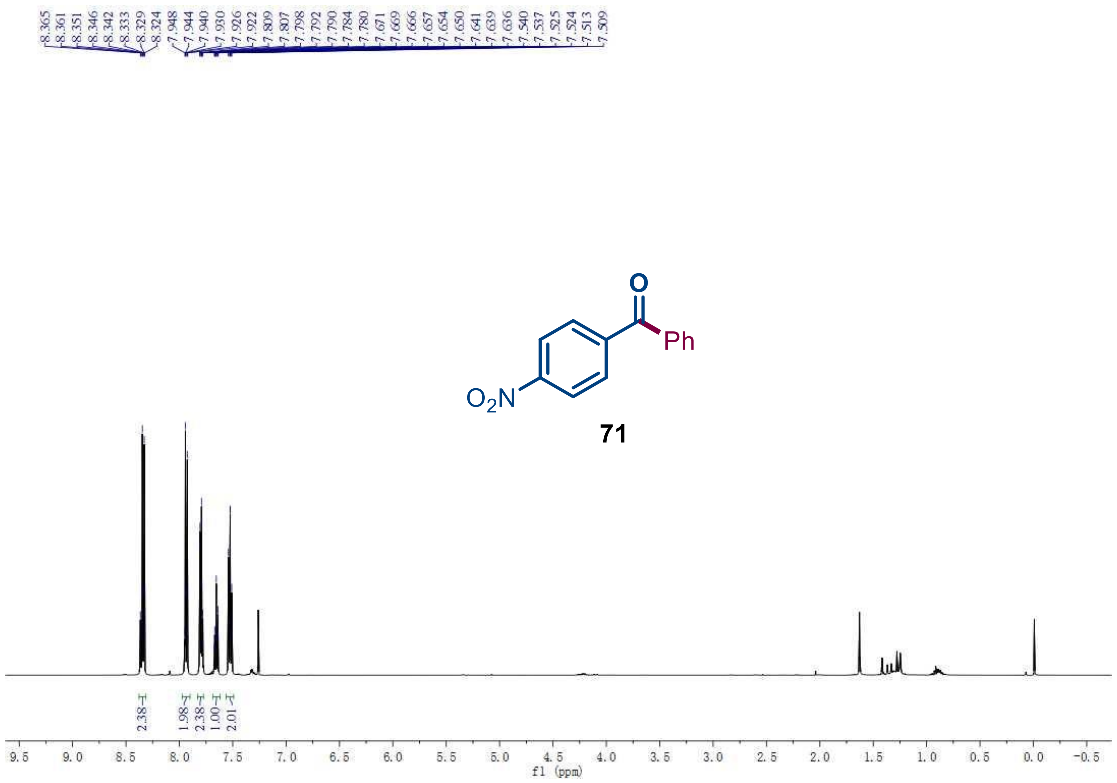

Supplementary Figure 184. ^{1}H NMR Spectrum of Compound **71**

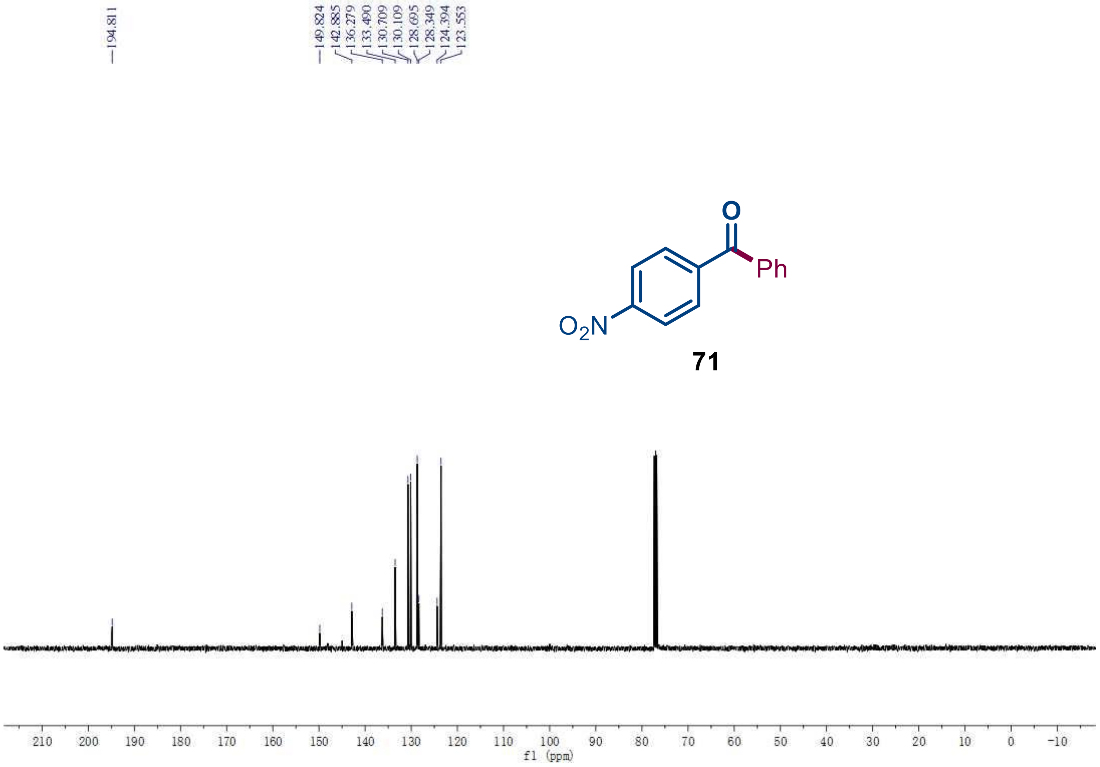

Supplementary Figure 185. ^{13}C NMR Spectrum of Compound **71**

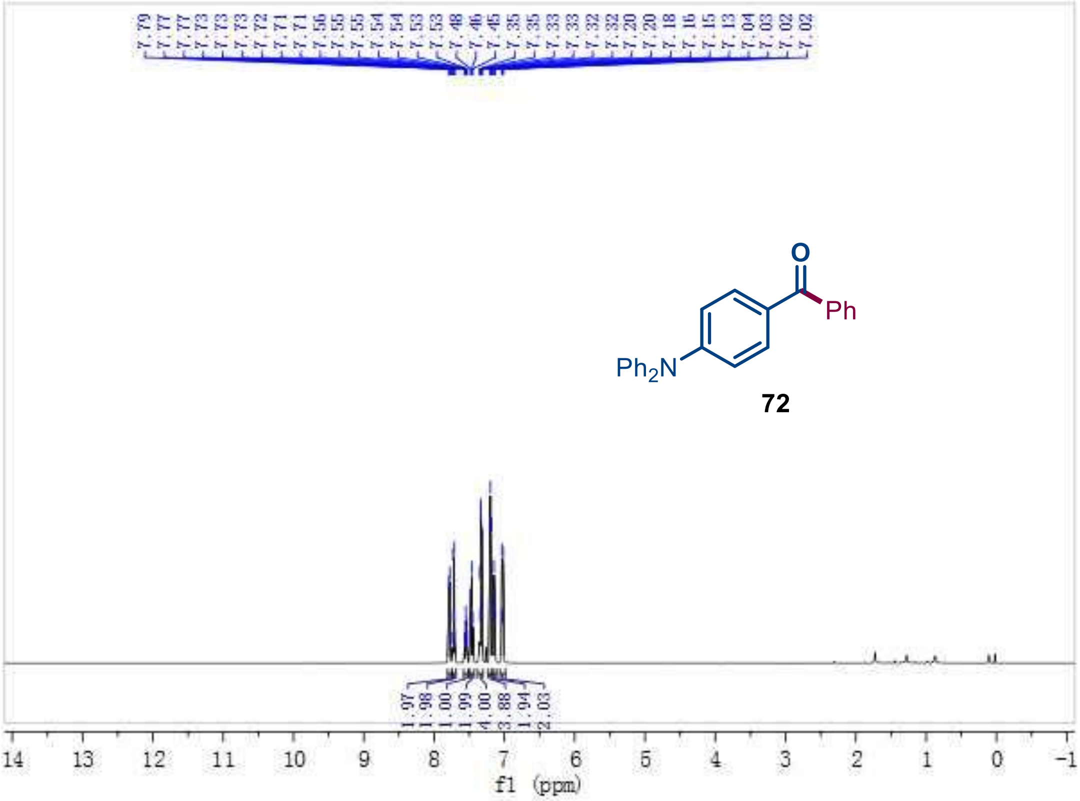

Supplementary Figure 186. ^{1}H NMR Spectrum of Compound **72**

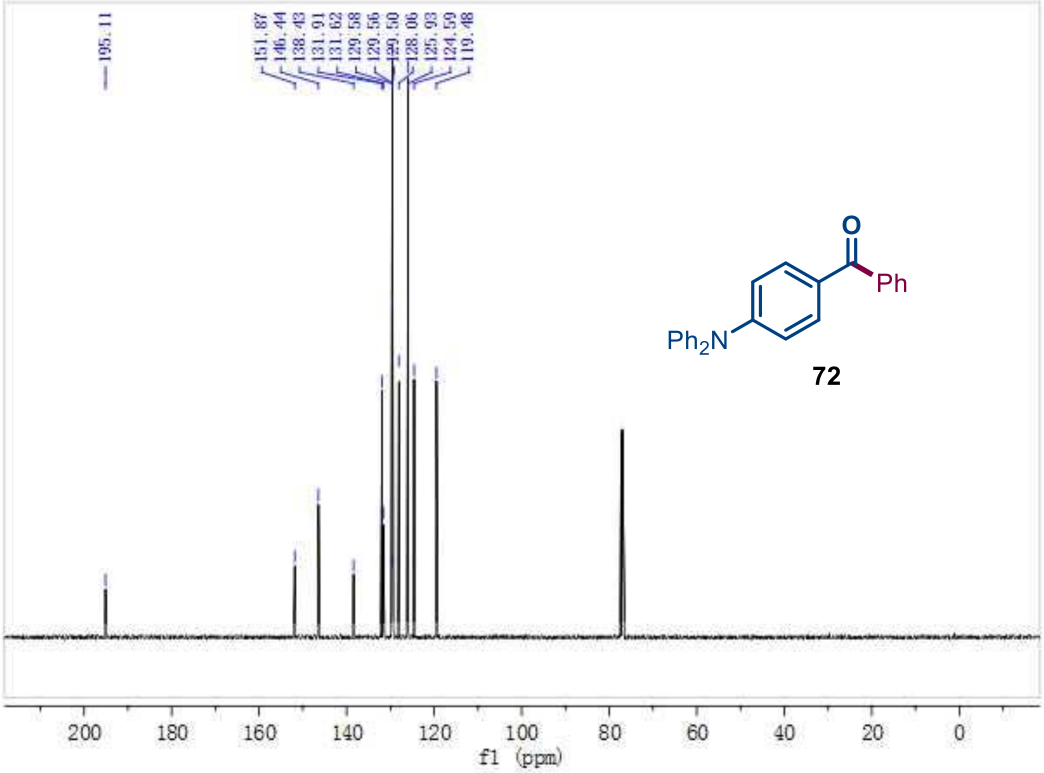

Supplementary Figure 187. ^{13}C NMR Spectrum of Compound **72**

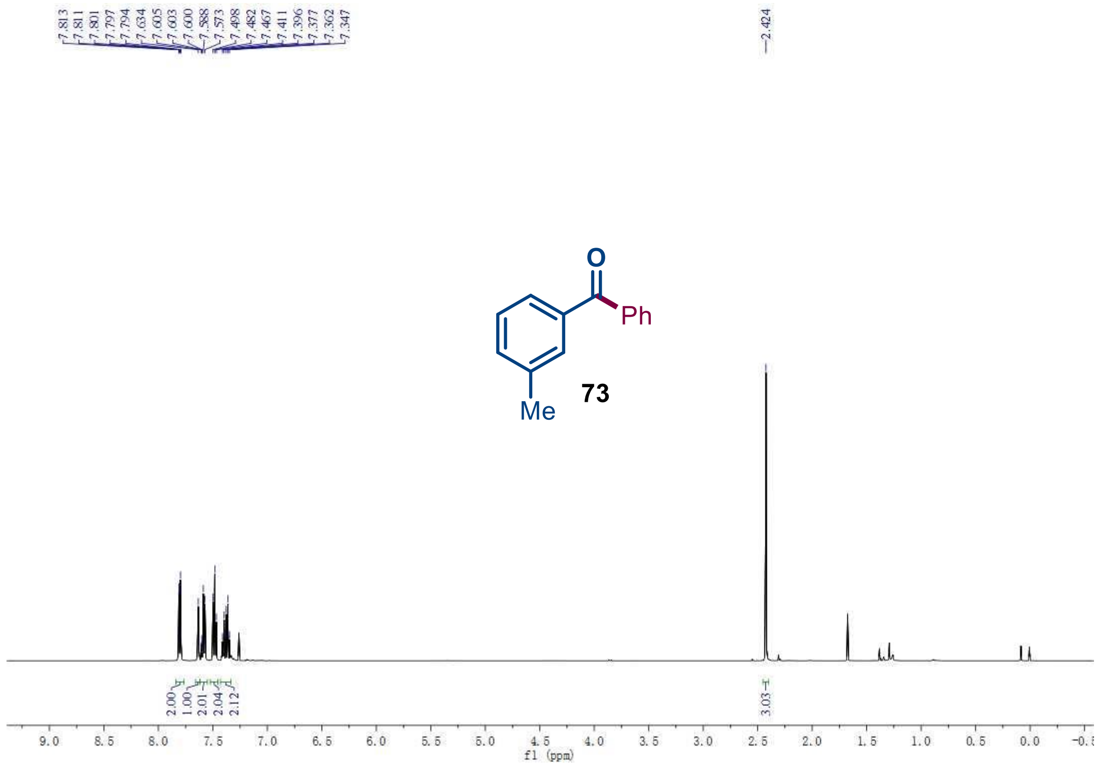

Supplementary Figure 188. ^{1}H NMR Spectrum of Compound **73**

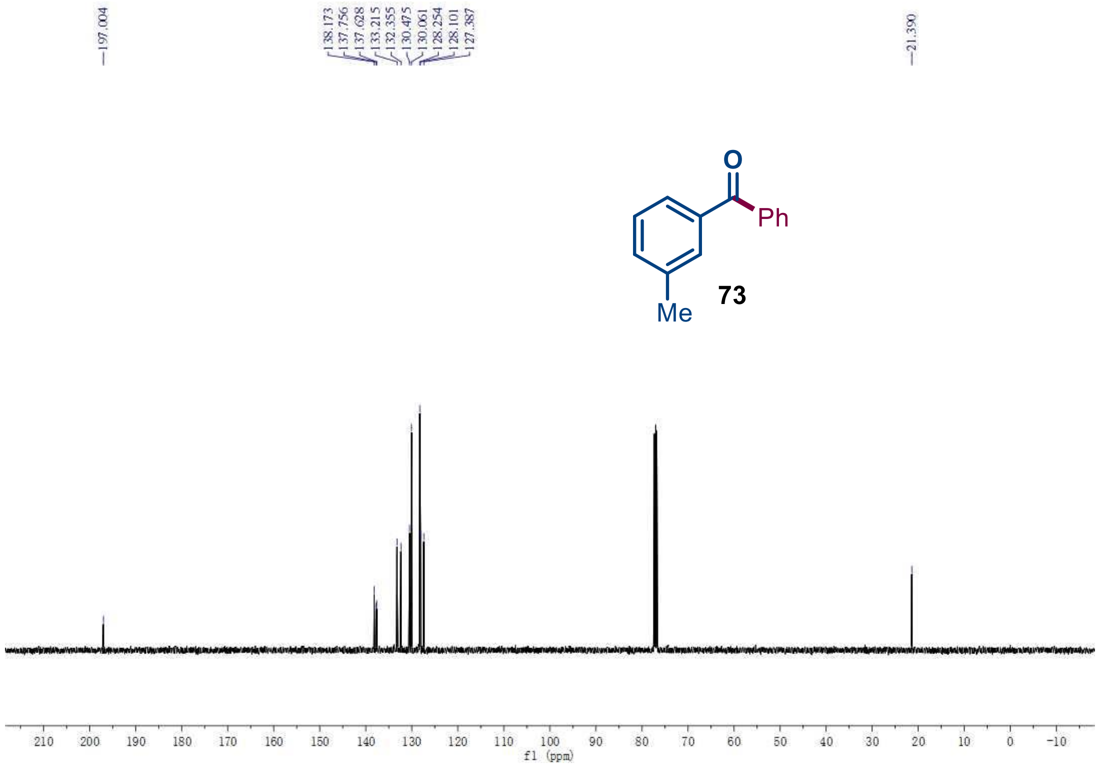

Supplementary Figure 189. ^{13}C NMR Spectrum of Compound **73**

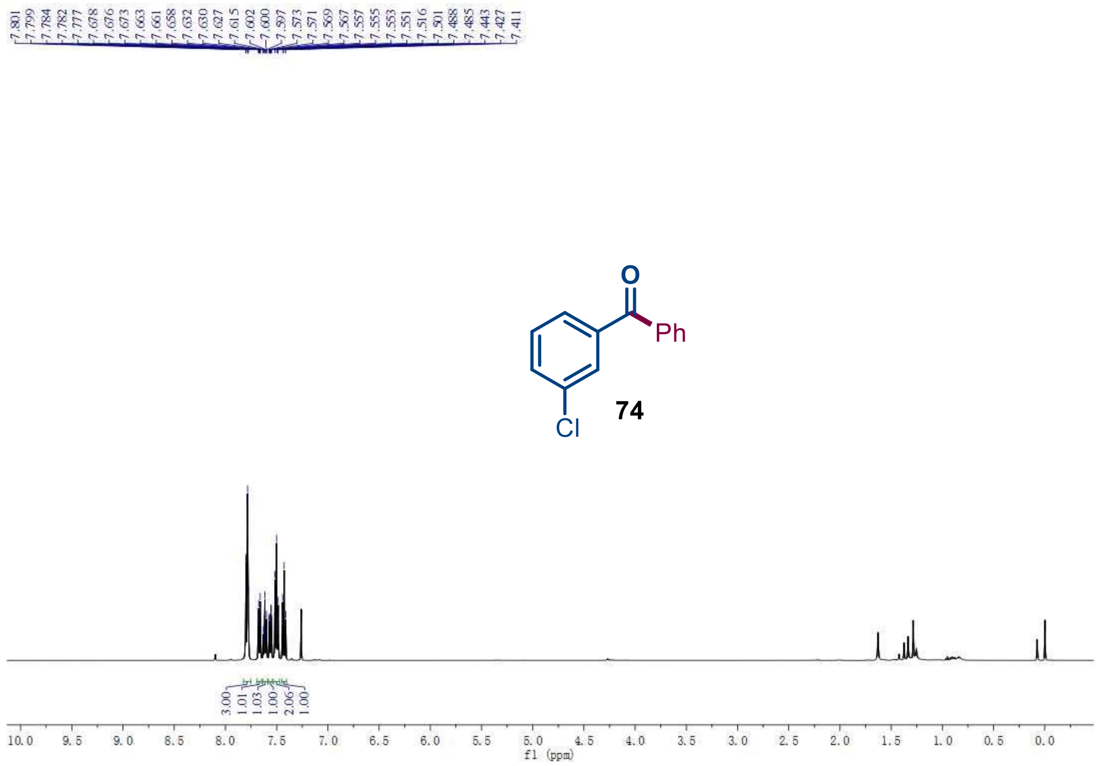

Supplementary Figure 190. ^{1}H NMR Spectrum of Compound **74**

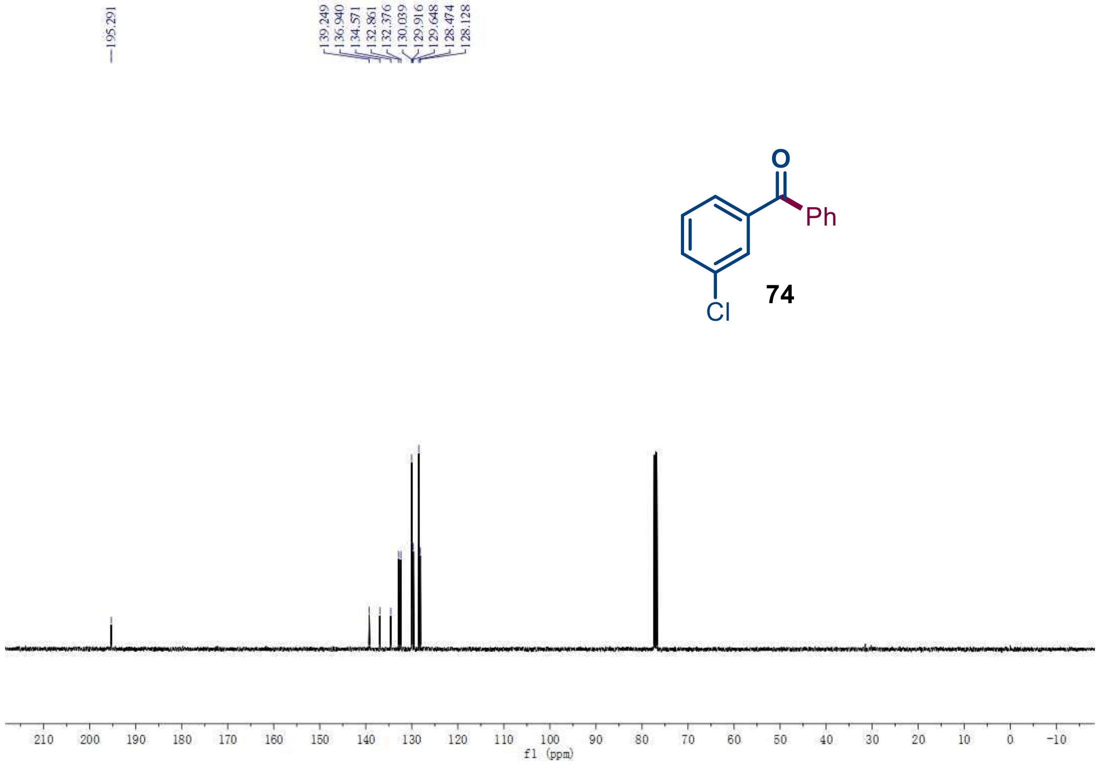

Supplementary Figure 191. ^{13}C NMR Spectrum of Compound **74**

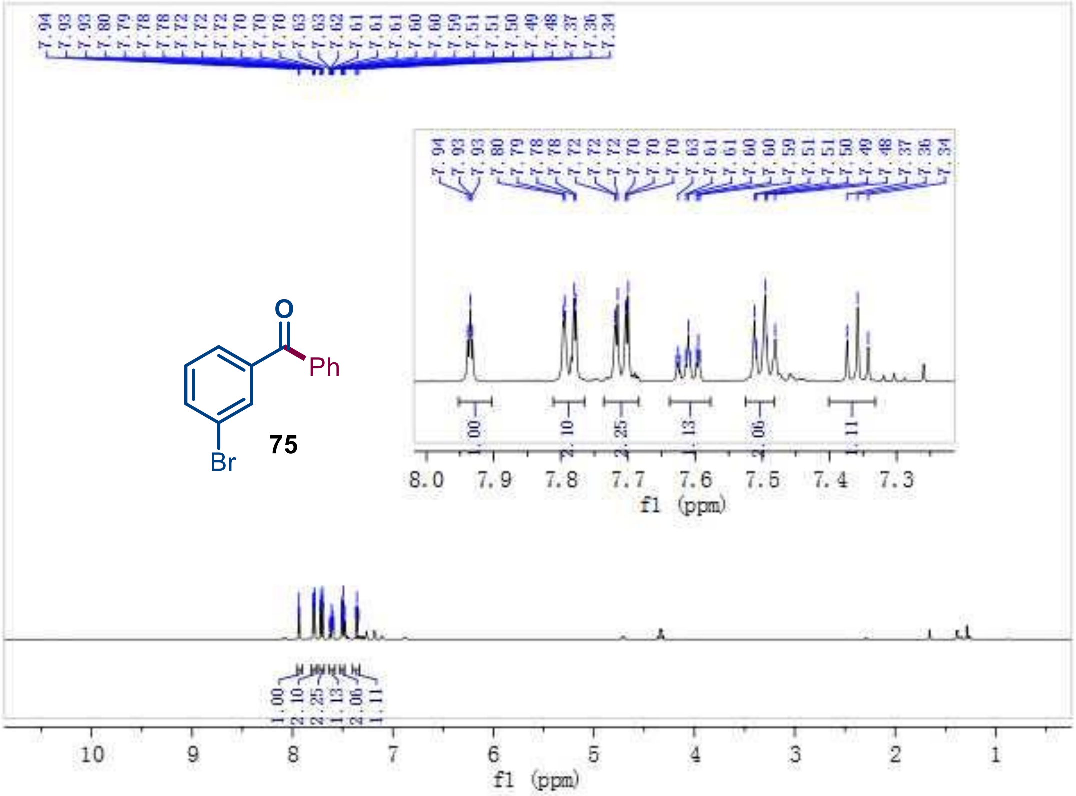

Supplementary Figure 192. ^{1}H NMR Spectrum of Compound **75**

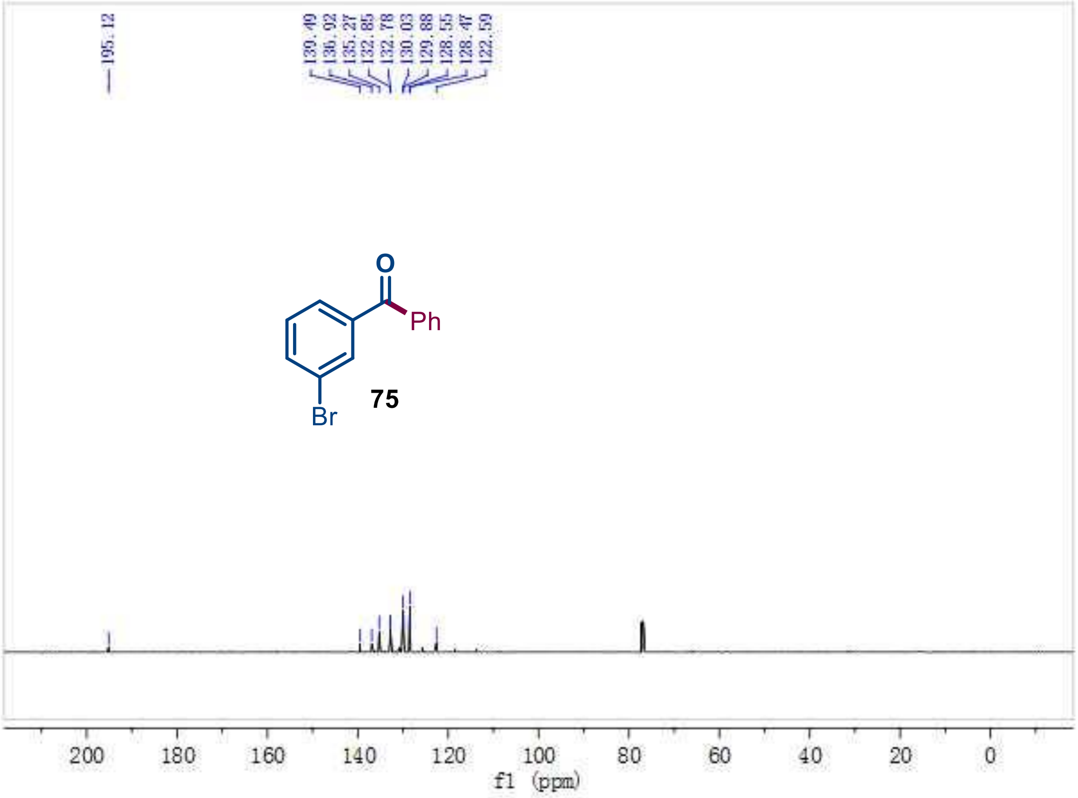

Supplementary Figure 193. ^{13}C NMR Spectrum of Compound **75**

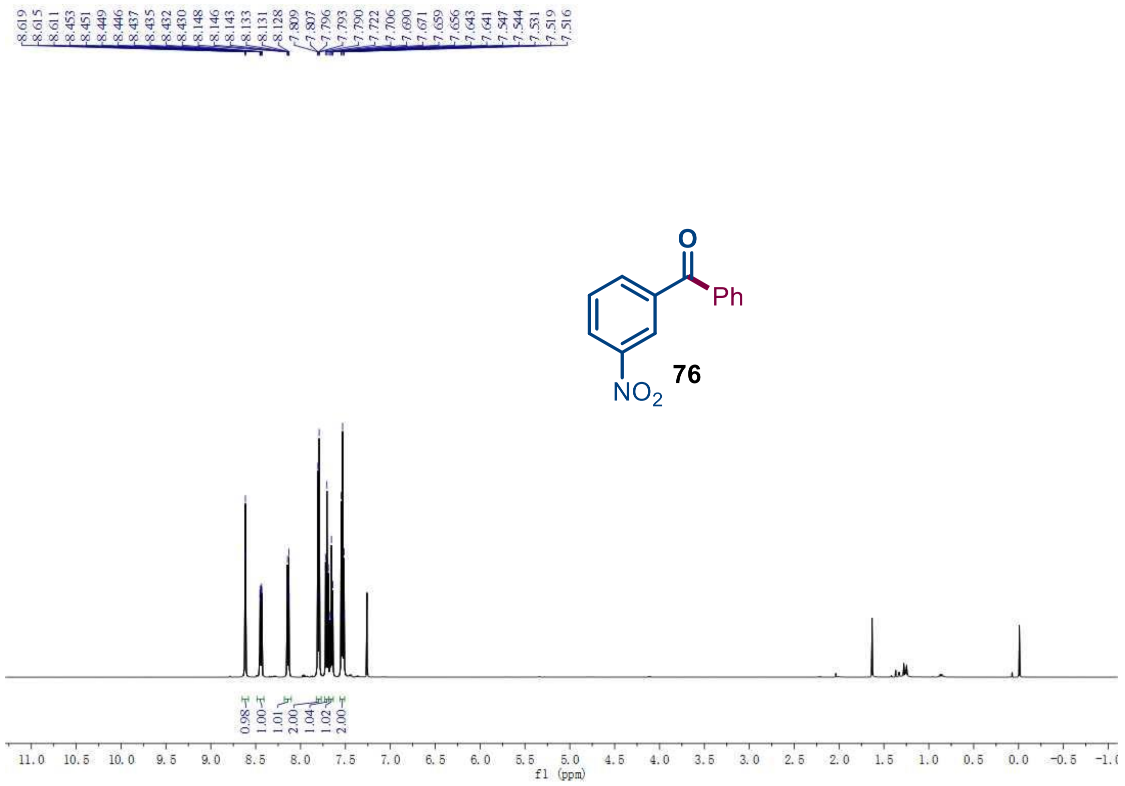

Supplementary Figure 194. ^{1}H NMR Spectrum of Compound **76**

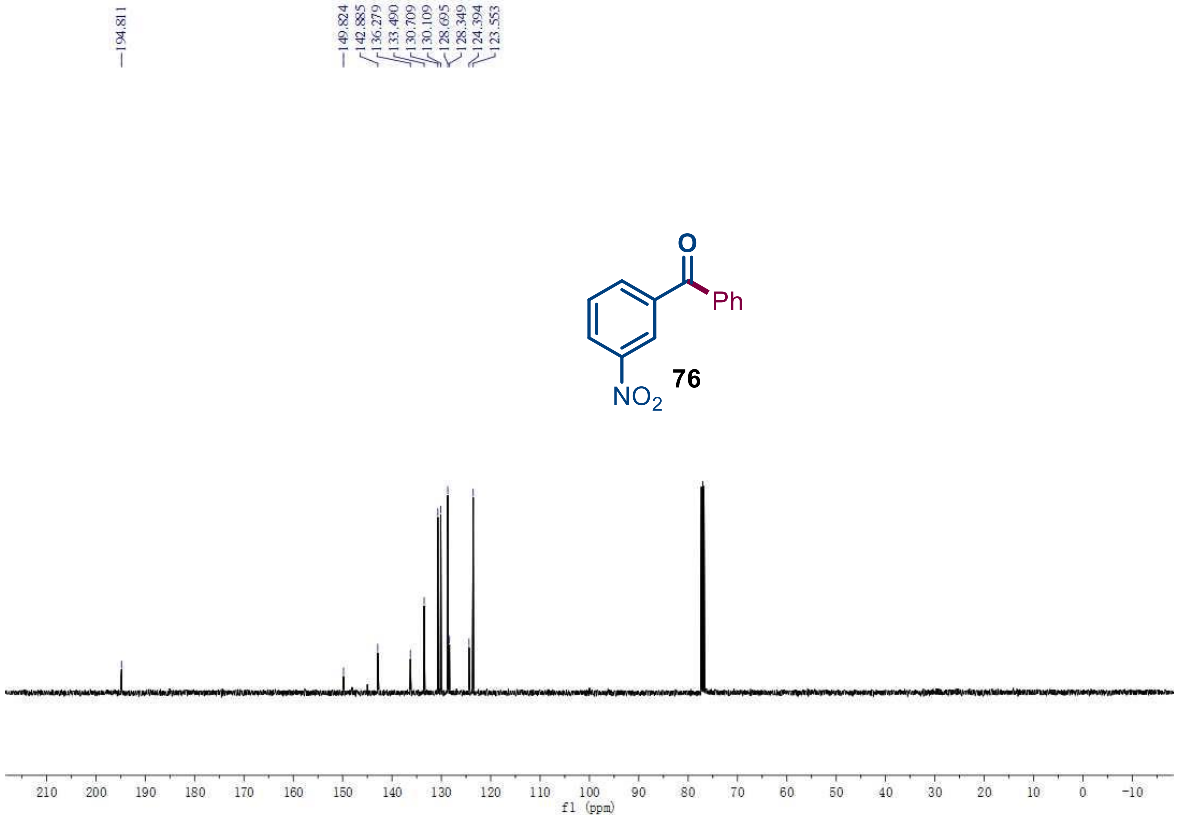

Supplementary Figure 195. ^{13}C NMR Spectrum of Compound **76**

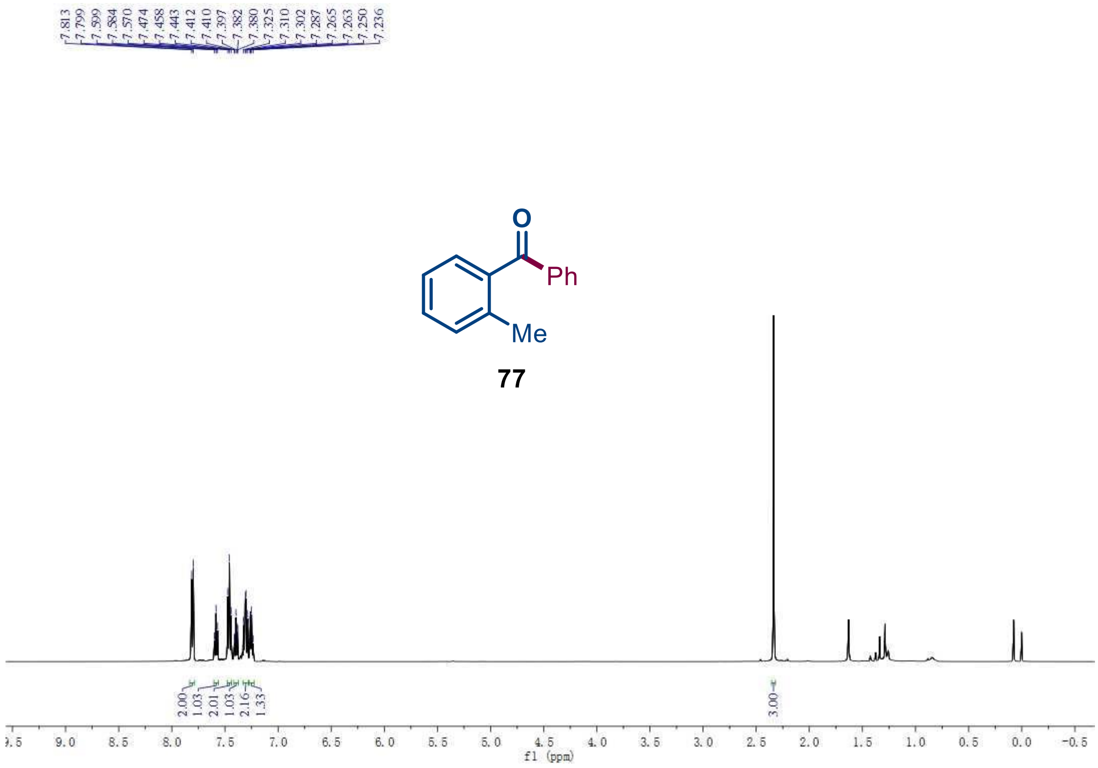

Supplementary Figure 196. ^{1}H NMR Spectrum of Compound **77**

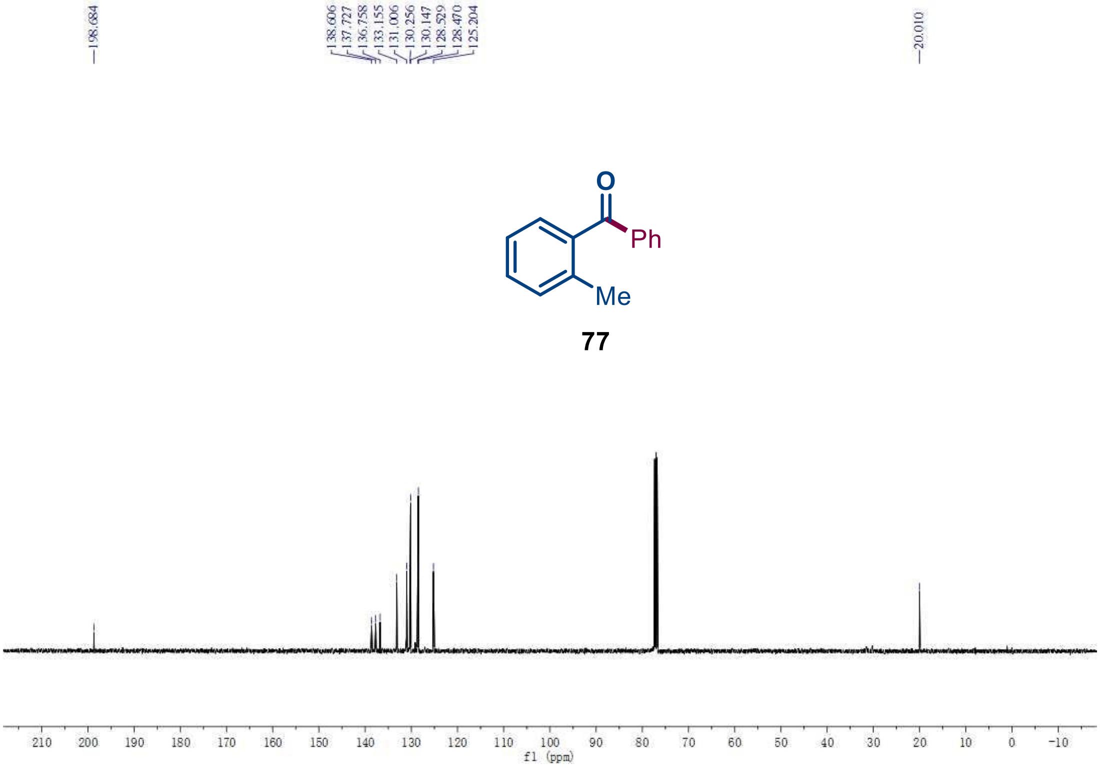

Supplementary Figure 197. ^{13}C NMR Spectrum of Compound **77**

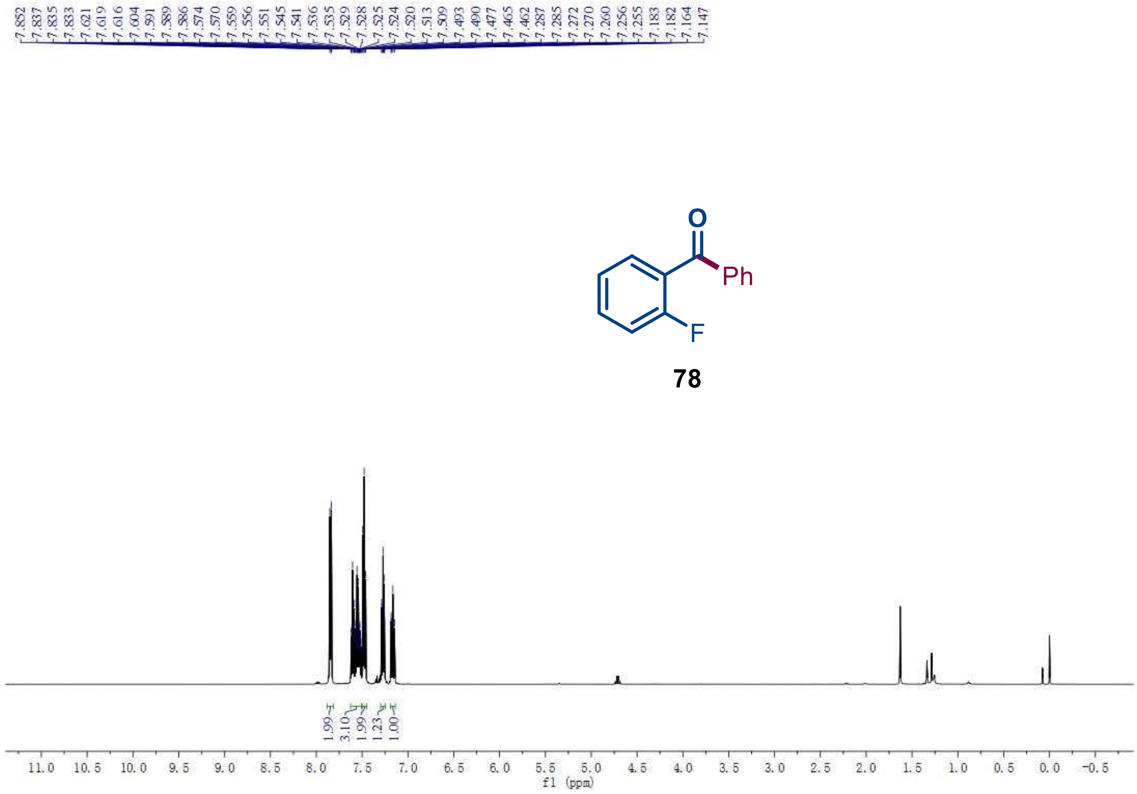

Supplementary Figure 198. ^{1}H NMR Spectrum of Compound **78**

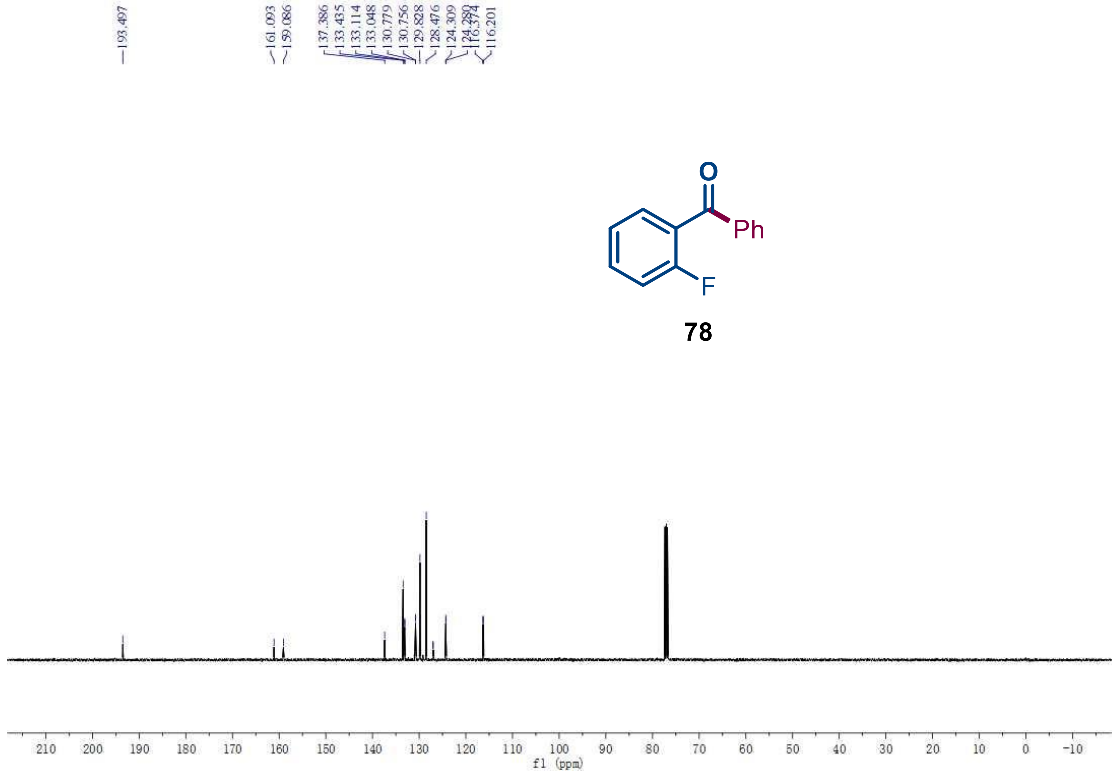

Supplementary Figure 199. ^{13}C NMR Spectrum of Compound **78**

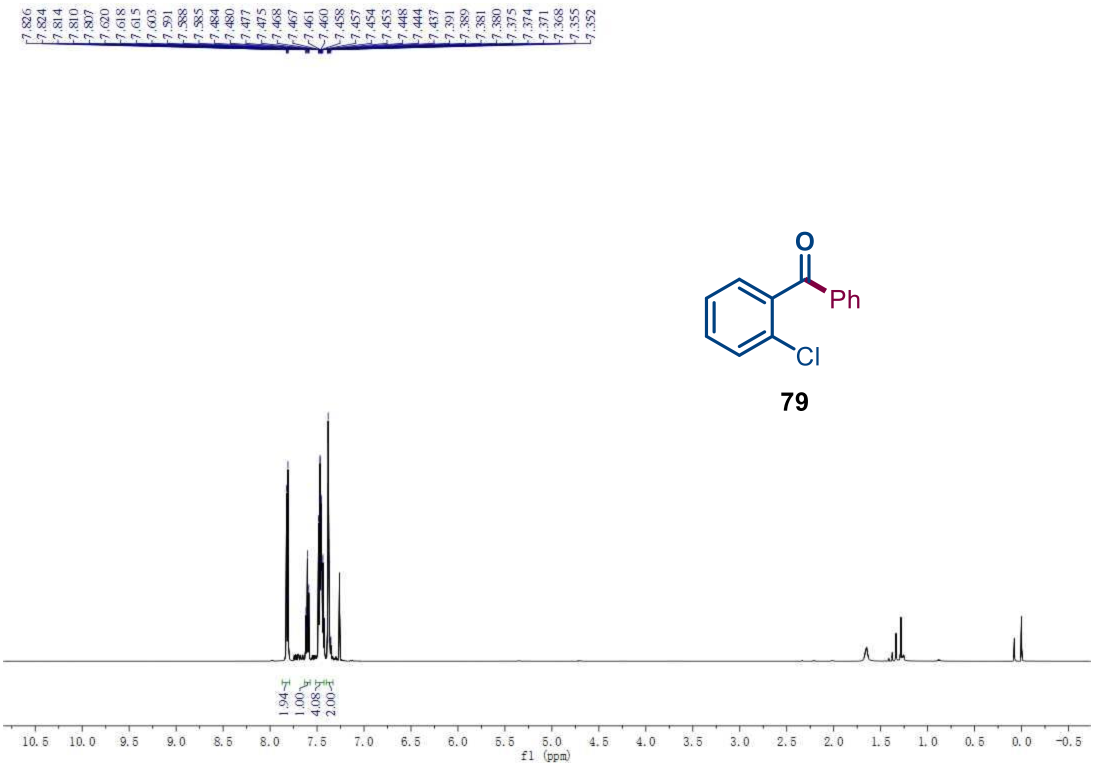

Supplementary Figure 200. ^{1}H NMR Spectrum of Compound **79**

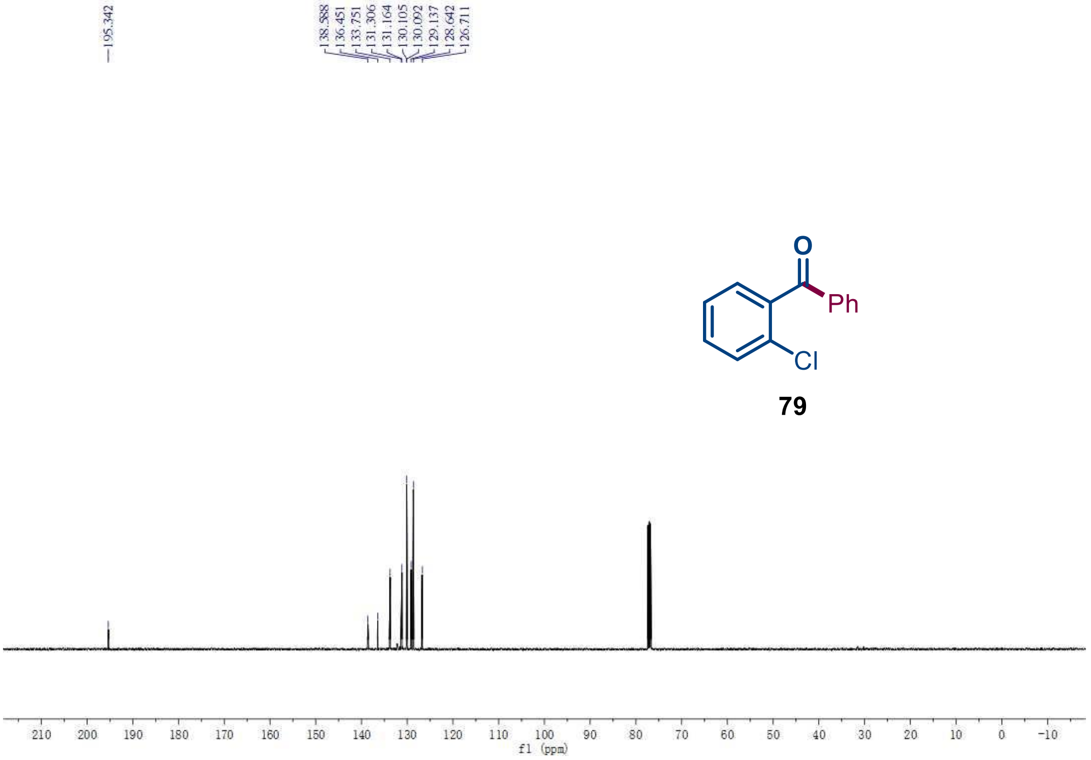

Supplementary Figure 201. ^{13}C NMR Spectrum of Compound **79**

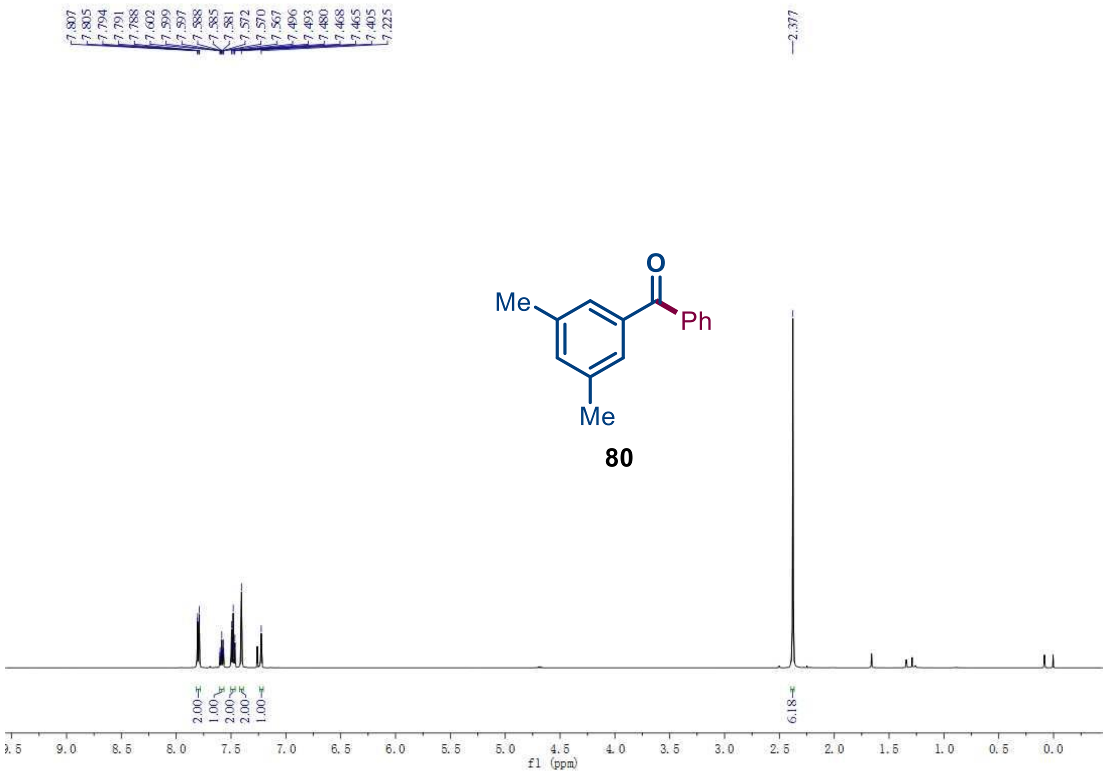

Supplementary Figure 202. ^{1}H NMR Spectrum of Compound **80**

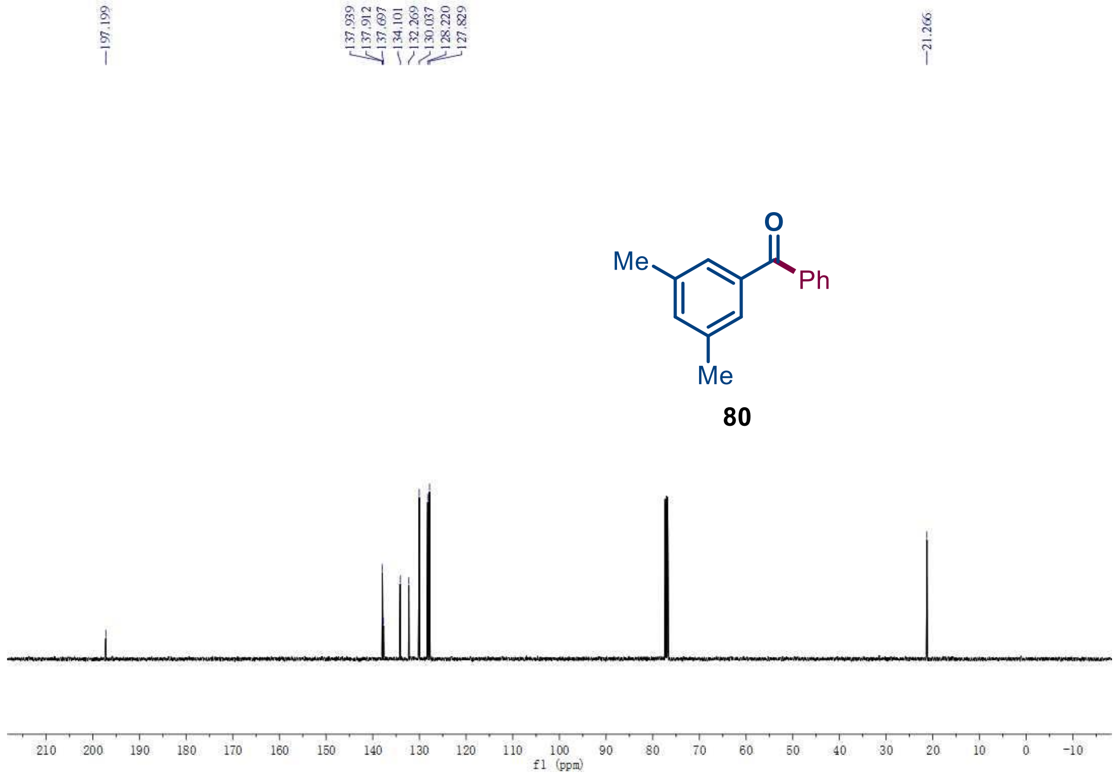

Supplementary Figure 203. ^{13}C NMR Spectrum of Compound **80**

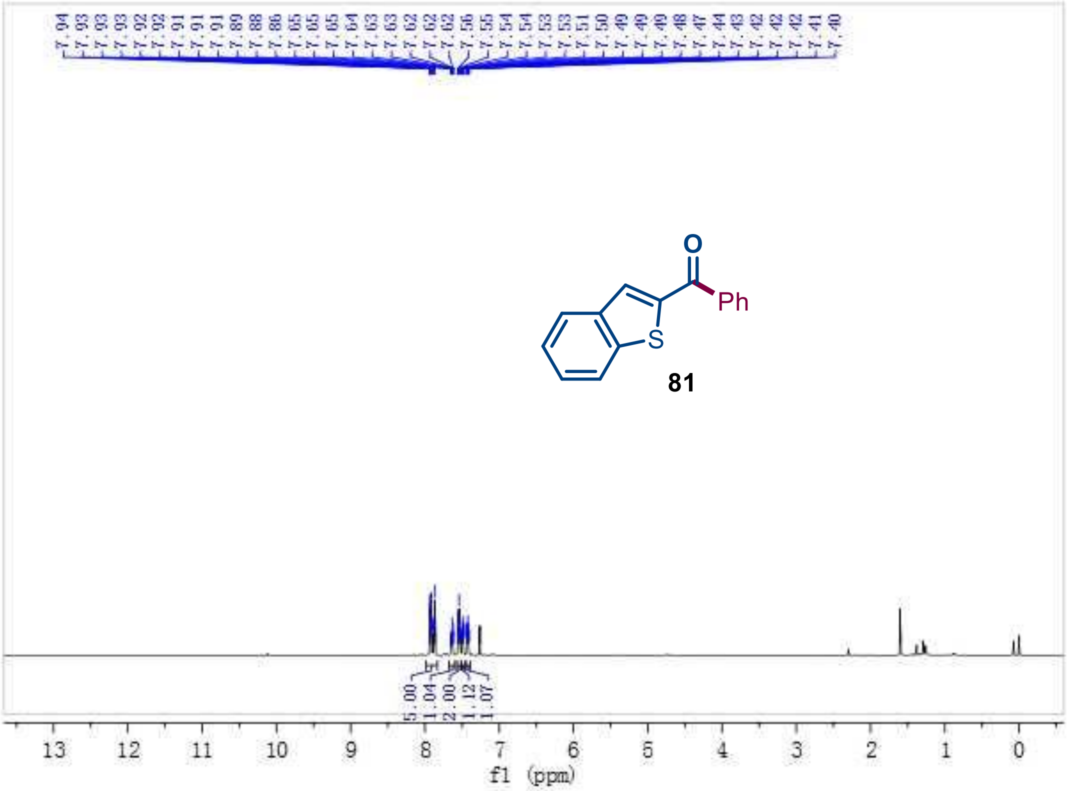

Supplementary Figure 204. ^{1}H NMR Spectrum of Compound **81**

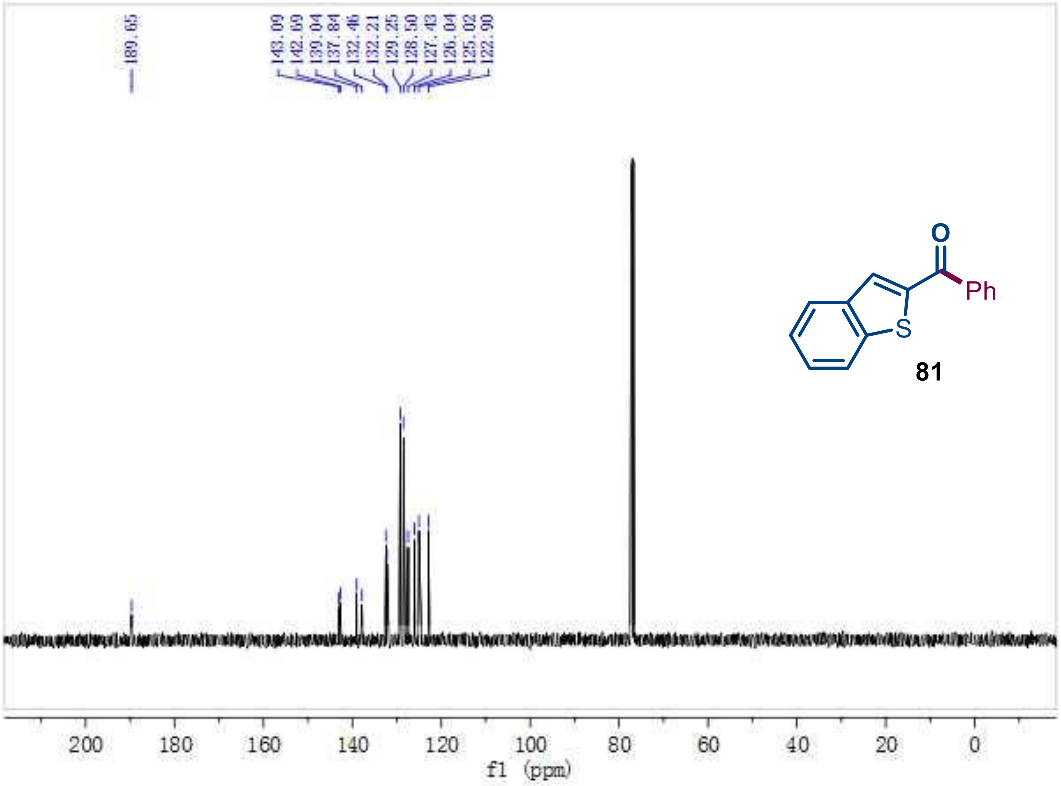

Supplementary Figure 205. ^{13}C NMR Spectrum of Compound **81**

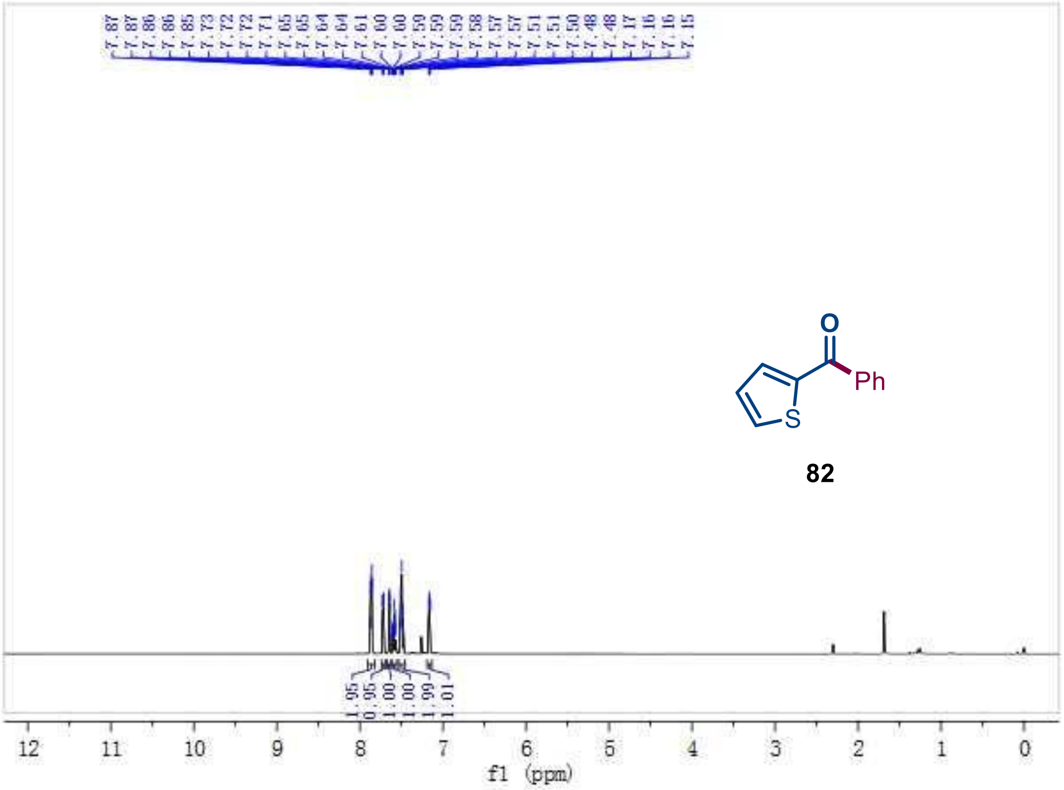

Supplementary Figure 206. ^{1}H NMR Spectrum of Compound **82**

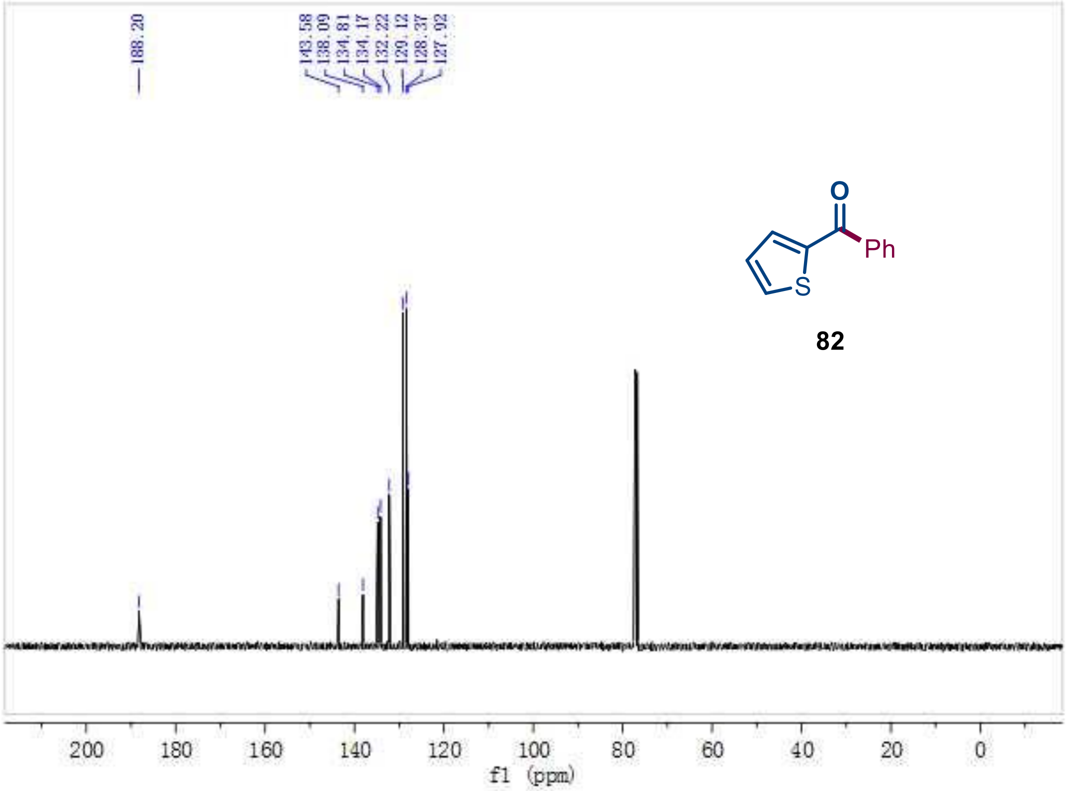

Supplementary Figure 207. ^{13}C NMR Spectrum of Compound **82**

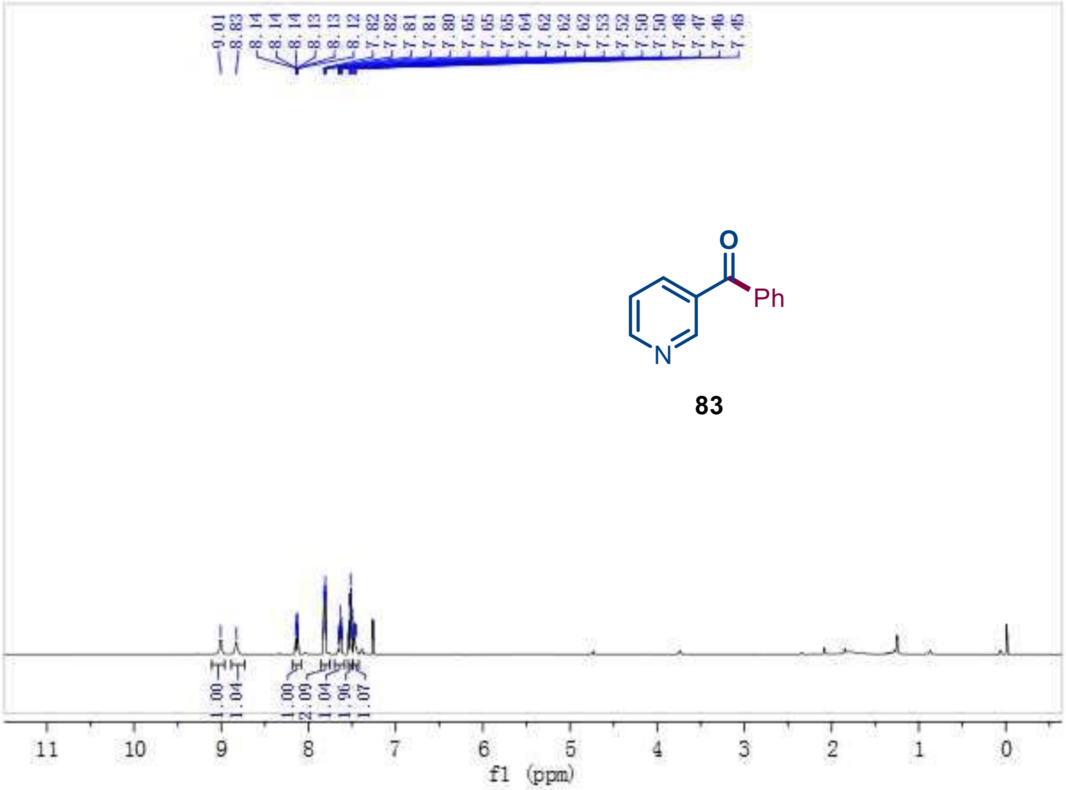

Supplementary Figure 208. ^{1}H NMR Spectrum of Compound **83**

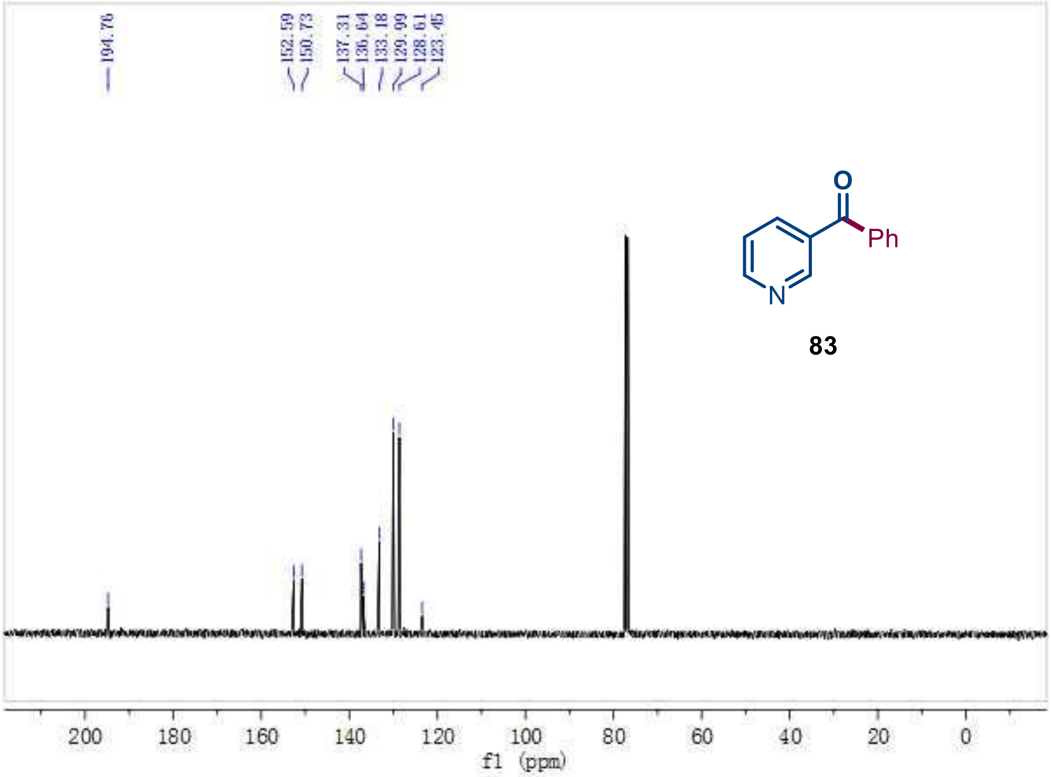

Supplementary Figure 209. ^{13}C NMR Spectrum of Compound **83**

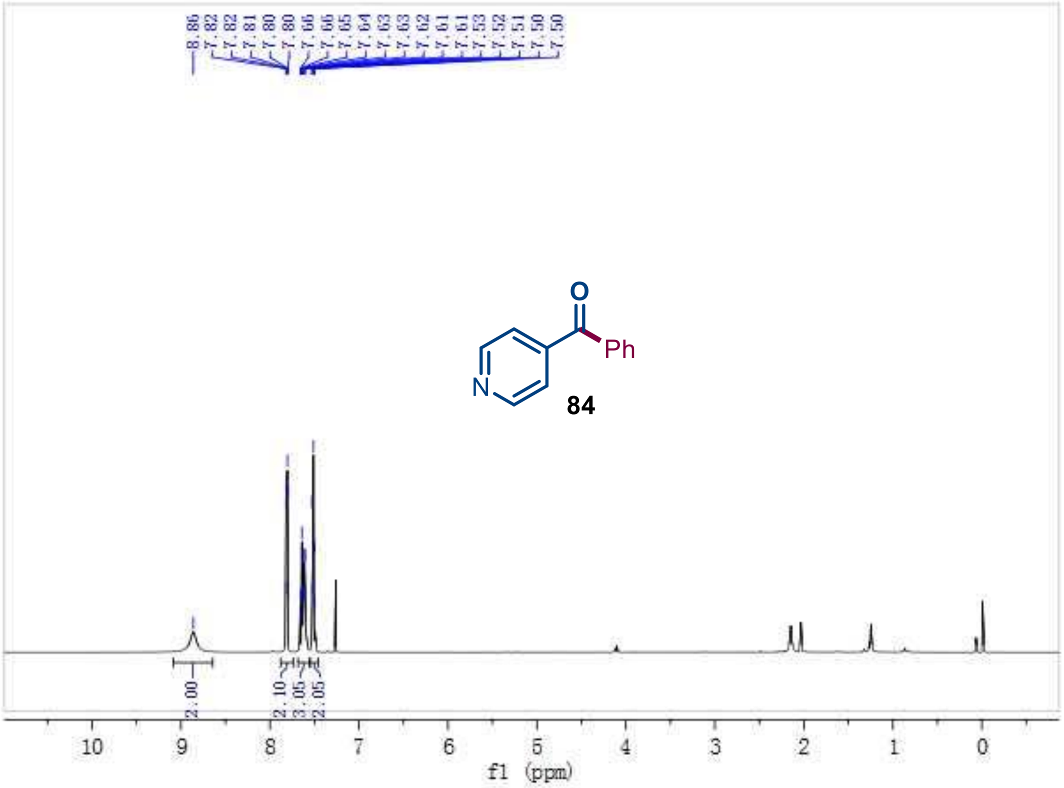

Supplementary Figure 210. ^{1}H NMR Spectrum of Compound **84**

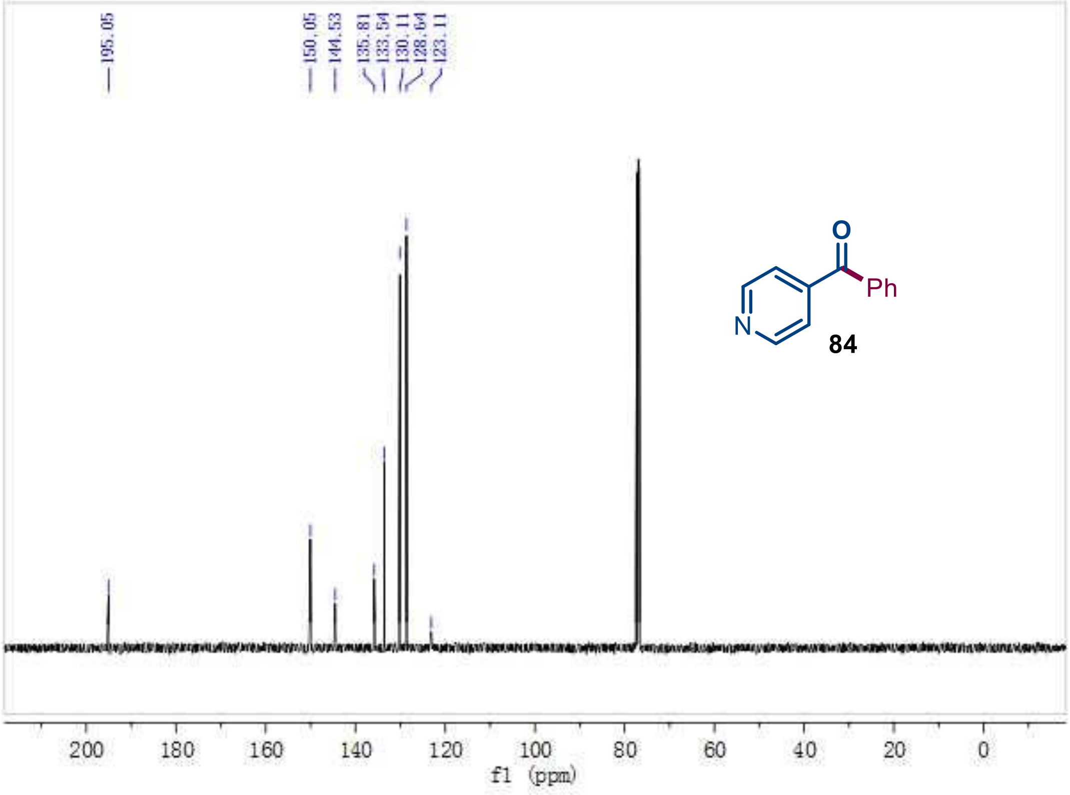

Supplementary Figure 211. ^{13}C NMR Spectrum of Compound **84**

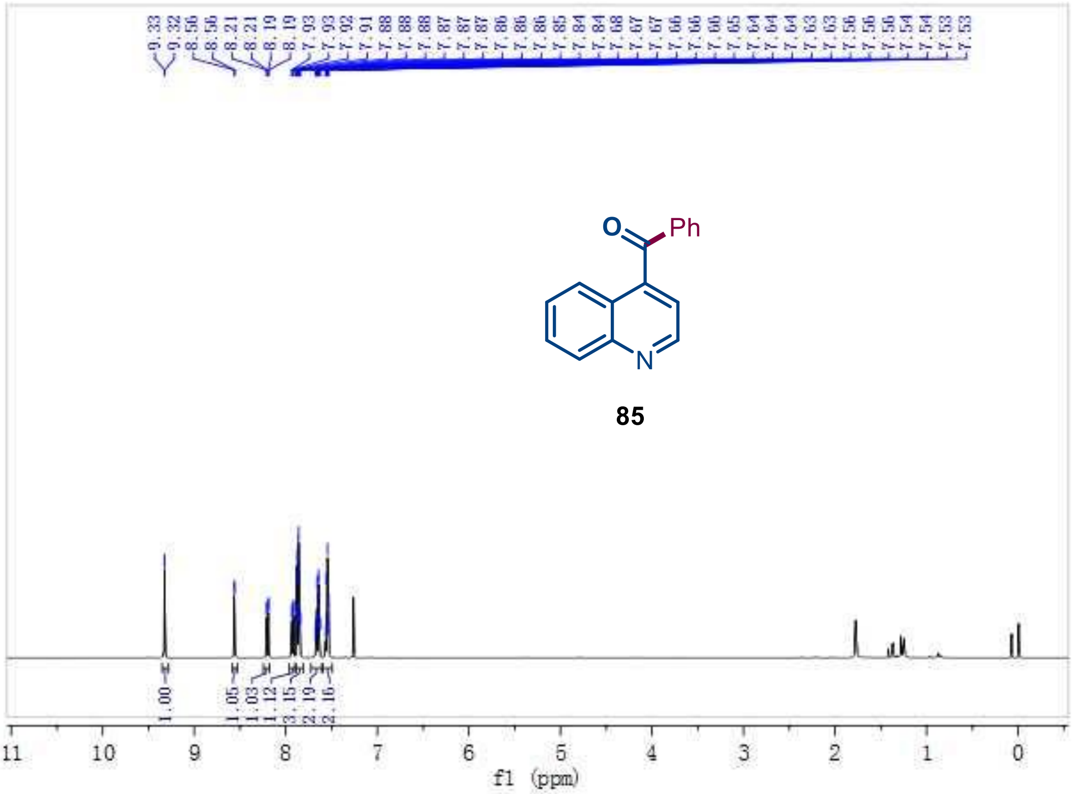

Supplementary Figure 212. ^{1}H NMR Spectrum of Compound **85**

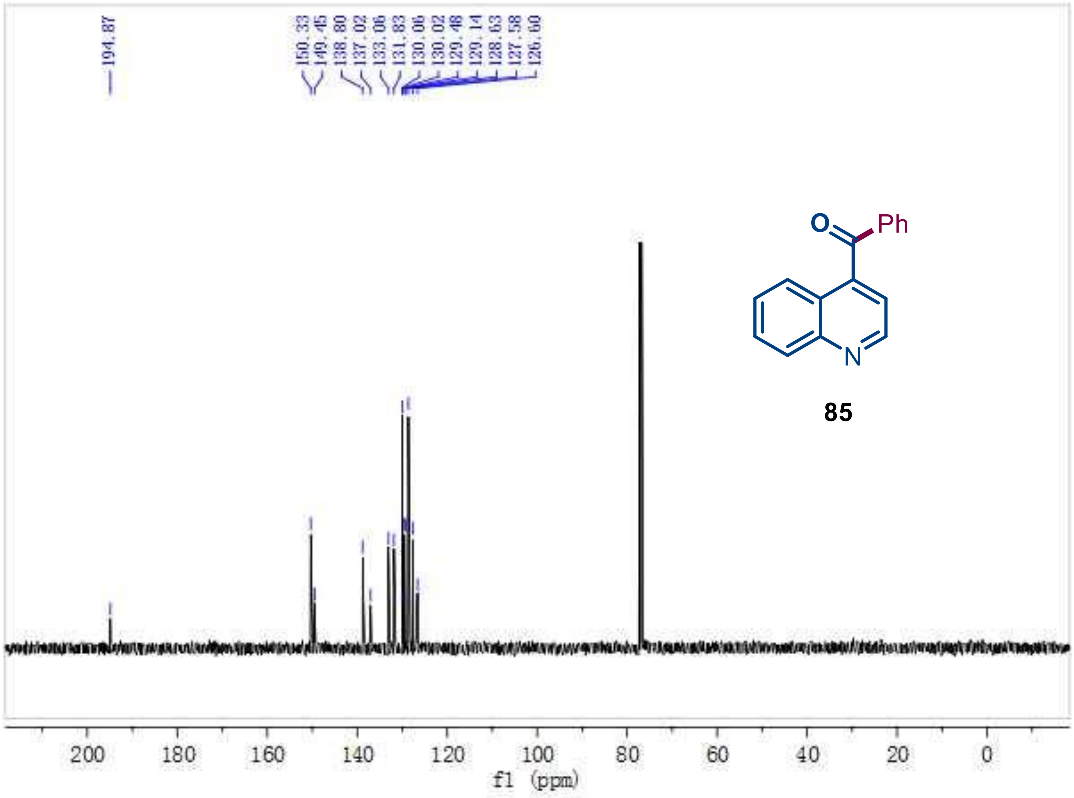

Supplementary Figure 213. ^{13}C NMR Spectrum of Compound **85**

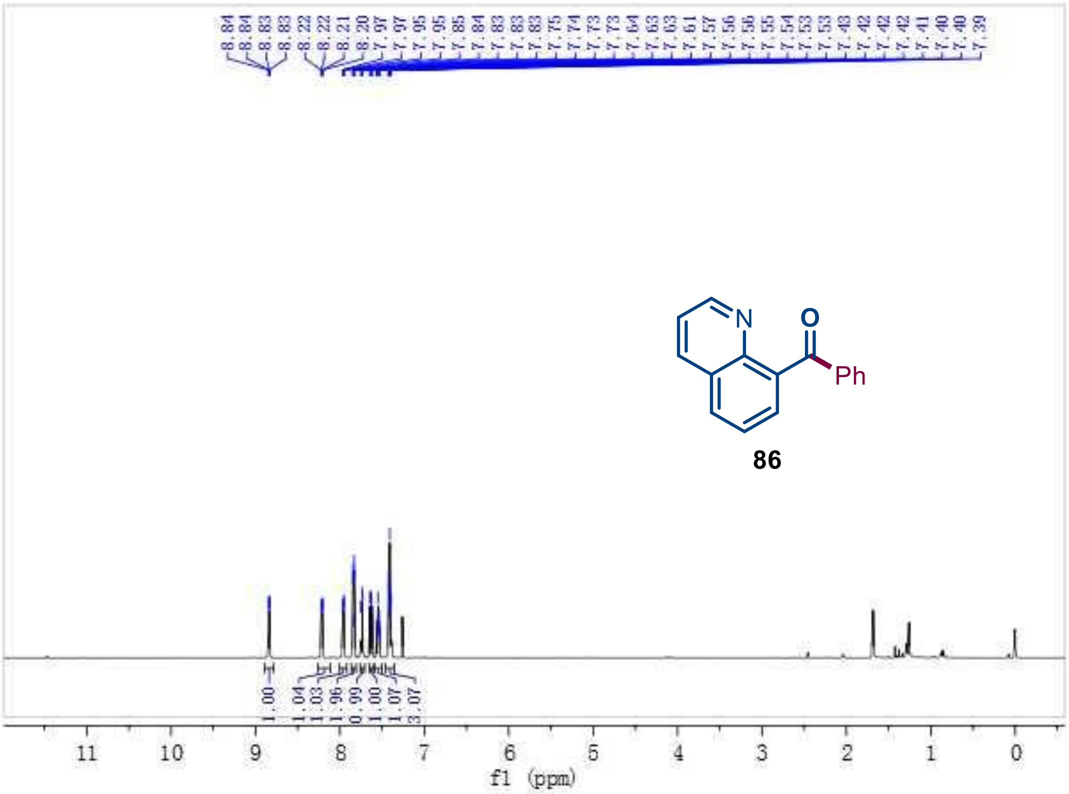

Supplementary Figure 214. ^{1}H NMR Spectrum of Compound **86**

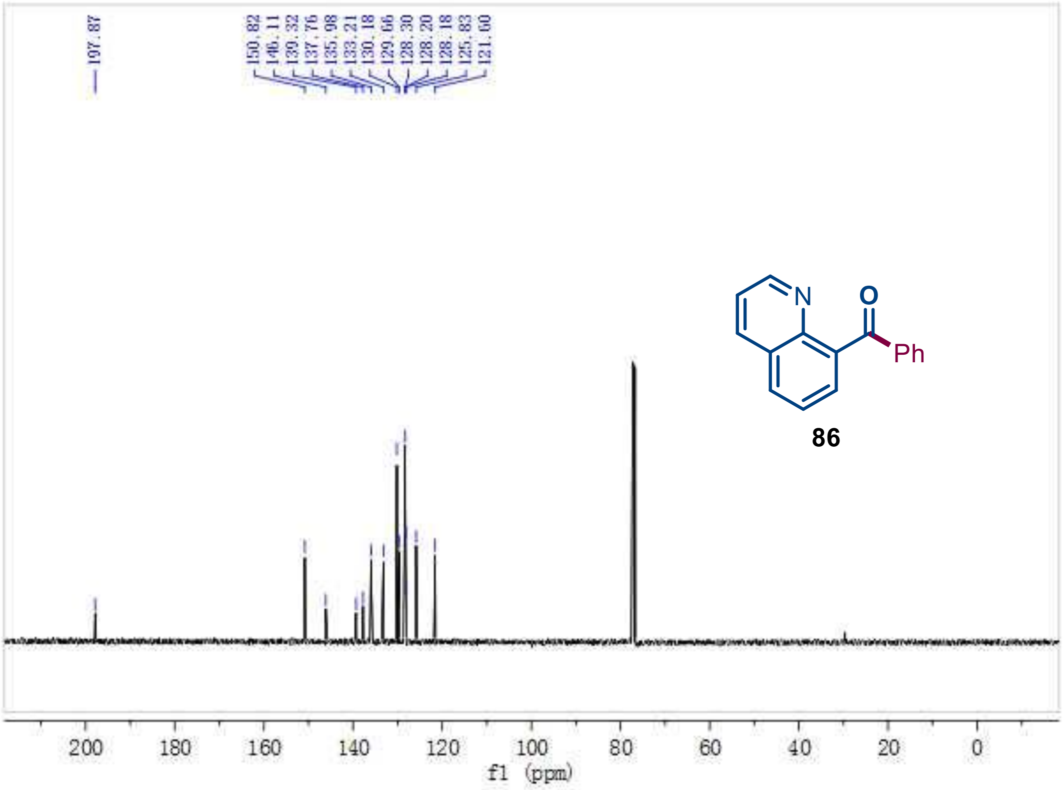

Supplementary Figure 215. ^{13}C NMR Spectrum of Compound **86**

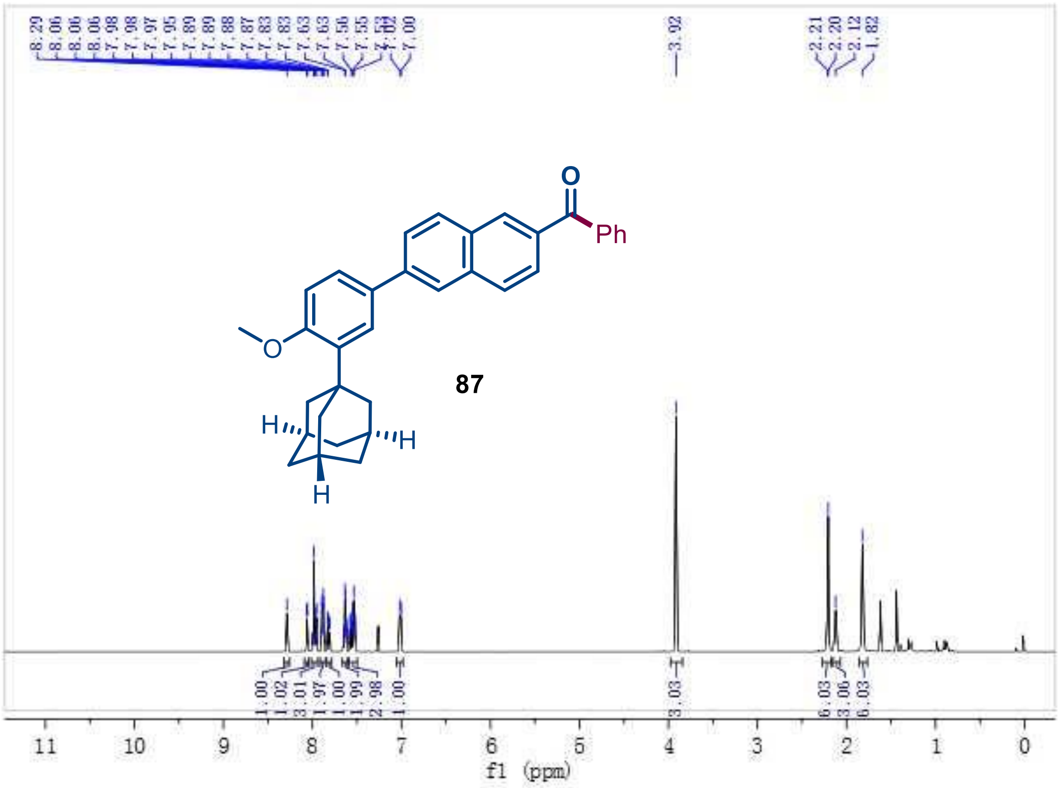

Supplementary Figure 216. ^{1}H NMR Spectrum of Compound **87**

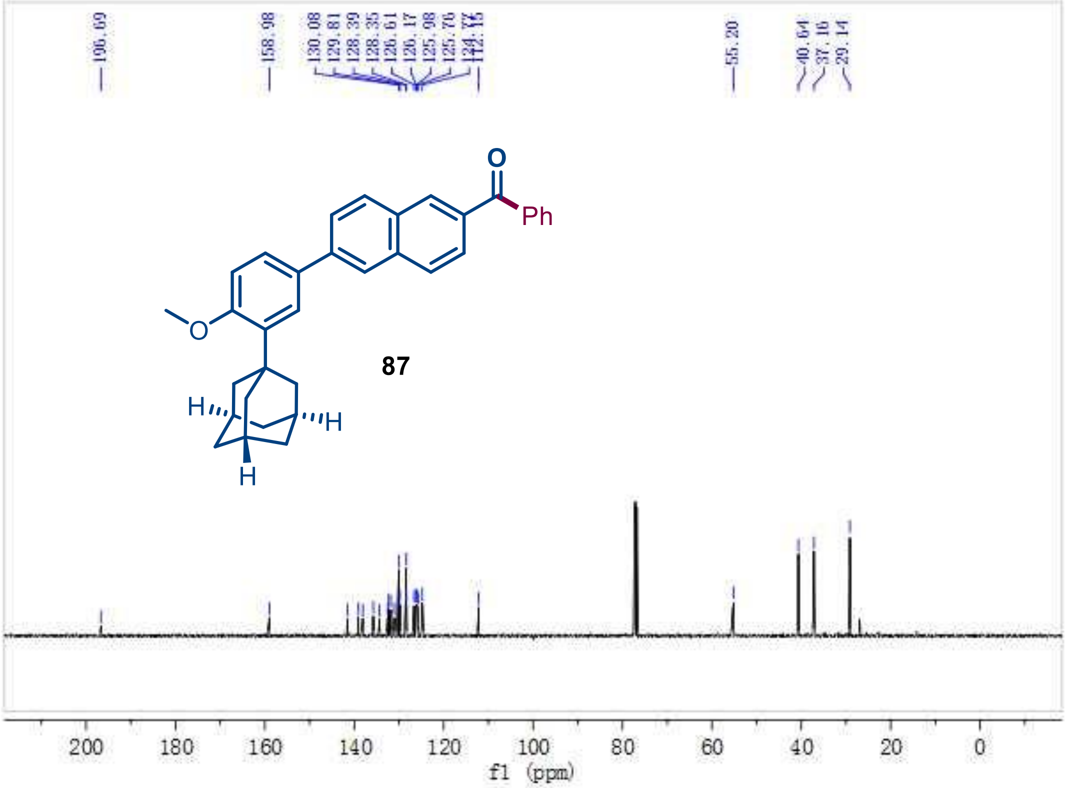

Supplementary Figure 217. ^{13}C NMR Spectrum of Compound **87**

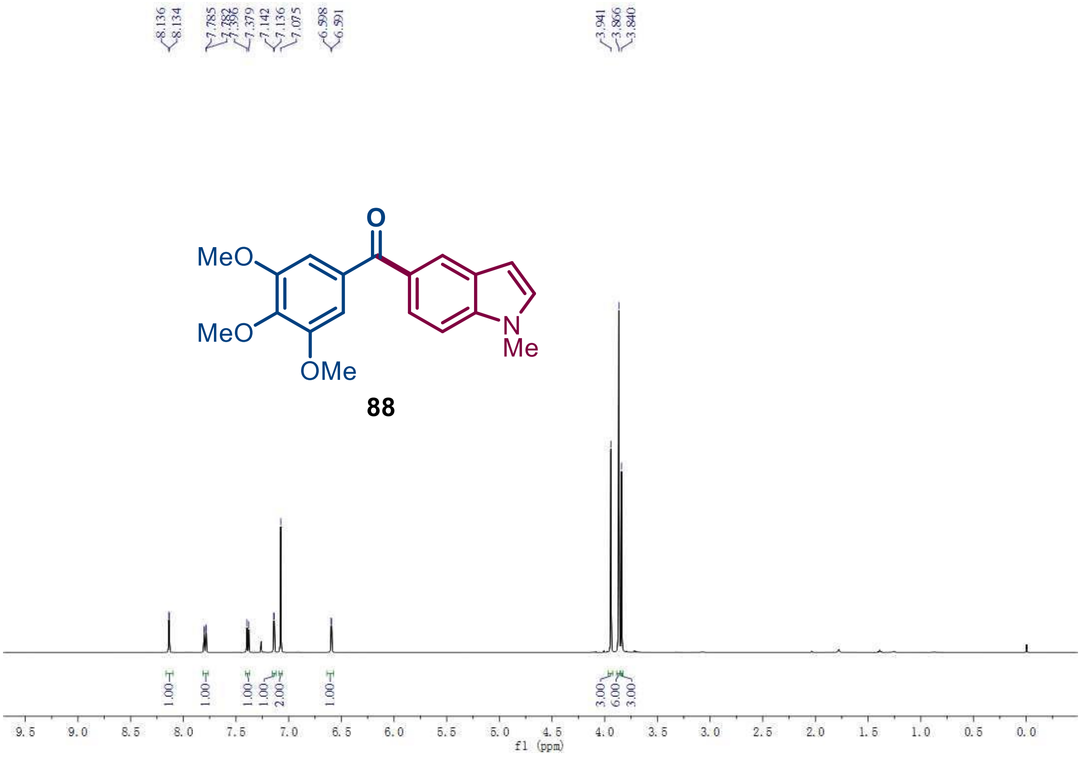

Supplementary Figure 218. ^{1}H NMR Spectrum of Compound **88**

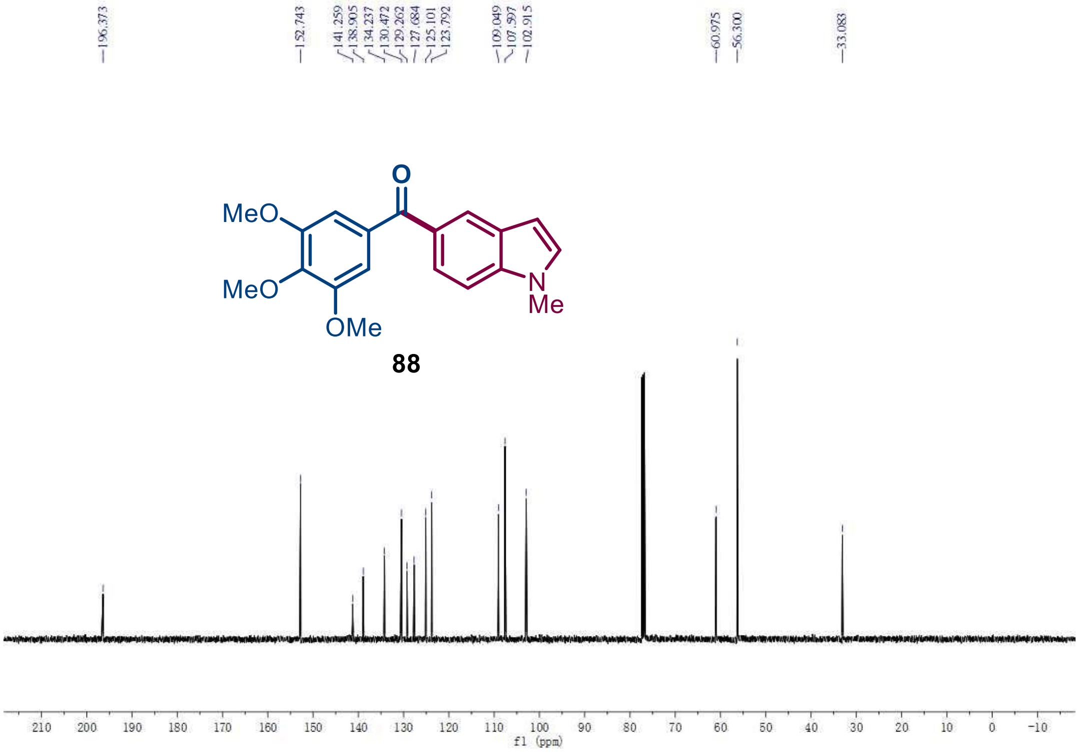

Supplementary Figure 219. ^{13}C NMR Spectrum of Compound **88**

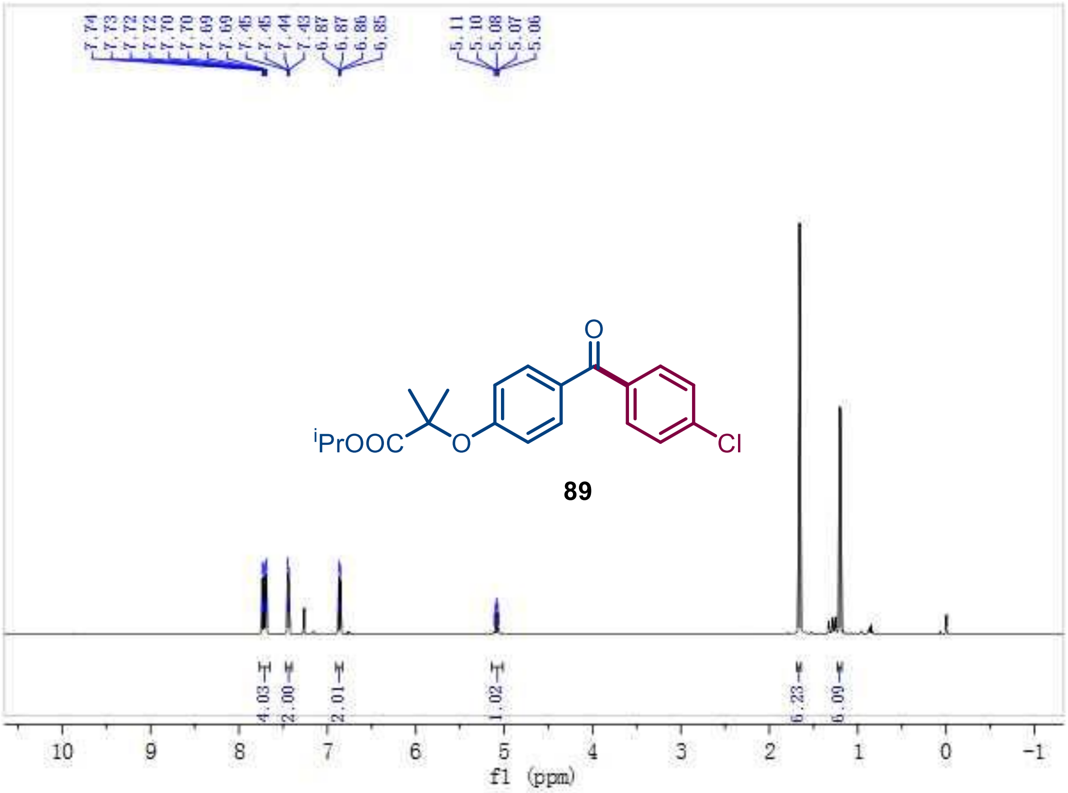

Supplementary Figure 220. ^{1}H NMR Spectrum of Compound **89**

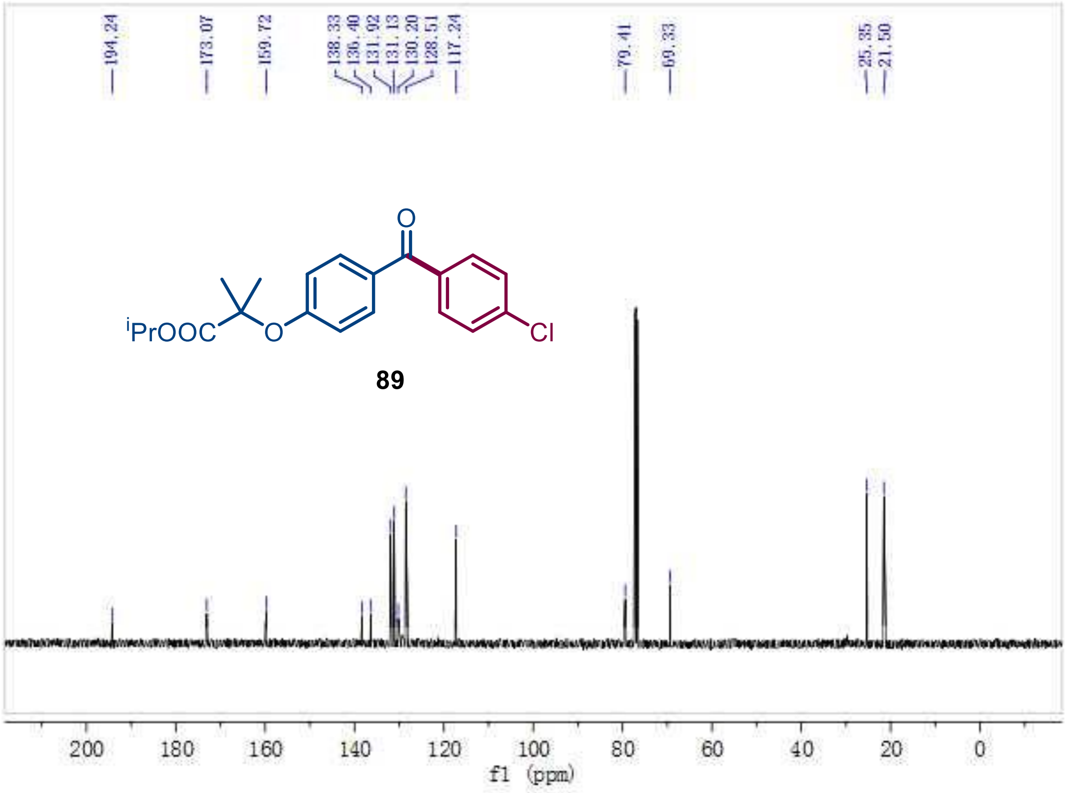

Supplementary Figure 221. ^{13}C NMR Spectrum of Compound **89**

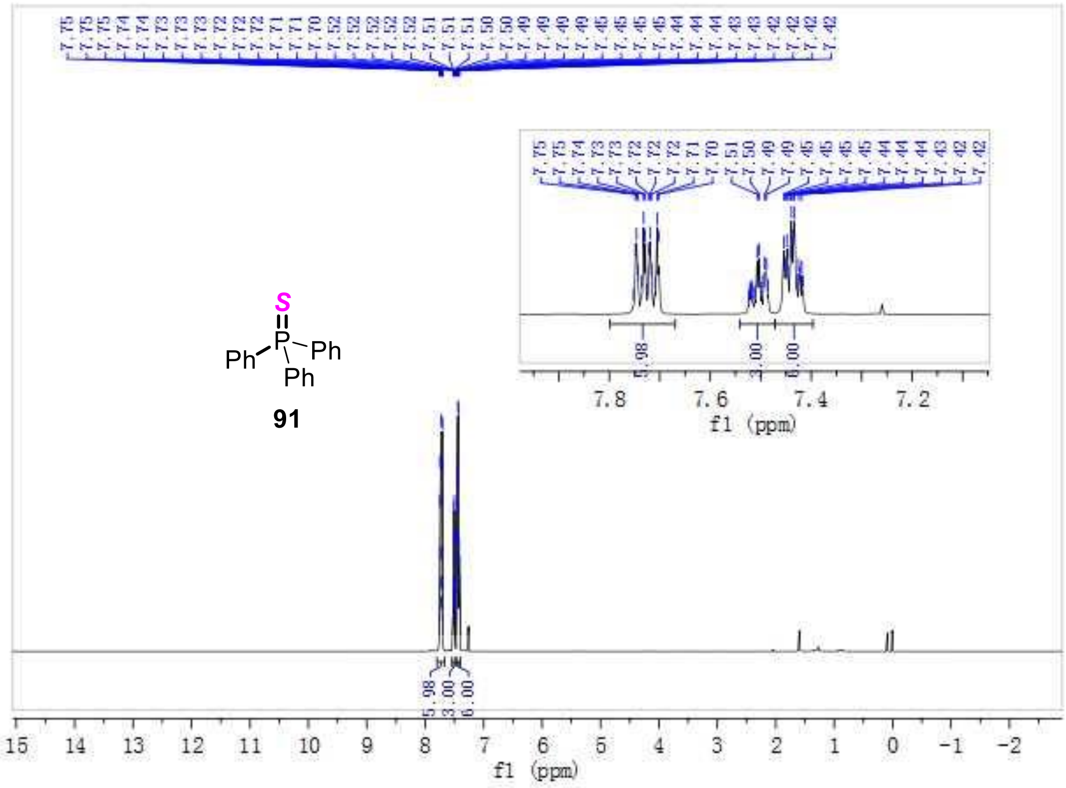

Supplementary Figure 222. ^{1}H NMR Spectrum of Compound **91**

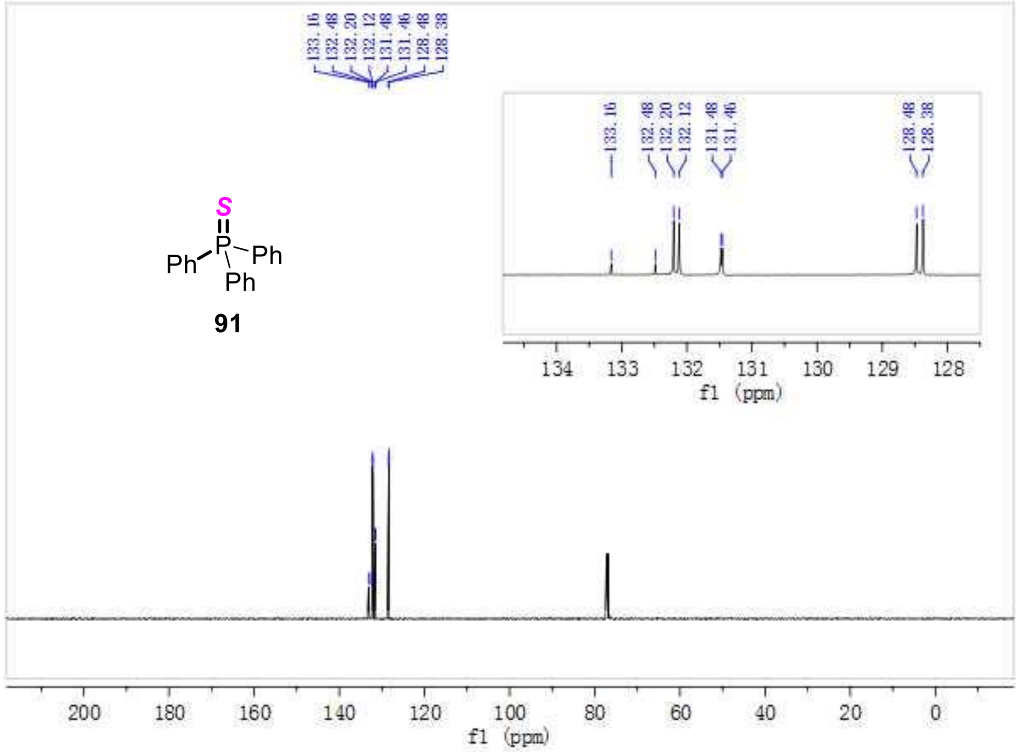

Supplementary Figure 223. ^{13}C NMR Spectrum of Compound **91**

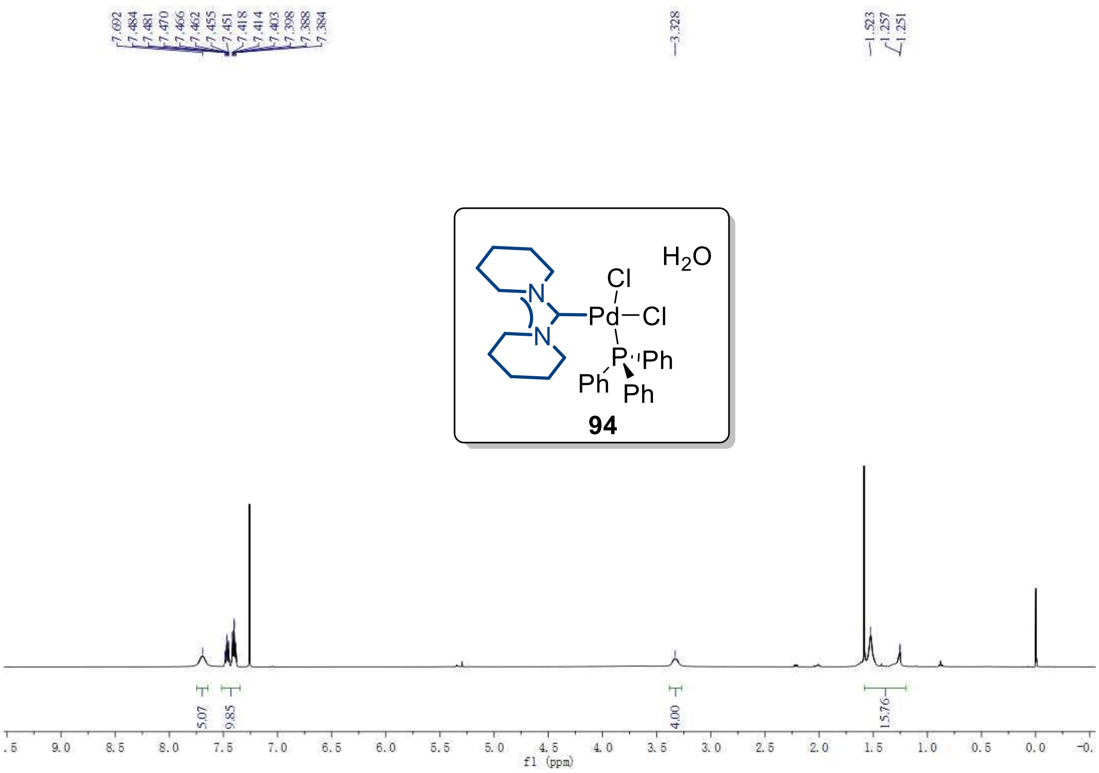

Supplementary Figure 224. ^{1}H NMR Spectrum of Compound **94**

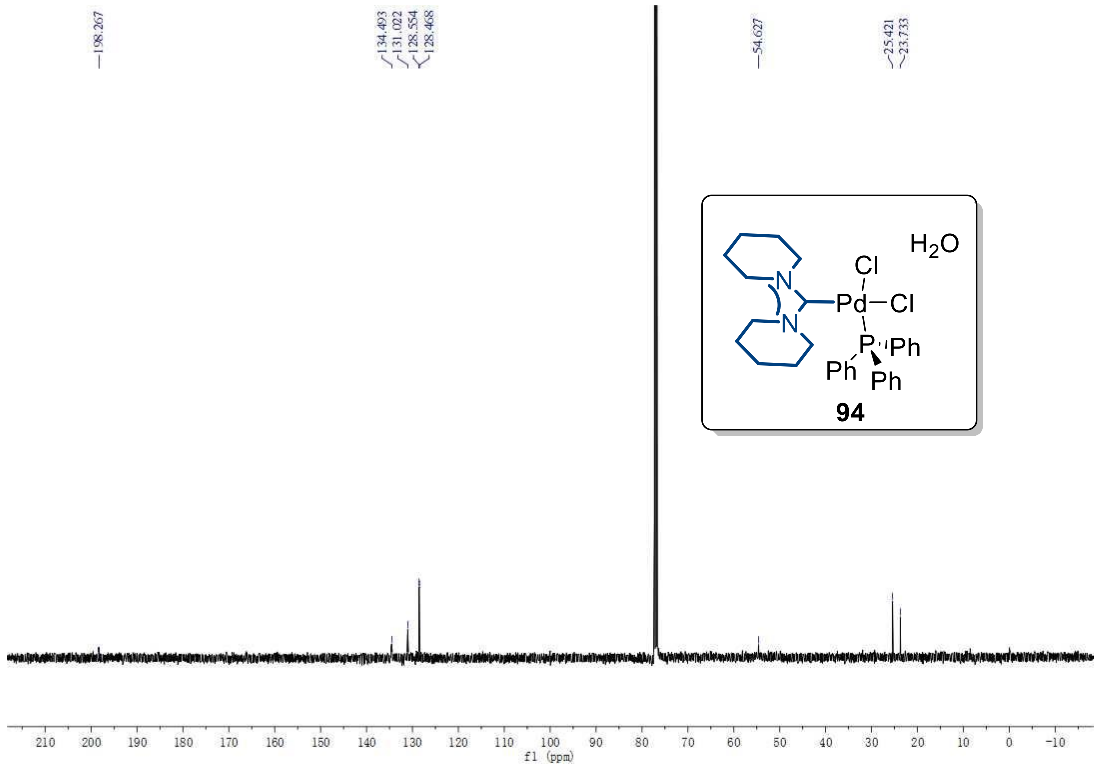

Supplementary Figure 225. ^{13}C NMR Spectrum of Compound **94**

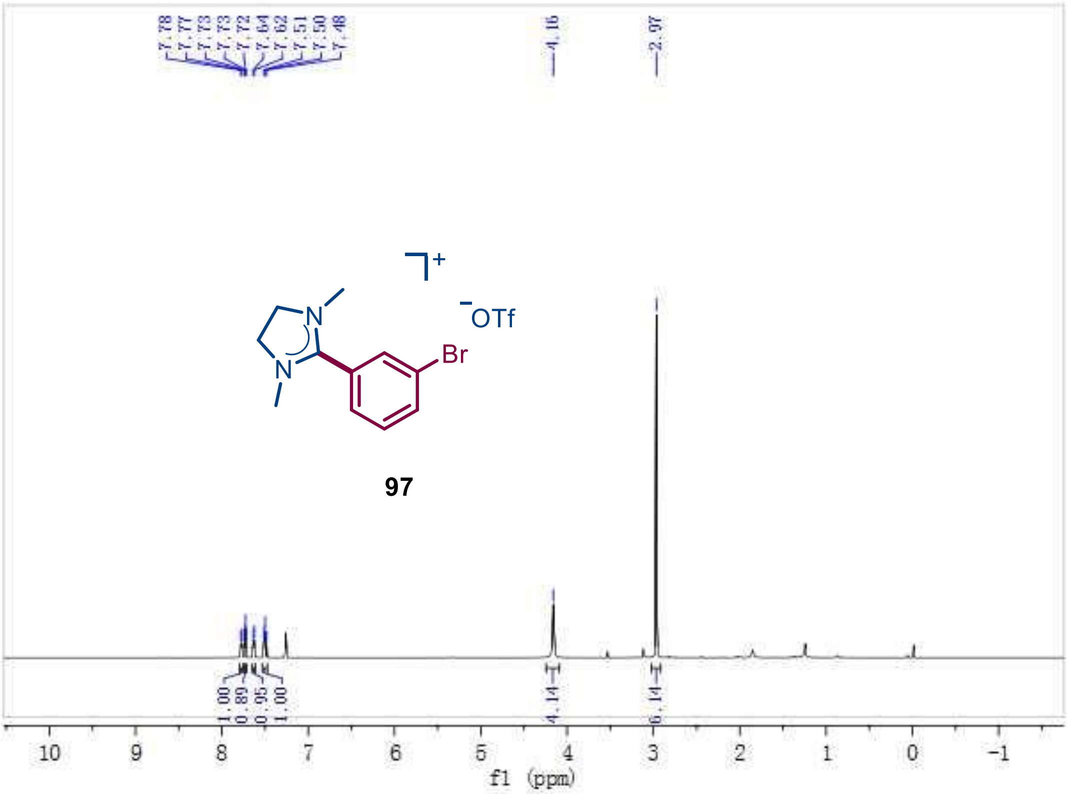

Supplementary Figure 226. ^{1}H NMR Spectrum of Compound **97**

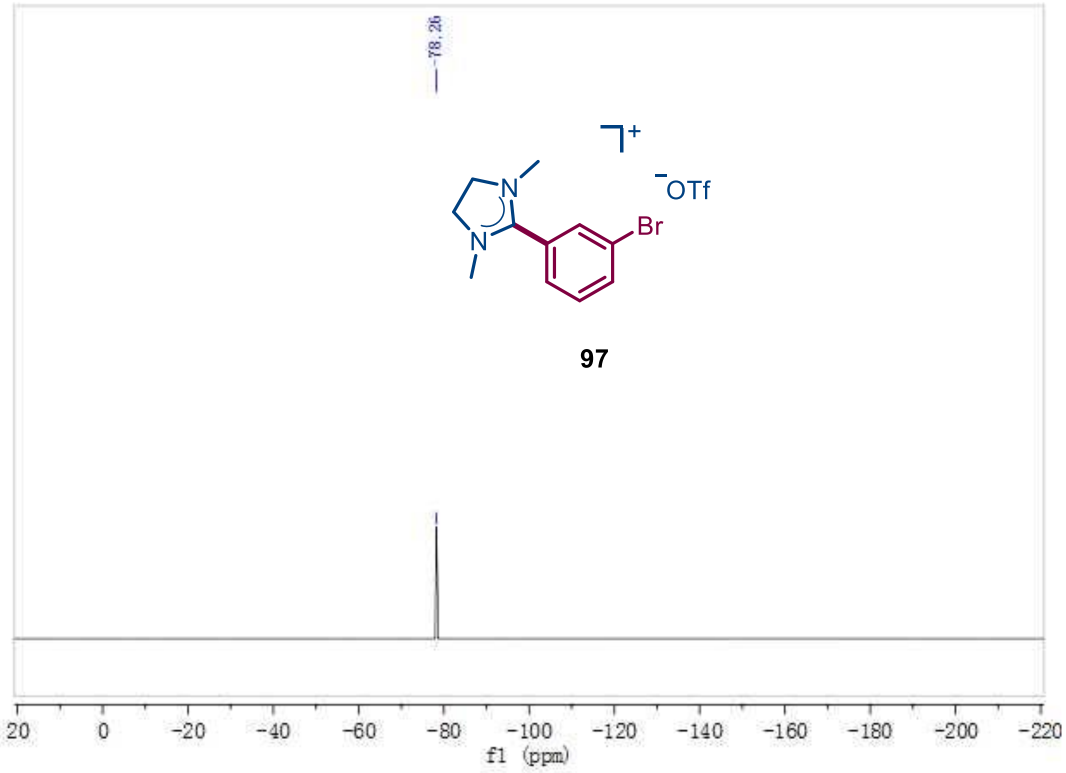

Supplementary Figure 227. ^{19}F NMR Spectrum of Compound **97**

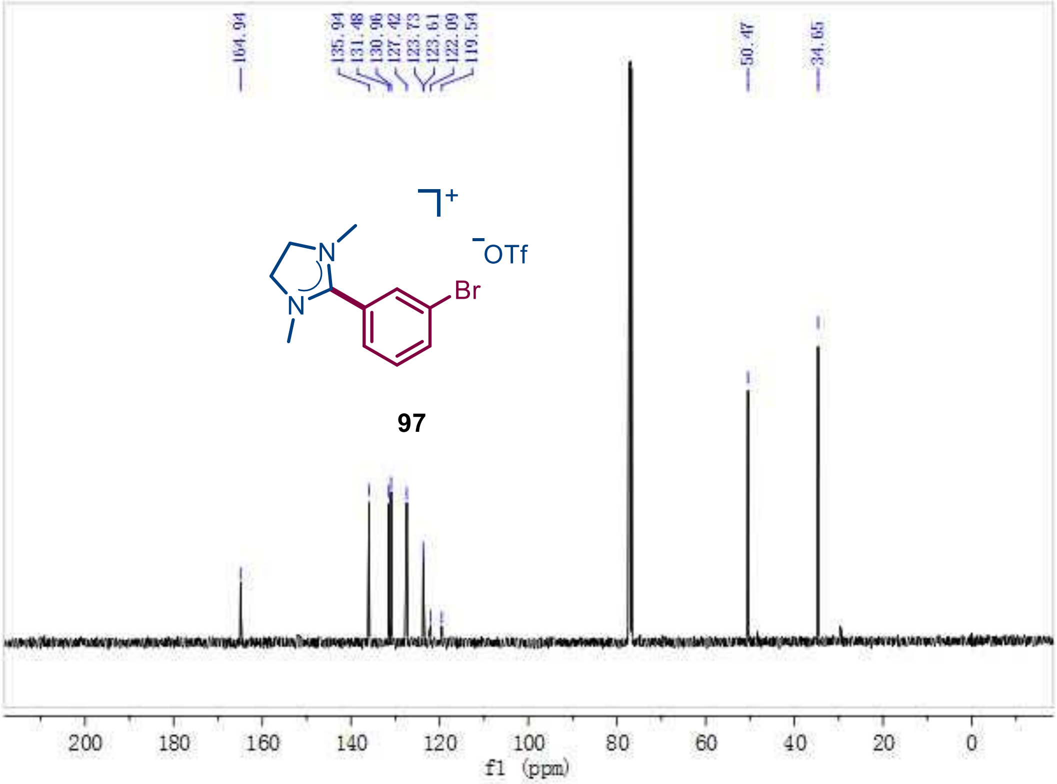

Supplementary Figure 228. ^{13}C NMR Spectrum of Compound **97**

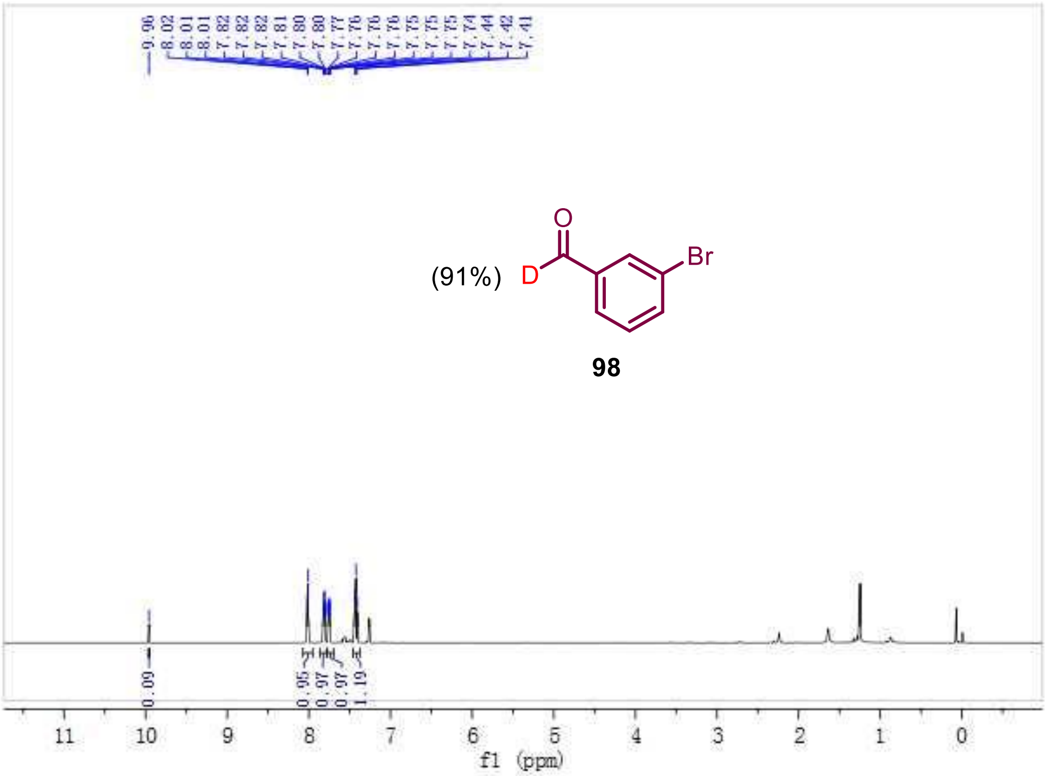

Supplementary Figure 229. ^{1}H NMR Spectrum of Compound **98**

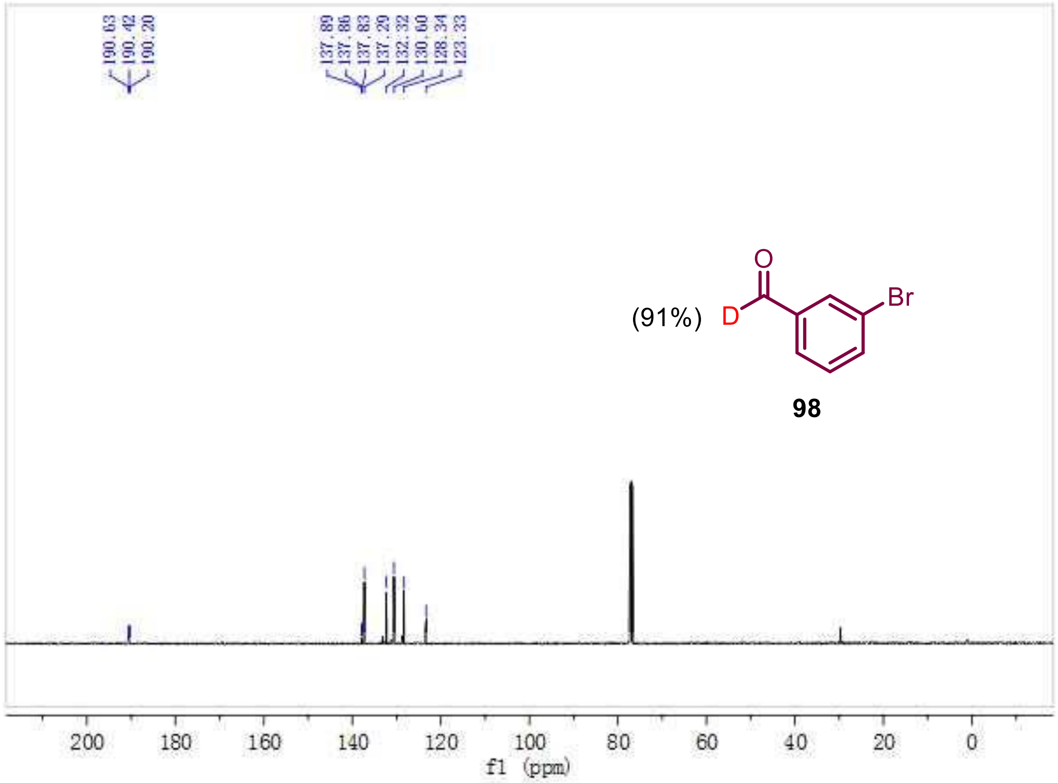

Supplementary Figure 230. ^{13}C NMR Spectrum of Compound **98**

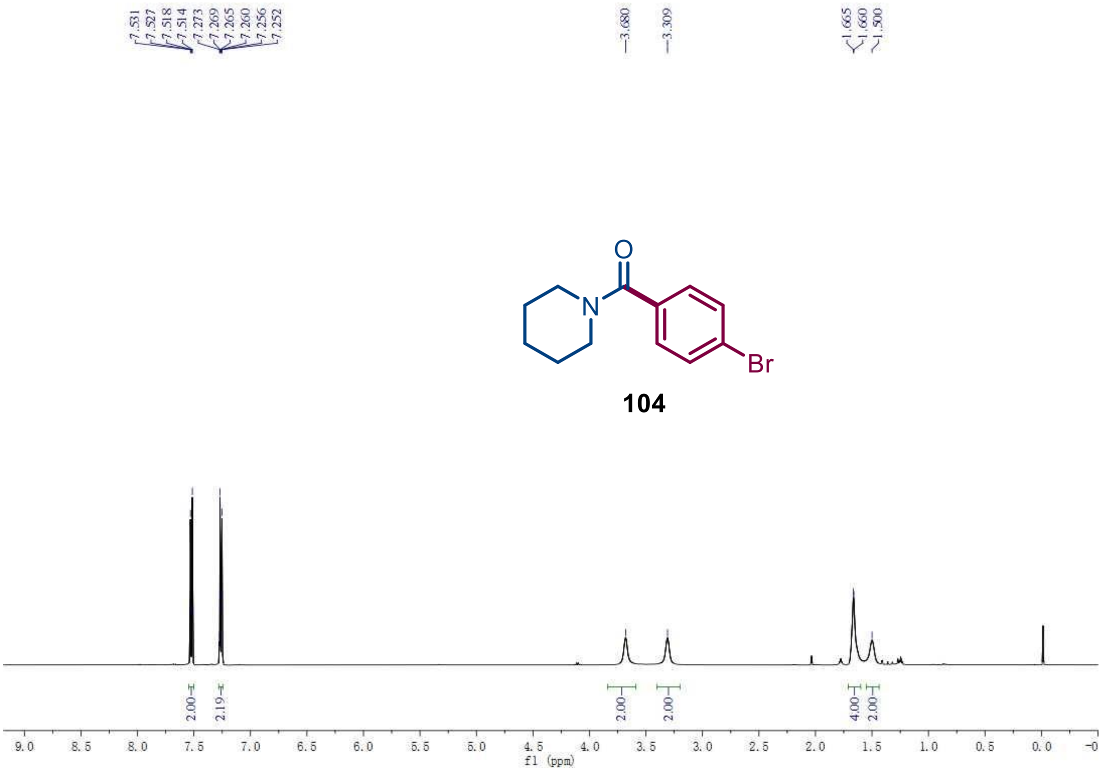

Supplementary Figure 231. ^{1}H NMR Spectrum of Compound **104**

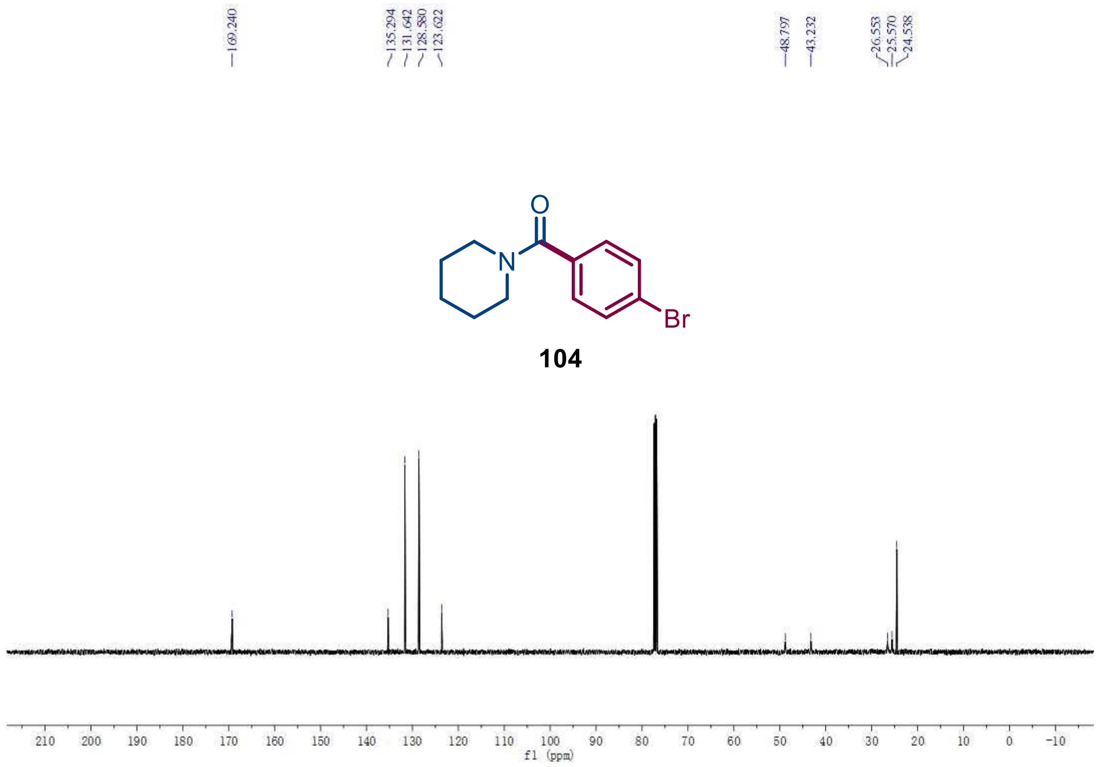

Supplementary Figure 232. ^{13}C NMR Spectrum of Compound **104**

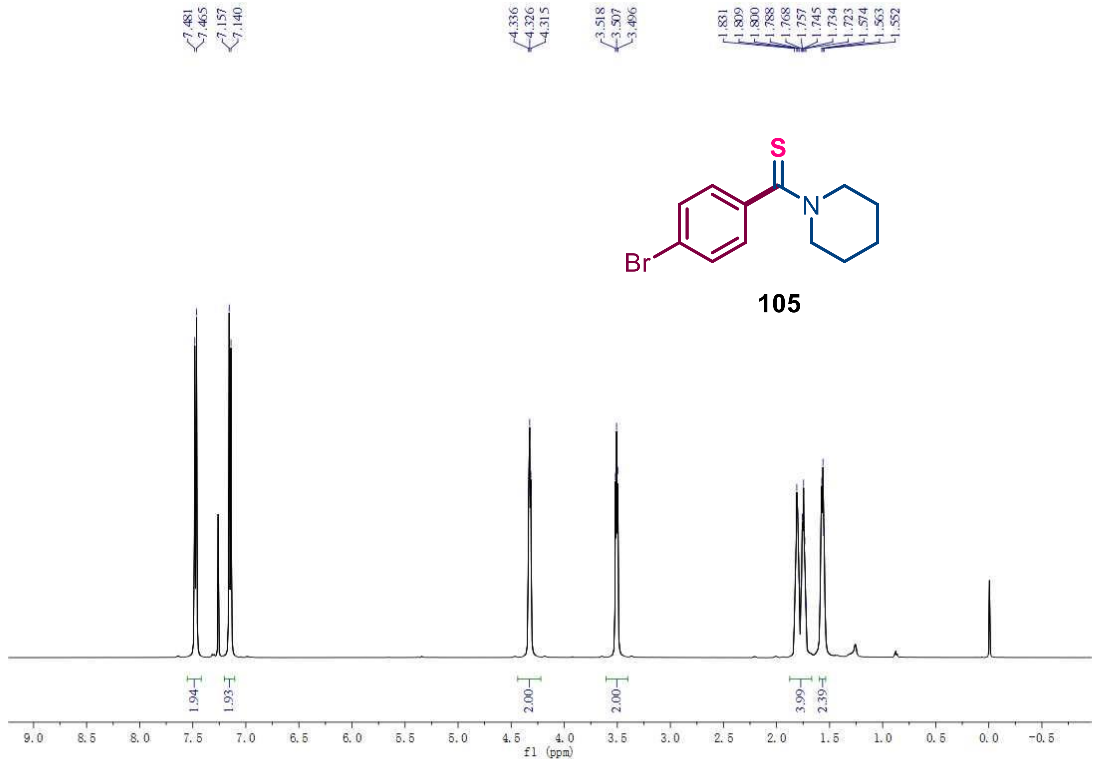

Supplementary Figure 233. ^{1}H NMR Spectrum of Compound **105**

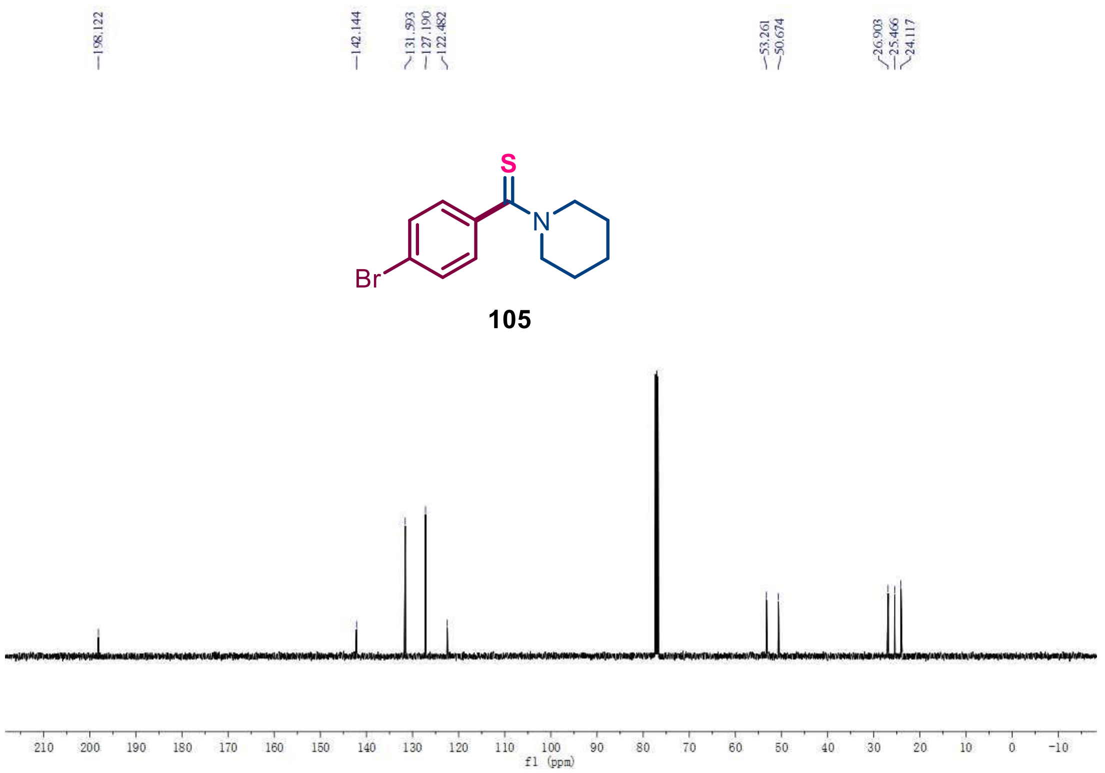

Supplementary Figure 234. ^{13}C NMR Spectrum of Compound **105**

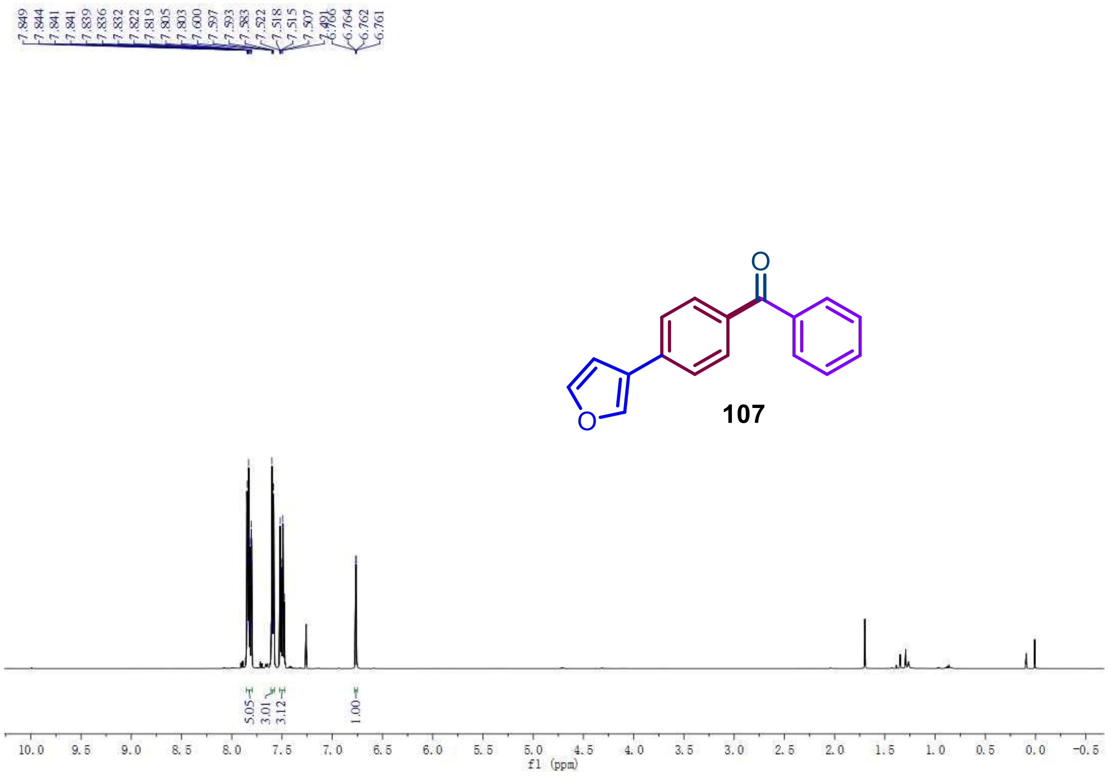

Supplementary Figure 235. ^{1}H NMR Spectrum of Compound **107**

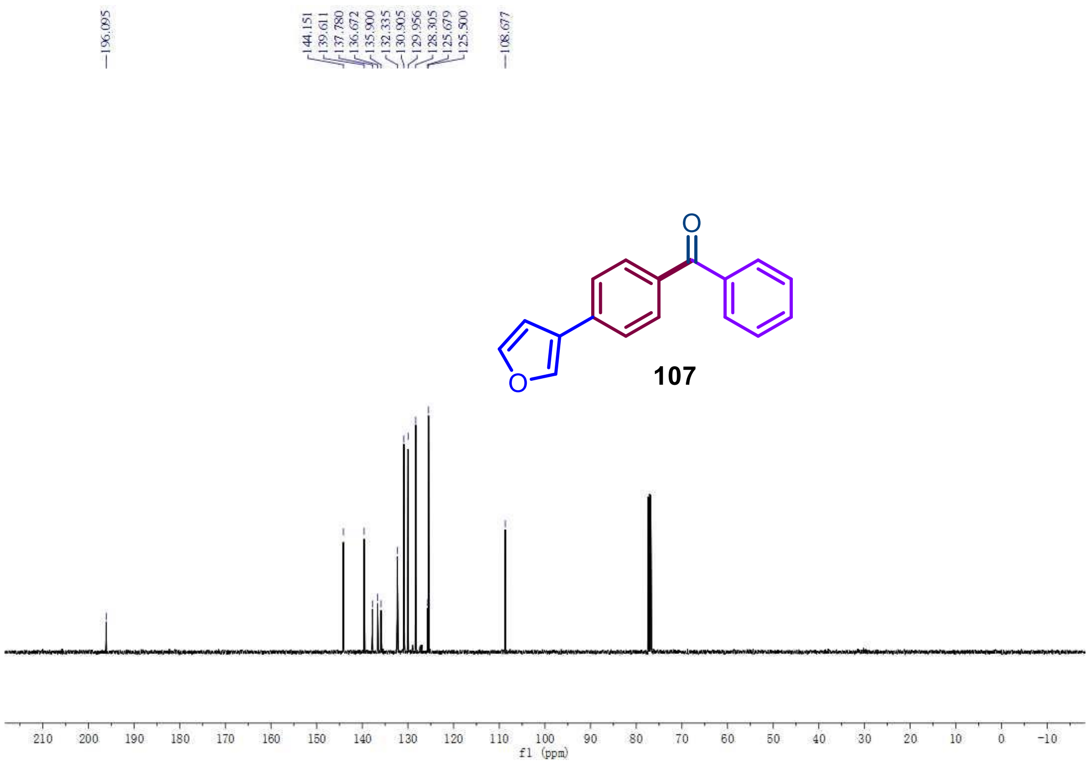

Supplementary Figure 236. ^{13}C NMR Spectrum of Compound **107**
